# Supplementary material for: Dihydroquinazolinones as adaptative C(sp3) handles in arylations and alkylations via dual catalytic C–C bond-functionalization
Source: Nat Commun. 2022 May 3;13:2394. doi: 10.1038/s41467-022-29984-0 (PMC9064991; doi:10.1038/s41467-022-29984-0)
Supplement: Supplementary file 1 — Supporting information [file 41467_2022_29984_MOESM1_ESM.pdf]

Supplementary Information for

Dihydroquinazolinones as Adaptative C(sp^3) Handles in Arylations and Alkylations via Dual Catalytic C–C Bond-Functionalization

Xin-Yang Lv,[†£] Roman Abrams,[†£] and Ruben Martin[†§]*

[†]*Institute of Chemical Research of Catalonia (ICIQ), Av. Països Catalans 16, 43007 Tarragona, Spain; Fax: (+34) 977-920- 222, E-mail: rmartinromo@iciq.es*

[£]*Universitat Rovira i Virgili, Departament de Química Analítica i Química Orgànica, c/Marcel·lí Domingo, 1, 43007 Tarragona, Spain*

[§]*Catalan Institution for Research and Advanced Studies (ICREA), Passeig Lluïs Companys, 23,08010, Barcelona, Spain*

Table of Contents

Supplementary Methods 2

1. General Considerations 2
2. General Procedures 4
3. Reaction Optimization 7
4. Synthesis of Starting Materials 9
5. Synthesis of Products 31
5.1 Scope Limitations 65
6. Telescoping the formation of Quinazolinones en route to *sp*^{3} Architectures 67
7. Mechanistic Studies 69
7.1 Experiments with a well-defined nickel complex 69
7.2 UV-Vis. Spectroscopy 74
7.3 Fluorescence Quenching Studies 75
7.4 Cyclic Voltammetry Analysis 80
7.5 Radical Clock Experiments 84
7.6 Radical Cyclisation as a Function of Catalyst Loading 86
7.7 TEMPO Radical Probe Experiments 90
7.8 X-ray diffraction of Ni-I 92
8. NMR Spectra 104

Supplementary References 198

Supplementary Methods

1. General Considerations

Analytical methods. ^{1}H and ^{13}C NMR spectra were recorded on Bruker 400 MHz or 500 MHz at 20 °C. All ^{1}H NMR spectra are reported in parts per million (ppm) downfield of TMS and were calibrated using the residual water peak (3.33 ppm in DMSO-d_6, 1.56 ppm in DMSO), residual solvent peak of $CHCl_3$ (7.26 ppm) or DSMO (2.50 ppm), unless otherwise indicated. All ^{13}C NMR spectra are reported in ppm relative to TMS, were calibrated using the signal of residual $CHCl_3$ (77.16 ppm) or DMSO (39.52), ^{19}F NMR was obtained with ^{1}H decoupling unless otherwise indicated. Coupling constants, J are reported in hertz (Hz). Melting points were measured using open glass capillaries in a Büchi B540 apparatus, with samples crystallized by slow evaporation of DCM unless the stated purification was by recrystallization. Infrared spectra (FT-IR) measurements were carried out on a Bruker Optics FT-IR Alpha spectrometer equipped with a DTGS detector, KBr beamsplitter at 4 cm^{-1} resolution using a one bounce ATR accessory with diamond windows. Mass spectra were recorded on a Waters LCT Premier spectrometer or in a MicroTOF Focus, Bruker Daltonics spectrometer. UV/Vis absorption spectra were recorded using an Agilent Technologies Cary 300 UV/Vis spectrophotometer and UV-1800PC spectrophotometer in quartz cuvettes with a path length of 1.0 cm. Bulk electrolysis was conducted on a PARSTAT 2273 potentiometer using a 3-electrode cell configuration at room temperature. The same electrodes were used as for CV experiments, namely a glassy carbon working electrode, platinum flag counter electrode and Ag/AgCl (KCl sat.) reference electrode. Flash chromatography was performed with EM Science silica gel 60 (230-400 mesh). Thin layer chromatography was used to monitor reaction progress and analysed fractions from column chromatography. To this purpose TLC Silica gel 60 F_{254} aluminium sheets from Merck were used and visualization was achieved using UV irradiation and/or staining with potassium permanganate or cerium molybdate solution. The procedures described in this section are representative. Thus, the yields may differ slightly from those given in the tables of the manuscript.

Reagents. Commercially available aryl bromides (**1, 4a-n**) and alkyl bromides (**7a-d, 7 f, 7h**) were used as received without further purification. $Ni(OAc)_2{\cdot}4H_2O$ (98% purity) was purchased from Aldrich. $NiBr_2{\cdot}$diglyme (97% purity) was purchased from Aldrich. 4,4',4"-Tri-tert-Butyl-2,2':6',2"-terpyridine (95 % purity) was purchased from Aldrich. 2,6-Di(1-pyrazolyl)pyridine (>98% purity) was purchased from TCI. Anhydrous Na_2CO_3

(99.5 % purity) was purchased from PanReac. NaBr (>99 % purity) was purchased from Across. $NaHCO_3$ (>99% purity) was purchased from Fisher. Anhydrous 1-Methyl-2-pyrrolidinone (NMP, 99.5% purity) and DMF (99.8% purity) were purchased from Across.

Photoreactor. Arylation and alkylation reactions were performed with 451 nm LEDs (OSRAM Oslon® SSL 80 royal- blue LEDs), which were installed at the bottom of a custom-made 8 flat-bottom Schlenk tubes holder (the distance between the flat-bottom Schlenk tube and the light source was measured to be ~7 mm), equipped with chiller cooling system (the thermostat was set at 40 °C) and a magnetic stirrer (1000 rpm)

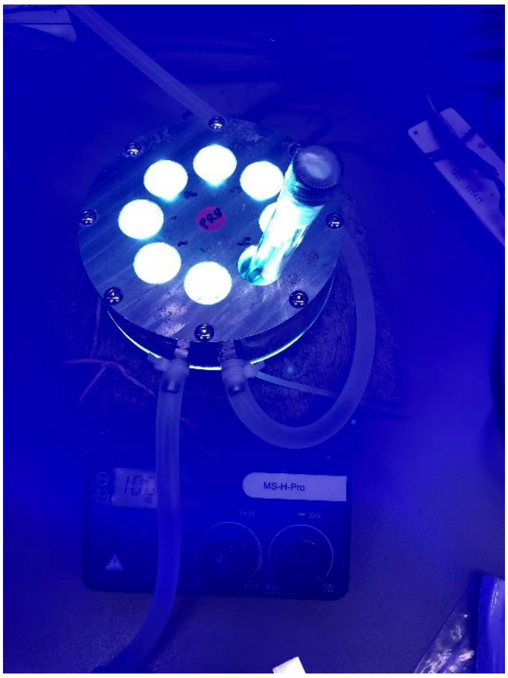

2. General Procedures

General procedure 1 (GP1): Synthesis of aryl bromides and alkyl bromides

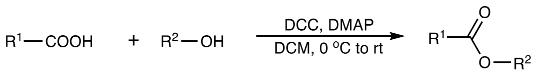

To a stirred solution of carboxylic acid (1.2 equiv.) and alcohol (1.0 equiv.) in DCM (0.5 M) was added DMAP (0.8 equiv.) and DCC (1.5 equiv.) at 0 °C. The reaction mixture was allowed to stir at 20 °C for 20 hours. The reaction mixture was filtered through silica gel and the precipitate was washed with DCM. The received crude material was concentrated under reduced pressure and purified by silica gel chromatography to yield the desired product.

General procedure 2 (GP2): Synthesis of 2,2-disubstituted dihydroquinazolinones

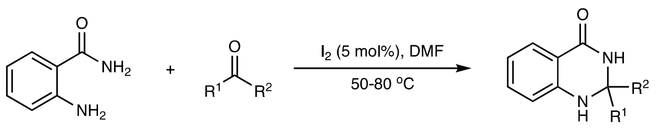

A 100 mL flask containing a stirring bar was charged with 2-aminobenzamide (1.0 equiv.), ketone (1.05 equiv.), iodine (5 mol%) and DMF (0.67 M). The reaction mixture was stirred at 50-80 °C for 24 hours. The reaction was cooled to 20 °C and water (50 mL) was added to the reaction generating precipitate that was collected as crude product by suction filtration. The crude material was washed with water and purified by recrystallization (EtOH) to give targeted product.

General procedure 3 (GP3): Alternative synthesis of 2,2-disubstituted dihydroquinazolinones

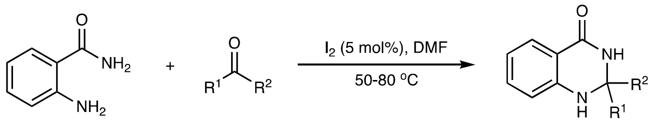

A 100 mL flask containing a stirring bar was charged with 2-aminobenzamide (1.0 equiv.), ketone (1.05 equiv.), iodine (5 mol%) and DMF (0.67 M). The reaction mixture was stirred at 50-80 °C for 24 hours. The reaction was cooled to 20 °C, quenched with 10% $Na_2S_2O_3$(aq) (50 mL) and extracted using EtOAc (2 x 50 mL). The combined organic extracts were dried (Na_2SO_4) and concentrated under reduced pressure affording crude material, which was purified by silica gel chromatography yielding the desired product.

General procedure 4 (GP4): Nickel-catalyzed coupling with aryl bromides

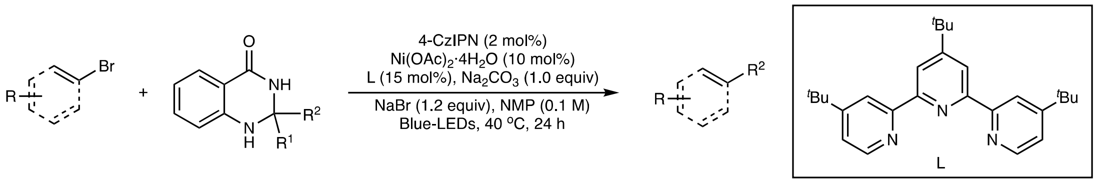

An oven-dried 8 mL screw-cap test tube containing a stirring bar was charged with 4-CzIPN (3.2 mg, 2 mol%), $Ni(OAc)_2\cdot 4H_2O$ (5.0 mg, 10 mol%), 4,4',4''-tri-tert-butyl-2,2':6'2''- terpyridine (12.1 mg, 15 mol%), NaBr (24.7 mg, 1.2 equiv.), aryl bromide (if solid, 1.0 equiv., 0.2 mmol) and ketone derivative (1.2 equiv.). The test tube was introduced in a nitrogen-filled glovebox where Na_2CO_3 (21.2 mg, 1.0 equiv.) was added. The reaction vessel was sealed with a screw cap and removed from the glovebox. Afterwards, aryl bromide (if liquid) or vinyl bromide and NMP (2 mL, 0.1 M) were added by syringe. Parafilm was used to reseal the pierced cap. The reaction mixture was stirred at rt for 1 minute, then exposed to blue LED irradiation at 40 °C for 24 hours. The reaction mixture was quenched with water/brine (10 mL) and extracted with ethyl acetate (3 x 10 mL). The combined organic extracts were dried (Na_2SO_4), concentrated under reduced pressure and purified by silica gel chromatography.

General procedure 5 (GP5): Alternative nickel-catalyzed coupling with aryl bromides

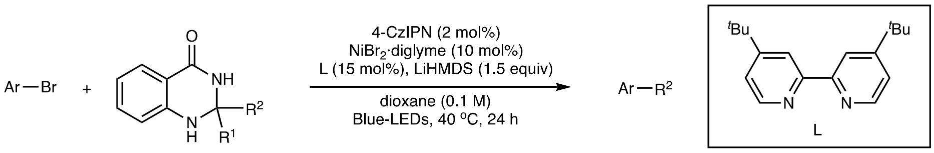

An oven-dried 8 mL screw-cap test tube containing a stirring bar was charged with 4-CzIPN (3.2 mg, 2 mol%), 4,4'-di-*tert*-butyl-2,2'-bipyridine (8.0 mg, 15 mol%), aryl bromide (if solid, 1.0 equiv., 0.2 mmol) and ketone derivative (1.5 equiv.). The test tube was taken into a nitrogen-filled glovebox where $NiBr_2\cdot$diglyme (7.1 mg, 10 mol%) and LiHMDS (50.1 mg, 1.5 equiv.) were added to the reaction vessel. The reaction tube was sealed with a screw cap and removed from the glovebox. Afterwards, aryl bromide (if liquid) and dioxane (2 mL, 0.1 M) were added by syringe. Parafilm was used to reseal the pierced cap. The reaction mixture was stirred at rt for 1 minute, then exposed to blue LED irradiation at 40 °C for 24 hours. The reaction mixture was quenched with water/brine (10 mL) and extracted with ethyl acetate (3 x 10 mL). The combined organic extracts

were dried (Na_2SO_4), concentrated under reduced pressure and purified by silica gel chromatography.

General procedure 6 (GP6): Nickel-catalyzed coupling with alkyl bromides

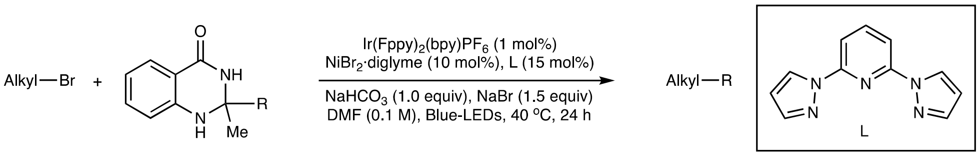

An oven-dried 8 mL screw-cap test tube containing a stirring bar was charged with $Ir(Fppy)_2(bpy)PF_6$ (1.6 mg, 1 mol%), 2,6-di(1-pyrazolyl)pyridine (12.1 mg, 15 mol%), NaBr (30.9 mg, 1.5 equiv.), alkyl bromide (if solid, 1.0 equiv., 0.2 mmol) and ketone derivative (1.5 equiv.). The test tube was introduced in a nitrogen-filled glovebox where $NiBr_2$·diglyme (7.1 mg, 10 mol%) and $NaHCO_3$ (16.8 mg, 1.0 equiv.) were added to the reaction vessel. The reaction tube was sealed with a screw cap and removed from the glovebox. Afterwards, alkyl bromide (if liquid) and DMF (2 mL, 0.1 M) were added by syringe. Parafilm was used to reseal the pierced cap. The reaction mixture was stirred at rt for 1 minute, then exposed to blue LED irradiation at 40 °C for 24 hours. The reaction mixture was quenched with water/brine (10 mL) and extracted with ethyl acetate (3 x 10 mL). The combined organic extracts were dried (Na_2SO_4), concentrated under reduced pressure and purified by silica gel chromatography.

3. Reaction Optimization

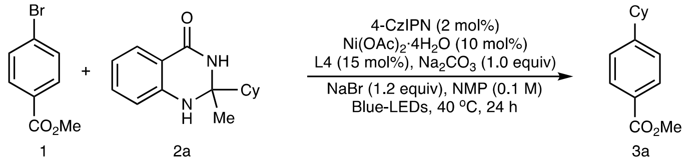

An 8 mL screw-cap test tube containing a stirring bar was charged with 4-CzIPN (1.6 mg, 2 mol%), $Ni(OAc)_2{\cdot}4H_2O$ (2.5 mg, 10 mol%), 4,4',4"-tri-tert-butyl-2,2':6'2"- terpyridine (6.0 mg, 15 mol%), NaBr (12.4 mg, 1.2 equiv.), methyl 4-bromobenzoate (21.5 mg, 0.1 mmol) and 2-cyclohexyl-2-methyl-2,3-dihydroquinazolin-4(1*H*)-one (29.3 mg, 1.2 equiv.). The test tube was introduced to a nitrogen-filled glovebox and charged with Na_2CO_3 (10.6 mg, 1.0 equiv.). The test tube was sealed with a screw cap and removed from the glovebox, after which NMP (1.0 mL, 0.1 M) was added by syringe and the cap was resealed with parafilm. The reaction mixture was stirred at rt for 1 minute then exposed to blue LED irradiation at 40 °C and allowed to stir for 24 hours. The reaction mixture was analyzed by GC using dodecane (17.0 mg, 0.1 mmol) as internal standard.

Entry	Deviation from standard conditions	Yield (%)[b]
1	none	99 (93)[c]
2	No Ligand	0
3	**L1** instead of **L4**	36
4	**L2** instead of **L4**	<1
5	**L3** instead of **L4**	37
6	**L5** instead of **L4**	<1
7	**L6** instead of **L4**	<1
8	**L7** instead of **L4**	9
9	No Ni catalyst	0
10	Using $NiBr_2$·diglyme	83
11	Using $Ni(COD)_2$	61
12	Using $NiCl_2$·DME	92
13	Using DMF	88
14	Using MeCN	63
15	No PC	0
16	Using $Ir(ppy)_2(dtbpy)PF_6$	99
17	Using $Ir[dF(CF_3)ppy]_2(dtbpy)PF_6$	69
18	K_3PO_4 instead of Na_2CO_3	81
19	$NaHCO_3$ instead of Na_2CO_3	81
20	KBr instead of NaBr	94
21	NaCl instead of NaBr	93
22	No light (40 °C)	0

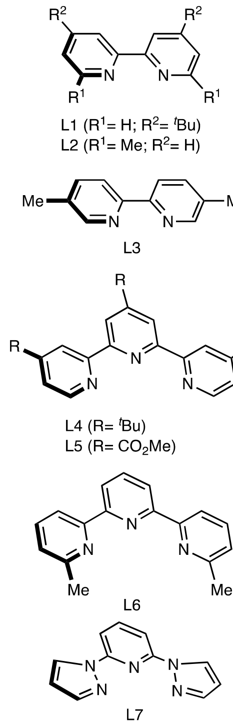

Supplementary Table 1. Conditions: [a] **1a** (0.10 mmol), **2a** (0.12 mmol), $Ni(OAc)_2\cdot 4H_2O$ (10 mol %), **L1** (15 mol %), 4-CzIPN (2 mol %), NaBr (0.12 mmol), Na_2CO_3 (0.10 mmol), in NMP (0.10 M) at 40 °C under irradiation of Blue-LEDs for 24 hours. [b] Yields were determined by GC using dodecane as internal standard. [c] Isolated yield. [d] The addition of NaBr was required for inhibiting dehalogenation product, a necessary goal to be able to purify the corresponding product.

4. Synthesis of Starting Materials

2-Cyclohexyl-2-methyl-2,3-dihydroquinazolin-4(1*H*)-one (2a)

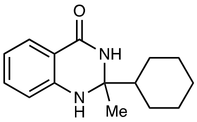

Following GP2, using 2-aminobenzamide (1.36 g, 10.0 mmol), 1-cyclohexylethan-1-one (1.32 g, 10.5 mmol) and iodine (0.13 g, 0.5 mmol) in DMF (15 mL) at 80 °C for 24 hours. The product was obtained as a white solid (1.61 g, 66% yield). **M.p.**: 215 °C. **^{1}H NMR (400 MHz, DMSO-*d*$_6$)** δ 7.87 (s, 1H), 7.52 (dd, *J* = 7.7, 1.6 Hz, 1H), 7.18 (ddd, *J* = 8.2, 7.1, 1.7 Hz, 1H), 6.66 (d, *J* = 8.1, 1H), 6.59 (s, 1H), 6.55 (ddd, *J* = 7.9, 7.2, 1.1 Hz, 1H), 1.83 – 1.66 (m, 4H), 1.63 – 1.49 (m, 2H), 1.30 (s, 3H), 1.14 – 0.95 (m, 5H) ppm. **^{13}C NMR (101 MHz, DMSO-*d*$_6$)** δ 162.8, 147.0, 133.2, 127.0, 115.7, 113.6, 113.5, 71.2, 47.8, 26.6, 26.3, 26.0, 25.9, 25.9, 24.8 ppm. Spectroscopic data was in agreement with the literature.[1]

2-Cyclohexyl-2-ethyl-2,3-dihydroquinazolin-4(1*H*)-one (2a´)

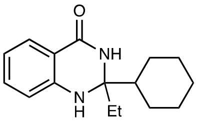

Following GP2, using 2-aminobenzamide (1.36 g, 10.0 mmol), 1-cyclohexylpropan-1-one (1.47 g, 10.5 mmol) and iodine (0.13 g, 0.5 mmol) in DMF (15 mL) at 80 °C for 24 hours. The product was obtained as a white solid (1.68 g, 60% yield). **M.p.**: 172 °C. **IR (neat, cm^{-1}):** 3370, 3184, 2934, 2858, 1636, 1611, 743. **^{1}H NMR (500 MHz, DMSO-*d*$_6$)** δ 7.67 (s, 1H), 7.49 (dd, *J* = 7.7, 1.5 Hz, 1H), 7.13 (ddd, *J* = 8.6, 7.2, 1.6 Hz, 1H), 6.64 (d, *J* = 8.2 Hz, 1H), 6.48 (td, *J* = 7.4, 1.0 Hz, 1H), 6.43 (s, 1H), 1.77 – 1.67 (m, 4H), 1.58 (q, *J* = 7.3, 2H), 1.54 – 1.47 (m, 1H), 1.19 – 0.97 (m, 6H), 0.87 (t, *J* = 7.2 Hz, 3H). **^{13}C NMR (126 MHz, DMSO-*d*$_6$)** δ 163.6, 148.2, 133.6, 127.4, 115.5, 113.6, 113.1, 74.4, 48.4, 26.8, 26.5, 26.5, 26.5, 8.3. **HRMS** [ESI$^+$] *calcd.* for ($C_{16}H_{23}N_2O$) $[M+H]^+$: 259.1805, *found*: 259.1798.

2-Cyclopentyl-2-methyl-2,3-dihydroquinazolin-4(1*H*)-one (2b)

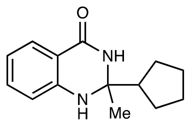

Following GP2, using 2-aminobenzamide (1.16 g, 8.5 mmol), 1-cyclopentylethan-1-one (1.00 g, 8.9 mmol) and iodine (0.11 g, 0.4 mmol) in DMF (12 mL) at 80 °C for 24 hours. The product was obtained as a white solid (0.91 g, 46% yield). **M.p.**: 190 °C. **IR (neat, cm^{-1}):** 3308, 3179, 2951, 2868, 1632, 1482, 1150, 754. **^{1}H NMR (400 MHz, DMSO-*d*$_6$)** δ 7.85 (s, 1H), 7.52 (dd, *J* = 7.7, 1.7 Hz, 1H), 7.17 (ddd, *J* = 8.1, 7.1, 1.7 Hz, 1H), 6.65 (dd, *J* = 8.2, 0.7 Hz, 1H), 6.58 – 6.52 (m, 1H), 6.51 (s, 1H), 2.25 – 2.13 (m, 1H), 1.61 – 1.37 (m, 8H), 1.33 (s, 3H) ppm. **^{13}C NMR (101 MHz, DMSO-*d*$_6$)** δ 162.9, 147.3, 133.1, 126.9, 115.7, 113.6, 113.4, 70.8, 50.8, 26.8, 26.6, 26.5, 25.5, 25.2 ppm. **HRMS** [ESI$^+$] *calcd*. for ($C_{14}H_{18}N_2NaO$) [M+Na]$^+$: 253.1317, *found*: 253.1317.

2-Cycloheptyl-2-methyl-2,3-dihydroquinazolin-4(1*H*)-one (2c)

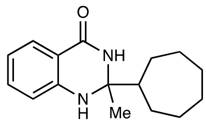

Following GP2, using 2-aminobenzamide (1.36 g, 10.0 mmol), 1-cycloheptylethan-1-one (1.47 g, 10.5 mmol) and iodine (0.13 g, 0.5 mmol) in DMF (15 mL) at 80 °C for 24 hours. The product was obtained as a white solid (1.54 g, 60% yield). **M.p.**: 198 °C. **IR (neat, cm^{-1}):** 3309, 3177, 2915, 2851, 1630, 1516, 1279, 1150, 749. **^{1}H NMR (400 MHz, DMSO-*d*$_6$)** δ 7.97 (s, 1H), 7.54 (dd, *J* = 7.7, 1.7 Hz, 1H), 7.19 (ddd, *J* = 8.1, 7.2, 1.7 Hz, 1H), 6.70 – 6.63 (m, 2H), 6.61 – 6.53 (m, 1H), 1.86 – 1.71 (m, 3H), 1.72 – 1.55 (m, 2H), 1.54 – 1.38 (m, 4H), 1.36 – 1.18 (m, 7H) ppm. **^{13}C NMR (101 MHz, DMSO-*d*$_6$)** δ 162.8, 146.8, 133.1, 127.0, 115.8, 113.9, 113.8, 71.8, 47.9, 28.1, 27.7, 27.7, 27.5, 26.9, 26.7, 23.8 ppm. **HRMS** [ESI$^+$] *calcd.* for ($C_{16}H_{22}N_2NaO$) [M+Na]$^+$: 281.1630, *found*: 281.1627.

Synthesis of Methyl 2-((1*R*)-2,2-dimethyl-3-(2-methyl-4-oxo-1,2,3,4-tetrahydroquinazolin-2-yl)cyclobutyl)acetate (2d)

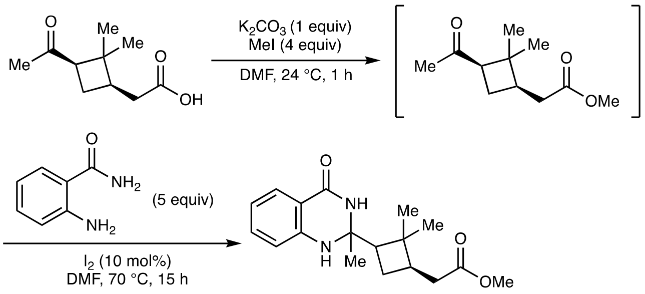

A 50 mL RBF was charged with *cis*-pinonic acid (500 mg, 2.7 mmol) and potassium carbonate (380 mg, 2.7 mmol), then equipped with a rubber septum with argon inlet. The reaction vessel was flushed with argon and then charged DMF (40 mL, anhydrous). The reaction mixture was stirred for an hour at 24 °C, after which iodomethane (0.67 mL, 10.8 mmol) was added and the reaction mixture was stirred for an additional 16 hours at 24 °C. The reaction mixture was quenched with saturated Na_2CO_3 (aq, 50 mL) and extracted with ethyl acetate (2 x 50 mL). The combined organic extracted were dried ($MgSO_4$) and concentrated under reduced pressure affording crude product as a a yellow oil. The crude material was filtered through a plug of silica (loaded using hexane, released using 20% EtOAc in Hexane) to afford a colourless oil as crude material (522 mg), which was used without further purification. A 10 mL RBF was containing a stirring bar was charged with the crude material of the previous step (552 mg), DMF (4 mL), 2-aminobenzamide (338 mg, 2.5 mmol) and iodine (30.4 mg, 0.12 mmol). The reaction mixture was stirred at 70 °C for 15 hours. Additional iodine (30.4 mg, 0.12 mmol) was added and the reaction mixture was stirred at 70 °C for 5 hours and then heated to 95 °C and stirred for 25 hours. The reaction mixture was cooled to 20 °C, quenched with 10% $Na_2S_2O_3$ (aq, 30 mL) and extracted using EtOAc (3 x 30 mL). The combined organic extracts were dried (Na_2SO_4) and concentrated under reduced pressure affording crude material, which was purified by silica gel chromatography (0 to 6% MeOH in DCM) yielding the title compound (224 mg, 28% over 2 step) as a colourless oil. **IR (film, cm^{-1}) ν_{max}** = 3297, 2952, 1723, 1651, 1612, 1486. **^{1}H NMR (500 MHz, $CDCl_3$, mixture of diasteromers A:B:C:D in a 0.41:0.34:0.13:0.12 ratio)** δ 7.85 – 7.78 (m, 1H), 7.29 – 7.21 (m, 1H), 6.62 (s, 0.34H), 6.60 – 6.49 (m, 1.25H), 6.29 (s, 0.41H), 3.65 – 3.58 (m, 3H), 2.50 – 2.41 (m, 0.25H), 2.37 – 2.27 (m, 1.16H), 2.26 – 1.91 (m, 3.65H), 1.77 – 1.69 (m, 0.13H), 1.65 – 1.53 (m, 0.87H), 1.48–1.42 (m, 3H), 1.28 (s, 0.36H), 1.27 (s, 0.39H), 1.16 (s, 1.15H), 1.13 (s,

1.32H), 1.12 (s, 2.18H), 1.03 (s, 0.39H), 1.02 (s, 0.36H) ppm. **^{13}C NMR (126 MHz, $CDCl_3$)** δ 173.6, 173.6, 173.4, 173.4, 164.3, 164.2, 164.2, 146.1, 146.0, 146.0, 145.9, 134.2, 134.2, 134.1, 134.1, 128.3, 128.3, 128.2, 118.4, 118.4, 118.4, 118.4, 114.2, 114.1, 114.1, 114.1, 71.4, 71.3, 71.0, 70.9, 53.9, 53.1, 51.7, 51.6, 51.6, 51.4, 42.5, 42.4, 40.3, 40.2, 38.6, 38.3, 38.2, 37.9, 35.5, 35.4, 34.7, 34.6, 31.3, 31.2, 28.3, 28.3, 28.1, 28.1, 26.2, 26.1, 25.6, 25.0, 24.9, 24.5, 24.1, 17.8, 17.7 ppm. **HRMS** [ESI$^+$] *calcd.* for ($C_{18}H_{24}N_2NaO_3$) [M+Na]$^+$: 339.1679, *found* 339.1690.

2-Methyl-2-(tetrahydro-2*H*-pyran-4-yl)-2,3-dihydroquinazolin-4(1*H*)-one (2e)

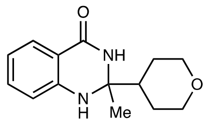

Following GP2, using 2-aminobenzamide (1.36 g, 10.0 mmol), 1-(tetrahydro-2*H*-pyran-4-yl)ethan-1-one (1.35 g, 10.5 mmol) and iodine (0.13 g, 0.5 mmol) in DMF (15 mL) at 80 °C for 24 hours. The product was obtained as a white solid (1.00 g, 41% yield). **M.p.**: 196 °C. **IR (neat, cm^{-1}):** 3355, 3170, 2968, 2840, 1631, 1504, 1271, 1149, 1095, 753. **^{1}H NMR (400 MHz, DMSO-d_6)** δ 7.92 (s, 1H), 7.53 (dd, J = 7.7, 1.6 Hz, 1H), 7.18 (ddd, J = 8.2, 7.1, 1.7 Hz, 1H), 6.66 (d, J = 8.0 Hz, 1H), 6.63 (s, 1H), 6.59 – 6.52 (m, 1H), 3.92 – 3.81 (m, 2H), 3.21 – 3.08 (m, 2H), 1.83 – 1.71 (m, 1H), 1.61 – 1.47 (m, 2H), 1.42 – 1.25 (m, 5H) ppm. **^{13}C NMR (101 MHz, DMSO-d_6)** δ 162.9, 147.0, 133.3, 127.0, 115.9, 113.6, 113.4, 70.7, 67.1, 67.0, 45.5, 26.8, 26.6, 24.9 ppm. **HRMS** [ESI$^+$] *calcd.* for ($C_{14}H_{18}N_2NaO_2$) [M+Na]$^+$: 269.1266, *found*: 269.1269.

***tert*-Butyl 4-(2-methyl-4-oxo-1,2,3,4-tetrahydroquinazolin-2-yl)piperidine-1-carboxylate (2f)**

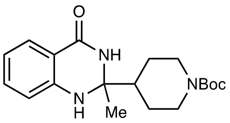

Following GP2, using 2-aminobenzamide (1.36 g, 10.0 mmol), *tert*-butyl 4-acetylpiperidine-1-carboxylate (2.38 g, 10.5 mmol) and iodine (0.13 g, 0.5 mmol) in DMF (15 mL) at 80 °C for 24 hours. The product was obtained as a white solid (1.95 g, 56% yield). **M.p.**: 206 °C. **IR (neat, cm^{-1}):** 3297, 3175, 2951, 1688, 1632, 1425, 1239, 1161, 1209, 755. **^{1}H NMR (400 MHz, DMSO-*d*$_6$)** δ 7.92 (s, 1H), 7.52 (dd, *J* = 7.7, 1.5 Hz, 1H), 7.17 (ddd, *J* = 8.1, 7.2, 1.6 Hz, 1H), 6.64 (d, *J* = 7.7 Hz, 1H), 6.61 (s, 1H), 6.58 – 6.52 (m, 1H), 4.13 – 3.84 (m, 2H), 2.65 – 2.39 (m, 2H), 1.75 – 1.57 (m, 3H), 1.36 (s, 9H), 1.30 (s, 3H), 1.22 – 1.08 (m, 2H) ppm. **^{13}C NMR (101 MHz, DMSO-*d*$_6$)** δ 162.8, 153.7, 146.9, 133.3, 127.0, 115.9, 113.6, 113.3, 78.5, 70.8, 46.5, 28.1, 26.0, 25.8, 25.2 ppm. **HRMS** [ESI$^+$] *calcd.* for ($C_{19}H_{27}N_3NaO_3$) [M+Na]$^+$: 368.1950, *found*: 368.1944.

2-Benzoyl-2-methyl-2,3-dihydroquinazolin-4(1*H*)-one (2g)

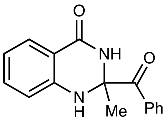

Following GP2, using 2-aminobenzamide (1.36 g, 10.0 mmol), 1-phenylpropane-1,2-dione (1.55 g, 10.5 mmol) and iodine (0.13 g, 0.5 mmol) in DMF (15 mL) at 80 °C for 24 hours. The product was obtained as a white solid (0.90 g, 34% yield). **M.p.**: 168 °C. **^{1}H NMR (400 MHz, DMSO-*d*$_6$)** δ 8.63 (s, 1H), 7.95 – 7.89 (m, 2H), 7.61 – 7.51 (m, 3H), 7.50 – 7.43 (m, 2H), 7.24 (ddd, *J* = 8.2, 7.2, 1.6 Hz, 1H), 6.77 – 6.72 (m, 1H), 6.69 – 6.63 (m, 1H), 1.67 (s, 3H) ppm. **^{13}C NMR (101 MHz, DMSO-*d*$_6$)** δ 200.7, 163.2, 146.6, 134.8, 133.5, 132.4, 128.9, 128.4, 127.3, 117.6, 114.5, 114.4, 73.8, 24.5 ppm. Spectral data was in agreement with the literature.[2]

2-(Dimethoxymethyl)-2-methyl-2,3-dihydroquinazolin-4(1*H*)-one (2h)

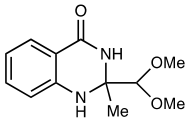

Following GP2, using 2-aminobenzamide (2.72 g, 20.0 mmol), 1,1-dimethoxypropan-2-one (2.48 g, 21.0 mmol) and iodine (0.25 g, 1.0 mmol) in DMF (30 mL) at 80 °C for 24 hours. The product was obtained as a pale-yellow solid (1.56 g, 33% yield). **M.p.**: 169 °C. **IR (neat, cm^{-1}):** 3300, 2935, 1649, 1517, 1483, 1331, 1272, 1070, 982, 740. **^{1}H NMR (400 MHz, DMSO-*d*$_6$)** δ 7.87 (s, 1H), 7.55 (dd, *J* = 7.7, 1.7 Hz, 1H), 7.19 (ddd, *J* = 8.2, 7.1, 1.6 Hz, 1H), 6.75 – 6.68 (m, 2H), 6.62 – 6.55 (m, 1H), 4.13 (s, 1H), 3.35 (s, 3H), 3.35 (s, 3H), 1.28 (s, 3H) ppm. **^{13}C NMR (101 MHz, DMSO-*d*$_6$)** δ 162.9, 146.9, 133.2, 126.9, 116.2, 113.8, 113.5, 107.9, 70.3, 57.6, 57.5, 21.4 ppm. **HRMS** [ESI$^+$] *calcd.* for ($C_{12}H_{16}N_2NaO_3$) [M+Na]$^+$: 259.1059, *found*: 259.1055.

2-((2-Methyl-4-oxo-1,2,3,4-tetrahydroquinazolin-2-yl)methyl)isoindoline-1,3-dione (2i)

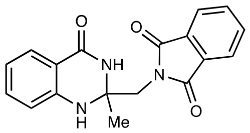

Following GP2, using 2-aminobenzamide (1.36 g, 10.0 mmol), 2-(2-oxopropyl)isoindoline-1,3-dione (2.13 g, 10.5 mmol) and iodine (0.13 g, 0.5 mmol) in DMF (15 mL) at 80 °C for 24 hours. The product was obtained as a pale-yellow solid (2.34 g, 73% yield). **M.p.**: 215 °C. **IR (neat, cm^{-1}):** 3371, 3335, 1699, 1661, 1613, 1516, 1425, 1391, 1150, 1076, 761, 532. **^{1}H NMR (400 MHz, DMSO-*d*$_6$)** δ 8.02 (s, 1H), 7.87 – 7.79 (m, 4H), 7.54 (dd, *J* = 7.7, 1.5 Hz, 1H), 7.21 (ddd, *J* = 8.2, 7.2, 1.6 Hz, 1H), 6.81 (s, 1H), 6.64 – 6.54 (m, 2H), 3.76 – 3.66 (m, 2H), 1.39 (s, 3H) ppm. **^{13}C NMR (101 MHz, DMSO-*d*$_6$)** δ 167.9, 162.6, 146.6, 134.2, 133.1, 131.8, 127.0, 123.0, 116.6, 114.5, 113.8, 69.2, 45.4, 26.3 ppm. **HRMS** [ESI$^+$] *calcd.* for ($C_{18}H_{15}N_3NaO_3$) [M+Na]$^+$: 344.1011, *found*: 344.1009.

(2-Methyl-4-oxo-1,2,3,4-tetrahydroquinazolin-2-yl)methyl acetate (2j)

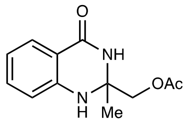

Following GP3, using 2-aminobenzamide (1.36 g, 10.0 mmol), 2-oxopropyl acetate (1.22 g, 10.5 mmol) and iodine (0.13 g, 0.5 mmol) in DMF (15 mL) at 80 °C for 24 hours. The product was obtained as a white solid (1.57 g, 67% yield), by using hexane/EtOAc (1:1) as chromatography eluent. **M.p.**: 138 °C. **^{1}H NMR (400 MHz, DMSO-*d*$_6$)** δ 7.96 (s, 1H), 7.49 (dd, *J* = 7.9, 1.5 Hz, 1H), 7.18 – 7.09 (m, 1H), 6.72 (s, 1H), 6.60 – 6.50 (m, 2H), 3.96 (d, *J* = 10.9 Hz, 1H), 3.87 (d, *J* = 10.9 Hz, 1H), 1.80 (s, 3H), 1.30 (s, 3H) ppm. **^{13}C NMR (101 MHz, DMSO-*d*$_6$)** δ 170.1, 163.1, 146.8, 133.4, 127.1, 116.7, 114.0, 113.7, 68.0, 67.9, 24.8, 20.5 ppm. Spectroscopic data was in agreement with the literature.[3]

2-(Methoxymethyl)-2-methyl-2,3-dihydroquinazolin-4(1*H*)-one (2k)

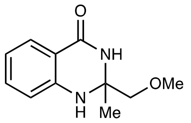

Following GP2, using 2-aminobenzamide (1.36 g, 10.0 mmol), 1-methoxypropan-2-one (0.92 g, 10.5 mmol) and iodine (0.13 g, 0.5 mmol) in DMF (15 mL) at 80 °C for 24 hours. The product was obtained as a white solid (0.61 g, 30% yield). **M.p.**: 210 °C. **^{1}H NMR (400 MHz, DMSO-*d*$_6$)** δ 7.91 (s, 1H), 7.54 (dd, *J* = 7.7, 1.6 Hz, 1H), 7.20 (ddd, *J* = 8.2, 7.2, 1.6 Hz, 1H), 6.71 (s, 1H), 6.69 – 6.64 (m, 1H), 6.63 – 6.57 (m, 1H), 3.36 (d, *J* = 9.4 Hz, 1H), 3.24 (s, 3H), 3.21 (d, *J* = 9.4 Hz, 1H), 1.35 (s, 3H) ppm. **^{13}C NMR (101 MHz, DMSO-*d*$_6$)** δ 163.0, 146.9, 133.3, 127.1, 116.4, 114.0, 113.7, 77.4, 68.3, 58.9, 24.8 ppm. Spectroscopic data was in agreement with the literature.[1]

2-Methyl-2-(phenoxymethyl)-2,3-dihydroquinazolin-4(1*H*)-one (2l)

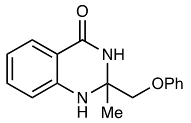

Following GP2, using 2-aminobenzamide (1.36 g, 10.0 mmol), 1-phenoxypropan-2-one (1.58 g, 10.5 mmol) and iodine (0.13 g, 0.5 mmol) in DMF (15 mL) at 80 °C for 24 hours. The product was obtained as a white solid (1.59 g, 59% yield). **M.p.**: 163 °C. **^{1}H NMR (400 MHz, DMSO-*d*$_6$)** δ 8.08 (s, 1H), 7.57 (dd, *J* = 7.7, 1.8 Hz, 1H), 7.26 – 7.16 (m, 3H), 6.93 – 6.86 (m, 2H), 6.86 – 6.80 (m, 2H), 6.70 – 6.58 (m, 2H), 3.94 (d, *J* = 9.4 Hz, 1H), 3.81 (d, *J* = 9.4 Hz, 1H), 1.48 (s, 3H) ppm. **^{13}C NMR (101 MHz, DMSO-*d*$_6$)** δ 163.1, 158.4, 146.8, 133.3, 129.5, 127.0, 120.9, 116.5, 114.8, 113.9, 113.8, 73.0, 68.2, 24.9 ppm. Spectroscopic data was in agreement with the literature.[7]

2-Ethyl-2-(phenoxymethyl)-2,3-dihydroquinazolin-4(1*H*)-one (2l´)

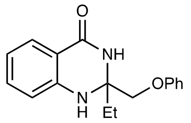

Following GP2, using 2-aminobenzamide (1.36 g, 10.0 mmol), 1-phenoxybutan-2-one (1.72 g, 10.5 mmol) and iodine (0.13 g, 0.5 mmol) in DMF (15 mL) at 80 °C for 24 hours. The product was obtained as a pale-yellow solid (1.68 g, 60% yield). **M.p.**: 167 °C. **IR (neat, cm^{-1}):** 3381, 2979, 2935, 1660, 1597, 1484, 1237, 746. **^{1}H NMR (500 MHz, DMSO-*d*$_6$)** δ 7.95 (s, 1H), 7.57 (dd, *J* = 7.7, 1.4 Hz, 1H), 7.23 (t, *J* = 7.9 Hz, 2H), 7.21 – 7.16 (m, 1H), 6.90 (t, *J* = 7.3 Hz, 1H), 6.84 (d, *J* = 7.9, 2H), 6.74 (s, 1H), 6.68 (d, *J* = 7.9 Hz, 1H), 6.59 (t, *J* = 7.4 Hz, 1H), 3.93 (d, *J* = 9.4 Hz, CH_aCH_b, 1H), 3.81 (d, *J* = 9.4 Hz, CH_aCH_b, 1H), 1.84 – 1.71 (m, 2H), 0.98 (t, *J* = 7.2 Hz, 3H). **^{13}C NMR (126 MHz, DMSO-*d*$_6$)** δ 163.5, 158.5, 147.3, 133.2, 129.5, 127.0, 120.9, 116.1, 114.8, 113.8, 113.3, 73.1, 71.0, 29.1, 7.5. **HRMS** [ESI$^+$] *calcd.* for ($C_{17}H_{18}N_2NaO_2$) [M+Na]$^+$: 305.1260, *found*: 305.1259.

2-Butyl-2-methyl-2,3-dihydroquinazolin-4(1*H*)-one (2m)

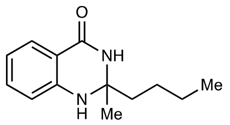

Following GP2, using 2-aminobenzamide (1.36 g, 10.0 mmol), hexan-2-one (1.20 g, 10.5 mmol) and iodine (0.13 g, 0.5 mmol) in DMF (15 mL) at 80 °C for 24 hours. The product was obtained as a white solid (816 mg, 37% yield). **M.p.**: 181 °C. **IR (neat, cm^{-1}):** 3326, 3166, 2952, 1636, 1611, 754. **^{1}H NMR (500 MHz, DMSO-*d_6*)** δ 7.85 (s, 1H), 7.54 (dd, *J* = 7.7, 1.6 Hz, 1H), 7.19 (ddd, *J* = 8.6, 7.2, 1.7 Hz, 1H), 6.63 (d, *J* = 8.1 Hz, 1H), 6.60 – 6.55 (m, 2H), 1.67 – 1.56 (m, 2H), 1.38 – 1.28 (m, 5H), 1.23 (h, *J* = 7.2, 2H), 0.84 (t, *J* = 7.2, 1.0 Hz, 3H). **^{13}C NMR (126 MHz, DMSO-*d_6*)** δ 163.1, 147.2, 133.2, 127.1, 116.0, 114.0, 113.5, 69.1, 41.1, 27.9, 25.6, 22.5, 14.0. **HRMS** [ESI^+] *calcd.* for ($C_{13}H_{18}N_2NaO$) $[M+Na]^+$: 241.1311, *found*: 241.1310.

2-Butyl-2-phenyl-2,3-dihydroquinazolin-4(1*H*)-one (2m´)

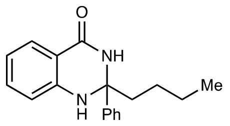

Following GP3, using 2-aminobenzamide (1.36 g, 10.0 mmol), 1-phenylpentan-1-one (1.70 g, 10.5 mmol) and iodine (0.13 g, 0.5 mmol) in DMF (15 mL) at 80 °C for 24 hours. The product was obtained as a white solid (1.67 g, 60% yield), by using hexane/EtOAc (1:1) as eluent. **M.p.**: 143 °C. **IR (neat, cm^{-1}):** 3270, 2945, 1610, 1482, 1378, 1149, 746, 697. **^{1}H NMR (400 MHz, $CDCl_3$)** δ 7.74 (dd, *J* = 7.8, 1.3 Hz, 1H), 7.44 (s, 1H), 7.40 – 7.33 (m, 2H), 7.24 – 7.10 (m, 4H), 6.71 – 6.61 (m, 2H), 1.97 (t, *J* = 8.2 Hz, 2H), 1.39 – 1.16 (m, 4H), 0.77 (t, *J* = 7.1 Hz, 2H) ppm. **^{13}C NMR (101 MHz, $CDCl_3$)** δ 165.3, 146.1, 145.1, 134.1, 128.6, 128.5, 127.9, 125.5, 119.0, 115.8, 115.0, 73.6, 42.7, 26.0, 22.7, 14.0 ppm. **HRMS** [ESI^+] *calcd.* for ($C_{18}H_{20}N_2NaO$) $[M+Na]^+$: 303.1473, *found*: 303.1474.

2-Butyl-2-(4-methoxyphenyl)-2,3-dihydroquinazolin-4(1*H*)-one (2m´´)

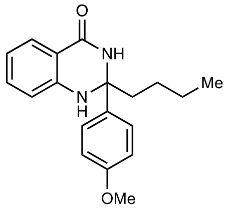

Following GP3, using 2-aminobenzamide (1.36 g, 10.0 mmol), 1-(4-methoxyphenyl)pentan-1-one (2.02 g, 10.5 mmol) and iodine (0.13 g, 0.5 mmol) in DMF (15 mL) at 80 °C for 24 hours. The product was obtained as a white solid (1.90 g, 61% yield), by using hexane/EtOAc (3:1) as eluent. **M.p.**: 163 °C. **IR (neat, cm^{-1}):** 3255, 3178, 2957, 1644, 1611, 1255, 752. **^{1}H NMR (500 MHz, DMSO-*d*$_6$)** δ 8.57 (s, 1H), 7.46 (dd, *J* = 7.7, 1.5 Hz, 1H), 7.39 (s, 1H), 7.36 (d, *J* = 8.9 Hz, 2H), 7.18 (ddd, *J* = 8.2, 7.2, 1.6 Hz, 1H), 6.82 (d, *J* = 8.9 Hz, 2H), 6.80 (d, *J* = 8.1 Hz, 1H), 6.54 (d, *J* = 7.6 Hz, 1H), 3.67 (s, 3H), 1.83 – 1.71 (m, 2H), 1.43 (quint., *J* = 7.9 Hz, 2H), 1.32 – 1.22 (m, 2H), 0.87 (t, *J* = 7.3 Hz, 3H). **^{13}C NMR (126 MHz, DMSO-*d*$_6$)** δ 164.1, 158.1, 147.5, 139.7, 133.1, 127.1, 126.6, 116.5, 114.8, 114.4, 113.2, 72.6, 55.0, 42.3, 26.0, 22.2, 14.0. **HRMS** [ESI$^+$] *calcd.* for ($C_{19}H_{23}N_2O_2$) $[M+H]^+$: 311.1754, *found*: 311.1750.

2-(Benzo[d][1,3]dioxol-5-yl)-2-butyl-2,3-dihydroquinazolin-4(1*H*)-one (2m´´´)

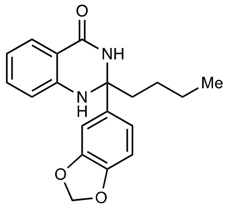

Following GP3, using 2-aminobenzamide (1.36 g, 10.0 mmol), 1-(benzo[*d*][1,3]dioxol-5-yl)pentan-1-one (2.16 g, 10.5 mmol) and iodine (0.13 g, 0.5 mmol) in DMF (15 mL) at 80 °C for 24 hours. The product was obtained as a white solid (1.84 g, 57% yield), by using hexane/EtOAc (3:1) as eluent. **M.p.**: 85 °C. **IR (neat, cm^{-1}):** 3257, 2957, 1646, 1611, 1484, 1242. **^{1}H NMR (500 MHz, DMSO-*d*$_6$)** δ 8.57 (s, 1H), 7.46 (d, *J* = 7.7 Hz, 1H), 7.38 (s, 1H), 7.22 – 7.17 (m, 1H), 7.03 (d, *J* = 1.7 Hz, 1H), 6.89 (dd, *J* = 8.1, 1.7 Hz, 1H), 6.79 (d, *J* = 8.2 Hz, 1H), 6.78 (d, *J* = 8.1 Hz, 1H), 6.56 (t, *J* = 7.6 Hz, 1H), 5.94 (s, CH_aCH_b, 1H), 5.93 (s, CH_aCH_b, 1H), 1.79 – 1.70 (m, 2H), 1.42 (quint., *J* = 7.8 Hz, 2H), 1.27 (h, *J* = 7.3 Hz, 2H), 0.87 (t, *J* = 7.3 Hz, 3H). **^{13}C NMR (126 MHz, DMSO-*d*$_6$)** δ 164.5, 147.9, 147.6, 146.6, 142.5, 133.7, 127.6, 119.1, 117.1, 115.3, 114.9, 107.8, 106.6,

101.4, 73.3, 42.7, 26.4, 22.7, 14.5. **HRMS** [ESI$^+$] *calcd.* for ($C_{19}H_{20}N_2NaO_3$) [M+Na]$^+$: 347.1366, *found*: 347.1360.

Isopropyl 4-(4-oxo-2-phenyl-1,2,3,4-tetrahydroquinazolin-2-yl)butanoate (2n)

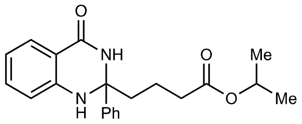

Following GP3, using 2-aminobenzamide (1.36 g, 10.0 mmol), isopropyl 5-oxo-5-phenylpentanoate (2.46 g, 10.5 mmol) and iodine (0.13 g, 0.5 mmol) in DMF (15 mL) at 80 °C for 24 hours. The product was obtained as a white solid (2.71 g, 49% yield), by using hexane/EtOAc (2:1) as chromatography eluent. **M.p.**: 165 °C. **IR (neat, cm^{-1}):** 3326, 2977, 1712, 1643, 1485, 1371, 1285, 1201, 1109, 757, 698. **^{1}H NMR (400 MHz, DMSO-*d*$_6$)** δ 8.67 (s, 1H), 7.49 (s, 1H), 7.47 – 7.42 (m, 3H), 7.27 (t, *J* = 7.6 Hz, 2H), 7.22 – 7.13 (m, 2H), 6.82 (d, *J* = 7.7 Hz, 1H), 6.58 – 6.52 (m, 1H), 4.86 (hept., *J* = 6.3 Hz, 1H), 2.28 – 2.20 (m, 2H), 1.83 – 1.68 (m, 4H), 1.15 (d, *J* = 6.2, Hz, 6H) ppm. **^{13}C NMR (101 MHz, DMSO-*d*$_6$)** δ 172.2, 164.0, 147.5, 147.4, 133.3, 128.0, 127.2, 127.1, 125.3, 116.7, 114.7, 114.4, 72.8, 67.0, 41.4, 33.8, 21.6, 19.8 ppm. **HRMS** [ESI$^+$] *calcd.* for ($C_{21}H_{24}N_2NaO_3$) [M+Na]$^+$: 375.1685, *found*: 375.1677.

2-(2-(Benzo[*d*][1,3]dioxol-5-yl)ethyl)-2-phenyl-2,3-dihydroquinazolin-4(1*H*)-one (2o)

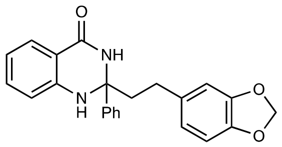

Following GP3, using 2-aminobenzamide (1.36 g, 10.0 mmol), 3-(benzo[*d*][1,3]dioxol-5-yl)-1-phenylpropan-1-one (2.67 g, 10.5 mmol) and iodine (0.13 g, 0.5 mmol) in DMF (15 mL) at 80 °C for 24 hours. The product was obtained as a white solid (2.83 g, 76% yield), by using hexane/EtOAc (2:1) as chromatography eluent. **M.p.**: 95 °C. **IR (neat, cm^{-1}):** 3293, 1647, 1609, 1482, 1443, 1244, 1035, 925, 752, 697. **^{1}H NMR (400 MHz, $CDCl_3$)** δ 7.99 (s, 1H), 7.77 (dd, *J* = 7.8, 1.3 Hz, 1H), 7.44 – 7.36 (m, 2H), 7.27 – 7.10 (m, 4H), 6.73 – 6.67 (m, 1H), 6.63 – 6.58 (m, 3H), 6.54 – 6.50 (m, 1H), 5.83 – 5.81 (m, 2H), 2.74 – 2.55 (m, 2H), 2.33 – 2.16 (m, 2H) ppm. **^{13}C NMR (101 MHz, $CDCl_3$)** δ 165.5, 147.9, 146.1, 146.0, 145.0, 134.9, 134.2, 128.8, 128.6, 128.1, 125.4, 121.3, 119.2, 115.7, 115.0, 109.1, 108.4, 101.0, 73.6, 44.8, 30.4 ppm. **HRMS** [ESI$^+$] *calcd.* for ($C_{23}H_{20}N_2NaO_3$) [M+Na]$^+$: 395.1372, *found*: 395.1371.

2-Methyl-2-(4-morpholino-4-oxobutyl)-2,3-dihydroquinazolin-4(1*H*)-one (2p)

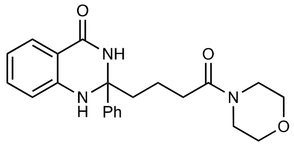

Following GP2, using 2-aminobenzamide (1.36 g, 10.0 mmol), 1-morpholino-5-phenylpentane-1,5-dione (2.74 g, 10.5 mmol) and iodine (0.13 g, 0.5 mmol) in DMF (15 mL) at 80 °C for 24 hours. The product was obtained as a white solid (2.21 g, 70% yield). **M.p.**: 254 °C. **IR (neat, cm^{-1}):** 3322, 3232, 1627, 1510, 1363, 1276, 1226, 1112, 1027, 757, 705. **^{1}H NMR (400 MHz, DMSO-*d*$_6$)** δ 8.67 (s, 1H), 7.49 (s, 1H), 7.47 – 7.42 (m, 3H), 7.31 – 7.24 (m, 2H), 7.22 – 7.13 (m, 2H), 6.82 (d, *J* = 7.7 Hz, 1H), 6.58 – 6.52 (m, 1H), 3.56 – 3.48 (m, 4H), 3.44 – 3.36 (m, 4H), 2.32 – 2.24 (m, 2H), 1.87 – 1.76 (m, 2H), 1.74 – 1.62 (m, 2H) ppm. **^{13}C NMR (101 MHz, DMSO-*d*$_6$)** δ 170.7, 164.0, 147.6, 147.5, 133.3, 127.9, 127.2, 127.0, 125.4, 116.6, 114.7, 114.5, 72.9, 66.1, 45.5, 41.8, 41.4, 32.2, 19.8 ppm. **HRMS** [ESI$^+$] *calcd.* for ($C_{22}H_{25}N_3NaO_3$) [M+Na]$^+$: 402.1794, *found*: 402.1784.

Synthesis of (3a*S*,4*S*,6a*R*)-4-(5-oxo-5-phenylpentyl)tetrahydro-1*H*-thieno[3,4-*d*]imidazol-2(3*H*)-one (S1)

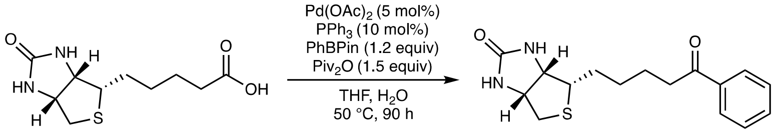

A 27 mL glass screw-cap vial was charged with a stirrer bar, palladium acetate (12.5 mg, 0.06 mmol, trimer), triphenylphosphine (31.7 mg, 0.12 mmol), D-(+)-biotin (266 mg, 1.09 mmol) and phenylboronic acid (161 mg, 1.32 mmol). The reaction vessel was equipped with a rubber septum with an argon inlet. The contents of the reaction vessel were flushed with argon, then charged with THF (5 mL), pivalic anhydride (0.32 mL, 1.64 mmol) and water (47 μL). The rubber septum and argon inlet were quickly removed and replaced with a screw cap. The reaction mixture was stirred for 90 hours at 50 °C, after which the reaction mixture was concentrated under reduced pressure yielding crude material, which was purified by silica gel chromatography (0 to 3% MeOH in DCM), yielding the title compound (68.4 mg, 21%) as a white solid. **M.p.** 171 °C. **IR (film, cm^{-1}) ν_{max}** = 3210, 2922, 1698. **^{1}H NMR (500 MHz, CDCl3)** δ 7.98 – 7.93 (m, 2H), 7.55 (tt, *J* = 7.4, 1.2 Hz, 1H), 7.46 (tt, *J* = 7.4 Hz, 2H), 4.52 (dd, *J* = 7.8, 5.0 Hz, 1H), 4.33 (dd, *J* = 7.8, 4.6 Hz, 1H), 3.19 (ddd, *J* = 8.5, 6.5, 4.6 Hz, 1H), 3.00 (t, *J* = 7.3 Hz, 2H), 2.92 (dd, *J* = 12.9, 5.0 Hz, 1H), 2.74 (d, *J* = 12.9 Hz, 1H), 1.85 – 1.67 (m, 4H), 1.57 – 1.46 (m, 2H) ppm. **^{13}C NMR (126 MHz, $CDCl_3$)** δ. 200.5, 163.5, 137.0, 133.2, 128.8, 128.2, 62.2, 60.4, 55.5, 40.7, 38.3, 28.7, 28.6, 24.2 ppm. **HRMS** [ESI$^+$] *calcd.* for ($C_{16}H_{20}N_2NaO_2S$) [M+Na]$^+$: 327.1138, *found* 327.1130.

Synthesis of (3a*S*,4*S*,6a*R*)-4-(4-(4-Oxo-2-phenyl-1,2,3,4-tetrahydroquinazolin-2-yl)butyl)tetrahydro-1*H*-thieno[3,4-*d*]imidazol-2(3*H*)-one (2q)

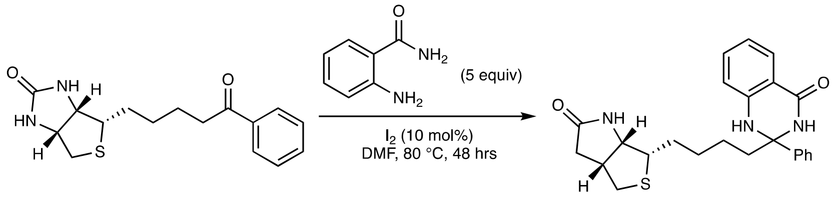

A 25 mL flask containing a stirring bar was charged with 2-aminobenzamide (558 mg, 4.1 mmol), (3a*S*,4*S*,6a*R*)-4-(5-oxo-5-phenylpentyl)tetrahydro-1*H*-thieno[3,4-*d*]imidazol-2(3*H*)-one (251 mg, 0.82 mmol), iodine (20.3 mg, 0.08 mmol) and DMF (10 mL). The reaction mixture was stirred at 70 °C for 68 hours. The reaction was cooled to 20 °C, quenched with 10% $Na_2S_2O_3$ (aq, 20 mL) and extracted using EtOAc (3 x 30 mL). The combined organic extracts were dried (Na_2SO_4) and concentrated under reduced pressure affording crude material, which was purified by silica gel chromatography (0 to 6% MeOH in DCM) yielding the title compound (44.3 mg, 10%) as a white solid. **M.p.** 170 °C. **IR (film, cm^{-1}) ν_{max}** = 3245, 2923, 1687, 1687, 1611, 1483. **^{1}H NMR (500 MHz, DMSO-*d6*)** δ 8.65 (s, 1H), 7.50 – 7.43 (m, 4H), 7.27 (t, *J* = 7.6 Hz, 2H), 7.21 – 7.15 (m, 2H), 6.81 (d, *J* = 8.2 Hz, 1H), 6.55 (t, *J* = 7.4 Hz, 1H), 6.40 (br. s, 1H), 6.36 (br. s, 1H), 4.32 – 4.28 (m, 1H), 4.14 – 4.10 (m, 1H), 3.11 (tt, *J* = 10.0, 5.5 Hz, 1H), 2.81 (ddd, *J* = 12.5, 5.1, 2.7 Hz, 1H), 2.57 (d, *J* = 12.4 Hz, 1H), 1.85 – 1.70 (m, 2H), 1.66 – 1.57 (dt, *J* = 17.7, 6.0 Hz, 1H), 1.55 – 1.43 (m, 3H), 1.39 – 1.26 (m, 2H) ppm. **^{13}C NMR (126 MHz, DMSO-*d6*)** δ 164.6, 163.2, 148.3, 148.0, 133.7, 128.4, 127.6, 127.4, 125.8, 117.1, 115.2, 114.9, 73.4, 61.6, 59.7, 55.8, 42.6, 40.2, 29.0, 28.7, 24.2 ppm. **HRMS** [ESI$^+$] *calcd.* for ($C_{23}H_{26}N_4NaO_2S$) $[M+Na]^+$: 445.1669, *found* 445.1662.

Synthesis of methyl 1-cyclopropyl-6-fluoro-4-oxo-7-(4-(4-(4-oxo-2-phenyl-1,2,3,4-tetrahydroquinazolin-2-yl)butanoyl)piperazin-1-yl)-1,4-dihydroquinoline-3-carboxylate (2r)

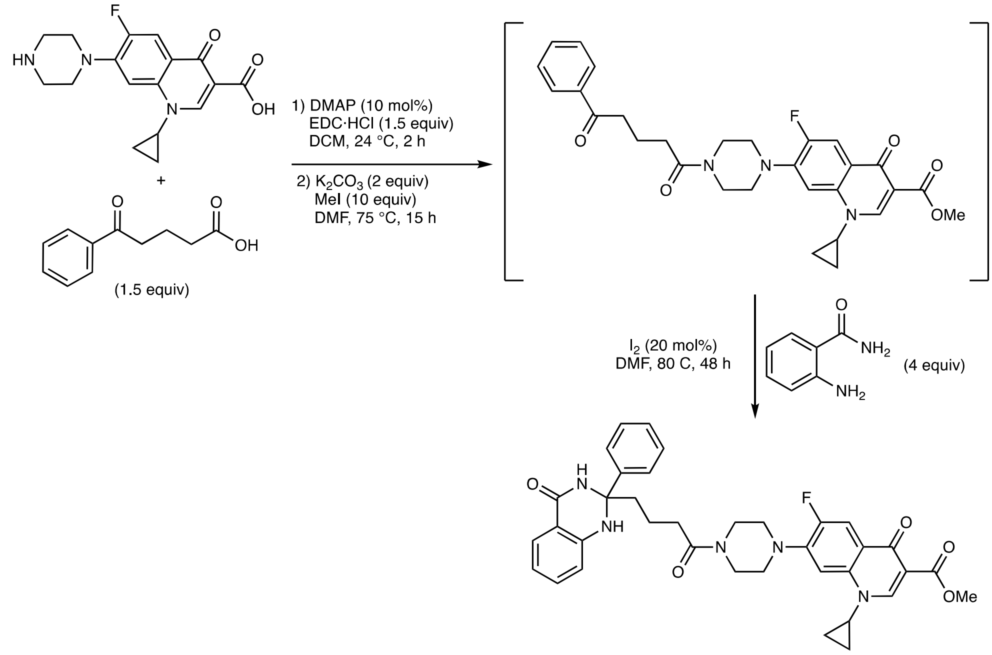

A 27 mL screw cap vial equipped with a stirrer bar was charged with ciprofloxacin (900 mg, 2.72 mmol) and DCM (15 mL). The mixture was cooled to 0 °C, then 5-oxo-5-phenylpentanoic acid (782 mg, 4.07 mmol), *N*-(3-Dimethylaminopropyl)-*N'*-ethylcarbodiimide hydrochloride (780 mg, 4.07 mmol) and DMAP (33 mg, 0.27 mmol) were sequentially added forming a white suspension in solution. The reaction mixture was warmed to 24 °C and stirred for 2 hours, during which the white suspension dissolved forming a yellow solution. The reaction was quenched with 1 M HCl (aq, 40 mL) and extracted with DCM (2 x 75 mL). The combined organic extracts were dried ($MgSO_4$) and concentrated under reduced pressure affording crude material (1.50 g) as a white solid, which was used without further purification. A 27 mL glass vial equipped with a stirrer bar was charged with the crude material (1.50 g) of the previous step and potassium carbonate (752 mg, 5.44 mmol). The reaction vessel was equipped with a rubber septum and an argon inlet. Contents of the reaction vessel were flushed with argon, then charged with DMF (20 mL, anhydrous) and stirred for 30 minutes at 60 °C. The reaction mixture was cooled to 24 °C and charged with iodomethane (0.68 mL, 10.9 mmol). The reaction vessel was sealed with a screw cap and the contents were stirred at 70 °C for 16 hours. The reaction mixture was cooled to 24 °C then quenched with saturated Na_2CO_3 (aq, 150 mL) and extracted with DCM (3 x 100 mL). The combined organic extracts were dried ($MgSO_4$) and concentrated under reduced pressure yielding crude material as a brown

residue, which was filtered through a plug of silica (loaded with DCM, released with 3% MeOH in DCM) affording crude material (1.15 g) as a white solid that was used without further purification. A 27 mL glass vial equipped with a stirring bar was charged with the crude material (1.15 mg) of the previous step, 2-aminobenzamide (1.37 g, 10.1 mmol), iodine (66 mg, 0.26 mmol) and DMF (10 mL). The reaction mixture was stirred for 5 hours at 80 °C, then an extra aliquot of iodine (33 mg, 0.13 mmol) was added and the reaction was stirred for an additional 3 hours at 80 °C. The reaction mixture was cooled to 20 °C, quenched with water (60 mL) and filtered affording a brown residue as crude material. A 500 mL RBF was charged with the crude material and while heating at 90 °C ethanol (125 mL) was added forming a white suspension. While still hot the white suspension was filtered yielding a white solid, which was sequentially washed with methanol (30 mL) and diethyl ether (30 mL), yielding the title compound (963 mg, 56%) as a white solid. **M.p.** 292 °C. **IR (film, cm^{-1}) ν_{max} =** 3300, 1686, 1660, 1618, 1446, 1242. **^{1}H NMR (500 MHz, DMSO-*d*$_6$)** δ 8.71 (s, 1H), 8.43 (s, 1H), 7.76 (d, *J* = 13.1 Hz, 1H), 7.56 –7.40 (m, 5 H), 7.29 (t, *J* = 7.7 Hz, 2H), 7.24 – 7.14 (m, 2H), 6.84 (d, *J* = 8.1 Hz, 1H), 6.56 (d, *J* = 8.1 Hz, 1H), 3.73 (s, 3H), 3.70 – 3.59 (m, 5H), 3.23 (br.s, 2H), 3.19 (br.s, 2H), 2.41 – 2.32 (m, 2H), 1.91 – 1.80 (m, 2H), 1.80 – 1.67 (m, 2H), 1.29 – 1.23 (m, 2H), 1.14 – 1.04 (m, 2H) ppm. **^{13}C NMR (126 MHz, DMSO-*d*$_6$)** δ 171.5, 170.6, 164.9, 164.0, 152.5 (d, *J* = 246.5 Hz), 148.30, 147.6, 147.5, 143.6 (d, *J* = 10.2 Hz), 138.0, 133.3, 127.9, 127.1, 127.0, 125.4, 122.1 (d, *J* = 6.2 Hz), 116.6, 114.7, 114.4, 111.6 (d, *J* = 22.6 Hz), 109.0, 106.6, 72.9, 51.3, 49.9, 49.4, 44.8, 41.8, 40.7, 34.8, 32.4, 19.9, 7.6 ppm. **^{19}F NMR (376 MHz, DMSO-*d*$_6$)** δ -124.7 (dd, *J* = 13.3, 7.5 Hz). **HRMS** [ESI$^+$] *calcd.* for ($C_{36}H_{37}FN_5O_5$) $[M+H]^+$: 638.2773, *found* 638.2767.

2-((2,5-Dimethylphenoxy)methyl)-2-methyl-2,3-dihydroquinazolin-4(1*H*)-one (2s)

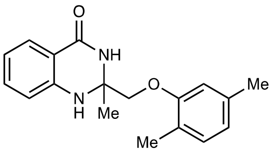

Following GP3, using 2-aminobenzamide (3.81 g, 28.0 mmol), 1-phenylpentan-1-one (2.52 g, 14.1 mmol) and iodine (0.36 g, 1.4 mmol) in DMF (20 mL) at 80 °C for 48 hours. The product was obtained as a pale-yellow solid (2.69 g, 64% yield), by using 0% to 20% acetone in hexane as eluent. **M.p.**: 159 °C. **IR (neat, cm^{-1}):** 3249, 3172, 3016, 1639, 1614, 1261, 752. **^{1}H NMR (500 MHz, DMSO-*d*$_6$)** δ 8.05 (s, 1H), 7.58 (dd, *J* = 7.7, 1.4 Hz, 1H), 7.19 (ddd, *J* = 8.6, 7.2, 1.6 Hz, 1H), 6.91 (d, *J* = 7.5 Hz, 1H), 6.81 (s, 1H), 6.66 – 6.63 (m, 2H), 6.62 – 6.58 (m, 2H), 3.90 (d, *J* = 9.3 Hz, CH_aCH_b, 1H), 3.80 (d, *J* = 9.3 Hz, CH_aCH_b, 1H), 2.19 (s, 3H), 1.85 (s, 3H), 1.52 (s, 3H). **^{13}C NMR (126 MHz, DMSO-*d*$_6$)** δ 163.3, 156.2, 147.1, 136.1, 133.2, 130.0, 127.1, 122.8, 120.9, 116.2, 113.8, 113.6, 112.0, 73.9, 68.3, 25.2, 20.9, 15.2. **HRMS** [ESI$^+$] *calcd.* for ($C_{18}H_{20}N_2NaO_2$) [M+Na]$^+$: 319.1417, *found*: 319.1418.

2-(Cyclopropylmethyl)-2-phenyl-2,3-dihydroquinazolin-4(1*H*)-one (2t)

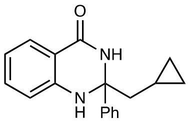

Following GP3, using 2-aminobenzamide (787 mg, 5.8 mmol), 2-cyclopropyl-1-phenylethan-1-one (926 mg, 5.8 mmol) and iodine (74 mg, 0.3 mmol) in DMF (8.5 mL) at 80 °C for 24 hours. The product was obtained as a white solid (1.24 g, 77% yield), by using hexane/EtOAc (3:1) as chromatography eluent. **M.p.**: 154 °C. **IR (neat, cm^{-1}):** 3438, 3011, 1657, 1611, 1488, 1373, 1268, 1155, 1028, 762, 699. **^{1}H NMR (400 MHz, DMSO-*d*$_6$)** δ 8.61 (s, 1H), 7.53 – 7.42 (m, 4H), 7.30 – 7.22 (m, 2H), 7.21 – 7.11 (m, 2H), 6.86 – 6.79 (m, 1H), 6.58 – 6.50 (m, 1H), 1.81 – 1.65 (m, 2H), 1.02 – 0.90 (m, 1H), 0.43 – 0.28 (m, 2H), 0.24 – 0.15 (m, 1H), 0.07 – -0.03 (m, 1H) ppm. **^{13}C NMR (101 MHz, DMSO-*d*$_6$)** δ 164.1, 147.6, 147.5, 133.2, 127.8, 127.2, 127.0, 125.5, 116.6, 114.7, 114.4, 73.5, 46.9, 6.2, 4.3, 4.1 ppm. **HRMS** [ESI$^+$] *calcd.* for ($C_{18}H_{18}N_2NaO$) [M+Na]$^+$: 301.1317, *found*: 301.1319.

2-(Hex-5-en-1-yl)-2-phenyl-2,3-dihydroquinazolin-4(1*H*)-one (2u)

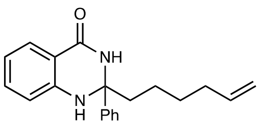

Following GP3, using 2-aminobenzamide (680 mg, 5.0 mmol), 1-phenylhept-6-en-1-one (987 mg, 5.3 mmol) and iodine (64 mg, 0.3 mmol) in DMF (8 mL) at 80 °C for 24 hours. The product was obtained as a white solid (1.18 g, 77% yield), by using hexane/EtOAc (3:1) as eluent. **M.p.**: 111 °C. **IR (neat, cm^{-1}):** 3274, 2930, 1639, 1608, 1487. **^{1}H NMR (500 MHz, DMSO-*d*$_6$)** δ 8.67 (s, 1H), 7.49 – 7.44 (m, 4H), 7.27 (t, *J* = 7.6 Hz, 2H), 7.21 – 7.14 (m, 2H), 6.81 (d, *J* = 8.1 Hz, 1H), 6.55 (t, *J* = 7.5 Hz, 1H), 5.79 (ddt, *J* = 17.0, 10.1, 6.7 Hz, 1H), 5.00 (dq, *J* = 17.0, 1.7 Hz, 1H), 4.93 (ddt, *J* = 10.1, 2.3, 1.2 Hz, 1H), 2.02 (q, *J* = 7.1 Hz, 2H), 1.84 – 1.73 (m, 2H), 1.51 (quint., *J* = 7.9 Hz, 2H), 1.39 – 1.30 (m, 2H). **^{13}C NMR (126 MHz, DMSO-*d*$_6$)** δ 164.1, 147.9, 147.5, 138.7, 133.2, 127.9, 127.1, 126.9, 125.3, 116.5, 114.8, 114.7, 114.4, 72.9, 42.2, 33.3, 28.4, 23.4. **HRMS** [ESI$^+$] *calcd.* for ($C_{20}H_{22}N_2NaO$) [M+Na]$^+$: 329.1624, *found*: 329.1619.

(3a*R*,5*R*,6*R*,6a*R*)-5-((*R*)-2,2-Dimethyl-1,3-dioxolan-4-yl)-2,2-dimethyltetrahydrofuro[2,3-*d*][1,3]dioxol-6-yl 4-bromobenzoate (4o)

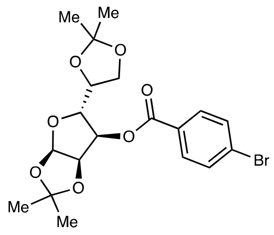

Following GP1, using 4-bromobenzoic acid (1.21 g, 6.0 mmol) and 1,2:5,6-di-o-isopropylidene-*α*-D-allofuranose (1.30 g, 5.0 mmol) were used, affording the title compound as an amorphous white solid (1.63 g, 74% yield), by using hexane/EtOAc (20:1) as chromatography eluent. **IR (neat, cm^{-1}):** 2987, 1724, 1590, 1372, 1268, 1011, 845, 756. **^{1}H NMR (400 MHz, CDCl$_3$)** δ 7.95 – 7.87 (m, 2H), 7.64 – 7.56 (m, 2H), 5.89 (d, *J* = 3.8 Hz, 1H), 5.06 (dd, *J* = 8.1, 5.1 Hz, 1H), 4.95 (dd, *J* = 5.1, 3.9 Hz, 1H), 4.37 – 4.26 (m, 2H), 4.11 (dd, *J* = 8.6, 6.6 Hz, 1H), 3.96 (dd, *J* = 8.6, 5.3 Hz, 1H), 1.54 (s, 3H), 1.39 (s, 3H), 1.33 (s, 3H), 1.33 (s, 3H) ppm. **^{13}C NMR (101 MHz, CDCl$_3$)** δ 165.1, 132.0, 131.5, 128.7, 128.5, 113.4, 110.2, 104.5, 78.0, 78.0, 75.4, 73.7, 66.1, 26.9, 26.8, 26.5, 25.1 ppm. **HRMS** [ESI$^+$] *calcd.* for ($C_{19}H_{23}BrNaO_7$) [M+Na]$^+$: 465.0525, *found*: 465.0521.

3-Chlorophenyl 6-bromohexanoate (7e)

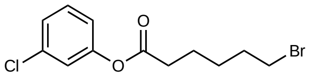

Following GP1, using 6-bromohexanoic acid (0.94 g, 4.8 mmol) and 3-chlorophenol (0.52 g, 4.0 mmol) were used, affording the title compound as a colourless liquid (0.93 g, 77% yield), by using hexane/EtOAc (20:1) as chromatography eluent. **^{1}H NMR (400 MHz, $CDCl_3$)** δ 7.30 (t, *J* = 8.1 Hz, 1H), 7.22 (ddd, *J* = 8.1, 2.0, 1.0 Hz, 1H), 7.12 (t, *J* = 2.1 Hz, 1H), 6.99 (ddd, *J* = 8.1, 2.2, 1.0 Hz, 1H), 3.44 (t, *J* = 6.7 Hz, 2H), 2.58 (t, *J* = 7.4 Hz, 2H), 1.98 – 1.88 (m, 2H), 1.84 – 1.72 (m, 2H), 1.63 – 1.52 (m, 2H) ppm. **^{13}C NMR (101 MHz, $CDCl_3$)** δ 171.6, 151.3, 134.8, 130.3, 126.2, 122.4, 120.1, 34.2, 33.5, 32.5, 27.7, 24.1 ppm. Spectral data in agreement with literature.[4]

6-Bromohexyl furan-2-carboxylate (7g)

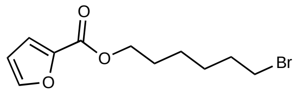

Following GP1, using furan-2-carboxylic acid (0.54 g, 4.8 mmol) and 6-bromohexan-1-ol (0.72 g, 4.0 mmol) were used, affording the title compound as a colourless liquid (0.55g, 50% yield), by using hexane/EtOAc (20:1) as chromatography eluent. **^{1}H NMR (400 MHz, $CDCl_3$)** δ 8.00 (dd, *J* = 1.5, 0.7 Hz, 1H), 7.42 (t, *J* = 1.7 Hz, 1H), 6.73 (dd, *J* = 1.9, 0.7 Hz, 1H), 4.25 (t, *J* = 6.6 Hz, 2H), 3.41 (t, *J* = 6.8 Hz, 2H), 1.94 – 1.82 (m, 2H), 1.79 – 1.68 (m, 2H), 1.55 – 1.39 (m, 4H) ppm. **^{13}C NMR (101 MHz, $CDCl_3$)** δ 163.3, 147.7, 143.8, 119.7, 110.0, 64.5, 33.8, 32.7, 28.7, 27.9, 25.4 ppm. Spectral data was in agreement with the literature.[5]

6-Bromohexyl 3-methylthiophene-2-carboxylate (7i)

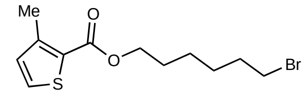

Following GP1, using 3-methylthiophene-2-carboxylic acid (0.68 g, 4.8 mmol) and 6-bromohexan-1-ol (0.72 g, 4.0 mmol) were used, affording the title compound as a colourless liquid (0.55g, 45% yield) by using hexane/EtOAc (20:1) as chromatography eluent. **IR (neat, cm^{-1}):** 2935, 1703, 1414, 1255, 1102, 1071, 767, 725, 609. **^{1}H NMR (400 MHz, $CDCl_3$)** δ 7.38 (d, *J* = 5.0 Hz, 1H), 6.91 (d, *J* = 5.0 Hz, 1H), 4.27 (t, *J* = 6.6 Hz, 2H), 3.41 (t, *J* = 6.8 Hz, 2H), 2.55 (s, 3H), 1.94 – 1.83 (m, 2H), 1.81 – 1.71 (m, 2H), 1.56 – 1.41 (m, 4H) ppm. **^{13}C NMR (101 MHz, $CDCl_3$)** δ 163.0, 146.2, 131.9, 130.1, 127.1, 64.7, 33.8, 32.8, 28.7, 27.9, 25.4, 16.1 ppm. **HRMS** [ESI$^+$] *calcd.* for ($C_{12}H_{17}BrNaO_2S$) $[M+Na]^+$: 327.0030, *found*: 327.0028.

Synthesis of (8*R*,9*S*,13*S*,14*S*)-3-(3-Bromopropoxy)-13-methyl-6,7,8,9,11,12,13,14,15,16-decahydro-17*H*-cyclopenta[*a*]phenanthren-17-one (7j)

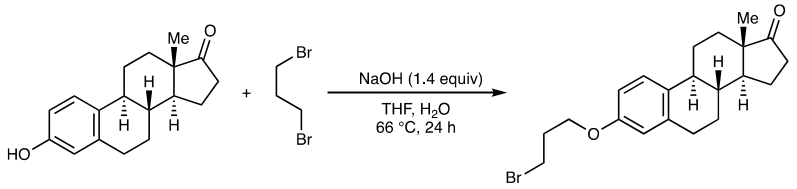

A 50 mL RBF was charged with a stirrer bar, estrone (1.0 g, 3.7 mmol), sodium hydroxide (200 mg, 5.0 mmol), THF (20 mL) and water (5 mL). 1,3-Dibromopropane (0.508 mL, 5.0 mmol) was added to the reaction mixture, which was stirred for 24 hours at 66 °C. The reaction mixture was quenched with 2.7 M HCl (aq, 75 mL) and extracted with EtOAc (2 x 75 mL). The combined organic extracts were dried over $MgSO_4$ and concentrated under reduced giving a light brown oil as crude material, which was purified by silica gel chromatography (0 to 10% Et_2O in hexane) and then recrystallization (MeOH), affording the title compound (186 mg, 12%) as white feathered crystals. **M.p.**: 103 °C. **^{1}H NMR (400 MHz, $CDCl_3$)** δ 7.20 (d, *J* = 8.5 Hz, 1H), 6.72 (dd, *J* = 8.5, 2.8 Hz, 1H), 6.66 (d, *J* = 2.6 Hz, 1H), 4.08 (t, *J* = 5.8 Hz, 2H), 3.60 (t, *J* = 6.5 Hz, 2H), 2.94 – 2.87 (m, 2H), 2.50 (dd, *J* = 18.6, 8.7 Hz, 1H), 2.43 – 2.37 (m, 1H), 2.35 – 2.22 (m, 3H), 2.20 – 1.93 (m, 4H), 1.68 – 1.40 (m, 6H), 0.91 (s, 3H) ppm. **^{13}C NMR (126 MHz, $CDCl_3$)** δ 220.9, 156.9, 138.0, 132.5, 126.5, 114.7, 112.3, 65.4, 50.6, 48.2, 44.1, 38.5, 36.0, 32.6,

31.7, 30.2, 29.8, 26.7, 26.1, 27.7, 14.0 ppm. Spectral data was in agreement with the literature.[6]

Synthesis of (3*S*,8*S*,9*S*,10*R*,13*R*,14*S*,17*R*)-10,13-Dimethyl-17-((*R*)-6-methylheptan-2-yl)-2,3,4,7,8,9,10,11,12,13,14,15,16,17-tetradecahydro-1*H*-cyclopenta[a]phenanthren-3-yl 6-bromohexanoate (7k)

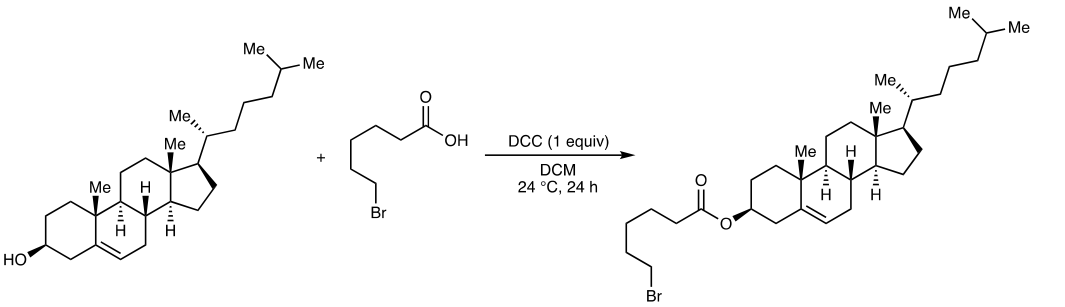

To a 100 mL RBF was sequentially added cholesterol (3.0 g, 7.7 mmol), *N*,*N'*-dicyclohexylmethanediimine (1.6 g, 7.7 mmol), DMAP (40 mg) and DCM (40 mL). The reaction mixture was stirred for 2 minutes at 24 °C, then 6-bromohexanoic acid (1.5 g, 7.7 mmol) was added and the reaction was stirred for another 18 hours at 24 °C during which a white precipitate formed. The reaction mixture was filtered and the collected organic filtrate was concentrated under reduced pressure affording a white solid as crude product, which was purified by recrystallization (EtOH) yielding the title compound (2.65 g, 61%) as white crystals. **M.p.**: 122 °C. **^{1}H NMR (400 MHz, $CDCl_3$)** δ 5.37 (br. d, *J* = 4.7 Hz, 1H), 4.67 – 4.56 (m, 1H), 3.40 (t, *J* = 6.8 Hz, 1H), 2.35 – 2.25 (m, 4H), 2.04 – 1.93 (m, 2H), 1.91 – 1.78 (m, 5H), 1.69 – 1.28 (m, 15H), 1.19 – 0.94 (m, 13H), 0.91 (d, *J* = 6.5 Hz, 3H), 0.87 (d, *J* = 1.8, Hz, 3H), 0.87 (d, *J* = 1.8, Hz, 3H), 0.67 (s, 3H) ppm. **^{13}C NMR (126 MHz, $CDCl_3$)** δ 173.0, 139.8, 122.8, 74.0, 56.8, 56.3, 50.2, 42.5, 39.9, 39.7, 38.3, 37.1, 36.7, 36.3, 35.9, 34.6, 33.6, 32.6, 32.0, 32.0, 28.4, 28.2, 28.0, 27.8, 24.4, 24.3, 24.0, 23.0, 22.7, 21.2, 19.5, 18.9, 12.0 ppm. Spectral data was in agreement with the literature.[7]

Synthesis of 1-(4-bromopiperidin-1-yl)-3-(4,5-diphenyloxazol-2-yl)propan-1-one (7p)

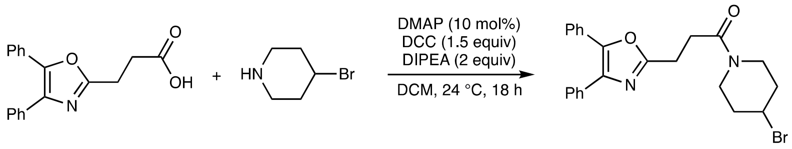

A 27 mL vial was charged with a stirrer bar, 4-bromopiperidine hydrobromide (486 mg, 2.0 mmol), *N,N'*-dicyclohexylmethanediimine (619 mg, 3.0 mmol) and DMAP (24.4 mg, 0.2 mmol), then equipped with a rubber septum with an argon inlet. The contents of the reaction vessel were flushed with argon, then charged with DCM (4 mL, anhydrous) and DIPEA (0.70 mL, 4.0 mmol). A solution of oxaprozin (486 mg, 2.0 mmol) in DCM (1 mL, anhydrous) was prepared under an inert atmosphere, which was added to the reaction mixture over 5 minutes. The reaction mixture was stirred for 18 hours at 24 °C then concentrated under reduced pressure yielding a white residue, which was suspended in 2 M NaOH (aq, 50 mL) and extracted with EtOAc (3 x 40 mL). The combined organic extracts were washed with 1 M HCl (aq, 50 mL), dried ($MgSO_4$) and concentrated under reduced pressure giving a white solid as crude material, which was purified by silica gel chromatography (0 to 50% EtOAc in hexane), affording the title compound (475 mg, 54%) as a colourless oil.**^{1}H NMR (500 MHz, $CDCl_3$)** δ 7.64 – 7.60 (m, 2H), 7.58 – 7.55 (m, 2H), 7.39 – 7.28 (m, 6H), 4.40 (sep., *J* = 3.6 Hz, 1H), 3.83 (ddd, 13.5, 7.7, 3.6 Hz, 1H), 3.76 (ddd, *J* = 13.9, 7.7, 3.4 Hz, 1H), 3.63 (ddd, *J* = 13.6, 7.1, 3.8 Hz, 1H), 3.47 (ddd, *J* = 13.9, 7.3, 3.6 Hz, 1H), 3.22 (dd, *J* = 8.5, 6.8 Hz, 2H), 2.94 (dd, *J* = 8.0, 6.8 Hz, 2H), 2.18 – 2.06 (m, 2H), 2.04 – 1.91 (m, 2H) ppm. **^{13}C NMR (126 MHz, $CDCl_3$)** δ 169.6, 162.8, 145.5, 135.1, 132.5, 130.0, 129.2, 129.0, 128.8, 128.7, 128.6, 128.2, 128.1, 126.6, 49.0, 43.7, 40.1, 36.0, 35.4, 29.9, 23.9 ppm. Spectral data was in agreement with the literature.[8]

5. Synthesis of Products

Methyl 4-cyclohexylbenzoate (3a)

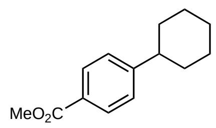

Following GP4, methyl 4-bromobenzoate (43.0 mg, 0.20 mmol) and 2-cyclohexyl-2-methyl-2,3-dihydroquinazolin-4(1*H*)-one (**2a**) (58.6 mg, 0.24 mmol) were used, affording the title compound as a colourless liquid (41.0 mg, 94% yield), by using hexane/EtOAc (100:1) as chromatography eluent. In a second independent experiment, 40.7 mg (93%) were obtained, giving an average of 93% yield. **^{1}H NMR (400 MHz, $CDCl_3$)** δ 7.98 – 7.93 (m, 2H), 7.30 – 7.24 (m, 2H), 3.89 (s, 3H), 2.62 – 2.49 (m, 1H), 1.93 – 1.80 (m, 4H), 1.80 – 1.71 (m, 1H), 1.49 – 1.33 (m, 4H), 1.32 – 1.19 (m, 1H) ppm. **^{13}C NMR (101 MHz, $CDCl_3$)** δ 167.3, 153.6, 129.8, 127.9, 127.0, 52.1, 44.8, 34.3, 26.9, 26.2 ppm. Spectral data was in agreement with the literature.[9]

Following GP4, methyl 4-bromobenzoate (43.0 mg, 0.20 mmol) and 2-cyclohexyl-2-ethyl-2,3-dihydroquinazolin-4(1*H*)-one (**2a´**) (61.9 mg, 0.24 mmol) were used, affording the title compound as a colourless liquid (40.8 mg, 94% yield), by using hexane/EtOAc (100:1) as chromatography eluent. In a second independent experiment, 40.6 mg (93%) were obtained, giving an average of 93% yield. **^{1}H NMR (400 MHz, $CDCl_3$)** δ 8.00 – 7.93 (m, 2H), 7.31 – 7.24 (m, 2H), 3.90 (s, 3H), 2.62 – 2.50 (m, 1H), 1.95 – 1.81 (m, 4H), 1.81 – 1.72 (m, 1H), 1.51 – 1.34 (m, 4H), 1.32 – 1.20 (m, 1H) ppm. **^{13}C NMR (101 MHz, $CDCl_3$)** δ 167.3, 153.6, 129.8, 127.9, 127.0, 52.1, 44.8, 34.3, 26.9, 26.2 ppm.

Methyl 4-cyclopentylbenzoate (3b)

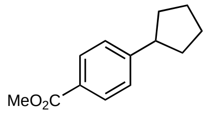

Following GP4, methyl 4-bromobenzoate (43.0 mg, 0.20 mmol) and 2-cyclopentyl-2-methyl-2,3-dihydroquinazolin-4(1*H*)-one (55.2 mg, 0.24 mmol) were used, affording the title compound as a colourless liquid (36.4 mg, 89% yield) by using hexane/EtOAc (100:1) as eluent. In a second independent experiment, 37.3 mg (91%) were obtained, giving an average of 90% yield. **^{1}H NMR (400 MHz, $CDCl_3$)** δ 7.98 – 7.92 (m, 2H), 7.32 – 7.27 (m, 2H), 3.90 (s, 3H), 3.04 (tt, *J* = 9.6, 7.5 Hz, 1H), 2.14 – 2.04 (m, 2H), 1.89 – 1.53 (m, 6H) ppm. **^{13}C NMR (101 MHz, $CDCl_3$)** δ 167.3, 152.4, 129.7, 127.8, 127.3, 52.1, 46.1, 34.6, 25.7 ppm. Spectral data was in agreement with the literature.[9]

Methyl 4-cycloheptylbenzoate (3c)

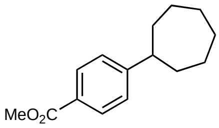

Following GP4, methyl 4-bromobenzoate (43.0 mg, 0.20 mmol) and 2-cycloheptyl-2-methyl-2,3-dihydroquinazolin-4(1*H*)-one (61.9 mg, 0.24 mmol) were used, affording the title compound as a colourless liquid (35.0 mg, 75% yield), by using hexane/EtOAc (100:1) as chromatography eluent. In a second independent experiment, 34.5 mg (74%) were obtained, giving an average of 75% yield. **^{1}H NMR (400 MHz, $CDCl_3$)** δ 7.98 – 7.90 (m, 2H), 7.29 – 7.21 (m, 2H), 3.89 (s, 3H), 2.71 (tt, *J* = 10.5, 3.6 Hz, 1H), 1.97 – 1.85 (m, 2H), 1.85 – 1.76 (m, 2H), 1.75 – 1.48 (m, 8H) ppm. **^{13}C NMR (101 MHz, $CDCl_3$)** δ 167.3, 155.5, 129.9, 127.6, 126.8, 52.0, 47.2, 36.6, 28.0, 27.4 ppm. Spectral data was in agreement with the literature.[11]

Methyl 4-((3*S*)-3-(2-methoxy-2-oxoethyl)-2,2-dimethylcyclobutyl)benzoate (3d)

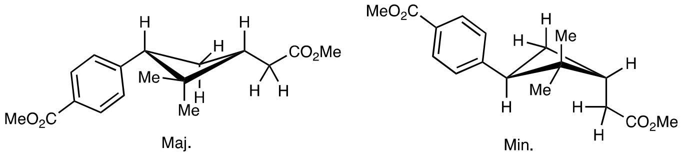

Following GP4, methyl 4-bromobenzoate (43.0 mg, 0.20 mmol) and methyl 2-((1*R*)-2,2-dimethyl-3-(2-methyl-4-oxo-1,2,3,4-tetrahydroquinazolin-2-yl)cyclobutyl)acetate (75.8 mg, 0.24 mmol) were used, affording the title compound as a colourless oil (30.9 mg, 53% yield, 2.3:1 d.r.), by using hexane/EtOAc (10:1) as chromatography eluent. In a second independent experiment, 30.1 mg (52%, 2.3:1 d.r.) were obtained, giving an average of 53% yield. **IR (film, cm^{-1}) ν_{max}** = 2952, 1720, 1435, 1277, 1179, 1110. **^{1}H NMR (500 MHz, $CDCl_3$, mixture of diasteromers A:B in a 0.7:0.3 ratio)** δ 8.01 – 7.69 (m, 2H), 7.23 (d, *J* = 8.1 Hz, 0.6H), 7.18 (d, *J* = 8.1 Hz, 1.4H), 3.93 (s, 0.9H), 3.92 (s, 2.1H), 3.32 (t, *J* = 8.3 Hz, 0.3H), 3.24 (dd, *J* = 11.0, 7.5 Hz, 0.7H), 2.62 (dd, *J* = 15.4, 6.7 Hz, 0.3H), 2.59 – 2.55 (m, 0.3H), 2.54 (d, *J* = 8.9 Hz, 0.3H), 2.50 – 2.44 (m, 0.7H), 2.43 (d, *J* = 7.1 Hz, 0.3H), 2.40 (d, *J* = 7.2 Hz, 0.7H), 2.38 (d, *J* = 7.9 Hz, 0.7H), 2.31 (td, *J* = 7.6, 3.2 Hz, 0.7H), 2.05 – 1.96 (m, 1H), 1.28 (s, 2.1H), 1.17 (s, 0.9H), 0.71 (s, 0.9H), 0.60 (s, 2.1H) ppm. **^{13}C NMR (126 MHz, $CDCl_3$)** δ 173.8 (Min.), 173.6 (Maj.), 167.3 (Maj. + Min.), 147.4 (Min.), 146.5 (Maj.), 129.5 (Min.), 129.5 (Maj.), 128.0 (Maj. + Min.), 127.65 (Min.), 127.4 (Maj.), 76.9 (Maj.), 52.1 (Maj. + Min.), 51.7 (Min.), 51.7 (Maj.), 47.8 (Maj.), 47.2 (Min.), 43.8 (Maj.), 41.8 (Min.), 38.5 (Maj.), 38.1 (Min.), 36.0 (Min.), 35.2 (Maj.), 30.1 (Maj.), 26.8 (Maj.), 26.0 (Min.), 25.1 (Min.), 24.9 (Min.), 17.7 (Maj.) ppm. **HRMS** [ESI^+] *calcd.* for ($C_{17}H_{22}NaO_4$) $[M+Na]^+$: 313.1410, *found* 313.1405.
NOESY:

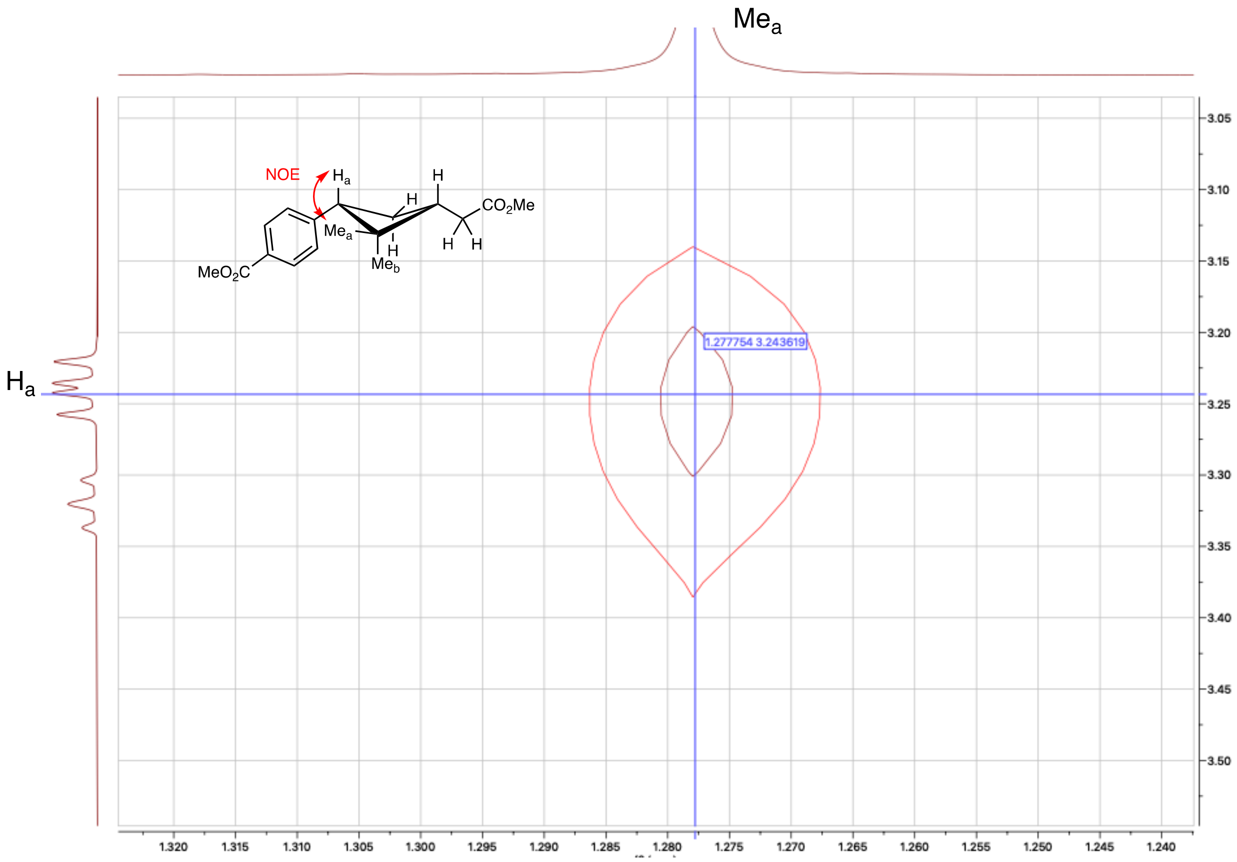

Supplementary Figure 1. NOESY NMR spectra of compound **3d**.

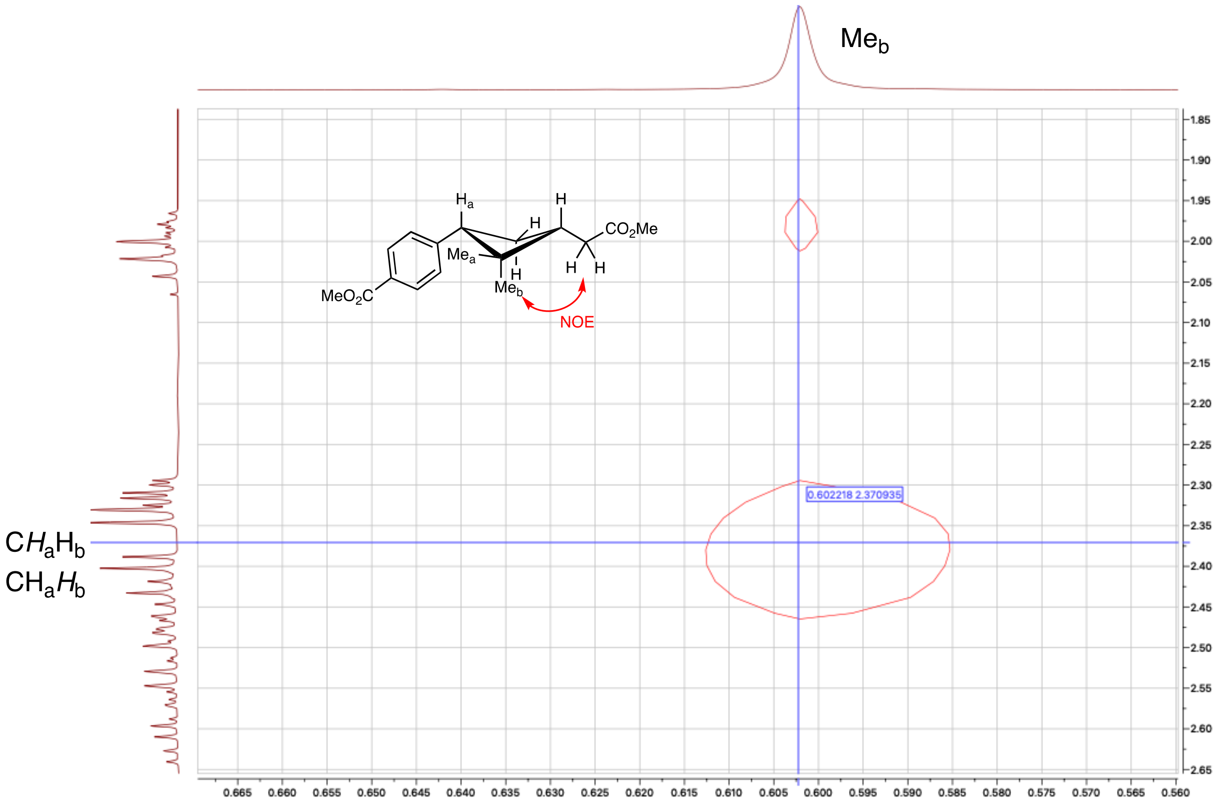

Supplementary Figure 2. NOESY NMR spectra of compound **3d**.

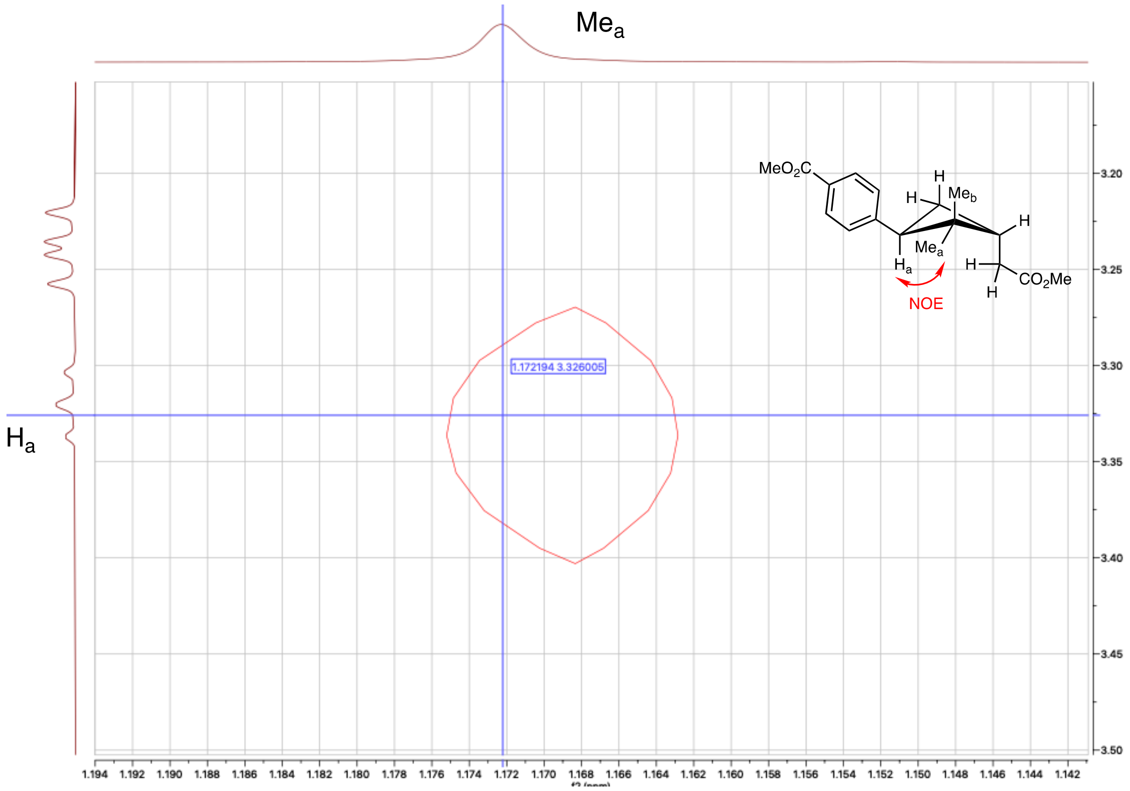

Supplementary Figure 3. NOESY NMR spectra of compound **3d**.

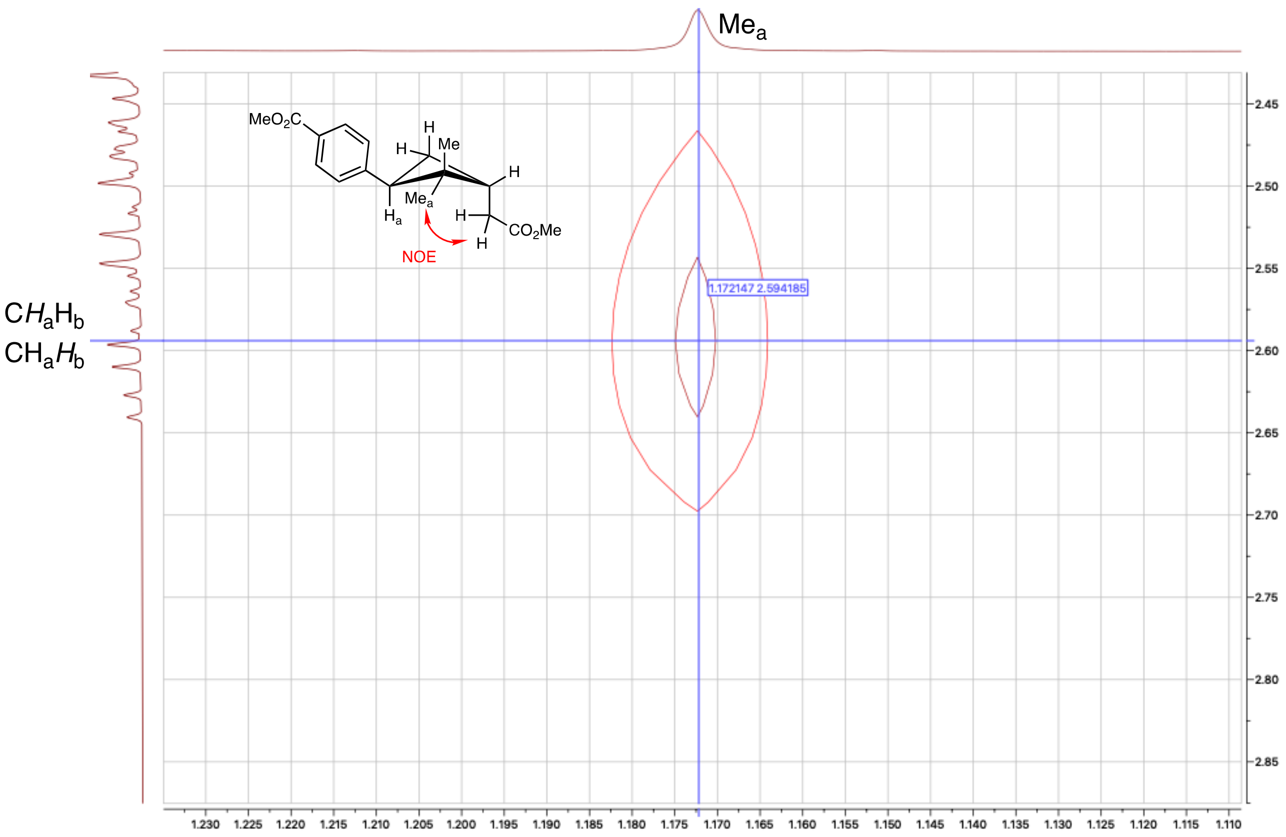

Supplementary Figure 4. NOESY NMR spectra of compound **3d**.

Methyl 4-(tetrahydro-2*H*-pyran-4-yl)benzoate (3e)

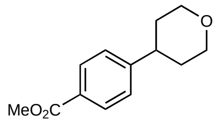

Following GP4, methyl 4-bromobenzoate (43.0 mg, 0.20 mmol) and 2-methyl-2-(tetrahydro-2*H*-pyran-4-yl)-2,3-dihydroquinazolin-4(1*H*)-one (59.0 mg, 0.24 mmol) were used, affording the title compound as a white solid (44.6 mg, 99% yield), by using hexane/EtOAc (50:1) as chromatography eluent. In a second independent experiment, 44.3 mg (99%) were obtained, giving an average of 99% yield. **M.p.**: 71 °C. **^{1}H NMR (400 MHz, CDCl$_3$)** δ 8.02 – 7.95 (m, 2H), 7.32 – 7.24 (m, 2H), 4.12 – 4.04 (m, 2H), 3.91 (s, 3H), 3.58 – 3.47 (m, 2H), 2.87 – 2.75 (m, 1H), 1.89 – 1.71 (m, 4H) ppm. **^{13}C NMR (101 MHz, CDCl$_3$)** δ 167.1, 151.2, 130.0, 128.4, 126.9, 68.3, 52.1, 41.8, 33.7 ppm. Spectral data was in agreement with the literature.[11]

Following GP4, methyl 4-(((trifluoromethyl)sulfonyl)oxy)benzoate (56.8 mg, 0.20 mmol) and 2-methyl-2-(tetrahydro-2H-pyran-4-yl)-2,3-dihydroquinazolin-4(1*H*)-one (59.0 mg, 0.24 mmol) were used, affording the title compound as a white solid (21.6 mg, 49% yield), by using hexane/EtOAc (20:1) as chromatography eluent. In a second independent experiment, 23.0 mg (52%) were obtained, giving an average of 51% yield. **^{1}H NMR (400 MHz, CDCl$_3$)** δ 8.02 – 7.95 (m, 2H), 7.32 – 7.29 (m, 2H), 4.14 – 4.04 (m, 2H), 3.90 (s, 3H), 3.53 (m, 2H), 2.88 – 2.76 (m, 1H), 1.90 – 1.71 (m, 4H) ppm. **^{13}C NMR (101 MHz, CDCl$_3$)** δ 167.2, 151.2, 130.0, 128.5, 126.9, 68.4, 52.2, 41.8, 33.7 ppm.

***tert*-Butyl 4-(4-(methoxycarbonyl)phenyl)piperidine-1-carboxylate (3f)**

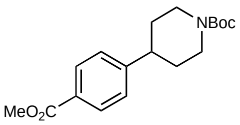

Following GP4, methyl 4-bromobenzoate (43.0 mg, 0.20 mmol) and *tert*-butyl 4-(2-methyl-4-oxo-1,2,3,4-tetrahydroquinazolin-2-yl)piperidine-1-carboxylate (82.8 mg, 0.24 mmol) were used, affording the title compound as a white solid (44.4 mg, 70% yield), by using hexane/EtOAc (30:1) as chromatography eluent. In an independent experiment, 39.8 mg (62%) were obtained, giving an average of 66% yield. **M.p.**: 79 °C. **^{1}H NMR (400 MHz, CDCl$_3$)** δ 8.00 – 7.95 (m, 2H), 7.29 – 7.25 (m, 2H), 4.26 (d, *J* = 13.2 Hz, 2H), 3.90 (s, 3H), 2.81 (td, *J* = 13.2, 2.5 Hz, 2H), 2.76 – 2.65 (m, 1H), 1.83 (d, *J* = 13.5 Hz, 2H), 1.70 – 1.57 (m, 2H), 1.48 (s, 9H) ppm. **^{13}C NMR (101 MHz, CDCl$_3$)** δ 167.1, 155.0, 151.2, 130.0, 128.5, 127.0, 79.7, 52.2, 44.4, 43.0, 33.0, 28.6 ppm. Spectral data was in agreement with the literature.[11]

Methyl 4-((1,3-dioxoisoindolin-2-yl)methyl)benzoate (3g)

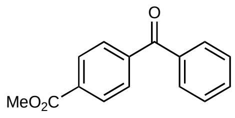

Following GP4, methyl 4-bromobenzoate (43.0 mg, 0.20 mmol) and 2-benzoyl-2-methyl-2,3-dihydroquinazolin-4(1*H*)-one (63.8 mg, 0.24 mmol) were used, affording the title compound as a white solid (24.7 mg, 51% yield) by using hexane/EtOAc (20:1) as chromatography eluent. In a second independent experiment, 24.5 mg (51%) were obtained, giving an average of 51% yield. **M.p.**: 110 °C. **^{1}H NMR (400 MHz, $CDCl_3$)** δ 8.18 – 8.12 (m, 2H), 7.87 – 7.78 (m, 4H), 7.65 – 7.58 (m, 1H), 7.54 – 7.46 (m, 2H), 3.97 (s, 3H) ppm. **^{13}C NMR (101 MHz, $CDCl_3$)** δ 196.2, 166.5, 141.5, 137.1, 133.4, 133.1, 130.3, 129.9, 129.7, 128.6, 52.6 ppm. Spectral data was in agreement with the literature.[10]

Methyl 4-(dimethoxymethyl)benzoate (3h)

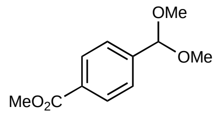

Following GP4, methyl 4-bromobenzoate (43.0 mg, 0.20 mmol) and 2-(dimethoxymethyl)-2-methyl-2,3-dihydroquinazolin-4(1*H*)-one (56.6 mg, 0.24 mmol) were used, affording the title compound as a white solid (30.8 mg, 73% yield), by using hexane/EtOAc (20:1) as chromatography eluent. In a second independent experiment, 32.1 mg (76%) were obtained, giving an average of 75% yield. **M.p.**: 206 °C. **^{1}H NMR (400 MHz, $CDCl_3$)** δ 8.07 – 7.97 (m, 2H), 7.52 (d, *J* = 8.7 Hz, 2H), 5.43 (s, 1H), 3.91 (s, 3H), 3.32 (s, 6H) ppm. **^{13}C NMR (101 MHz, $CDCl_3$)** δ 167.0, 143.1, 130.4, 129.7, 126.9, 102.5, 52.8, 52.3 ppm. Spectral data was in agreement with the literature.[11]

Methyl 4-((1,3-dioxoisoindolin-2-yl)methyl)benzoate (3i)

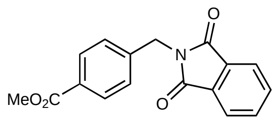

Following GP4, methyl 4-bromobenzoate (43.0 mg, 0.20 mmol) and 2-((2-methyl-4-oxo-1,2,3,4-tetrahydroquinazolin-2-yl)methyl)isoindoline-1,3-dione (77.0 mg, 0.24 mmol) were used, affording the title compound as a white solid (46.1 mg, 78% yield), by using hexane/EtOAc (6:1) as chromatography eluent. In a second independent experiment, 42.1 mg (71%) were obtained, giving an average of 75% yield. **M.p.**: 153 °C. **^{1}H NMR (400 MHz, $CDCl_3$)** δ 8.01 – 7.96 (m, 2H), 7.89 – 7.83 (m, 2H), 7.75 – 7.69 (m, 2H), 7.47 (d, *J* = 8.5 Hz, 2H), 4.89 (s, 2H), 3.89 (s, 3H) ppm. **^{13}C NMR (101 MHz, $CDCl_3$)** δ 168.1, 166.9, 141.4, 134.3, 132.2, 130.2, 129.8, 128.6, 123.6, 52.3, 41.4 ppm. Spectral data was in agreement with the literature.[12]

Methyl 4-(acetoxymethyl)benzoate (3j)

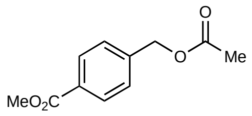

Following GP4, methyl 4-bromobenzoate (43.0 mg, 0.20 mmol) and (2-methyl-4-oxo-1,2,3,4-tetrahydroquinazolin-2-yl)methyl acetate (56.2 mg, 0.24 mmol) were used, affording the title compound as a colourless liquid (15.5 mg, 37% yield), by using hexane/EtOAc (20:1) as chromatography eluent. In a second independent experiment, 17.3 mg (42%) were obtained, giving an average of 39% yield. **^{1}H NMR (400 MHz, $CDCl_3$)** δ 8.05 – 8.01 (m, 2H), 7.44 – 7.39 (m, 2H), 5.15 (s, 2H), 3.92 (s, 3H), 2.13 (s, 3H) ppm. **^{13}C NMR (101 MHz, $CDCl_3$)** δ 170.8, 166.9, 141.1, 130.1, 123.0, 127.8, 65.6, 52.3, 21.1 ppm. Spectral data was in agreement with the literature.[13]

Methyl 4-(methoxymethyl)benzoate (3k)

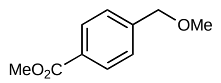

Following GP4, methyl 4-bromobenzoate (43.0 mg, 0.20 mmol) and 2-(methoxymethyl)-2-methyl-2,3-dihydroquinazolin-4(1*H*)-one (49.4 mg, 0.24 mmol) were used, affording the title compound as a colourless liquid (28.5 mg, 79% yield), by using hexane/EtOAc (50:1) as chromatography eluent. In a second independent experiment, 28.3 mg (79%) were obtained, giving an average of 79% yield. **^{1}H NMR (400 MHz, $CDCl_3$)** δ 8.05 – 7.99 (m, 2H), 7.43 – 7.37 (m, 2H), 4.51 (s, 2H), 3.91 (s, 3H), 3.41 (s, 3H) ppm. **^{13}C NMR (101 MHz, $CDCl_3$)** δ 167.1, 143.7, 129.9, 129.5, 127.3, 74.2, 58.5, 52.2 ppm. Spectral data was in agreement with the literature.[14]

Methyl 4-(phenoxymethyl)benzoate (3l)

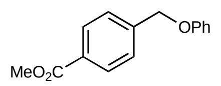

Following GP4, methyl 4-bromobenzoate (43.0 mg, 0.20 mmol) and 2-methyl-2-(phenoxymethyl)-2,3-dihydroquinazolin-4(1*H*)-one (64.3 mg, 0.24 mmol) were used, affording the title compound as a white solid (39.3 mg, 81% yield), by using hexane/EtOAc (50:1) as chromatography eluent. In a second independent experiment, 39.1 mg (81%) were obtained, giving an average of 81% yield. **M.p.**: 88 °C. **^{1}H NMR (400 MHz, $CDCl_3$)** δ 8.09 – 8.04 (m, 2H), 7.51 (d, *J* = 8.1 Hz, 2H), 7.34 – 7.27 (m, 2H), 7.01 – 6.95 (m, 3H), 5.13 (s, 2H), 3.93 (s, 3H) ppm. **^{13}C NMR (101 MHz, $CDCl_3$)** δ 167.0, 158.6, 142.5, 130.0, 129.8, 129.7, 127.1, 121.4, 115.0, 69.4, 52.2 ppm. Spectral data was in agreement with the literature.[15]

Following GP4, methyl 4-bromobenzoate (43.0 mg, 0.20 mmol) and 2-ethyl-2-(phenoxymethyl)-2,3-dihydroquinazolin-4(1*H*)-one (67.7 mg, 0.24 mmol) were used, affording the title compound as a white solid (39.4 mg, 81% yield), by using hexane/EtOAc (50:1) as chromatography eluent. In a second independent experiment, 39.8 mg (82%) were obtained, giving an average of 82% yield. **^{1}H NMR (400 MHz, $CDCl_3$)** δ 8.10 – 8.03 (m, 2H), 7.55 – 7.48 (m, 2H), 7.34 – 7.27 (m, 2H), 7.02 – 6.94 (m, 3H), 5.13 (s, 2H), 3.93 (s, 3H) ppm. **^{13}C NMR (101 MHz, $CDCl_3$)** δ 167.0, 158.6, 142.5, 130.0, 129.8, 129.7, 127.1, 121.4, 115.0, 69.4, 52.3 ppm.

Methyl 4-butylbenzoate (3m)

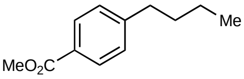

Following GP4, methyl 4-bromobenzoate (43.0 mg, 0.20 mmol) and 2-butyl-2-phenyl-2,3-dihydroquinazolin-4(1*H*)-one (**2m´**) (67.2 mg, 0.24 mmol) were used, affording the title compound as a colourless liquid (33.8 mg, 88% yield), by using hexane/EtOAc (100:1) as chromatography eluent. In a second independent experiment, 35.5 mg (92%) were obtained, giving an average of 90% yield. **^{1}H NMR (400 MHz, $CDCl_3$)** δ 7.97 – 7.91 (m, 2H), 7.25 – 7.21 (m, 2H), 3.90 (s, 3H), 2.66 (t, *J* = 7.7 Hz, 2H), 1.67 – 1.55 (m, 2H), 1.41 – 1.29 (m, 2H), 0.93 (t, *J* = 7.3 Hz, 3H) ppm. **^{13}C NMR (101 MHz, $CDCl_3$)** δ 167.3, 148.6, 129 .8, 128.6, 127.8, 52.1, 35.8, 33.4, 22.4, 14.0 ppm. Spectral data was in agreement with the literature.[16]

Following GP4, methyl 4-bromobenzoate (43.0 mg, 0.20 mmol) and 2-butyl-2-(4-methoxyphenyl)-2,3-dihydroquinazolin-4(1*H*)-one (**2m´´**) (74.4 mg, 0.24 mmol) were used, affording the title compound as a colourless liquid (30.8 mg, 80% yield), by using hexane/EtOAc (100:1) as chromatography eluent. In a second independent experiment, 30.7 mg (80%) were obtained, giving an average of 80% yield. **^{1}H NMR (400 MHz, $CDCl_3$)** δ 7.98 – 7.91 (m, 2H), 7.26 – 7.21 (m, 2H), 3.90 (s, 3H), 2.70 – 2.62 (m, 2H), 1.67 – 1.56 (m, 2H), 1.41 – 1.29 (m, 2H), 0.93 (t, *J* = 7.3 Hz, 3H) ppm. **^{13}C NMR (101 MHz, $CDCl_3$)** δ 167.3, 148.6, 129.8, 128.6, 127.8, 52.1, 35.8, 33.4, 22.4, 14.0 ppm.

Following GP4, methyl 4-bromobenzoate (43.0 mg, 0.20 mmol) and 2-(benzo[d][1,3]dioxol-5-yl)-2-butyl-2,3-dihydroquinazolin-4(1*H*)-one (**2m´´´**) (77.8 mg, 0.24 mmol) were used, affording the title compound as a colourless liquid (30.6 mg, 80% yield), by using hexane/EtOAc (100:1) as chromatography eluent. In a second independent experiment, 29.7 mg (77%) were obtained, giving an average of 79% yield. **^{1}H NMR (400 MHz, $CDCl_3$)** δ 7.97 – 7.92 (m, 2H), 7.26 – 7.21 (m, 2H), 3.90 (s, 3H), 2.70 – 2.62 (m, 2H), 1.66 – 1.56 (m, 2H), 1.41 – 1.30 (m, 2H), 0.93 (t, J = 7.3 Hz, 3H) ppm. **^{13}C NMR (101 MHz, $CDCl_3$)** δ 167.4, 148.6, 129.8, 128.6, 127.8, 52.1, 35.8, 33.4, 22.5, 14.0 ppm.

Methyl 4-(4-isopropoxy-4-oxobutyl)benzoate (3n)

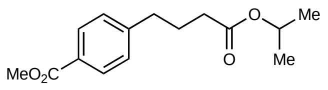

Following GP4, methyl 4-bromobenzoate (43.0 mg, 0.20 mmol) and isopropyl 4-(4-oxo-2-phenyl-1,2,3,4-tetrahydroquinazolin-2-yl)butanoate (84.5 mg, 0.24 mmol) were used, affording the title compound as a colourless liquid (22.9 mg, 43% yield), by using hexane/EtOAc (40:1) as chromatography eluent. In a second independent experiment, 24.2 mg (46%) were obtained, giving an average of 45% yield. **IR (neat, cm^{-1}):** 2980, 1719, 1610, 1435, 1374, 1275, 1178, 1105, 763. **^{1}H NMR (400 MHz, $CDCl_3$)** δ 7.98 – 7.92 (m, 2H), 7.24 (d, *J* = 8.2 Hz, 2H), 5.01 (hept, *J* = 6.3 Hz, 1H), 3.90 (s, 3H), 2.74 – 2.66 (m, 2H), 2.28 (t, *J* = 7.4 Hz, 2H), 2.00 – 1.90 (m, 2H), 1.23 (d, *J* = 6.3 Hz, 6H) ppm. **^{13}C NMR (101 MHz, $CDCl_3$)** δ 172.9, 167.2, 147.2, 129.9, 128.7, 128.2, 67.8, 52.1, 35.3, 34.0, 26.4, 22.0 ppm. **HRMS** [ESI^+] *calcd.* for ($C_{15}H_{20}NaO_4$) $[M+Na]^+$: 287.1259, *found*: 287.1252.

Methyl 4-(2-(benzo[*d*][1,3]dioxol-5-yl)ethyl)benzoate (3o)

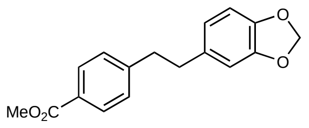

Following GP4, methyl 4-bromobenzoate (43.0 mg, 0.20 mmol) and 2-(2-(benzo[*d*][1,3]dioxol-5-yl)ethyl)-2-phenyl-2,3-dihydroquinazolin-4(1*H*)-one (89.3 mg, 0.24 mmol) were used, affording the title compound as a white solid (32.0 mg, 56% yield), by using hexane/EtOAc (20:1) as chromatography eluent. In a second independent experiment, 34.3 mg (60%) were obtained, giving an average of 58% yield. **M.p.**: 83 °C. **^{1}H NMR (400 MHz, $CDCl_3$)** δ 7.97 – 7.92 (m, 2H), 7.21 (d, *J* = 8.4 Hz, 2H), 6.71 (d, *J* = 7.9 Hz, 1H), 6.64 (d, *J* = 1.6 Hz, 1H), 6.57 (dd, *J* = 7.9, 1.7 Hz, 1H), 5.92 (s, 2H), 3.90 (s, 3H), 2.97 – 2.89 (m, 2H), 2.89 – 2.82 (m, 2H) ppm. **^{13}C NMR (101 MHz, $CDCl_3$)** δ 167.3, 147.7, 147.2, 145.9, 135.1, 129.8, 128.7, 128.1, 121.4, 109.0, 108.3, 100.9, 52.1, 38.3, 37.3 ppm. Spectral data was in agreement with the literature.[17]

Methyl 4-(4-morpholino-4-oxobutyl)benzoate (3p)

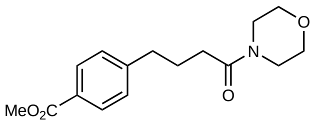

Following GP4, methyl 4-bromobenzoate (43.0 mg, 0.20 mmol) and 2-(4-morpholino-4-oxobutyl)-2-phenyl-2,3-dihydroquinazolin-4(1*H*)-one (91.0 mg, 0.24 mmol) were used, affording the title compound as a white solid (52.2 mg, 90% yield), by using hexane/EtOAc (3:1) as chromatography eluent. In a second independent experiment, 49.6 mg (85%) were obtained, giving an average of 87% yield. **M.p.**: 68 °C. **IR (neat, cm^{-1}):** 2956, 2865, 1712, 1639, 1608, 1432, 1275, 1187, 1109, 1028, 961, 833, 762. **^{1}H NMR (400 MHz, $CDCl_3$)** δ 7.92 – 7.86 (m, 2H), 7.19 (d, *J* = 8.1 Hz, 2H), 3.83 (s, 3H), 3.57 (s, 6H), 3.31 (s, 2H), 2.67 (t, *J* = 7.5 Hz, 2H), 2.23 (t, *J* = 7.4 Hz, 2H), 1.99 – 1.88 (m, 2H) ppm. **^{13}C NMR (101 MHz, $CDCl_3$)** δ 171.2, 167.2, 147.3, 129.9, 128.6, 128.2, 67.0, 66.8, 52.1, 46.0, 42.0, 35.4, 32.1, 26.3 ppm. **HRMS** [ESI$^+$] *calcd.* for ($C_{16}H_{21}NNaO_4$) $[M+Na]^+$: 314.1368, *found*: 314.1354.

Methyl 4-(4-((3a*R*,4*R*,6a*S*)-2-oxohexahydro-1*H*-thieno[3,4-d]imidazol-4-yl)butyl)benzoate (3q)

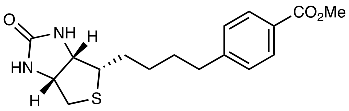

An oven-dried 8 mL screw-cap test tube containing a stirring bar was charged with (3a*S*,4*S*,6a*R*)-4-(4-(4-oxo-2-phenyl-1,2,3,4-tetrahydroquinazolin-2-yl)butyl)tetrahydro-1*H*-thieno[3,4-*d*]imidazol-2(3*H*)-one (**2r**) (101.4 mg, 0.24 mmol), methyl 4-bromobenzoate (**1a**) (43.0 mg, 0.2 mmol) and NaBr (24.8 mg, 0.24 mmol). The reaction vessel was brough into a nitrogen-filled glove box, then charged with 4-CzIPN (3.2 mg, 2 mol%), $Ni(OAc)_2 \cdot 4H_2O$ (5.0 mg, 10 mol%), 4,4',4"-tri-tert-butyl-2,2':6'2"- terpyridine (12.2 mg, 15 mol%), Na_2CO_3 (22.0 mg, 0.2 mmol) and NMP (2.0 mL, 0.1 M). The reaction mixture was stirred for 3 minutes, then the reaction vessel was sealed with a screw cap and removed from the glovebox. The cap of the reaction vessel was further sealed with Parafilm, and then the reaction mixture was irradiated with blue LEDs at 40 °C for 48 hours. The reaction mixture was quenched with brine (5 mL) and extracted with ethyl acetate (5 x 3 mL). The combined organic extracts were dried ($MgSO_4$), concentrated under reduced pressure, purified by silica gel chromatography (0 to 3% MeOH in DCM) and then purified a second time by silica gel chromatography (EtOAc), affording the title compound (16.1 mg, 24%) as a white solid. In a second independent experiment, 20.1 mg (30%) of the title compound was obtained, giving an average of 27% yield. **M.p.** 192 °C. **IR (film, cm^{-1}) ν_{max}** = 3220, 2927, 1698, 1283. **M.p.**: 192 °C. **^{1}H NMR (500 MHz, CD_3OD)** δ 7.94 (d, *J* = 8.3, Hz, 2H), 7.33 (d, *J* = 8.3, Hz, 2H), 4.50 (ddd, *J* = 7.9, 5.0, 0.9 Hz, 1H), 4.30 (dd, *J* = 7.9, 4.5, 0.9 Hz, 1H), 3.24 – 3.19 (m, 1H), 2.94 (dd, *J* = 12.7, 5.0 Hz, 1H), 2.75 – 2.70 (m, 3H), 1.83 – 1.68 (m, 3H), 1.67 – 1.60 (m, 1H), 1.50 (q, *J* = 12.7, 7.5 Hz, 1H) ppm. **^{13}C NMR (126 MHz, CD_3OD)** δ 168.7, 166.1, 149.8, 130.6, 129.7, 128.9, 63.5, 61.6, 57.1, 52.5, 41.0, 36.7, 32.2, 29.9, 29.6 ppm. **HRMS** [ESI$^+$] *calcd.* for ($C_{17}H_{22}N_2NaO_3S$) $[M+Na]^+$: 357.1243, *found* 357.1232.

Methyl 1-cyclopropyl-6-fluoro-7-(4-(4-(4-(methoxycarbonyl)phenyl)butanoyl)piperazin-1-yl)-4-oxo-1,4-dihydroquinoline-3-carboxylate (3r)

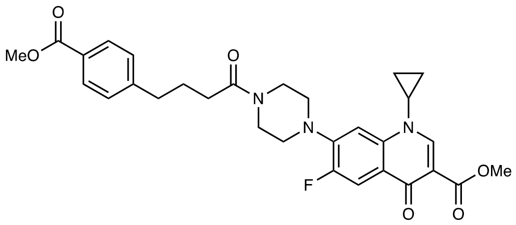

An oven-dried 8 mL screw-cap test tube containing a stirring bar was charged with methyl 1-cyclopropyl-6-fluoro-4-oxo-7-(4-(4-(4-oxo-2-phenyl-1,2,3,4-tetrahydroquinazolin-2-yl)butanoyl)piperazin-1-yl)-1,4-dihydroquinoline-3-carboxylate (**2q**) (98.1 mg, 0.15 mmol), methyl 4-bromobenzoate (**1a**) (28.0 mg, 0.13 mmol) and NaBr (16.1 mg, 0.15 mmol). The reaction vessel was brough into a nitrogen-filled glove box, then charged with 4-CzIPN (2.1 mg, 2 mol%), $Ni(OAc)_2{\cdot}4H_2O$ (3.2 mg, 10 mol%), 4,4',4"-tri-tert-butyl-2,2':6'2"- terpyridine (7.8 mg, 15 mol%), Na_2CO_3 (13.8 mg, 0.13 mmol) and NMP (1.2 mL, 0.1 M). The reaction mixture was stirred for 3 minutes, then the reaction vessel was sealed with a screw cap and removed from the glovebox. The cap of the reaction vessel was further sealed with Parafilm, and then the reaction mixture was irradiated with blue LEDs at 40 °C for 48 hours. The reaction mixture was quenched with brine (5 mL) and extracted with ethyl acetate (5 x 3 mL). The combined organic extracts were dried ($MgSO_4$), concentrated under reduced pressure and purified twice by silica gel chromatography (0 to 3% MeOH in DCM, then again using 0 to 20% acetone in DCM) , affording the title compound (24.4 mg, 22%) as a colorless film. In a second independent experiment, 30.3 mg (30%) of the title compound was obtained, giving an average of 26% yield. **IR (film, cm^{-1})** ν_{max} = 3488, 2951, 1718, 1618, 1434, 1247. **^{1}H NMR (400 MHz, $CDCl_3$)** δ 8.54 (s, 1H), 8.03 (d, *J* = 8.1 Hz, 13.0, 1H), 7.67 (d, *J* = 8.1 Hz, 8.1, 2H), 7.30 – 7.24 (m, 3H), 3.91 (s, 3H), 3.90 (s, 3H), 3.85 (br. t, *J* = 8.1 Hz, 4.7, 2H), 3.62 (br. t, *J* = 8.1 Hz, 4.7, 2H), 3.43 (tt, *J* = 7.2, 3.9 Hz, 4.7, 1H), 3.25 – 3.18 (m, 4H), 2.77 (t, *J* = 8.1 Hz, 7.4, 2H), 2.38 (t, *J* = 8.1 Hz, 7.4, 2H), 2.04 (p, *J* = 7.5 Hz, 7.4, 2H), 1.33 (p, *J* = 6.8 Hz, 7.4, 2H), 1.17 – 1.11 (m, 2H) ppm. **^{13}C NMR (126 MHz, $CDCl_3$)** δ 173.13, 171.15, 167.20, 166.48, 153.5 (d, *J* = 248.9 Hz), 148.61, 147.22, 144.2 (d, *J* = 10.7 Hz), 138.11, 129.91, 128.66, 128.21, 123.74 (d, *J* = 6.7 Hz), 113.77 (d, *J* = 22.9 Hz), 110.35, 105.22, 52.24, 52.16, 50.60, 49.74, 45.50, 41.42, 35.38, 34.66, 32.18, 26.29, 8.30. **^{19}F NMR (376**

MHz, $CDCl_3$) δ -124.0 (dd, *J* = 13.1, 7.0 Hz) ppm. **HRMS** [ESI^+] *calcd.* for ($C_{30}H_{32}FN_3NaO_6$) $[M+Na]^+$: 572.2167, *found* 572.2160.

4-(Phenoxymethyl)benzaldehyde (5a)

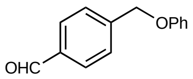

Following GP4, 4-bromobenzaldehyde (37.0 mg, 0.20 mmol) and 2-methyl-2-(phenoxymethyl)-2,3-dihydroquinazolin-4(1*H*)-one (64.3 mg, 0.24 mmol) were used, affording the title compound as a white solid (37.2 mg, 88% yield), by using hexane/EtOAc (40:1) as chromatography eluent. In a second independent experiment, 36.4 mg (86%) were obtained, giving an average of 87% yield. **M.p.**: 78 °C. **IR (neat, cm^{-1}):** 2917, 2852, 1692, 1580, 1486, 1466, 1375, 1230, 1166, 1014, 810, 755, 691, 508. **^{1}H NMR (400 MHz, $CDCl_3$)** δ 10.03 (s, 1H), 7.94 – 7.87 (m, 2H), 7.61 (d, *J* = 8.0 Hz, 2H), 7.34 – 7.27 (m, 2H), 7.02 – 6.95 (m, 3H), 5.16 (s, 2H) ppm. **^{13}C NMR (101 MHz, $CDCl_3$)** δ 192.0, 158.5, 144.3, 136.1, 130.2, 129.7, 127.6, 121.5, 115.0, 69.3 ppm. **HRMS** [ESI^+] *calcd.* for ($C_{15}H_{16}NaO_3$) $[M+CH_3OH+Na]^+$: 267.0997, *found*: 267.1005.

4-(Phenoxymethyl)benzonitrile (5b)

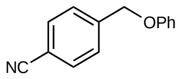

Following GP4, 4-bromobenzonitrile (36.4 mg, 0.20 mmol) and 2-methyl-2-(phenoxymethyl)-2,3-dihydroquinazolin-4(1*H*)-one (64.3 mg, 0.24 mmol) were used, affording the title compound as a white solid (37.8 mg, 90% yield), by using hexane/EtOAc (10:1) as chromatography eluent. In a second independent experiment, 36.5 mg (87%) were obtained, giving an average of 89% yield. **M.p.**: 62 °C. **^{1}H NMR (400 MHz, $CDCl_3$)** δ 7.71 – 7.65 (m, 2H), 7.58 – 7.52 (m, 2H), 7.35 – 7.27 (m, 2H), 7.03 – 6.93 (m, 3H), 5.13 (s, 2H) ppm. **^{13}C NMR (101 MHz, $CDCl_3$)** δ 158.3, 142.7, 132.5, 129.8, 127.7, 121.6, 118.8, 114.9, 111.8, 69.0 ppm. Spectral data was in agreement with the literature.[18]

(4-(Phenoxymethyl)phenyl)(phenyl)methanone (5c)

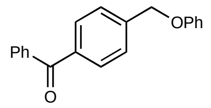

Following GP4, (4-bromophenyl)(phenyl)methanone (52.2 mg, 0.20 mmol) and 2-methyl-2-(phenoxymethyl)-2,3-dihydroquinazolin-4(1*H*)-one (64.3 mg, 0.24 mmol) were used, affording the title compound as a white solid (52.2 mg, 91% yield) by using hexane/EtOAc (40:1) as chromatography eluent. In a second independent experiment, 49.8 mg (86%) were obtained, giving an average of 89% yield. **M.p.**: 76 °C. **^{1}H NMR (400 MHz, $CDCl_3$)** δ 7.87 – 7.79 (m, 4H), 7.63 – 7.54 (m, 3H), 7.53 – 7.46 (m, 2H), 7.35 – 7.28 (m, 2H), 7.03 – 6.96 (m, 3H), 5.17 (s, 2H). ppm. **^{13}C NMR (101 MHz, $CDCl_3$)** δ 196.4, 158.6, 142.0, 137.7, 137.2, 132.6, 130.5, 130.2, 129.7, 128.4, 127.1, 121.4, 115.0, 69.4 ppm. Spectral data was in agreement with the literature.[19]

1-(Phenoxymethyl)-4-(trifluoromethyl)benzene (5d)

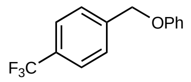

Following GP4, 1-bromo-4-(trifluoromethyl)benzene (45.0 mg, 0.20 mmol) and 2-methyl-2-(phenoxymethyl)-2,3-dihydroquinazolin-4(1*H*)-one (64.3 mg, 0.24 mmol) were used, affording the title compound as a white solid (35.1 mg, 70% yield), by using hexane/EtOAc (100:1) as chromatography eluent. In a second independent experiment, 37.8 mg (75%) were obtained, giving an average of 72% yield. **M.p.**: 77 °C. **^{1}H NMR (400 MHz, $CDCl_3$)** δ 7.65 (d, J = 8.1 Hz, 2H), 7.56 (d, J = 8.0 Hz, 2H), 7.36 – 7.28 (m, 2H), 7.03 – 6.94 (m, 3H), 5.14 (s, 2H) ppm. **^{13}C NMR (126 MHz, $CDCl_3$)** δ 158.5, 141.3, 130.2 (q, $J_{C,F}$ = 32 Hz), 129.7, 127.5, 125.7 (q, $J_{C,F}$ = 4 Hz), 124.3 (q, $J_{C,F}$ = 272 Hz), 121.5, 115.0, 69.2 ppm. **^{19}F NMR (376 MHz, $CDCl_3$)** δ -62.64 ppm. Spectral data was in agreement with the literature.[20]

1-(*tert*-Butyl)-4-(phenoxymethyl)benzene (5e)

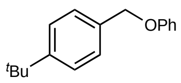

Following GP5, 1-bromo-4-(*tert*-butyl)benzene (42.6 mg, 0.20 mmol) and 2-methyl-2-(phenoxymethyl)-2,3-dihydroquinazolin-4(1*H*)-one (80.4 mg, 0.30 mmol) were used, affording the title compound as a white solid (26.7 mg, 56% yield), by using hexane/EtOAc (100:1) as chromatography eluent. In a second independent experiment, 24.3 mg (51%) were obtained, giving an average of 53% yield. **M.p.**: 100 °C. **^{1}H NMR (400 MHz, $CDCl_3$)** δ 7.45 – 7.36 (m, 4H), 7.34 – 7.27 (m, 2H), 7.03 – 6.93 (m, 3H), 5.04 (s, 2H), 1.34 (s, 9H) ppm. **^{13}C NMR (101 MHz, $CDCl_3$)** δ 159.1, 151.2, 134.2, 129.6, 127.6, 125.7, 121.0, 115.0, 69.9, 34.7, 31.5 ppm. Spectral data was in agreement with the literature.[21]

1,2-Dichloro-4-(phenoxymethyl)benzene (5f)

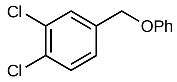

Following GP4, 4-bromo-1,2-dichlorobenzene (45.2 mg, 0.20 mmol) and 2-methyl-2-(phenoxymethyl)-2,3-dihydroquinazolin-4(1*H*)-one (64.3 mg, 0.24 mmol) were used, affording the title compound as a colourless liquid (15.5 mg, 31% yield), by using hexane/EtOAc (100:1) as chromatography eluent. In an independent experiment, 29.6 mg (58%) were obtained, giving an average of 45% yield. **IR (neat, cm^{-1}):** 3061, 1598, 1494, 1402, 1235, 1172, 1130, 1030, 880, 808, 751, 689. **^{1}H NMR (400 MHz, $CDCl_3$)** δ 7.57 (d, *J* = 2.0 Hz, 1H), 7.47 (d, *J* = 8.2 Hz, 1H), 7.37 – 7.25 (m, 3H), 7.05 – 6.95 (m, 3H), 5.04 (s, 2H) ppm. **^{13}C NMR (101 MHz, $CDCl_3$)** δ 158.4, 137.6, 132.9, 132.0, 130.7, 129.8, 129.4, 126.7, 121.5, 115.0, 68.6 ppm. Spectral data was in agreement with the literature. **HRMS** [ESI^+] *calcd.* for ($C_{15}H_{12}N_2NaO_2$) $[M+Na]^+$: 275.0796, *found*: 275.0800.

4-((4-(Phenoxymethyl)phenyl)sulfonyl)morpholine (5g)

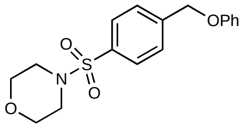

Following GP4, 4-((4-bromophenyl)sulfonyl)morpholine (61.2 mg, 0.20 mmol) and 2-methyl-2-(phenoxymethyl)-2,3-dihydroquinazolin-4(1*H*)-one (64.3 mg, 0.24 mmol) were used, affording the title compound as a white solid (47.2 mg, 71% yield), by using hexane/EtOAc (3:1) as chromatography eluent. In a second independent experiment, 48.3 mg (73%) were obtained, giving an average of 72% yield. **M.p.**: 178 °C. **IR (neat, cm^{-1}):** 2860, 1601, 1588, 1491, 1450, 1410, 1345, 1245, 1159, 1109, 941, 815, 733, 531. **^1H NMR (400 MHz, $CDCl_3$)** δ 7.81 – 7.74 (m, 2H), 7.63 (d, *J* = 8.6 Hz, 2H), 7.36 – 7.28 (m, 2H), 7.04 – 6.95 (m, 3H), 5.16 (s, 2H), 3.79 – 3.71 (m, 4H), 3.06 – 2.97 (m, 4H) ppm. **^{13}C NMR (101 MHz, $CDCl_3$)** δ 158.4, 142.9, 134.7, 129.8, 128.3, 127.8, 121.6, 114.9, 68.9, 66.2, 46.1 ppm. **HRMS** [ESI^+] *calcd.* for ($C_{17}H_{19}NNaO_4S$) $[M+Na]^+$: 356.0932, *found*: 356.0920.

(Cinnamyloxy)benzene (5h)

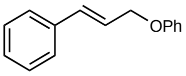

Following GP4, (*E*)-(2-bromovinyl)benzene (36.6 mg, 0.20 mmol) and 2-methyl-2-(phenoxymethyl)-2,3-dihydroquinazolin-4(1*H*)-one (64.3 mg, 0.24 mmol) were used, affording the title compound as a white solid (21.2 mg, 50% yield), by using hexane/EtOAc (100:1) as chromatography eluent. In a second independent experiment, 23.6 mg (56%) were obtained, giving an average of 53% yield. **M.p.**: 66 °C. **^1H NMR (400 MHz, $CDCl_3$)** δ 7.49 – 7.41 (m, 2H), 7.39 – 7.25 (m, 5H), 7.04 – 6.96 (m, 3H), 6.77 (d, *J* = 16.0 Hz, 1H), 6.45 (dt, *J* = 16.0, 5.8 Hz, 1H), 4.73 (dd, *J* = 5.8, 1.5 Hz, 2H) ppm. **^{13}C NMR (101 MHz, $CDCl_3$)** δ 158.8, 136.6, 133.1, 129.6, 128.7, 128.0, 126.7, 124.7, 121.1, 114.9, 68.7 ppm. Spectral data was in agreement with the literature.[22]

4-(Phenoxymethyl)pyridine (5i)

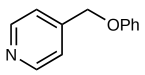

Following GP4, 4-bromopyridine (31.6 mg, 0.20 mmol) and 2-methyl-2-(phenoxymethyl)-2,3-dihydroquinazolin-4(1*H*)-one (64.3 mg, 0.24 mmol) were used, affording the title compound as a colourless oil (25.5 mg, 69% yield), by using hexane/EtOAc (3:1) as chromatography eluent. In a second independent experiment, 20.9 mg (56%) were obtained, giving an average of 63% yield. **IR (neat, cm^{-1}):** 3031, 1597, 1493, 1415, 1240, 1043, 798, 751, 690. **^{1}H NMR (400 MHz, $CDCl_3$)** δ 8.67 – 8.56 (m, 2H), 7.39 – 7.34 (m, 2H), 7.33 – 7.27 (m, 2H), 7.03 – 6.93 (m, 3H), 5.09 (s, 2H) ppm. **^{13}C NMR (101 MHz, $CDCl_3$)** δ 158.3, 150.1, 146.5, 129.8, 121.6, 121.6, 114.9, 68.2 ppm. **HRMS** [ESI$^+$] *calcd.* for ($C_{12}H_{12}NO$) $[M+H]^+$: 186.0919, *found*: 186.0913.

2-(Phenoxymethyl)-5-(trifluoromethyl)pyridine (5j)

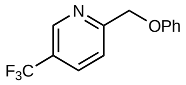

Following GP4, 2-bromo-5-(trifluoromethyl)pyridine (45.2 mg, 0.20 mmol) and 2-methyl-2-(phenoxymethyl)-2,3-dihydroquinazolin-4(1*H*)-one (64.3 mg, 0.24 mmol) were used, affording the title compound as a white solid (28.9 mg, 57% yield), by using hexane/EtOAc (20:1) as chromatography eluent. In a second independent experiment, 29.2 mg (58%) were obtained, giving an average of 57% yield. **M.p.**: 57 °C. **IR (neat, cm^{-1}):** 2927, 1604, 1586, 1491, 1448, 1394, 1324, 1244, 1211, 1078, 1014, 848, 755, 691, 568. **^{1}H NMR (400 MHz, $CDCl_3$)** δ 8.89 – 8.84 (m, 1H), 7.96 (dd, *J* = 8.2, 2.0 Hz, 1H), 7.70 (d, *J* = 8.2 Hz, 1H), 7.35 – 7.27 (m, 2H), 7.04 – 6.94 (m, 3H), 5.28 (s, 2H) ppm. **^{13}C NMR (126 MHz, $CDCl_3$)** δ 161.7 (d, $J_{C,F}$ = 1 Hz), 158.1, 146.3 (q, $J_{C,F}$ = 4 Hz), 134.1 (q, $J_{C,F}$ = 4 Hz), 129.8, 125.7 (q, $J_{C,F}$ = 33 Hz), 123.6 (q, $J_{C,F}$ = 273 Hz), 121.7, 120.9, 114.9, 70.1 ppm. **^{19}F NMR** (376 MHz, $CDCl_3$) δ -62.43. **HRMS** [ESI$^+$] *calcd.* for ($C_{13}H_{11}F_3NO$) $[M+H]^+$: 254.0793, *found*: 254.0786.

6-(Phenoxymethyl)quinoline (5k)

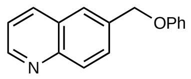

Following GP4, 6-bromoquinoline (41.6 mg, 0.20 mmol) and 2-methyl-2-(phenoxymethyl)-2,3-dihydroquinazolin-4(1*H*)-one (64.3 mg, 0.24 mmol) were used, affording the title compound as a white solid (26.0 mg, 55% yield), by using hexane/EtOAc (3:1) as chromatography eluent. In a second independent experiment, 21.3 mg (45%) were obtained, giving an average of 50% yield. **M.p.**: 81 °C. **IR (neat, cm^{-1}):** 2911, 1586, 1492, 1224, 768. **^{1}H NMR (400 MHz, $CDCl_3$)** δ 8.92 (dd, J = 4.2, 1.7 Hz, 1H), 8.19 – 8.11 (m, 2H), 7.91 – 7.86 (m, 1H), 7.78 (dd, J = 8.7, 1.9 Hz, 1H), 7.41 (dd, J = 8.3, 4.2 Hz, 1H), 7.35 – 7.28 (m, 2H), 7.07 – 6.95 (m, 3H), 5.26 (s, 2H) ppm. **^{13}C NMR (101 MHz, $CDCl_3$)** δ 158.7, 150.6, 148.1, 136.2, 135.7, 123.0, 129.7, 128.9, 128.3, 126.0, 121.5, 121.3, 115.0, 69.7 ppm. **HRMS** [ESI$^+$] *calcd.* for ($C_{16}H_{14}NO$) $[M+Na]^+$: 236.1070, *found*: 236.1068.

2-Methyl-6-(phenoxymethyl)quinoline (5l)

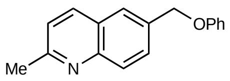

Following GP4, 6-bromo-2-methylquinoline (44.4 mg, 0.20 mmol) and 2-methyl-2-(phenoxymethyl)-2,3-dihydroquinazolin-4(1*H*)-one (64.3 mg, 0.24 mmol) were used, affording the title compound as a white solid (24.7 mg, 50% yield), by using hexane/EtOAc (3:1) as chromatography eluent. In a second independent experiment, 25.4 mg (51%) were obtained, giving an average of 50% yield. **M.p.**: 89 °C. **IR (neat, cm^{-1}):** 2913, 1595, 1489, 1461, 1370, 1230, 1175, 1028, 1009, 895, 817, 770, 699. **^{1}H NMR (400 MHz, $CDCl_3$)** δ 8.05 (dd, *J* = 8.4, 5.6 Hz, 2H), 7.83 (s, 1H), 7.74 (dd, *J* = 8.7, 1.9 Hz, 1H), 7.35 – 7.27 (m, 3H), 7.05 – 6.95 (m, 3H), 5.23 (s, 2H), 2.76 (s, 3H) ppm. **^{13}C NMR (101 MHz, $CDCl_3$)** δ 159.3, 158.8, 147.6, 136.4, 134.7, 129.7, 129.1, 129.0, 126.5, 125.9, 122.5, 121.3, 115.0, 69.8, 25.4 ppm. **HRMS** [ESI$^+$] *calcd.* for ($C_{17}H_{16}NO$) $[M+H]^+$: 250.1232, *found*: 250.1229.

2-(4-(Phenoxymethyl)phenyl)-1,3,4-oxadiazole (5m)

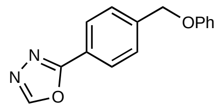

Following GP4, 2-(4-bromophenyl)-1,3,4-oxadiazole (45.0 mg, 0.20 mmol) and 2-methyl-2-(phenoxymethyl)-2,3-dihydroquinazolin-4(1*H*)-one (64.3 mg, 0.24 mmol) were used, affording the title compound as a white solid (36.0 mg, 71% yield), by using hexane/EtOAc (3:1) as chromatography eluent. In a second independent experiment, 33.0 mg (65%) were obtained, giving an average of 68% yield. **M.p.**: 134 °C. **IR (neat, cm^{-1}):** 3158, 2917, 1586, 1559, 1486, 1466, 1375, 1230, 1067, 1013, 871, 827, 755, 639. **^{1}H NMR (400 MHz, $CDCl_3$)** δ 8.47 (s, 1H), 8.14 – 8.07 (m, 2H), 7.60 (d, *J* = 8.6 Hz, 2H), 7.34 – 7.27 (m, 2H), 7.02 – 6.95 (m, 3H), 5.15 (s, 2H) ppm. **^{13}C NMR (101 MHz, $CDCl_3$)** δ 164.7, 158.5, 152.7, 141.6, 129.7, 127.9, 127.5, 123.1, 121.5, 115.0, 69.3 ppm. **HRMS** [ESI^+] *calcd.* for ($C_{15}H_{12}N_2NaO_2$) $[M+Na]^+$: 275.0796, *found*: 275.0800.

4-(Phenoxymethyl)dibenzo[*b*,*d*]furan (5n)

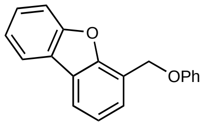

Following GP5, 4-bromodibenzo[*b*,*d*]furan (49.4 mg, 0.20 mmol) and 2-methyl-2-(phenoxymethyl)-2,3-dihydroquinazolin-4(1*H*)-one (80.4 mg, 0.30 mmol) were used, affording the title compound as a white solid (34.2 mg, 62% yield), by using hexane/EtOAc (50:1) as chromatography eluent. In an independent experiment, 36.0 mg (66%) were obtained, giving an average of 64% yield. **M.p.**: 105 °C. **IR (neat, cm^{-1}):** 3038, 1596, 1583, 1489, 1450, 1432, 1386, 1229, 1184, 1080, 1030, 1013, 844, 748, 691. **^{1}H NMR (400 MHz, $CDCl_3$)** δ 8.01 – 7.90 (m, 2H), 7.62 (d, *J* = 8.1 Hz, 2H), 7.52 – 7.46 (m, 1H), 7.42 – 7.28 (m, 4H), 7.12 – 7.06 (m, 2H), 7.03 – 6.95 (m, 1H), 5.49 (s, 2H) ppm. **^{13}C NMR (101 MHz, $CDCl_3$)** δ 158.9, 156.3, 153.9, 129.7, 127.4, 126.7, 124.4, 124.4, 123.1, 123.0, 121.3, 121.2, 120.9, 120.5, 115.1, 111.9, 64.7 ppm. **HRMS** [ESI^+] *calcd.* for ($C_{19}H_{14}NaO_2$) $[M+Na]^+$: 297.0891, *found*: 297.0887.

Methyl 4-methyl-3-(phenoxymethyl)benzoate (5o)

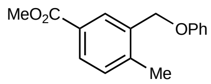

Following GP5, methyl 3-bromo-4-methylbenzoate (45.8 mg, 0.20 mmol) and 2-methyl-2-(phenoxymethyl)-2,3-dihydroquinazolin-4(1*H*)-one (80.4 mg, 0.30 mmol) were used, affording the title compound as a colourless liquid (24.7 mg, 48% yield), by using hexane/EtOAc (20:1) as chromatography eluent. In a second independent experiment, 18.1 mg (35%) were obtained, giving an average of 42% yield. **IR (neat, cm^{-1}):** 2950, 1716, 1598, 1495, 1435, 1294, 1211, 1101, 1007, 839, 752, 690. **^{1}H NMR (400 MHz, $CDCl_3$)** δ 8.12 (d, *J* = 1.7 Hz, 1H), 7.93 (dd, *J* = 7.9, 1.8 Hz, 1H), 7.36 – 7.27 (m, 3H), 7.04 – 6.96 (m, 3H), 5.06 (s, 2H), 3.91 (s, 3H), 2.44 (s, 3H) ppm. **^{13}C NMR (101 MHz, $CDCl_3$)** δ 167.1, 158.9, 142.6, 135.4, 130.7, 130.0, 129.7, 129.63, 128.3, 121.3, 115.0, 68.4, 52.2, 19.3 ppm. **HRMS** [ESI$^+$] *calcd.* for ($C_{16}H_{16}NaO_3$) $[M+Na]^+$: 279.0997, *found*: 279.0998.

(3a*R*,5*R*,6*R*,6a*R*)-5-((*R*)-2,2-dimethyl-1,3-dioxolan-4-yl)-2,2-dimethyltetrahydrofuro[2,3-*d*][1,3]dioxol-6-yl 4-(phenoxymethyl)benzoate (5p)

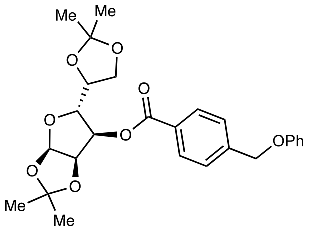

Following GP4, (3a*R*,5*R*,6*R*,6a*R*)-5-((*R*)-2,2-dimethyl-1,3-dioxolan-4-yl)-2,2-dimethyltetrahydrofuro[2,3-*d*][1,3]dioxol-6-yl 4-bromobenzoate (88.6 mg, 0.20 mmol) and 2-methyl-2-(phenoxymethyl)-2,3-dihydroquinazolin-4(1*H*)-one (64.3 mg, 0.24 mmol) were used, affording the title compound as an amorphous white solid (76.7 mg, 81% yield), by using hexane/EtOAc (5:1) as chromatography eluent. In a second independent experiment, 79.9 mg (85%) were obtained, giving an average of 83% yield. **IR (neat, cm^{-1}):** 3468, 2988, 1712, 1599, 1494, 1382, 1270, 1100, 1015, 841, 750, 690. **^{1}H NMR (400 MHz, $CDCl_3$)** δ 8.10 – 8.04 (m, 2H), 7.53 (d, *J* = 8.5 Hz, 2H), 7.34 – 7.27 (m, 2H), 7.01 – 6.94 (m, 3H), 5.90 (d, *J* = 3.8 Hz, 1H), 5.14 (s, 2H), 5.08 (dd, *J* = 8.1, 5.1 Hz, 1H), 4.97 (dd, *J* = 5.1, 3.9 Hz, 1H), 4.40 – 4.31 (m, 2H), 4.15 – 4.08 (m, 1H), 4.01 – 3.95 (m, 1H), 1.55 (s, 3H), 1.41 (s, 3H), 1.34 (s, 3H), 1.33 (s, 3H) ppm. **^{13}C NMR (101 MHz, $CDCl_3$)** δ 165.5, 158.6, 143.0, 130.3, 129.7, 129.1, 127.2, 121.4, 115.0, 113.3, 110.2, 104.5, 78.0, 78.0, 75.4, 73.4, 69.3, 65.9, 26.9, 26.8, 26.5, 25.2 ppm. **HRMS** [ESI^{+}] *calcd*. for ($C_{26}H_{30}NaO_8$) [M+Na]$^{+}$: 493.1838, *found*: 493.1830.

4-Dodecyltetrahydro-2*H*-pyran (8a)

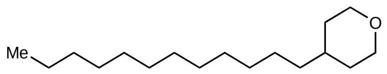

Following GP6, 1-bromododecane (49.8 mg, 0.20 mmol) and 2-methyl-2-(tetrahydro-2*H*-pyran-4-yl)-2,3-dihydroquinazolin-4(1*H*)-one (73.8 mg, 0.30 mmol) were used, affording the title compound as a colourless liquid (26.5 mg, 52% yield), by using hexane/EtOAc (100:1) as chromatography eluent. In a second independent experiment, 25.9 mg (51%) were obtained, giving an average of 51% yield. **IR (neat, cm^{-1}):** 2921, 2851, 1465, 1099. **^{1}H NMR (400 MHz, $CDCl_3$)** δ 3.98 – 3.89 (m, 2H), 3.36 (td, *J* = 11.9, 2.1 Hz, 2H), 1.64 – 1.54 (m, 2H), 1.51 – 1.38 (m, 1H), 1.35 – 1.17 (m, 24H), 0.92 – 0.83 (m, 3H) ppm. **^{13}C NMR (101 MHz, $CDCl_3$)** δ 68.4, 37.1, 35.1, 33.4, 32.1, 30.0, 29.8, 29.8, 29.8, 29.8, 29.8, 29.5, 26.5, 22.8, 14.3 ppm. **HRMS** [$APCI^+$] *calcd.* for ($C_{17}H_{35}O$) $[M+H]^+$: 255.2688, *found*: 255.2680.

4-(4-Phenylbutyl)tetrahydro-2*H*-pyran (8b)

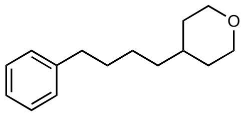

Following GP6, (4-bromobutyl)benzene (42.6 mg, 0.20 mmol) and 2-methyl-2-(tetrahydro-2*H*-pyran-4-yl)-2,3-dihydroquinazolin-4(1*H*)-one (73.8 mg, 0.30 mmol) were used, affording the title compound as a colourless liquid (22.3 mg, 51% yield), by using hexane/EtOAc (100:1) as chromatography eluent. In a second independent experiment, 21.7 mg (50%) were obtained, giving an average of 50% yield. **^{1}H NMR (400 MHz, $CDCl_3$)** δ 7.32 – 7.25 (m, 2H), 7.21 – 7.15 (m, 3H), 4.00 – 3.88 (m, 2H), 3.36 (td, *J* = 11.9, 2.1 Hz, 2H), 2.66 – 2.55 (m, 2H), 1.66 – 1.53 (m, 4H), 1.52 – 1.40 (m, 1H), 1.40 – 1.18 (m, 6H) ppm. **^{13}C NMR (101 MHz, $CDCl_3$)** δ 142.9, 128.5, 128.4, 125.8, 68.3, 36.9, 36.1, 35.1, 33.4, 31.8, 26.2 ppm. Spectral data was in agreement with the literature.[23]

4-(Undec-10-en-1-yl)tetrahydro-2*H*-pyran (8c)

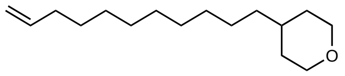

Following GP6, 11-bromoundec-1-ene (46.6 mg, 0.20 mmol) and 2-methyl-2-(tetrahydro-2*H*-pyran-4-yl)-2,3-dihydroquinazolin-4(1*H*)-one (73.8 mg, 0.30 mmol) were used, affording the title compound as a colourless liquid (26.5 mg, 56% yield), by using hexane/EtOAc (100:1) as eluent. In a second independent experiment, 25.4 mg (53%) were obtained, giving an average of 55% yield. **IR (neat, cm^{-1}):** 2922, 2852, 1100, 908. **^{1}H NMR (400 MHz, $CDCl_3$)** δ 5.81 (ddt, *J* = 16.9, 10.2, 6.7 Hz, 1H), 5.04 – 4.89 (m, 2H), 3.99 – 3.89 (m, 2H), 3.36 (td, *J* = 11.9, 2.1 Hz, 2H), 2.09 – 1.98 (m, 2H), 1.67 – 1.54 (m, 2H), 1.50 – 1.17 (m, 19H) ppm. **^{13}C NMR (101 MHz, $CDCl_3$)** δ 139.4, 114.2, 68.4, 37.1, 35.1, 34.0, 33.4, 30.0, 29.8, 29.7, 29.6, 29.3, 29.1, 26.5 ppm. **HRMS** [APCI$^+$] *calcd*. for ($C_{16}H_{31}O$) $[M+H]^+$: 239.2375, *found*: 239.2365.

7-Cyclohexylheptanenitrile (8d)

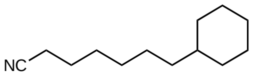

Following GP6, 7-bromoheptanenitrile (38.0 mg, 0.20 mmol) and 2-cyclohexyl-2-methyl-2,3-dihydroquinazolin-4(1*H*)-one (73.2 mg, 0.30 mmol) were used, affording the title compound as a colourless liquid (25.6 mg, 67% yield), by using hexane/EtOAc (10:1) as chromatography eluent. In a second independent experiment, 24.6 mg (65%) were obtained, giving an average of 66% yield. **^{1}H NMR (400 MHz, $CDCl_3$)** δ 2.33 (t, *J* = 7.1 Hz, 2H), 1.74 – 1.59 (m, 7H), 1.49 – 1.39 (m, 2H), 1.35 – 1.09 (m, 10H), 0.92 – 0.78 (m, 2H) ppm. **^{13}C NMR (101 MHz, $CDCl_3$)** δ 120.0, 37.7, 37.5, 33.6, 29.2, 28.8, 26.9, 26.7, 26.5, 25.5, 17.3 ppm. Spectral data was in agreement with the literature.[24]

3-Chlorophenyl 6-cyclohexylhexanoate (8e)

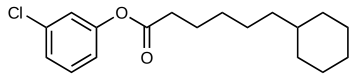

Following GP6, 3-chlorophenyl 6-bromohexanoate (61.0 mg, 0.20 mmol) and 2-cyclohexyl-2-methyl-2,3-dihydroquinazolin-4(1*H*)-one (73.2 mg, 0.30 mmol) were used, affording the title compound as a colourless liquid (40.8 mg, 66% yield), by using hexane/EtOAc (50:1) as chromatography eluent. In a second independent experiment, 45.8 mg (73%) were obtained, giving an average of 69% yield. **IR (neat, cm^{-1}):** 2920, 2850, 1763, 1590, 1473, 1199, 1114, 873, 778, 676. **^{1}H NMR (400 MHz, $CDCl_3$)** δ 7.30 (t, *J* = 8.1 Hz, 1H), 7.21 (ddd, *J* = 8.1, 2.0, 1.0 Hz, 1H), 7.12 (t, *J* = 2.0 Hz, 1H), 6.99 (ddd, *J* = 8.1, 2.2, 1.0 Hz, 1H), 2.54 (t, *J* = 7.5 Hz, 2H), 1.80 – 1.61 (m, 7H), 1.43 – 1.30 (m, 4H), 1.30 – 1.10 (m, 6H), 0.96 – 0.80 (m, 2H) ppm. **^{13}C NMR (101 MHz, $CDCl_3$)** δ 172.0, 151.4, 134.8, 130.2, 126.1, 122.4, 120.2, 37.8, 37.4, 34.5, 33.6, 29.5, 26.9, 26.6, 26.6, 25.0 ppm. **HRMS** [ESI$^+$] *calcd.* for ($C_{18}H_{25}ClNaO_2$) [M+Na]$^+$: 331.1441, *found*: 331.1439.

4-((6-Cyclohexylhexyl)oxy)benzaldehyde (8f)

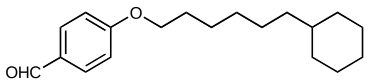

Following GP6, 4-((6-bromohexyl)oxy)benzaldehyde (57.0 mg, 0.20 mmol) and 2-cyclohexyl-2-methyl-2,3-dihydroquinazolin-4(1*H*)-one (73.2 mg, 0.30 mmol) were used, affording the title compound as a colourless liquid (37.8 mg, 66% yield), by using hexane/EtOAc (50:1) as chromatography eluent. In a second independent experiment, 39.4 mg (68%) were obtained, giving an average of 67% yield. **IR (neat, cm^{-1}):** 2920, 2850, 1691, 1599, 1577, 1509, 1448, 1311, 1253, 1157, 830, 617. **^{1}H NMR (400 MHz, $CDCl_3$)** δ 9.88 (s, 1H), 7.82 (d, *J* = 8.8 Hz, 2H), 6.99 (d, *J* = 8.7 Hz, 2H), 4.03 (t, *J* = 6.6 Hz, 2H), 1.87 – 1.76 (m, 2H), 1.74 – 1.57 (m, 5H), 1.52 – 1.41 (m, 2H), 1.39 –1.27 (m, 4H), 1.27 – 1.09 (m, 6H), 0.94 – 0.78 (m, 2H) ppm. **^{13}C NMR (126 MHz, $CDCl_3$)** δ 190.9, 164.4, 132.1, 129.9, 114.9, 68.5, 37.8, 37.5, 33.6, 29.8, 29.2, 26.9, 26.6, 26.1 ppm. (two peaks overlap in 26.9) **HRMS** [ESI$^+$] *calcd.* for ($C_{19}H_{28}NaO_2$) [M+Na]$^+$: 311.1987, *found*: 311.1986.

6-Cyclohexylhexyl furan-2-carboxylate (8g)

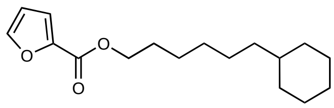

Following GP6, 6-bromohexyl furan-2-carboxylate (55.0 mg, 0.20 mmol) and 2-cyclohexyl-2-methyl-2,3-dihydroquinazolin-4(1*H*)-one (73.2 mg, 0.30 mmol) were used, affording the title compound as a colourless liquid (25.9 mg, 47% yield), by using hexane/EtOAc (50:1) as chromatography eluent. In a second independent experiment, 21.6 mg (39%) were obtained, giving an average of 43% yield. **IR (neat, cm^{-1}):** 2921, 2851, 1723, 1578, 1507, 1448, 1304, 1159, 1075, 974, 874, 761, 603. **^{1}H NMR (400 MHz, CDCl$_3$)** δ 8.00 (dd, *J* = 1.5, 0.8 Hz, 1H), 7.43 – 7.40 (m, 1H), 6.74 (dd, *J* = 1.9, 0.8 Hz, 1H), 4.23 (t, *J* = 6.7 Hz, 2H), 1.77 – 1.60 (m, 7H), 1.45 – 1.09 (m, 12H), 0.92 – 0.79 (m, 2H) ppm. **^{13}C NMR (101 MHz, CDCl$_3$)** δ 163.4, 147.7, 143.8, 119.8, 110.0, 64.8, 37.8, 37.6, 33.6, 29.7, 28.8, 26.9, 26.9, 26.6, 26.2 ppm. **HRMS** [ESI$^+$] *calcd.* for ($C_{17}H_{26}NaO_3$) [M+Na]$^+$: 301.1780, *found*: 301.1770.

6-Cyclohexylhexyl 1-methyl-1*H*-pyrrole-2-carboxylate (8h)

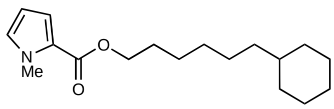

Following GP6, 6-bromohexyl 1-methyl-1*H*-pyrrole-2-carboxylate (57.6 mg, 0.20 mmol) and 2-cyclohexyl-2-methyl-2,3-dihydroquinazolin-4(1*H*)-one (73.2 mg, 0.30 mmol) were used, affording the title compound as a colourless liquid (36.0 mg, 62% yield) by using Hexane/EtOAc (50:1) as eluent. In an independent experiment, 38.7 mg (66%) were obtained, giving an average of 64% yield. **IR (neat, cm^{-1}):** 2920, 2850, 1701, 1531, 1414, 1319, 1243, 1107, 731. **^{1}H NMR (400 MHz, CDCl$_3$)** δ 6.94 (dd, *J* = 4.0, 1.8 Hz, 1H), 6.77 (t, *J* = 2.1 Hz, 1H), 6.11 (dd, *J* = 3.9, 2.5 Hz, 1H), 4.21 (t, *J* = 6.7 Hz, 2H), 3.92 (s, 3H), 1.77 – 1.59 (m, 7H), 1.46 – 1.36 (m, 2H), 1.36 – 1.25 (m, 5H), 1.24 – 1.13 (m, 5H), 0.93 – 0.79 (m, 2H) ppm. **^{13}C NMR (101 MHz, CDCl$_3$)** δ 161.6, 129.4, 122.9, 117.8, 107.9, 64.1, 37.8, 37.6, 36.9, 33.6, 29.7, 29.0, 26.9, 26.9, 26.6, 26.2 ppm. **HRMS** [ESI$^+$] *calcd.* for ($C_{18}H_{29}NNaO_2$) [M+Na]$^+$: 314.2096, *found*: 314.2094.

6-Cyclohexylhexyl 3-methylthiophene-2-carboxylate (8i)

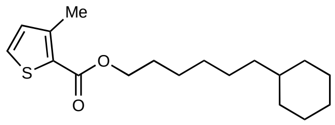

Following GP6, 6-bromohexyl 3-methylthiophene-2-carboxylate (61.0 mg, 0.20 mmol) and 2-cyclohexyl-2-methyl-2,3-dihydroquinazolin-4(1*H*)-one (73.2 mg, 0.30 mmol) were used, affording the title compound as a colourless liquid (30.8 mg, 50% yield), by using hexane/EtOAc (50:1) as chromatography eluent. In a second independent experiment, 37.1 mg (60%) were obtained, giving an average of 55% yield. **IR (neat, cm^{-1}):** 2920, 2850, 1707, 1541, 1415, 1255, 1101, 1071, 767, 721. **^{1}H NMR (400 MHz, $CDCl_3$)** δ 7.37 (d, *J* = 5.0 Hz, 1H), 6.90 (d, *J* = 5.0 Hz, 1H), 4.26 (t, *J* = 6.7 Hz, 2H), 2.55 (s, 3H), 1.78 – 1.60 (m, 7H), 1.47 – 1.36 (m, 2H), 1.36 – 1.09 (m, 10H), 0.93 – 0.78 (m, 2H) ppm. **^{13}C NMR (101 MHz, $CDCl_3$)** δ 163.1, 146.0, 131.8, 123.0, 127.3, 65.0, 37.8, 37.6, 33.6, 29.7, 28.9, 26.9, 26.9, 26.6, 26.2, 16.1 ppm. **HRMS** [ESI^{+}] *calcd.* for ($C_{18}H_{28}NaO_2S$) $[M+Na]^{+}$: 331.1708, *found*: 331.1701.

(8*R*,9*S*,13*S*,14*S*)-13-Methyl-3-(3-(tetrahydro-2*H*-pyran-4-yl)propoxy)-6,7,8,9,11,12,13,14,15,16-decahydro-17*H*-cyclopenta[a]phenanthren-17-one (8j)

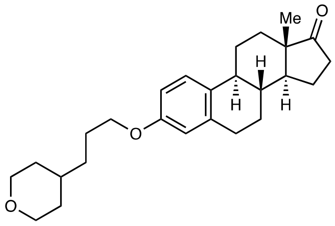

Following GP6 by running the reaction for 50 hours, (8*R*,9*S*,13*S*,14*S*)-3-(3-bromopropoxy)-13-methyl-6,7,8,9,11,12,13,14,15,16-decahydro-17*H*-cyclopenta[*a*]phenanthren-17-one (**6j**) (78.3 mg, 0.20 mmol) and 2-methyl-2-(tetrahydro-2*H*-pyran-4-yl)-2,3-dihydroquinazolin-4(1*H*)-one (**2d**) (73.9 mg, 0.30 mmol) were used. The resulting crude material was purified by silica gel chromatography (0 to 7.5% THF in hexane), affording the title compound as a white solid (40.3 mg, 51%). In a second independent experiment, 33.6 mg (42%) of the title compound was obtained, giving an average of 47% yield. **M.p.** 116 °C. **IR (film, cm^{-1}) ν_{max}** = 2925, 2853, 1737, 1499, 1234. **^{1}H NMR (500 MHz, $CDCl_3$)** δ 7.19 (d, *J* = 8.6 Hz, 1H), 6.70 (dd, *J* = 8.6, 2.7 Hz, 1H), 6.64 (d, *J* = 2.7 Hz, 1H), 4.02 – 3.88 (m, 4H), 3.37 (td, *J* = 11.7, 1.8 Hz, 2H), 2.95 – 2.83 (m, 2H), 2.57 – 2.45 (m, 1H), 2.30 – 2.21 (m, 1H), 2.18 – 1.92 (m, 4H), 1.83 – 1.74 (m, 2H), 1.66 – 1.22 (m, 14H), 0.91 (s, 3H) ppm. **^{13}C NMR (126 MHz, $CDCl_3$)** δ 157.2, 137.9, 132.1, 126.4, 114.7, 112.2, 68.22, 68.1, 50.5, 48.2, 44.1, 38.5, 36.0, 34.9, 33.4, 33.3, 31.7, 29.8, 26.7, 26.4, 26.1, 21.7, 14.0 ppm. **HRMS** [ESI$^+$] *calcd.* for ($C_{26}H_{36}NaO_3$) [M+Na]$^+$: 419.2557, *found* 419.2559.

(3*S*,8*S*,9*S*,10*R*,13*R*,14*S*,17*R*)-10,13-Dimethyl-17-((*R*)-6-methylheptan-2-yl)-2,3,4,7,8,9,10,11,12,13,14,15,16,17-tetradecahydro-1*H*-cyclopenta[a]phenanthren-3-yl 6-(tetrahydro-2H-pyran-4-yl)hexanoate (8k)

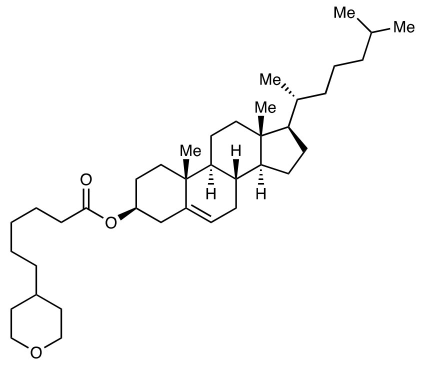

Following GP6 by running the reaction for 72 hours, (3*S*,8*S*,9*S*,10*R*,13*R*,14*S*,17*R*)-10,13-dimethyl-17-((*R*)-6-methylheptan-2-yl)-2,3,4,7,8,9,10,11,12,13,14,15,16,17-tetradecahydro-1*H*-cyclopenta[a]phenanthren-3-yl 6-bromohexanoate (**6k**) (112.7 mg, 0.20 mmol) and 2-methyl-2-(tetrahydro-2*H*-pyran-4-yl)-2,3-dihydroquinazolin-4(1*H*)-one (**2d**) (73.9 mg, 0.30 mmol) were used. The resulting crude material was purified by silica gel chromatography (0 to 4% Et_2O in hexane), affording the title compound as a white solid (53.0 mg, 47%) and recovered (3*S*,8*S*,9*S*,10*R*,13*R*,14*S*,17*R*)-10,13-dimethyl-17-((*R*)-6-methylheptan-2-yl)-2,3,4,7,8,9,10,11,12,13,14,15,16,17-tetradecahydro-1*H*-cyclopenta[a]phenanthren-3-yl 6-bromohexanoate (**6k**) (25.4 mg, 45 μmol). In a second independent experiment, 57.1 mg (50%) of the title compound along with recovered (3*S*,8*S*,9*S*,10*R*,13*R*,14*S*,17*R*)-10,13-dimethyl-17-((*R*)-6-methylheptan-2-yl)-2,3,4,7,8,9,10,11,12,13,14,15,16,17-tetradecahydro-1*H*-cyclopenta[a]phenanthren-3-yl 6-bromohexanoate (**6k**) (21.5 mg, 38 μmol) were obtained, giving an average of 49% yield (61% brsm). **M.p.** 94 °C. **IR (film, cm^{-1}) ν_{max}** = 2929, 2849, 1734, 1165. **^{1}H NMR (400 MHz, $CDCl_3$)** δ 5.37 (br. d, *J* = 4.9, Hz, 1H), 4.66 – 4.56 (m, 1H), 3.93 (dd, *J* = 11.5, 3.5 Hz, 2H), 3.35 (td, *J* = 11.5, 1.7 Hz, 2H), 2.31 (d, *J* = 8.1, 3.5 Hz, 2H), 2.27 (t, *J* = 7.5 Hz, 2H), 2.06 – 1.91 (m, 2H), 1.90 – 1.78 (m, 3H), 1.66 – 1.41 (m, 12H), 1.39 – 1.20 (m, 12H), 1.19 – 1.05 (m, 7H), 1.05 – 0.95 (m, 6H), 0.91 (d, *J* = 6.6 Hz, 3H), 0.87 (d, *J* = 1.8 Hz, 3H), 0.85 (d, *J* = 1.8 Hz, 3H), 0.68 (s, 3H) ppm. **^{13}C NMR (126 MHz, $CDCl_3$)** δ 173.2, 139.7, 122.6, 73.7, 68.2, 56.7, 56.1, 50.0, 42.3, 39.7, 39.5, 38.2, 37.0, 36.7, 36.6, 36.2, 35.8, 34.9, 34.7, 33.2, 31.9, 31.9, 29.2, 28.2, 28.0, 27.8, 26.0, 25.0, 24.3, 23.8, 22.8, 22.6, 21.0, 19.3, 18.7, 11.9 ppm. **HRMS** [ESI$^+$] *calcd.* for ($C_{38}H_{64}NaO_3$) $[M+Na]^+$: 591.4748, *found* 591.4734.

(5-Phenoxypentyl)benzene (8l)

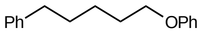

Following GP6, (4-bromobutyl)benzene (42.6 mg, 0.20 mmol) and 2-methyl-2-(phenoxymethyl)-2,3-dihydroquinazolin-4(1*H*)-one (80.4 mg, 0.30 mmol) were used, affording the title compound as a colourless liquid (24.0 mg, 50% yield), by using hexane/EtOAc (100:1) as chromatography eluent. In a second independent experiment, 23.0 mg (48%) were obtained, giving an average of 49% yield. **^{1}H NMR (400 MHz, $CDCl_3$)** δ 7.32 – 7.24 (m, 4H), 7.23 – 7.15 (m, 3H), 6.96 – 6.86 (m, 3H), 3.96 (t, *J* = 6.5 Hz, 2H), 2.70 – 2.61 (m, 2H), 1.87 – 1.78 (m, 2H), 1.76 – 1.65 (m, 2H), 1.58 – 1.47 (m, 2H) ppm. **^{13}C NMR (101 MHz, $CDCl_3$)** δ 159.2, 142.7, 129.6, 128.6, 128.4, 125.8, 120.6, 114.7, 67.9, 36.0, 31.4, 29.3, 25.9 ppm. Spectral data was in agreement with the literature.[25]

1-Morpholino-8-phenyloctan-1-one (8m)

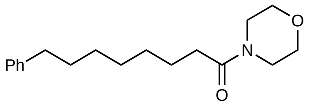

Following GP6, (4-bromobutyl)benzene (42.6 mg, 0.20 mmol) and 2-(4-morpholino-4-oxobutyl)-2-phenyl-2,3-dihydroquinazolin-4(1*H*)-one (113.7 mg, 0.30 mmol) were used, affording the title compound as a colourless liquid (30.3 mg, 52% yield), by using hexane/EtOAc (3:1) as chromatography eluent. In a second independent experiment, 32.1 mg (56%) were obtained, giving an average of 54% yield. **IR (neat, cm^{-1}):** 2924, 2853, 1643, 1427, 1229, 1114, 1036, 699. **^{1}H NMR (400 MHz, $CDCl_3$)** δ 7.32 – 7.26 (m, 2H), 7.22 – 7.16 (m, 3H), 3.73 – 3.56 (m, 6H), 3.48 (s, 2H), 2.67 – 2.58 (m, 2H), 2.37 – 2.28 (m, 2H), 1.70 – 1.57 (m, 4H), 1.44 – 1.32 (m, 6H) ppm. **^{13}C NMR (101 MHz, $CDCl_3$)** δ 172.0, 142.9, 128.5, 128.4, 125.7, 67.1, 66.8, 46.2, 42.0, 36.1, 33.2, 31.6, 29.5, 29.4, 29.3, 25.4 ppm. **HRMS** [ESI$^+$] *calcd.* for ($C_{18}H_{27}NNaO_2$) $[M+Na]^+$: 312.1939, *found*: 312.1942.

Ethyl 5-(2,5-dimethylphenoxy)-2,2-dimethylpentanoate (8n)

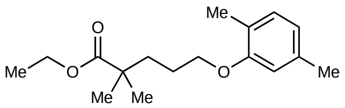

Following GP6, ethyl 4-bromo-2,2-dimethylbutanoate (44.6 mg, 0.20 mmol) and 2-((2,5-dimethylphenoxy)methyl)-2-methyl-2,3-dihydroquinazolin-4(1*H*)-one (88.8 mg, 0.30 mmol) were used, affording the title compound as a colourless liquid (25.2 mg, 45% yield), by using hexane/EtOAc (50:1) as chromatography eluent. In a second independent experiment, 21.1 mg (38%) were obtained, giving an average of 42% yield. **IR (neat, cm^{-1}):** 2978, 2930, 1724, 1266, 1140, 1129. **^{1}H NMR (400 MHz, $CDCl_3$)** δ 7.01 (d, J = 7.5 Hz, 1H), 6.66 (d, J = 7.5 Hz, 1H), 6.61 (s, 1H), 4.13 (q, J = 7.1 Hz, 2H), 3.92 (t, J = 5.7 Hz, 2H), 2.31 (s, 3H), 2.18 (s, 3H), 1.79 – 1.69 (m, 4H), 1.25 (t, J = 7.1 Hz, 3H), 1.22 (s, 6H) ppm. **^{13}C NMR (101 MHz, $CDCl_3$)** δ 178.0, 157.1, 136.6, 130.4, 123.7, 120.8, 112.1, 68.1, 60.4, 42.1, 37.2, 25.3, 25.3, 21.5, 15.9, 14.4 ppm. **HRMS** [ESI$^+$] *calcd.* for ($C_{17}H_{26}NaO_3$) $[M+Na]^+$: 301.1774, *found*: 301.1773.

(Cyclohexylmethoxy)benzene (8o)

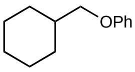

Following GP6, bromocyclohexane (32.6 mg, 0.20 mmol) and 2-methyl-2-(phenoxymethyl)-2,3-dihydroquinazolin-4(1*H*)-one (80.4 mg, 0.30 mmol) were used, affording the title compound as a colourless liquid (22.5 mg, 59% yield), by using hexane/EtOAc (100:1) as chromatography eluent. In a second independent experiment, 19.0 mg (50%) were obtained, giving an average of 55% yield. **^{1}H NMR (400 MHz, $CDCl_3$)** δ 7.33 – 7.23 (m, 2H), 6.97 – 6.86 (m, 3H), 3.76 (d, *J* = 6.4 Hz, 2H), 1.93 – 1.84 (m, 2H), 1.84 – 1.67 (m, 4H), 1.38 – 1.15 (m, 3H), 1.12 – 1.00 (m, 2H) ppm. **^{13}C NMR (101 MHz, $CDCl_3$)** δ 159.5, 129.5, 120.5, 114.7, 73.6, 37.9, 30.1, 26.7, 26.0 ppm. Spectral data was in agreement with the literature.[26]

3-(4,5-Diphenyloxazol-2-yl)-1-(4-(phenoxymethyl)piperidin-1-yl)propan-1-one (8p)

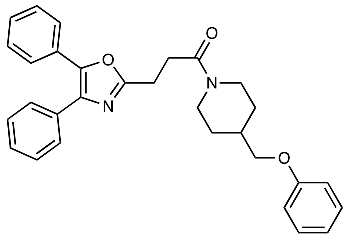

Following GP6 by running the reaction for 48 hours, 1-(4-bromopiperidin-1-yl)-3-(4,5-diphenyloxazol-2-yl)propan-1-one (**6m**) (87.9 mg, 0.20 mmol) and 2-methyl-2-(phenoxymethyl)-2,3-dihydroquinazolin-4(1*H*)-one (**2l**) (80.5 mg, 0.30 mmol) were used. The resulting crude material was purified by silica gel chromatography (0 to 10% Et_2O in DCM) and then a second time by silica gel chromatography (0 to 40% EtOAc in *n*-pentane), affording the title compound as a colourless oil (37.3 mg, 40%). In a second independent experiment, 46.8 mg (50%) of the title compound was obtained, giving an average of 45% yield. **IR (film, cm^{-1}) ν_{max}** = 2921, 1643, 1599, 1497, 1445, 1244. **^{1}H NMR (500 MHz, $CDCl_3$)** δ 7.66 – 7.62 (m, 2 H), 7.59 – 7.55 (m, 2 H), 7.39 – 7.55 (m, 6 H), 7.30 – 7.26 (m, 2 H), 6.94 (tt, *J* = 7.4, 1.0 Hz, 1H), 6.87 (dd, *J* = 8.7, 1.1, 2H), 4.70 (d, *J* = 13.5 Hz, 1H), 4.01 (d, *J* = 13.5 Hz, 1H), 3.84 – 3.73 (m, 2H), 3.29 – 3.21 (m, 2H), 3.10 (td, *J* = 12.9, 2.3, 1H), 2.99 (q, *J* = 7.3, 2H), 2.65 (td, *J* = 12.9, 2.3, 1H), 2.11 – 2.00 (m, 1H), 1.94 (d, *J* = 12.9, 1H), 1.87 (d, *J* = 12.9, 1H), 1.38 – 1.23 (m, 2H) ppm. **^{13}C NMR (126 MHz, $CDCl_3$)** δ 169.4, 163.3, 159.0, 145.5, 134.7, 132.1, 129.6, 128.9, 128.8, 128.8, 128.7, 128.4, 128.2, 126.6, 120.9, 114.6, 72.0, 45.6, 42.0, 36.5, 30.2, 29.7, 28.8, 24.0 ppm. **HRMS** [ESI$^+$] *calcd.* for ($C_{30}H_{30}N_2NaO_3$) [M+Na]$^+$: 489.2149, *found* 489.2140.

5.1 Scope Limitations

The following dihydroquinazolinone radical precursors failed to give satisfactory levels of cross-coupling product (>25%):

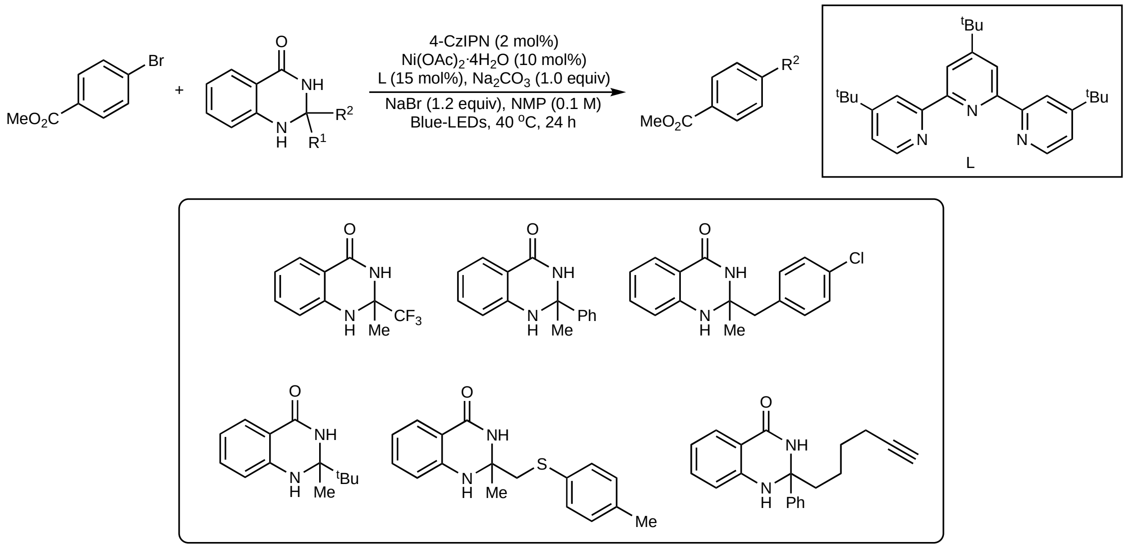

Supplementary Figure 5. Unsuccessful examples of radical precursors.

An adaptation of Molander and co-worker´s conditions[27] for the generation and capture of tertiary alkyl radicals under nickel catalysis was also attempted:

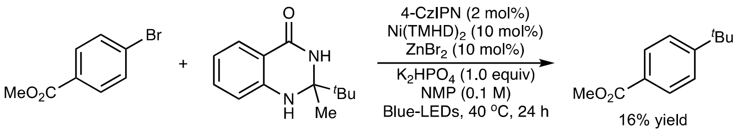

The following C(sp^2)-X substrates failed to give satisfactory levels of cross-coupling product (>25%):

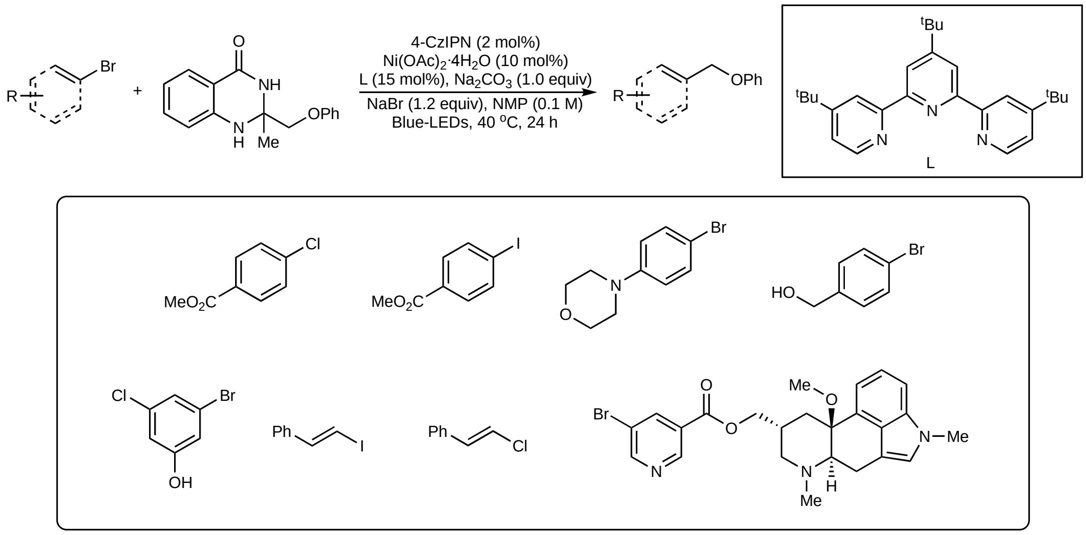

Supplementary Figure 6. Unsuccessful examples of aryl halides and vinyl halides.

The following alkyl-Br failed to give satisfactory levels of cross-coupling (>25%):

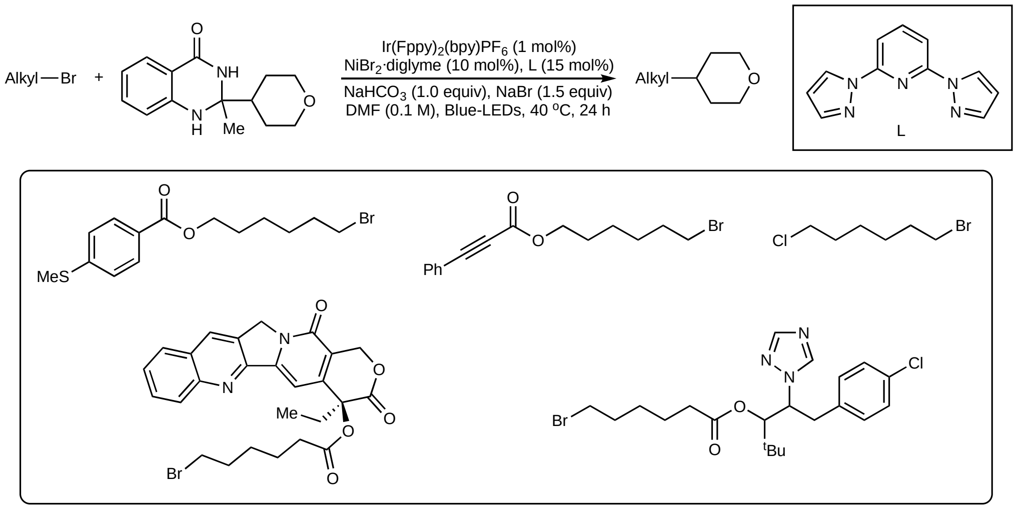

Supplementary Figure 7. Unsuccessful examples of alkyl bromides.

6. Telescoping the formation of Quinazolinones en route to *sp*^{3} Architectures

Methyl 4-cyclohexylbenzoate (3a)

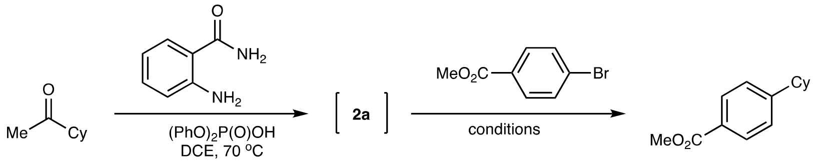

3a

An oven-dried 8 mL screw-cap test tube was charged with a stirring bar, 2-aminobenzamide (81.6 mg, 0.6 mmol), methyl cyclohexyl ketone (79.4 mg, 0.63 mmol), diphenyl phosphate (15.0 mg, 0.06 mmol) and DCE (1.2 mL). The reaction mixture was stirred at 70 °C for 24 hours. The solvent was removed under reduced pressure, then 4-CzIPN (3.2 mg, 0.004 mmol), $NiCl_2$·DME (4.4 mg, 0.02 mmol), 4,4',4''-tri-tert-butyl-2,2':6'2''-terpyridine (12.1 mg, 0.03 mmol), NaBr (24.7 mg, 0.24 mmol) and methyl 4-bromobenzoate (43.0 mg, 1.0 equiv, 0.2 mmol) were added. The test tube was taken into a nitrogen-filled glovebox where Na_2CO_3 (21.2 mg, 0.2 mmol) and NMP (2 mL) were added to the reaction vessel. The reaction vessel was sealed with a screw cap, the reaction mixture was stirred for 1 min. at rt, and then the reaction vessel was removed from the glovebox. Parafilm was used to ensure the tightness of the reaction system. The reaction mixture was stirred at 40 °C under blue LEDs irradiation for 24 hours. The reaction mixture was quenched with brine (10 mL) and extracted with ethyl acetate (3 x 5 mL). The combined organic extracts were dried (Na_2SO_4) and concentrated under reduced pressure yielding crude material. The crude material was purified by silica gel chromatography (hexane/EtOAc =100:1), obtaining **3a** (38.8 mg, 89%) as a colourless oil. **^{1}H NMR** (400 MHz, Chloroform-*d*) δ 7.91 – 7.85 (m, 2H), 7.23 – 7.16 (m, 2H), 3.82 (s, 3H), 2.54 – 2.42 (m, 1H), 1.88 – 1.73 (m, 4H), 1.72 – 1.64 (m, 1H), 1.43 – 1.25 (m, 4H), 1.25 – 1.12 (m, 1H) ppm. **^{13}C NMR** (101 MHz, $CDCl_3$) δ 167.3, 153.6, 129.8, 127.9, 127.0, 52.1, 44.8, 34.3, 26.9, 26.2 ppm.

Methyl 4-(phenoxymethyl)benzoate (3l)

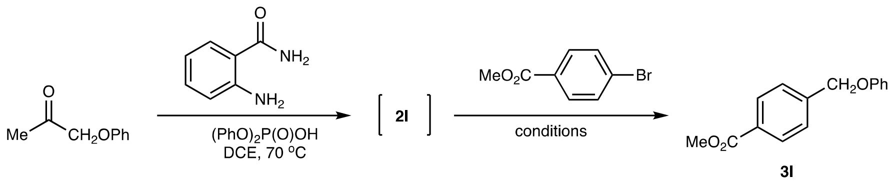

An oven-dried 8 mL screw-cap test tube was charged with a stirrer bar, 2-aminobenzamide (81.6 mg, 0.6 mmol), 1-phenoxypropan-2-one (94.5 mg, 0.63 mmol), diphenyl phosphate (15.0 mg, 0.06 mmol) and DCE (1.2 mL). The reaction mixture was stirred at 70 °C for 24 hours. The solvent was removed under reduced pressure, then 4-CzIPN (3.2 mg, 0.004 mmol), $NiCl_2$·DME (4.4 mg, 0.02 mmol), 4,4',4"-tri-tert-butyl-2,2':6'2"-terpyridine (12.1 mg, 0.03 mmol), NaBr (24.7 mg, 0.24 mmol) and methyl 4-bromobenzoate (43.0 mg, 1.0 equiv, 0.2 mmol) were added. The test tube was taken into a nitrogen-filled glovebox where Na_2CO_3 (21.2 mg, 0.2 mmol) and NMP (2 mL) were added to the reaction vessel. The reaction vessel was sealed with a screw cap, the reaction mixture was stirred for 1 min. at rt, and then the reaction vessel was removed from the glovebox. Parafilm was used to ensure the tightness of the reaction system. The reaction mixture was stirred at 40 °C under blue LED irradiation for 24 hours. The reaction mixture was quenched with brine (10 mL) and extracted with ethyl acetate (3 x 5 mL). The combined organic extracts were dried (Na_2SO_4) and concentrated under reduced pressure yielding crude material. The crude material was purified by silica gel chromatography (hexane/EtOAc =50:1), obtaining **3l** (35.6 mg, 55%) as a white solid. **^{1}H NMR** (400 MHz, Chloroform-*d*) δ 8.07 – 8.03 (m, 2H), 7.53 – 7.47 (m, 2H), 7.33 – 7.26 (m, 2H), 7.00 – 6.93 (m, 3H), 5.12 (s, 2H), 3.91 (s, 3H) ppm. ^{13}C NMR (101 MHz, $CDCl_3$) δ 167.0, 158.6, 142.5, 130.0, 129.8, 129.7, 127.1, 121.4, 115.0, 69.4, 52.2 ppm.

7. Mechanistic Studies

7.1 Experiments with a well-defined nickel complex

Synthesis of Nickel Complex · toluene (Ni-I)

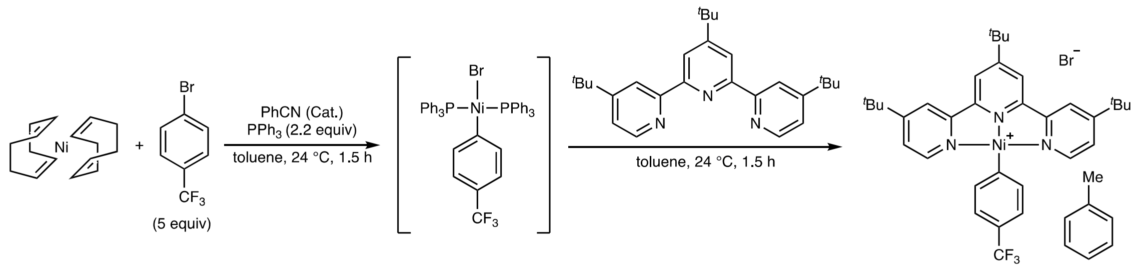

Nickel Complex • toluene (Ni-I)

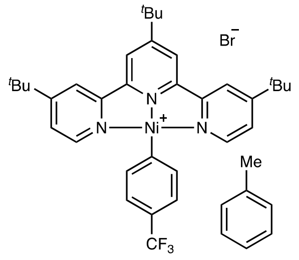

A 27 mL glass vial equipped with a stirrer bar was taken into a nitrogen-filled glovebox, then charged with Ni(COD)$_2$ (128 mg, 0.5 mmol), triphenylphosphine (289 mg, 1.1 mmol) and toluene (3 mL, anhydrous). The reaction mixture was stirred for 1.5 hours at 24 °C, then 1-bromo-4-(trifluoromethyl)benzene (0.35 mL, 2.5 mmol) and benzonitrile (2 drops) were added. Reaction mixture was stirred for 5 minutes at 24 °C, after which the solution produces a thick yellow precipitate. Extra toluene (2 mL, anhydrous) was added, and the reaction mixture was stirred for 10 minutes at 24 °C. The reaction vessel was removed from the glovebox and charged with hexane (20 mL) forming more yellow precipitate. The yellow suspension was filtered yielding a yellow solid, which was sequentially washed with hexane (20 mL) and ice-cold methanol (2 x 4 mL) affording a yellow solid (231 mg) that was used in the next step without further purification. A 27 mL glass vial equipped with a stirrer bar was charged with the yellow solid (220 mg) obtained from the previous step, taken into a nitrogen-filled glovebox, and charged with 4,4′,4″-tri-*tert*-butyl-2,2′:6′,2″-terpyridine (120 mg, 0.30 mmol) and toluene (5 mL, anhydrous). The reaction mixture was stirred for 1.5 hours at 24 °C. The reaction vessel was removed from the glovebox and charged with hexane (20 mL) forming a yellow precipitate. The yellow suspension was filtered yielding a yellow solid, which was

washed with hexane (20 mL) affording the desired complex (168 mg, 43%) as a yellow solid. Crystals for analysis by x-ray diffraction were prepared by layering diethyl ether upon a saturated solution of **Ni-I** in DCM. **IR (neat, cm^{-1}) ν_{max}** = 2962, 1614, 1582, 1317, 1154, 1115, 1073. **^{1}H NMR (400 MHz, CD_3CN)** δ 8.28 (s, 2H), 8.25 (d, *J* = 2.0 Hz, 2H), 8.03 (d, *J* = 8.0 Hz, 2H), 7.47 (d, *J* = 8.0 Hz, 2H), 7.37 (dd, *J* = 6.1, 2.0 Hz, 2H), 7.28 (d, *J* = 6.1 Hz, 2H), 7.30 – 7.20 (m, 5H, toluene), 2.33 (s, 3H, toluene), 1.53 (s, 9H), 1.38 (s, 18H) ppm. **^{1}H NMR (500 MHz, DMSO-d_6)** δ 8.63 (br. s, 2H), 8.62 (br. s, 2H), 8.06 (br. d, *J* = 6.1 Hz, 2H), 7.59 (br. s, 2H), 7.46 (br. d, *J* = 8.0 Hz, 2H), 7.31 – 7.07 (m, 2H + toluene 5H), 2.30 (s, 3H, toluene), 1.52 (s, 9H), 1.35 (s, 18H) ppm. **^{13}C NMR (126 MHz, DMSO-d_6)** δ 166.3, 155.6, 153.3, 151.9, 137.8 (Tol), 137.0, 129.3 (Tol), 128.6 (Tol), 125.8 (Tol), 125.6, 125.5 (q, *J* = 271.4 Hz, CF_3), 125.4, 125.3, 125.1, 122.8, 122.8, 121.6, 37.3, 36.3, 30.8, 30.2, 21.5 (Tol) ppm. **^{19}F NMR (376 MHz, DMSO-d_6)** δ -60.4.

Catalytic Competency of Ni-I

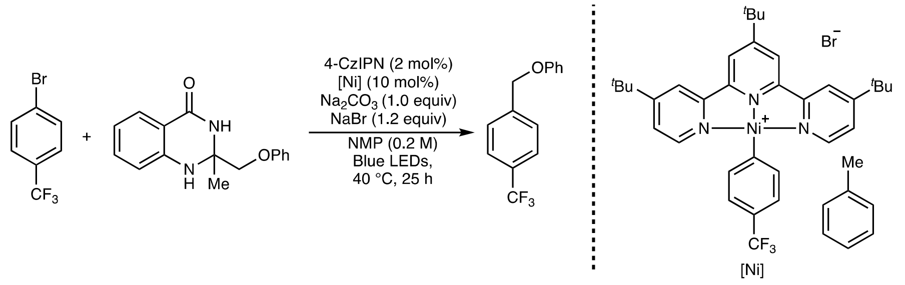

An oven-dried 8 mL screw-cap test tube containing a stirring bar was charged with sodium bromide (24.7 mg, 0.24 mmol), 4-CzIPN (3.2 mg, 2 mol%) and 2-methyl-2-(phenoxymethyl)-2,3-dihydroquinazolin-4(1*H*)-one (64.3 mg, 0.24 mmol). The reaction vessel was taken into a nitrogen-filled glove box, then sequentially charged with nickel complex (15.5 mg, 10 mol%), sodium carbonate (21.2 mg, 0.2 mmol), NMP (1 mL) and 1-bromo-4-(trifluoromethyl)benzene (28 μL, 45.0 mg, 0.2 mmol). The reaction mixture was stirred for 3 minutes, then the reaction vessel was sealed, removed from the glovebox and the screw cap was further sealed with parafilm. The reaction mixture was stirred while exposed to blue LED irradiation for 25 hours at 40 °C. The reaction was quenched with brine (5 mL) and extracted with EtOAc (5 x 3 mL). The combined organic extracts were dried ($MgSO_4$) and concentrated under reduced pressure. The received crude residue was analyzed by ^{19}F NMR using 1-fluoro-3-nitrobenzene (22.0 μL, 0.206 mmol) as an internal standard, showing had 1-(phenoxymethyl)-4-(trifluoromethyl)benzene formed (60%).

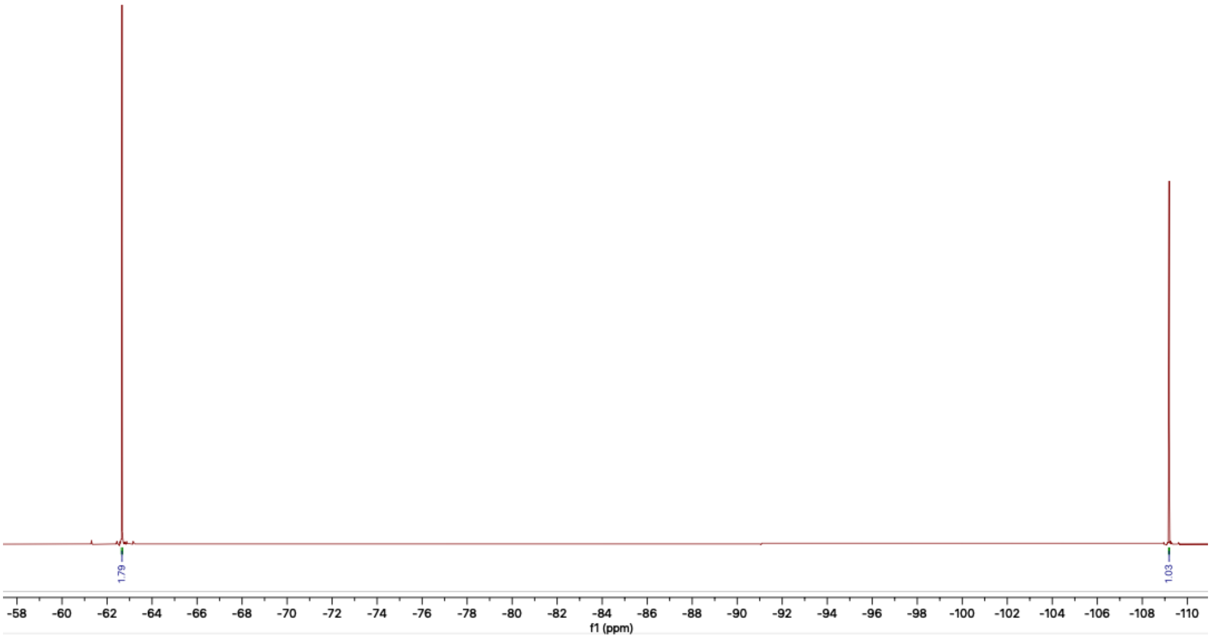

Supplementary Figure 8. Crude ^{19}F NMR spectrum of catalytic experiment of **Ni-I**.

Stoichiometric experiments with Ni-I

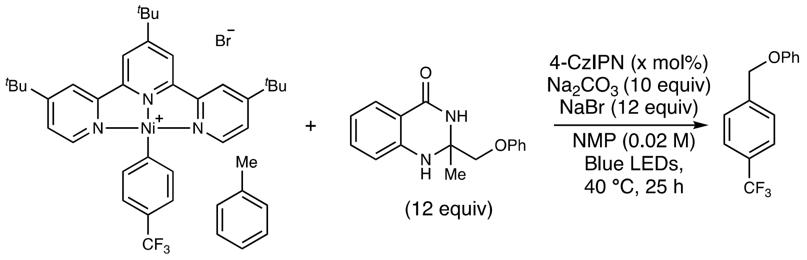

An oven-dried 8 mL screw-cap test tube containing a stirring bar was charged with sodium bromide (61.7 mg, 0.6 mmol). The reaction vessel was taken into a nitrogen-filled glove box, then sequentially charged with 4-CzIPN (8 mg, 20 mol%), nickel complex (38.8 mg, 0.05 mmol), sodium carbonate (53.0 mg, 0.5 mmol), 2-methyl-2-(phenoxymethyl)-2,3-dihydroquinazolin-4(1*H*)-one (161 mg, 0.6 mmol) and NMP (2.5 mL). The reaction mixture was stirred for 3 minutes, then the reaction vessel was sealed, removed from the glovebox and the screw cap was further sealed with parafilm. The reaction mixture was stirred while exposed to blue LED irradiation for 25 hours at 40 °C. The reaction was quenched with brine (5 mL) and extracted with EtOAc (5 x 3 mL). The combined organic extracts were dried ($MgSO_4$) and concentrated under reduced pressure. The received crude residue was analyzed by ^{19}F NMR using 1-fluoro-3-nitrobenzene (6.0 μL, 0.056 mmol) as an internal standard, showing had 1-(phenoxymethyl)-4-(trifluoromethyl)benzene formed (24%).

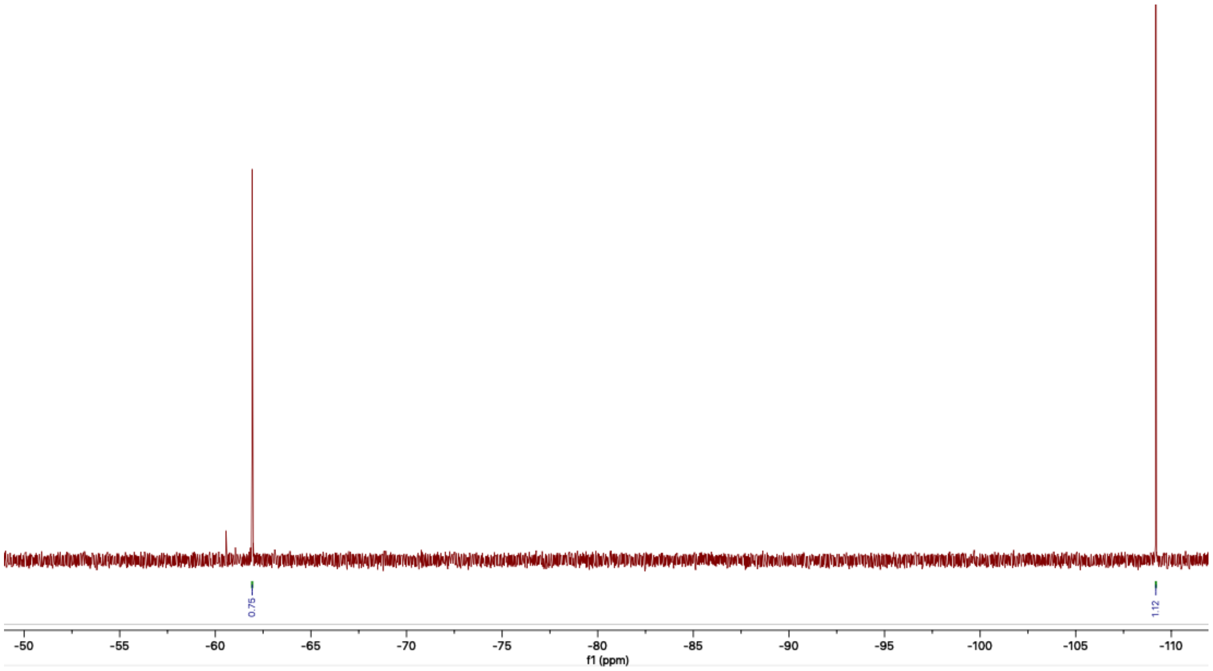

Supplementary Figure 9. Crude ^{19}F NMR spectrum of stoichiometric experiments of **Ni-I**.

The reaction was repeated in the absence of 4-CzIPN, the resulting crude was analyzed by ^{19}F NMR using 1-fluoro-3-nitrobenzene (6.0 μL, 0.056 mmol) as an internal standard, showing had 1-(phenoxymethyl)-4-(trifluoromethyl)benzene formed (2%).

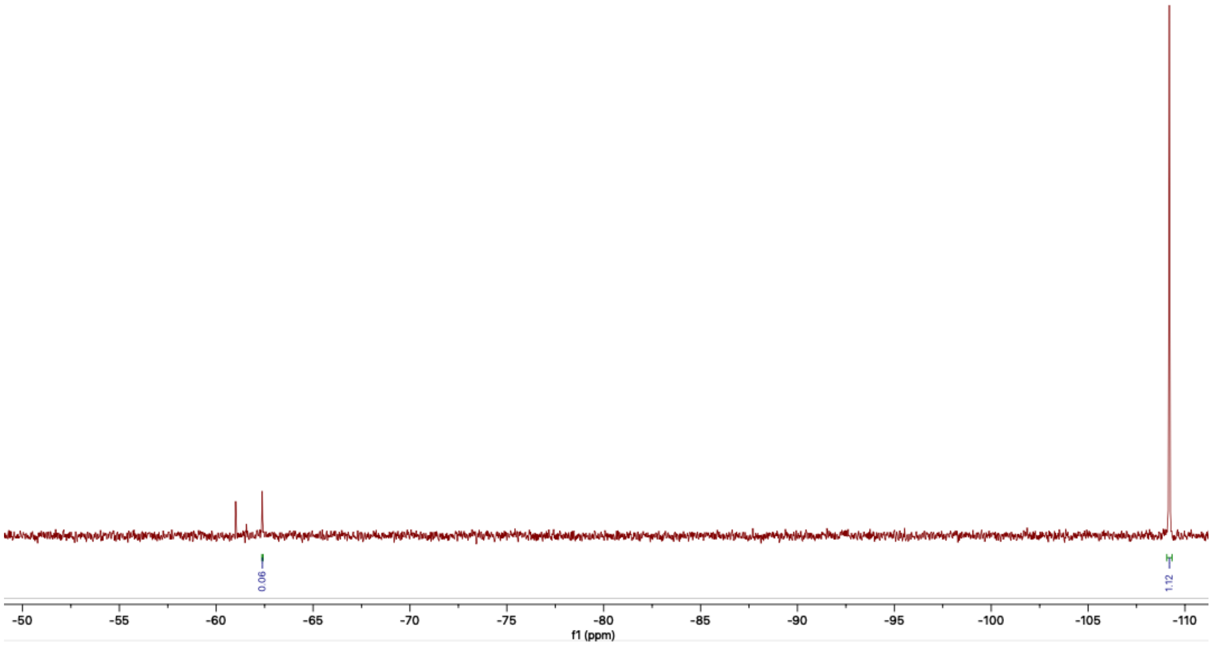

Supplementary Figure 10. Crude ^{19}F NMR spectrum of stoichiometric experiments in the absence of 4-CzIPN.

7.2 UV-Vis. Spectroscopy

Samples for UV-Vis. analysis were prepared in a 4 mL quartz cuvette (path length: l = 1.0 cm) equipped with a rubber septum screwcap under an atmosphere of nitrogen. A 4.4 x 10^{-5} M solution of nickel complex **Ni-I** and a 7.0 x 10^{-6} M solution of 4-CzIPN were prepared in a nitrogen-filled glovebox from dry and degassed NMP.

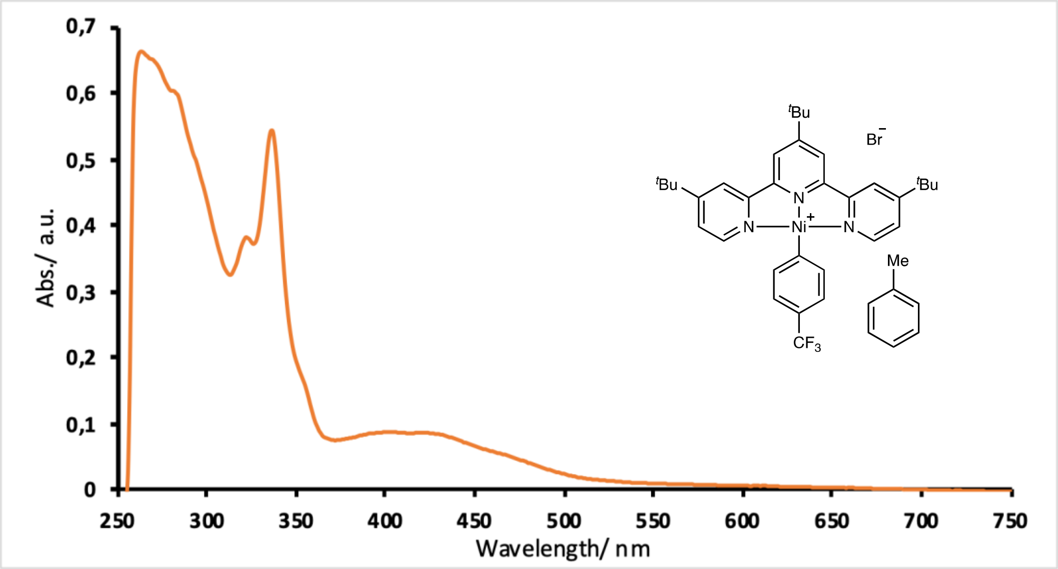

Supplementary Figure 11. UV-Vis. spectrum of a 4.4 x 10^{-5} M solution of **Ni-I** in NMP.

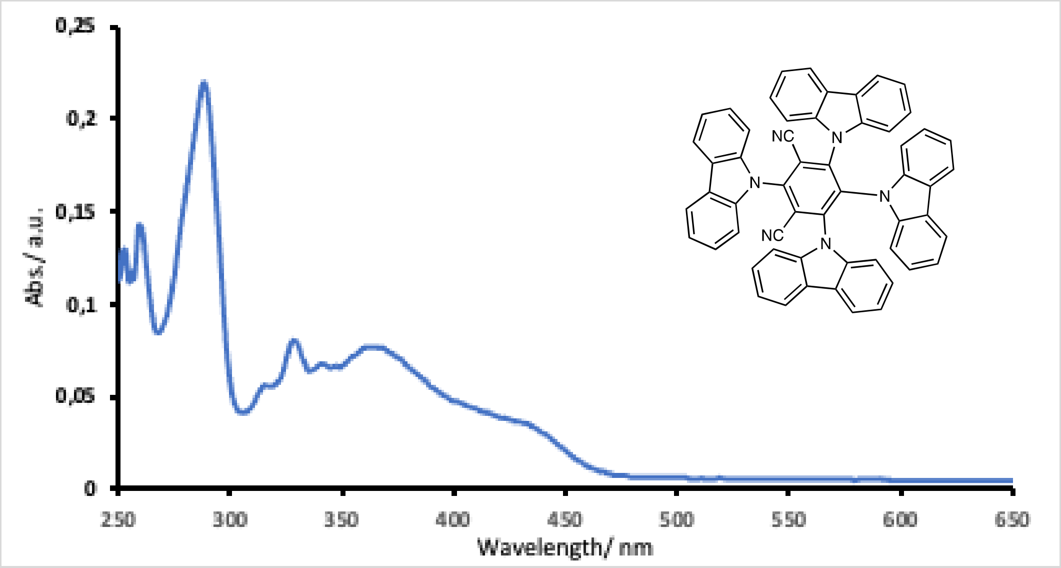

Supplementary Figure 12. UV-Vis. spectrum of a 7.0 x 10^{-6} M solution of 4-CzIPN in NMP.

7.3 Fluorescence Quenching Studies

A 7.0 x 10^{-6} M solution of 4-CzIPN in anhydrous and degassed NMP was prepared in a nitrogen filled glovebox. 4-CzIPN solution (2.0 mL) was transferred to a 4 mL quartz cuvette (path length: l = 1.0 cm) under an atmosphere of nitrogen, where upon irradiation at 450 nm an emission at maximum at 530 nm was observed. Separate solutions of quenchers 2-methyl-2-(tetrahydro-2*H*-pyran-4-yl)-2,3-dihydroquinazolin-4(1*H*)-one (**3e**, 160 mM) and **Ni-I** (2.9 mM) were prepared from anhydrous and degassed NMP in a nitrogen filled glovebox. Distilled and degassed 1-bromo-4-(trifluoromethyl)benzene (**4d**) was used neat. Aliquots of the quencher solutions were added to the solution of 4-CzIPN contained in a quartz cuvette (path length: l = 1.0 cm) under an atmosphere of nitrogen, followed by recording of the emission spectra (Supplementary Figure 13 and Supplementary Figure 15).

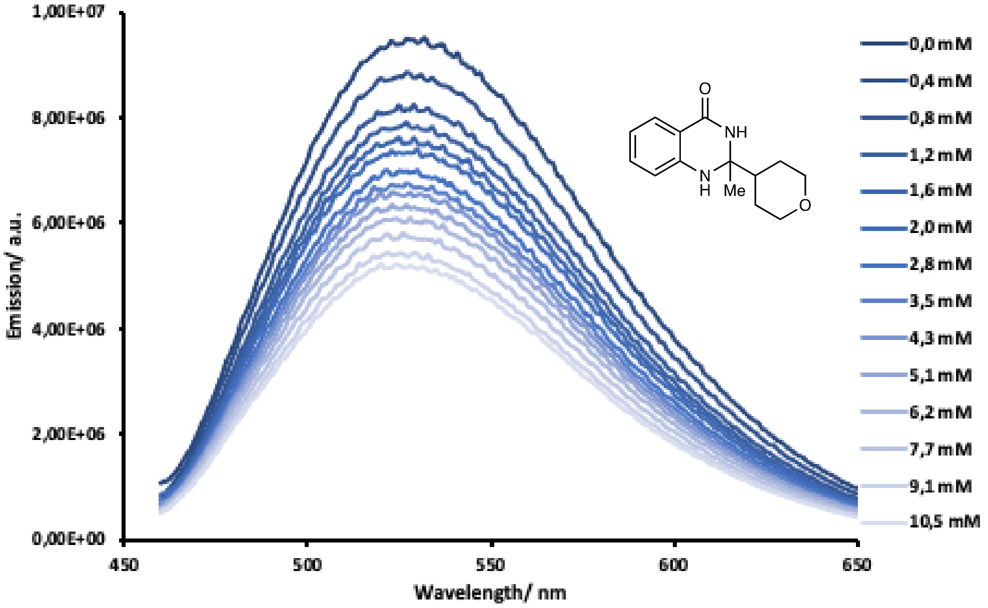

Supplementary Figure 13. Emission spectra of a 7.0 x 10^{-6} M solution of 4-CzIPN in NMP containing varying amounts of 2-methyl-2-(tetrahydro-2*H*-pyran-4-yl)-2,3-dihydroquinazolin-4(1*H*)-one (**2e**) quencher.

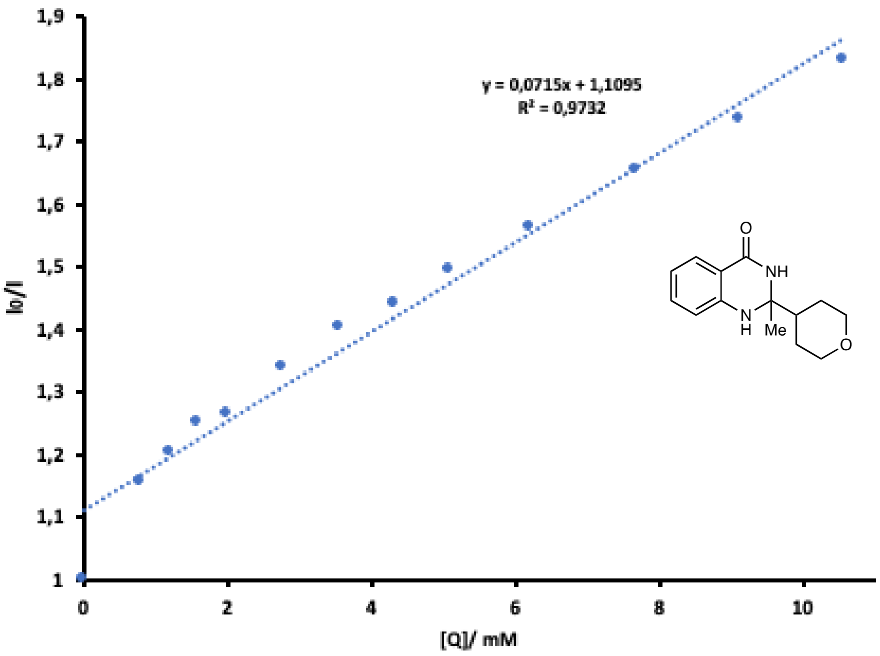

Supplementary Figure 14. Emission intensities observed at 530 nm plotted against 2-methyl-2-(tetrahydro-2*H*-pyran-4-yl)-2,3-dihydroquinazolin-4(1*H*)-one (**2e**) concentration.

By using the gradient of Supplementary Figure 14, the excited-state lifetime of 4-CzIPN (2.15 μs)[28] and the Stern-Volmer equation (1), the fluorescence quenching rate constant was calculated to be 3.32 x 10^7 $M^{-1}.s^{-1}$.

$$\frac{I_0}{I} = 1 + k_q . t_0 . [Q] \qquad \text{(Eq. 1)}$$

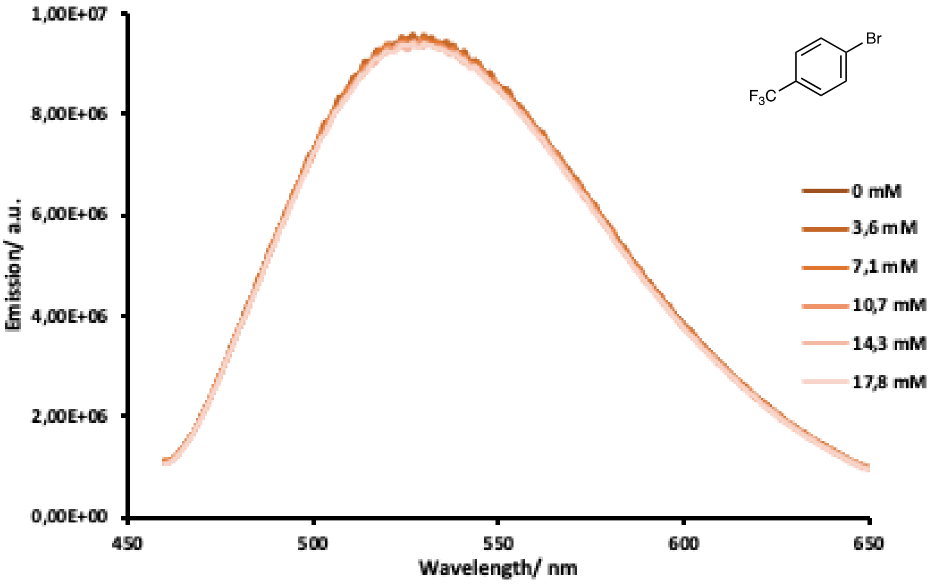

Supplementary Figure 15. Emission spectra of a 7.0 x 10^{-6} M solution of 4-CzIPN in NMP containing varying amounts of 1-bromo-4-(trifluoromethyl)benzene (**4d**) quencher.

No fluorescence quenching of 4-CzIPN was observed in the presence of 1-bromo-4-(trifluoromethyl)benzene.

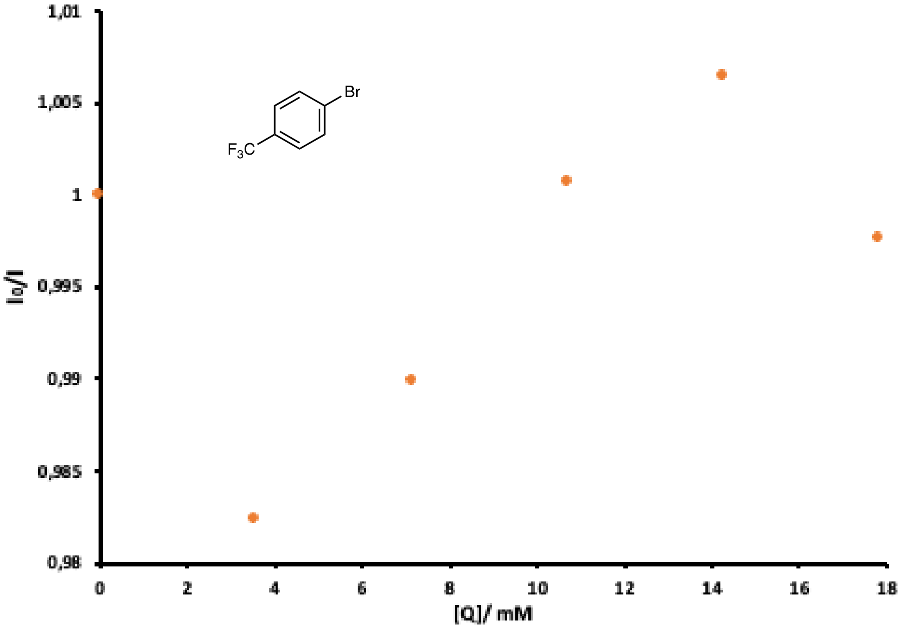

Supplementary Figure 16. Emission intensities observed at 530 nm plotted against 1-bromo-4-(trifluoromethyl)benzene (**4d**) concentration.

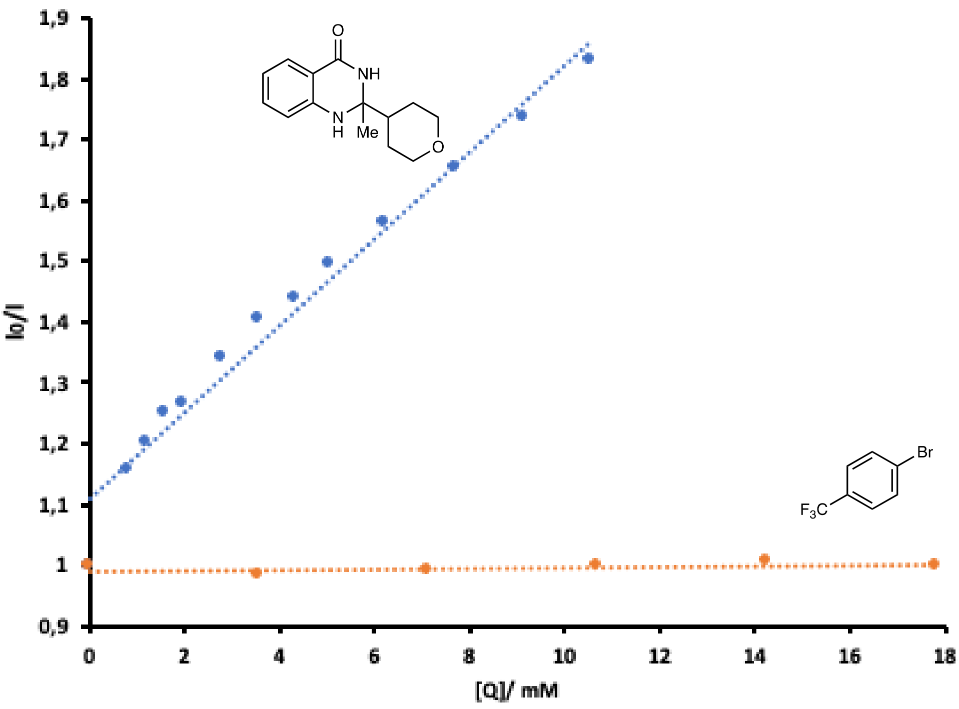

Supplementary Figure 17. Overlay of Supplementary Figure 14 and Supplementary Figure 16.

4-CzIPN fluorescence decay was also observed when a 3.0 mM solution of **Ni-I** in NMP was titrated into a 7.0 x 10^{-6} M solution of 4-CzIPN in NMP (Supplementary Figure 18 and Supplementary Figure 19). However, due to **Ni-**I absorbing light at 450 nm and containing a redox active anion, the degree or if any 4-CzIPN fluorescence quenching is occurring by transfer of photonic energy to the nickel complex cannot be deduced.

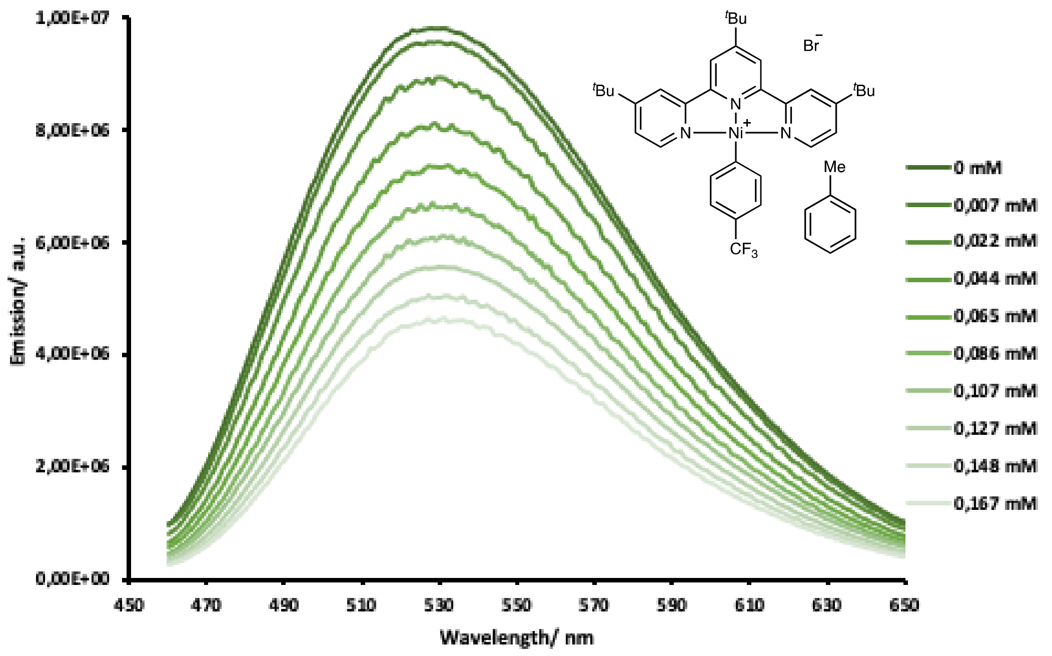

Supplementary Figure 18. Emission intensities observed at 530 nm plotted against **Ni-I** concentration.

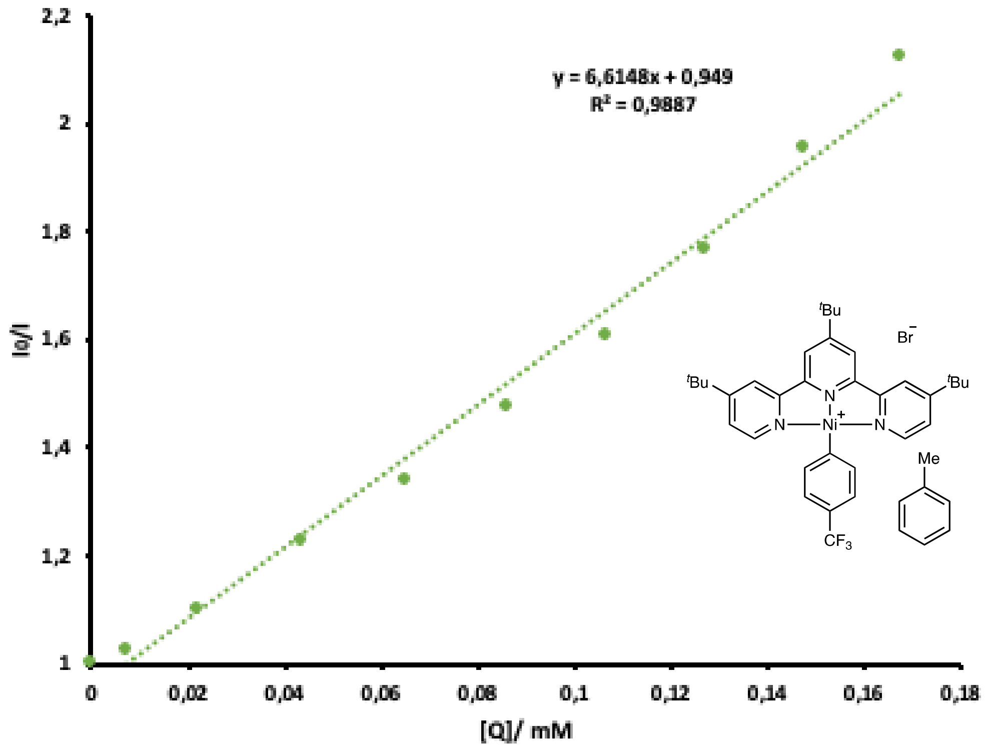

Supplementary Figure 19. Emission intensities observed at 530 nm plotted against **Ni-I** concentration.

7.4 Cyclic Voltammetry Analysis

Cyclic voltammetry was performed on a CH Instruments Electrochemical Analyzer. Analyte (25 μmol) was added to a 0.1 M solution of tetra-*n*-butylammonium hexafluorophosphate in dry, degassed NMP (5 mL). A glassy carbon working electrode, a platinum flag counter electrode and a silver wire reference electrode were used. Three cycles at a scan rate of 100 or 500 mV.s $^{-1}$ were applied.

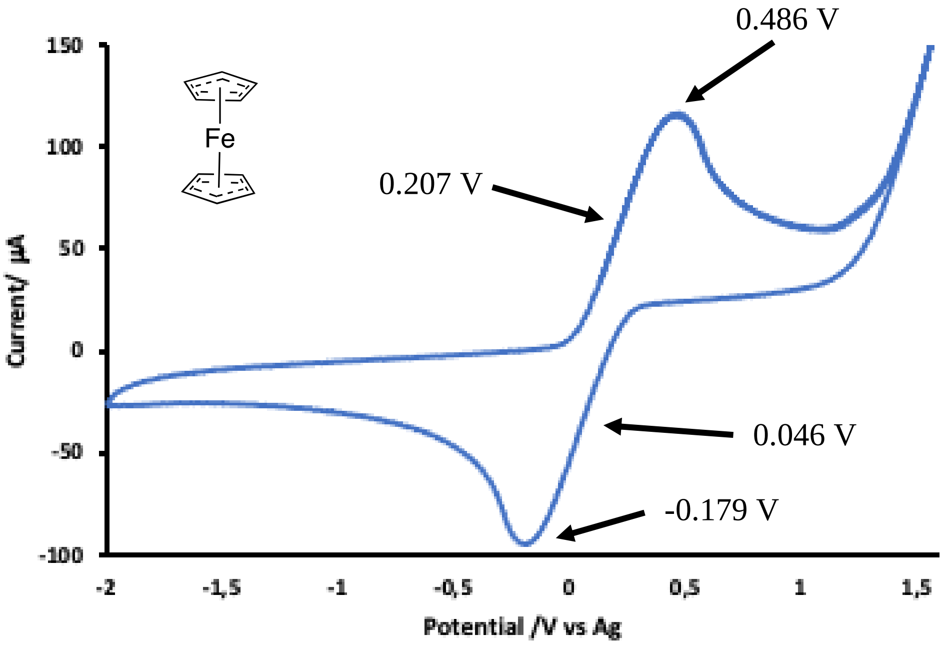

Supplementary Figure 20. Cyclic voltamogram of ferrocene (4.7 mg, 25 μmol) at a scan rate of 500 mV.s$^{-1.}$

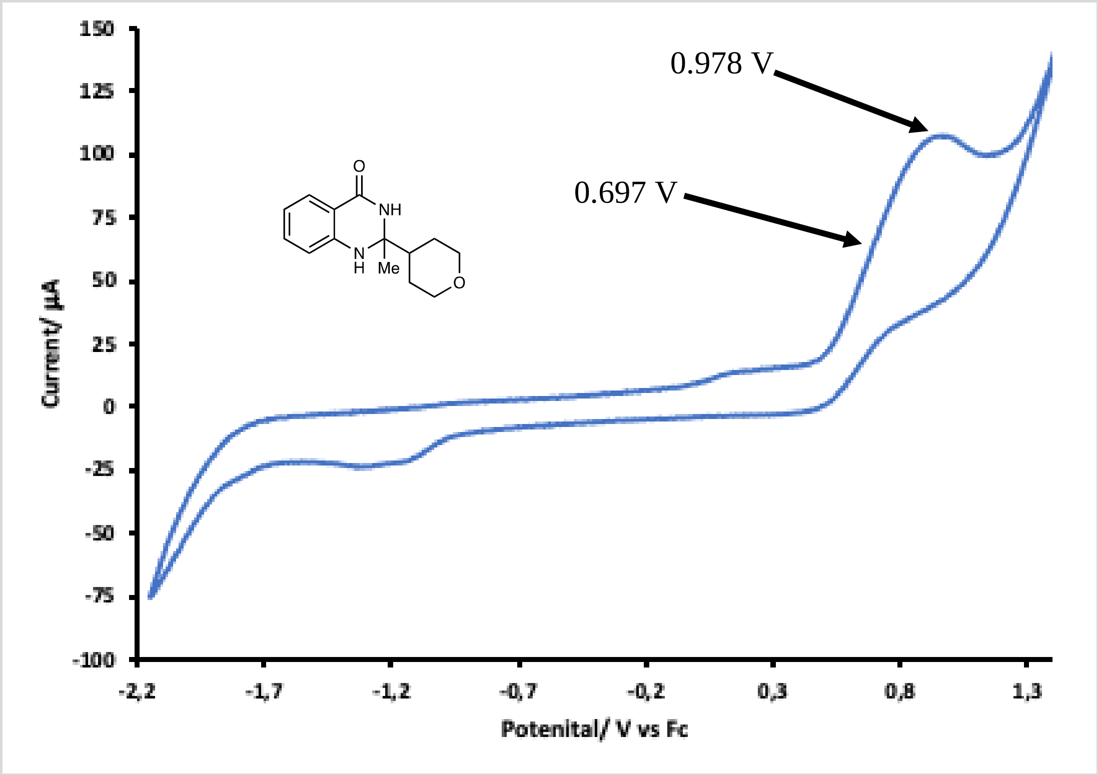

Supplementary Figure 21. Cyclic voltamogram of 2-methyl-2-(tetrahydro-2*H*-pyran-4-yl)-2,3-dihydroquinazolin-4(1*H*)-one (**2e**) (6.2 mg, 25 µmol) at a scan rate of 500 mV.s^{-1}.

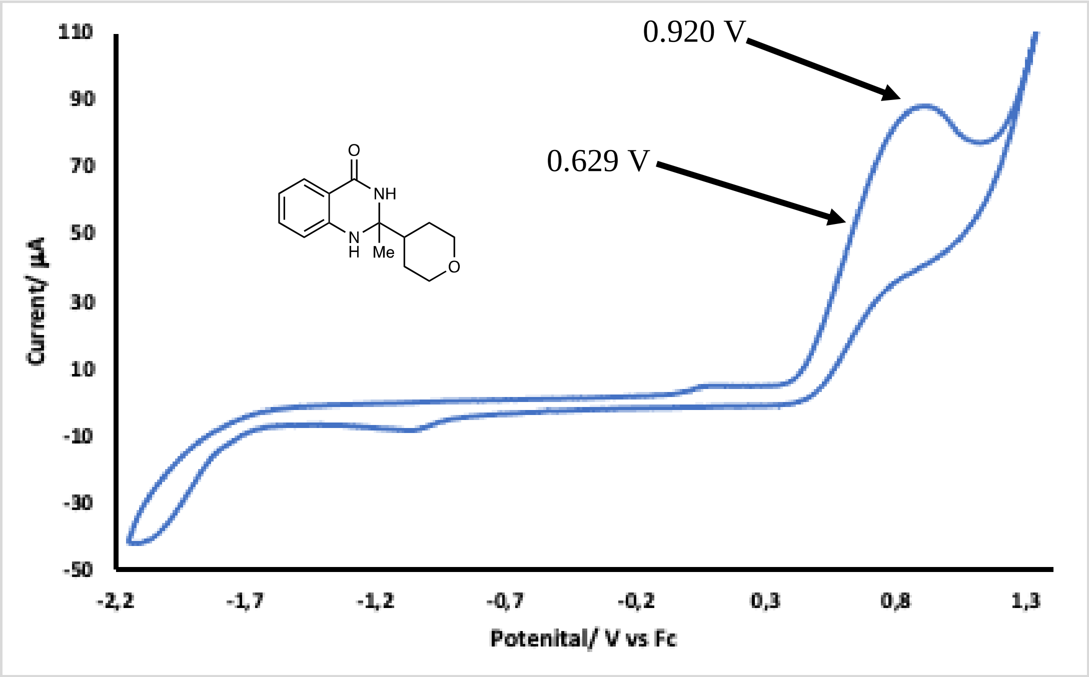

Supplementary Figure 22. Cyclic voltamogram of 2-methyl-2-(tetrahydro-2*H*-pyran-4-yl)-2,3-dihydroquinazolin-4(1*H*)-one (**2e**) (6.2 mg, 25 µmol) at a scan rate of 100 mV.s^{-1}

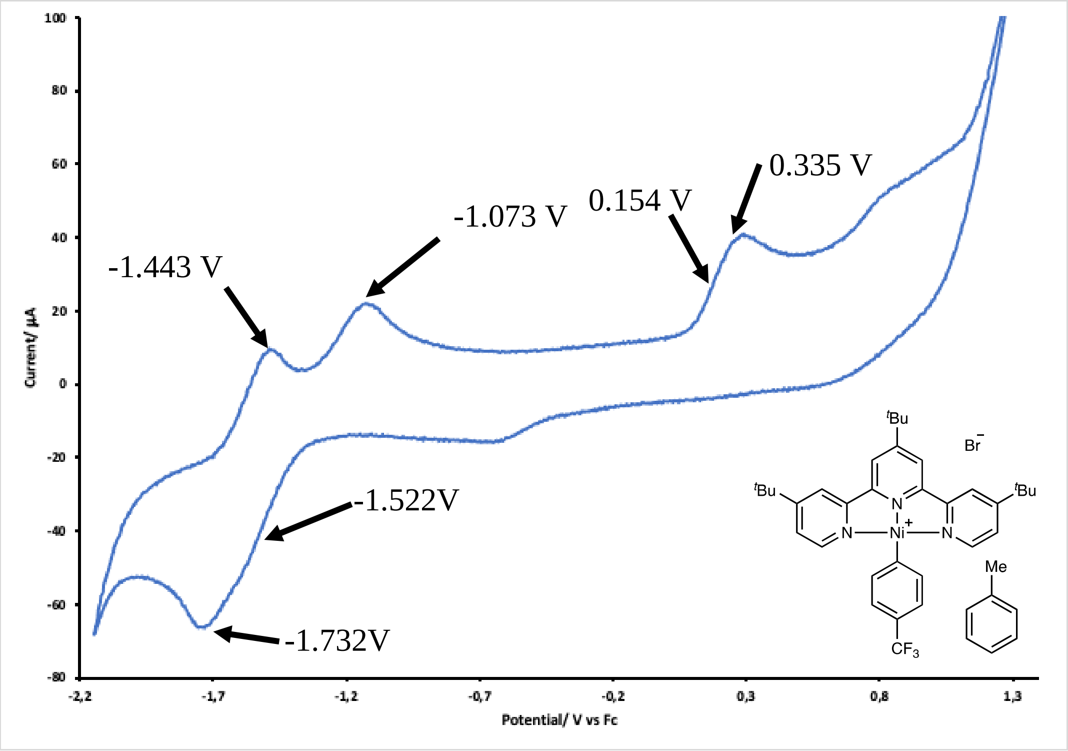

Supplementary Figure 23. Cyclic voltamogram of **Ni-I** (19.4 mg, 25 µmol) at a scan rate of 500 mV.s^{-1}.

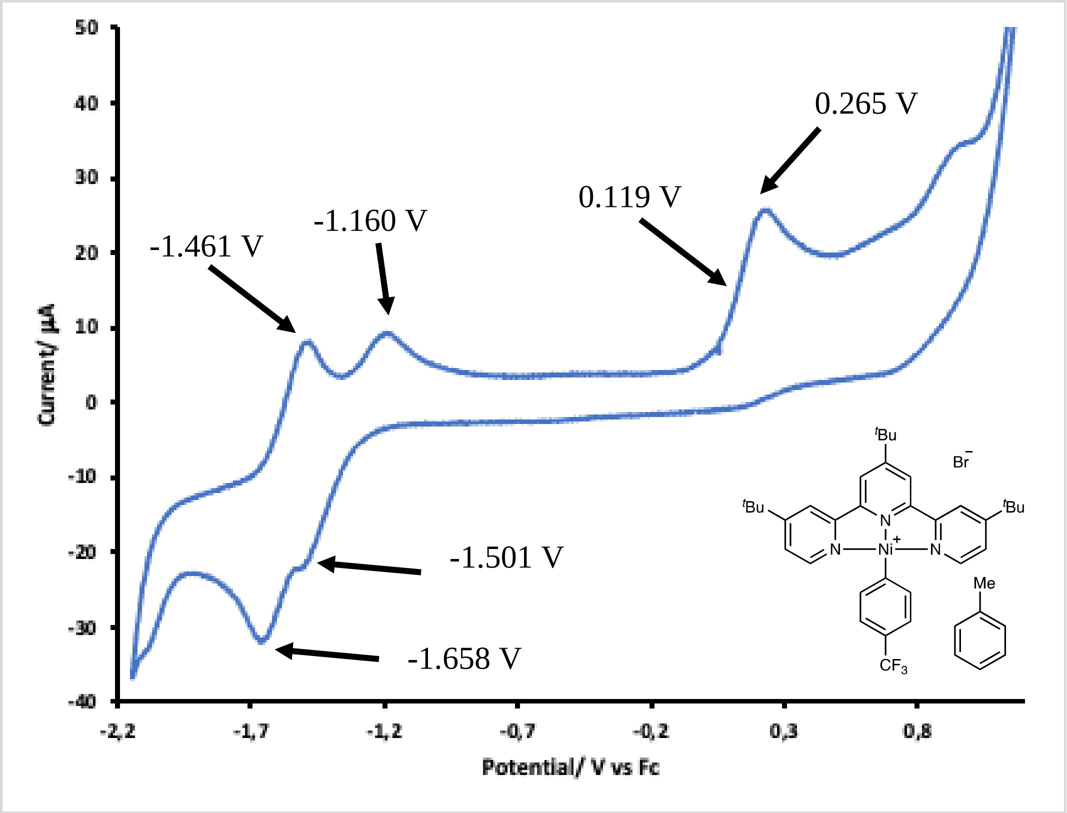

Supplementary Figure 24. Cyclic voltamogram of **Ni-I** (19.4 mg, 25 µmol) at a scan rate of 100 mV.s^{-1}.

Oxidation peaks observed in the CVs of **Ni-I** (Supplementary Figure 23 and Supplementary Figure 24) are likely due to oxidation of the bromide counter ion of the complex (Supplementary Figure 25 and Supplementary Figure 26).

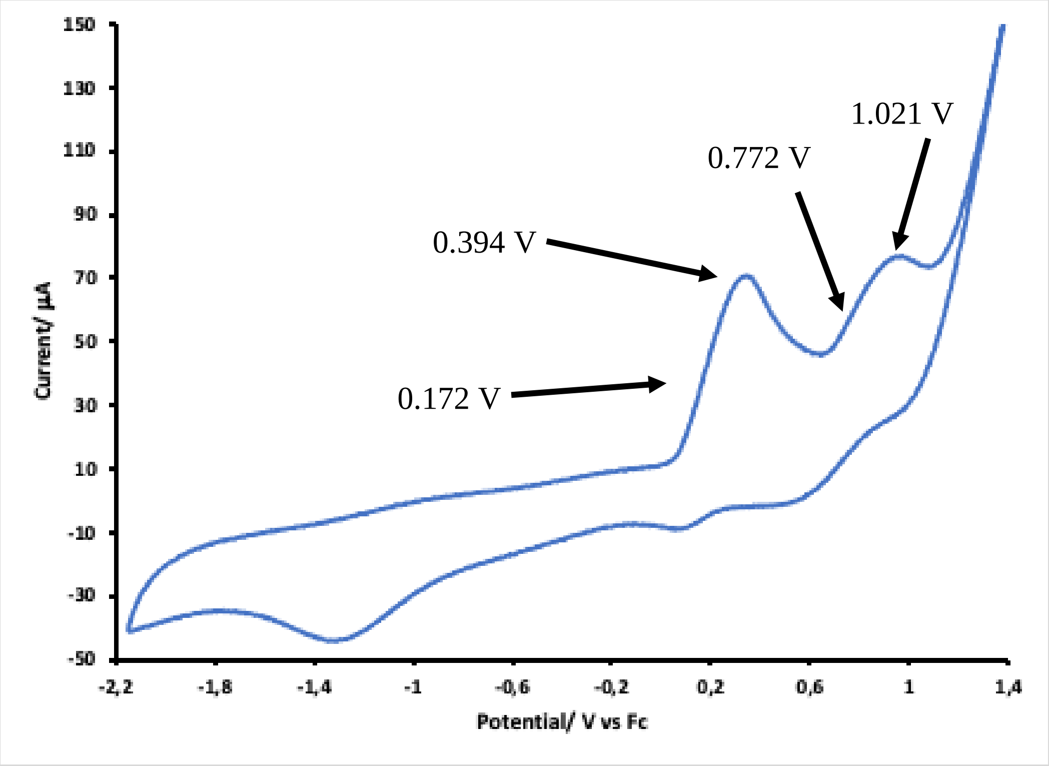

Supplementary Figure 25. Cyclic voltamogram of tetramethylammonium bromide (8.1 mg, 25 μmol) at a scan rate of 500 mV.s^{-1}.

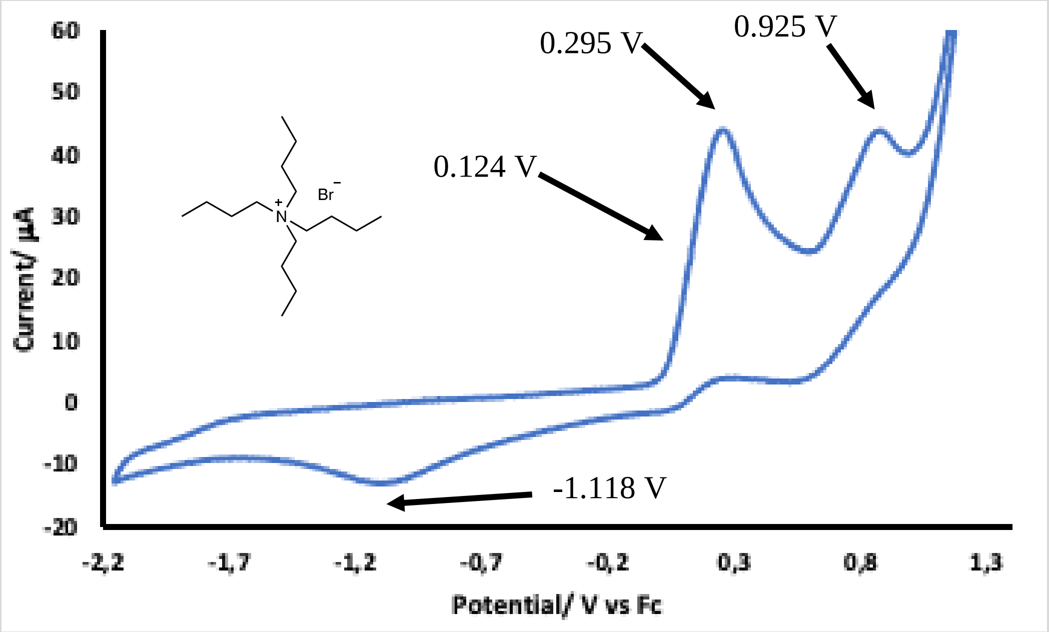

Supplementary Figure 26. Cyclic voltamogram of tetramethylammonium bromide (8.1 mg, 25 μmol) at a scan rate of 100 mV.s^{-1}.

7.5 Radical Clock Experiments

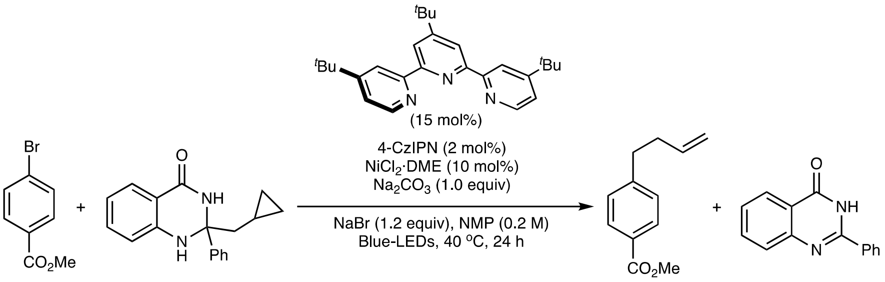

1 **2t** **10** **11**

An oven-dried 8 mL screw-cap test tube was charged with a stirring bar, 4-CzIPN (3.2 mg, 2 mol%), $NiCl_2$·DME (4.4 mg, 10 mol%), 4,4',4''-tri-tert-butyl-2,2':6'2''-terpyridine (12.1 mg, 15 mol%), NaBr (24.7 mg, 1.2 equiv), methyl 4-bromobenzoate (43.0 mg, 0.2 mmol) and 2-(cyclopropylmethyl)-2-phenyl-2,3-dihydroquinazolin-4(1*H*)-one (66.7 mg, 1.2 equiv). The test tube was taken into a nitrogen-filled glovebox where Na_2CO_3 (21.2 mg, 1.0 equiv) was added to the reaction vessel. The reaction vessel was sealed with a screw cap and removed from the glovebox. Afterwards, NMP (1.0 mL, 0.2 M) was added by syringe. Parafilm was used to ensure the tightness of the reaction system. The reaction mixture was stirred at rt for 1 min. before exposure to blue LED irradiation at 40 °C for 24 hours. The reaction mixture was quenched with brine (10 mL) and extracted with ethyl acetate (3 x 5 mL). The combined organic extracts were dried (Na_2SO_4) and concentrated under reduced pressure yielding crude material, which was purified by silica gel chromatography (0 to 25% EtOAc in hexane), affording **10** as a colourless liquid (31.3 mg, 91%) and **11** as a white solid (47.1 mg, 88%).

Methyl 4-(but-3-en-1-yl)benzoate (10). ^{1}H NMR (400 MHz, $CDCl_3$) δ 7.99 – 7.93 (m, 2H), 7.26 (d, *J* = 8.3 Hz, 2H), 5.84 (ddt, *J* = 16.9, 10.2, 6.6 Hz, 1H), 5.08 – 4.96 (m, 2H), 3.91 (s, 3H), 2.81 – 2.73 (m, 2H), 2.44 – 2.35 (m, 2H) ppm. **^{13}C NMR (101 MHz, $CDCl_3$)** δ 167.3, 147.5, 137.6, 129.8, 128.6, 128.0, 115.5, 52.1, 35.5, 35.2 ppm. Spectral data was in agreement with the literature.[9]

2-Phenylquinazolin-4(3*H*)-one (11). M.p.: 233 °C. **^{1}H NMR (400 MHz, DMSO-*d*$_6$)** δ 8.22 – 8.09 (m, 3H), 7.84 – 7.75 (m, 1H), 7.74 – 7.68 (m, 1H), 7.59 – 7.44 (m, 4H) ppm. **^{13}C NMR (101 MHz, DMSO)** δ 162.3, 152.3, 148.7, 134.6, 132.7, 131.4, 128.6, 127.8, 127.5, 126.5, 125.9, 121.0 ppm. Spectral data was in agreement with the literature.[29]

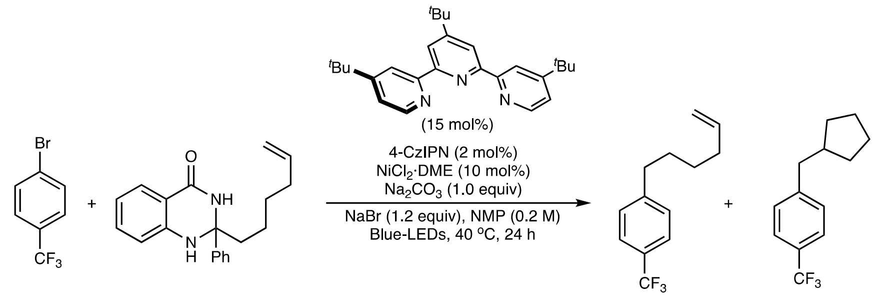

An oven-dried 8 mL screw-cap test tube was charged with a stirring bar, 4-CzIPN (1.6 mg, 2 mol%), $NiCl_2$·DME (2.2 mg, 10 mol%), 4,4',4"-tri-tert-butyl-2,2':6'2"-terpyridine (6.0 mg, 15 mol%), NaBr (12.4 mg, 1.2 equiv), 1-bromo-4-(trifluoromethyl)benzene (22.5 mg, 0.10 mmol), and 2-(hex-5-en-1-yl)-2-phenyl-2,3-dihydroquinazolin-4(1*H*)-one (36.7 mg, 0.12 mmol). The test tube was taken into a nitrogen-filled glovebox where Na_2CO_3 (10.6 mg, 1.0 equiv) was added to the reaction vessel. The reaction vessel was sealed with a screw cap and removed from the glovebox. Afterwards, NMP (0.5 mL, 0.2 M) was added by syringe. Parafilm was used to ensure the tightness of the reaction system. The reaction mixture was stirred at rt for 1 min. before exposure to blue LED irradiation at 40 °C for 24 hours. The reaction mixture was quenched with brine (10 mL) and extracted with ethyl acetate (3 x 5 mL). The combined organic extracts were dried (Na_2SO_4) and concentrated under reduced pressure yielding crude material, which was purified by silica gel chromatography (0 to 1% EtOAc in hexane), affording **12** and **13** as a colourless liquid (13.2 mg, 58%, 0.62:0.38 **12**:**13** ratio).

IR (film, cm^{-1}): 2930, 2855, 1622, 1324, 1166, 1118, 1066. **^{1}H NMR (500 MHz, $CDCl_3$)** δ 7.55 (d, *J* = 8.1 Hz, 0.8H A), 7.54 (d, *J* = 8.1 Hz, 1.2H B), 7.32 – 7.29 (m, 0.8H A + 1.2H B), 5.82 (ddt, *J* = 16.9, 10.2, 6.7 Hz, 0.4H A), 5.03 (dq, *J* = 17.1, 1.7 Hz, 0.4H A), 4.98 (ddt, *J* = 10.2, 2.2, 1.2 Hz, 0.4H A), 2.70 (t, *J* = 7.4 Hz, 0.8H A), 2.69 (d, *J* = 7.5 Hz, 1.2H B), 2.16 – 2.07 (m, 0.8H A + 0.6H B), 1.77 – 1.71 (m, 1.2H B), 1.70 – 1.64 (m, 0.8H A + 1.2H B), 1.58 – 1.54 (m, 1.2H, B), 1.46 (quint., *J* = 7.5 Hz, 0.8H A), 1.24 – 1.17 (m, 1.2H, B). **^{13}C NMR (126 MHz, $CDCl_3$)** δ 146.8 (C_{quat} A), 146.5 (C_{quat} B), 138.6 (*C*H A), 129.0 (2 x Ar*C*H B), 128.7 (2 x Ar*C*H A), 128.0 (C_{quat} A + B , q, J_{CF} = 32.2 Hz), 125.2 (2 x Ar*C*H A, q, J_{CF} = 3.7 Hz), 125.1 (2 x Ar*C*H B, q, J_{CF} = 3.7 Hz), 124.4 (CF_3 A + B, q, J_{CF} = 271.8 Hz), 114.6 (CH_2 A), 41.9 (CH_2 B), 41.8 (*C*H B), 35.6 (CH_2 A), 33.5 (CH_2 A), 32.4 (2 x CH_2 B), 30.6 (CH_2 A), 28.4 (CH_2 A), 24.9 (2 x CH_2 B). **^{19}F NMR (471 MHz, DMSO-d_6)** δ -62.43 (B), -62.40 (A). **HRMS** [APCI$^+$] *calcd.* for ($C_{13}H_{15}F_2$) [M-F]$^+$: 209.1136, *found*: 209.1148.

7.6 Radical Cyclisation as a Function of Catalyst Loading

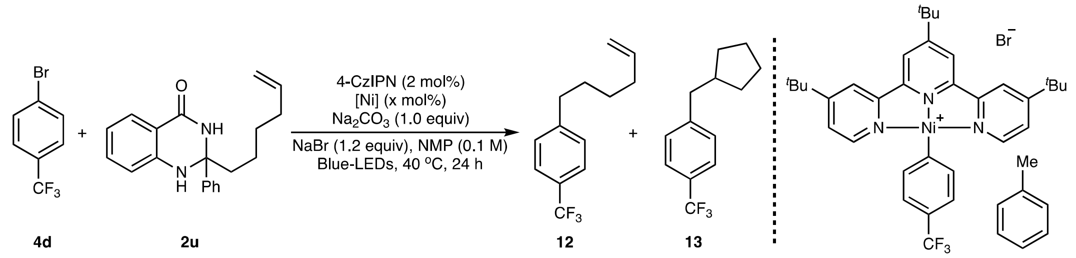

An oven-dried 8 mL screw-cap test tube containing a stirring bar was charged with sodium bromide (12 mg, 0.12 mmol), 4-CzIPN (1.6 mg, 2 mol%) and 2-(hex-5-en-1-yl)-2-phenyl-2,3-dihydroquinazolin-4(1*H*)-one (37 mg, 0.12 mmol). The reaction vessel was taken into a nitrogen-filled glove box, then sequentially charged with nickel complex **Ni-I**, sodium carbonate (11 mg, 0.1 mmol), NMP (1.0 mL) and 1-bromo-4-(trifluoromethyl)benzene (14 µL, 23 mg, 0.1 mmol). The reaction mixture was stirred for 3 minutes, then the reaction vessel was sealed, removed from the glovebox and the screw cap was further sealed with parafilm. The reaction mixture was stirred while exposed to blue LED irradiation for 25 hours at 40 °C. The reaction was quenched with brine (5 mL) and extracted with EtOAc (5 x 3 mL). The combined organic extracts were dried ($MgSO_4$) and concentrated under reduced pressure. The received crude residue was purified by silica gel chromatography using hexane/EtOAc (100:1), giving a mixture of **12** and **13** (50-60%) as a colourless oil. ^{19}F NMR was used to evaluate the ratio of **13** (-62.43 ppm) against **12** (-62.40 ppm).

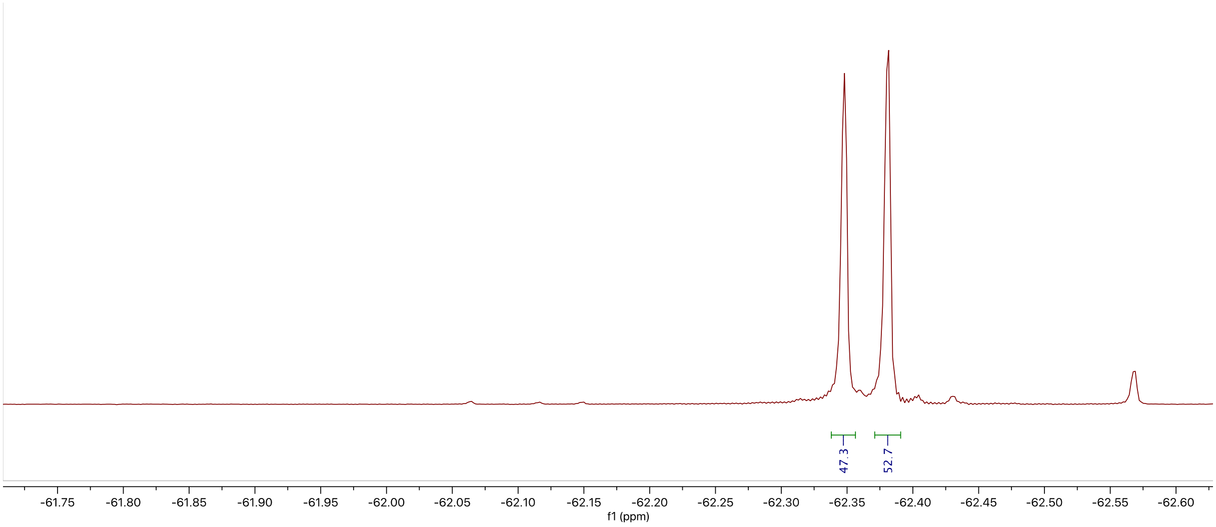

Supplementary Figure 27. ^{19}F NMR spectra of compound **12** and **13** at 15 mol% **Ni-I** loading.

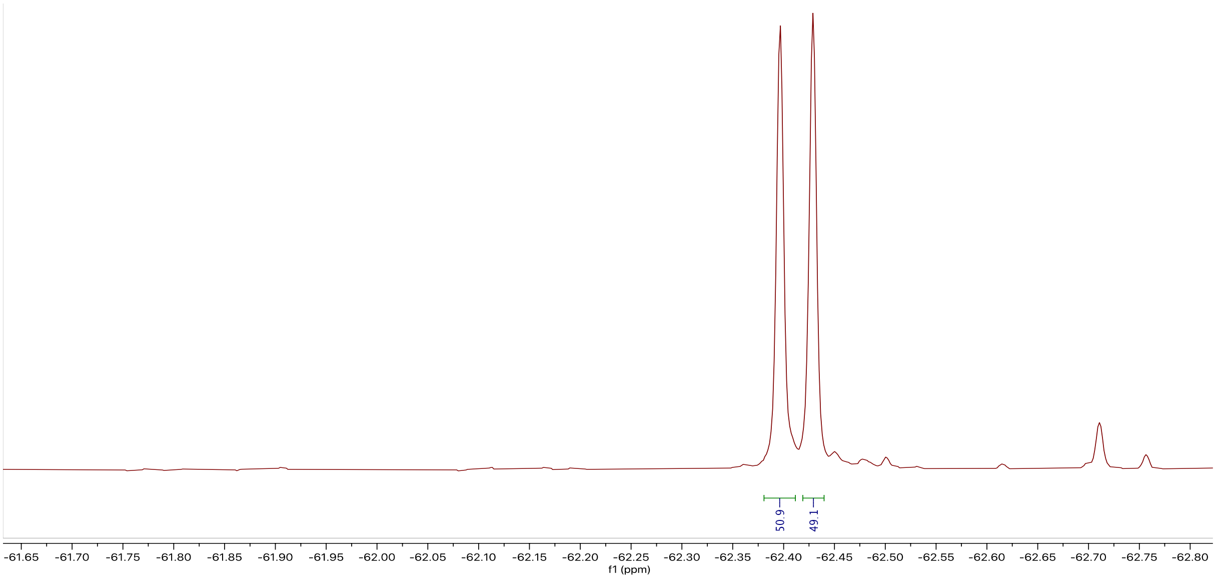

Supplementary Figure 28. ^{19}F NMR spectra of compound **12** and **13** at 12.5 mol% **Ni-I** loading.

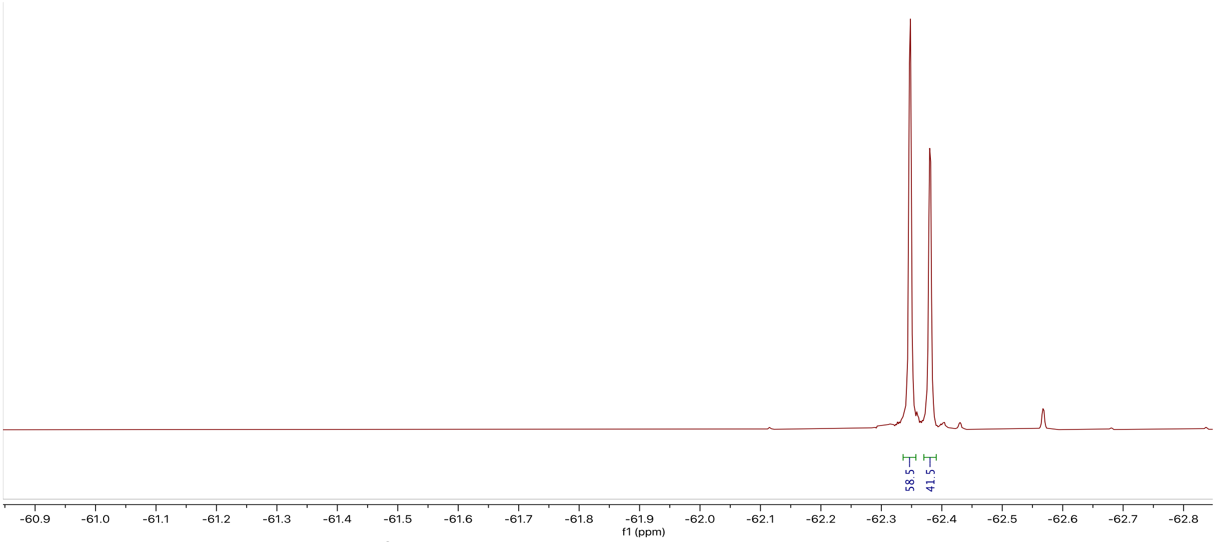

Supplementary Figure 29. ^{19}F NMR spectra of compound **12** and **13** at 10 mol% **Ni-I** loading.

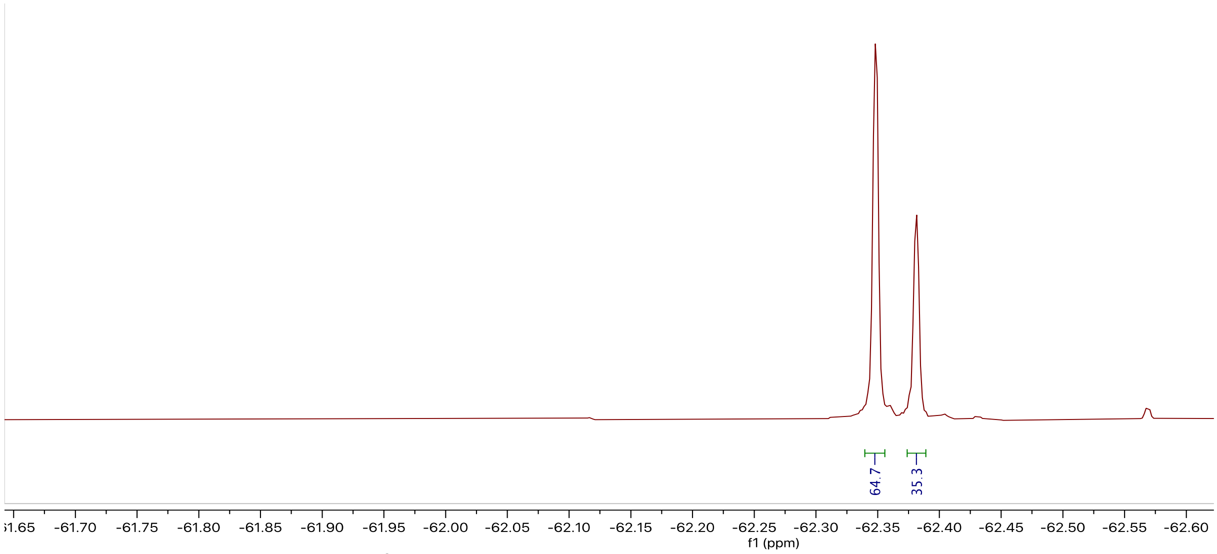

Supplementary Figure 30. ^{19}F NMR spectra of compound **12** and **13** at 7.5 mol% **Ni-I** loading.

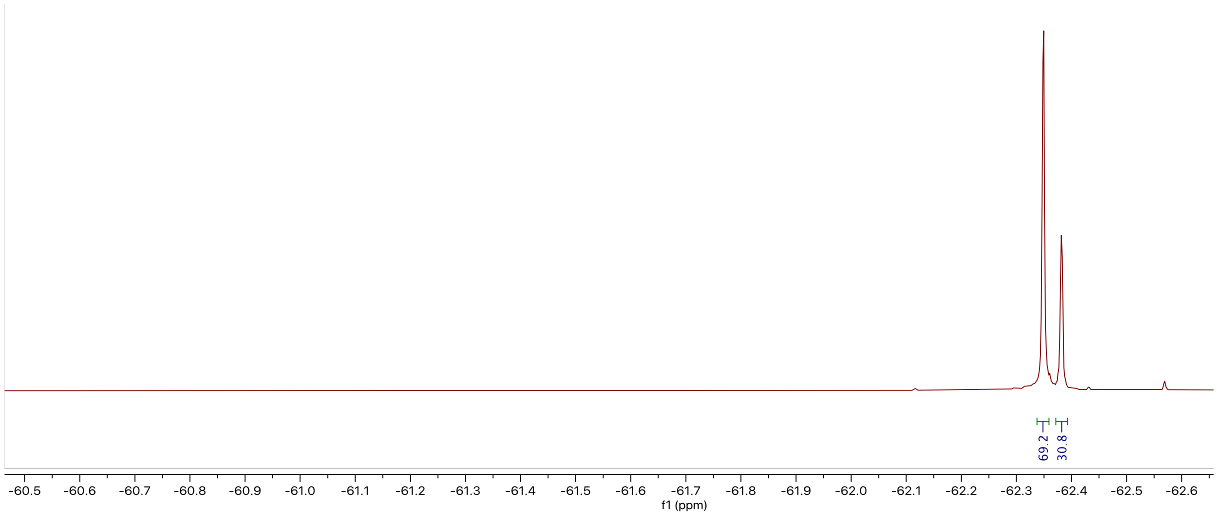

Supplementary Figure 31. ^{19}F NMR spectra of compound **12** and **13** at 5 mol% **Ni-I** loading.

**Ni-I** mol%	**12** selectivity/%	**13** selectivity/%
15	52.7	47.3
12.5	50.9	49.1
10	41.5	58.5
7.5	35.3	64.7
5	30.8	69.2

Supplementary Table 2. Tabulated ratios of cross-coupling products **12** and **13** at different **Ni-I** catalyst loadings.

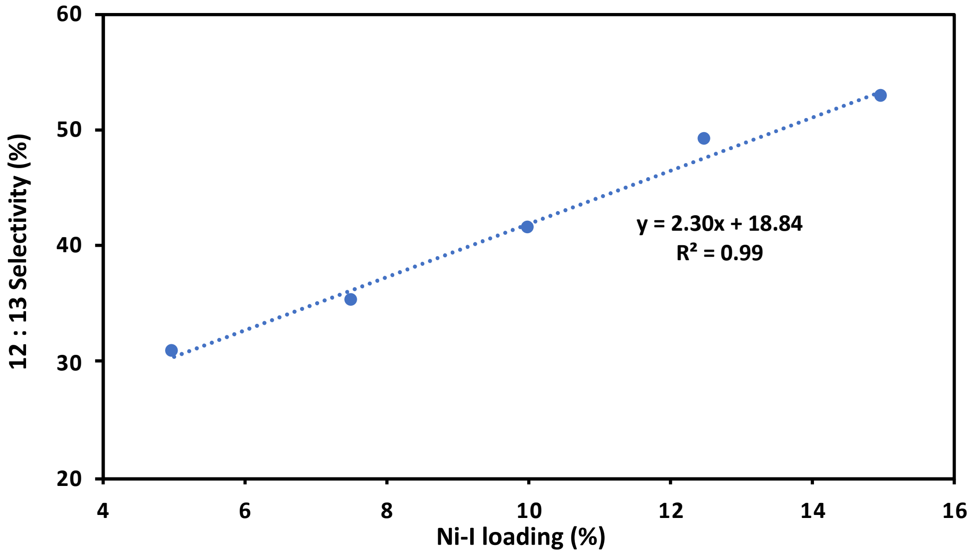

Supplementary Figure 32. Plot ratios of cross-coupling products **12** and **13** at different **Ni-I** catalyst loadings.

7.7 TEMPO Radical Probe Experiments

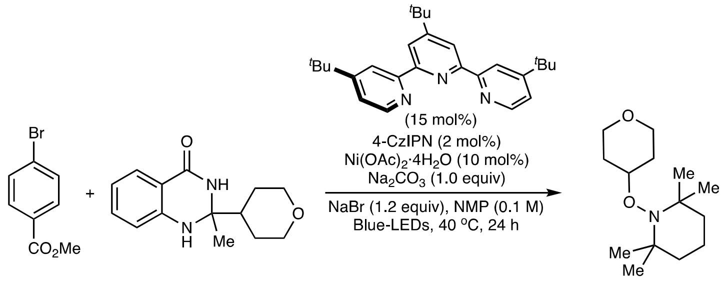

1 2e 9

detected by GCMS

An oven-dried 8 mL screw-cap test tube was charged with a stirring bar, 4-CzIPN (1.6 mg, 2 mol%), $Ni(OAc)_2{\cdot}4H_2O$ (2.5 mg, 10 mol%), 4,4',4"-tri-tert-butyl-2,2':6'2"-terpyridine (6.0 mg, 15 mol%), NaBr (12.4 mg, 1.2 equiv), methyl 4-bromobenzoate (21.5 mg, 0.1 mmol), 2-methyl-2-(tetrahydro-2H-pyran-4-yl)-2,3-dihydroquinazolin-4(1*H*)-one (29.5 mg, 1.2 equiv) and TEMPO (18.7 mg, 1.2 equiv). The test tube was taken into a nitrogen-filled glovebox where Na_2CO_3 (10.6 mg, 1.0 equiv) was added to the reaction vessel. The reaction vessel was sealed with a screw cap and removed from the glovebox. Afterwards, NMP (1.0 mL, 0.1 M) was added by syringe. Parafilm was used to ensure the tightness of the reaction system. The reaction mixture was stirred at rt for 1 min. before exposure to blue LED irradiation at 40 °C for 24 hours. After that, the reaction mixture was analyzed by GC-mass and the corresponding TEMPO- adduct was detected by GC mass spectroscopy.

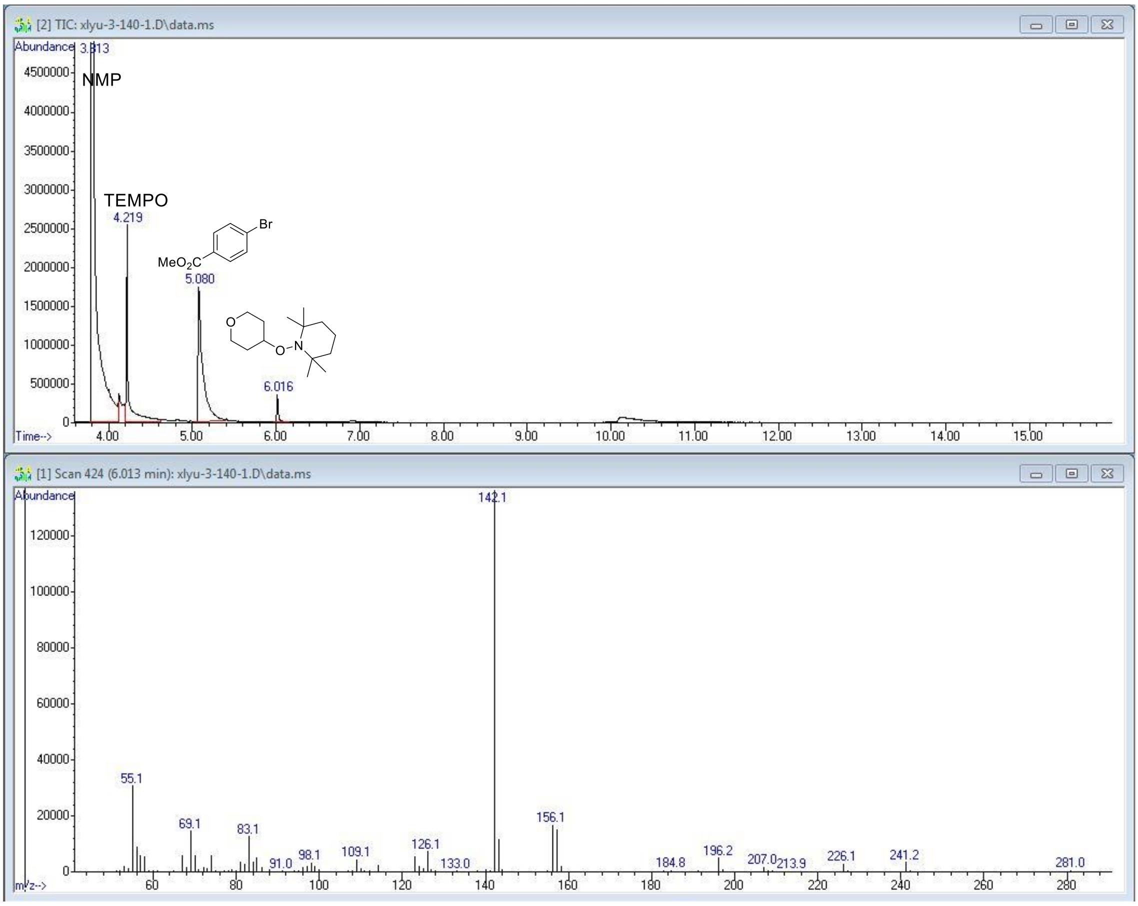

Supplementary Figure 33. GCMS spectra of TEMPO radical trapping experiment.

7.8 X-ray diffraction of Ni-I

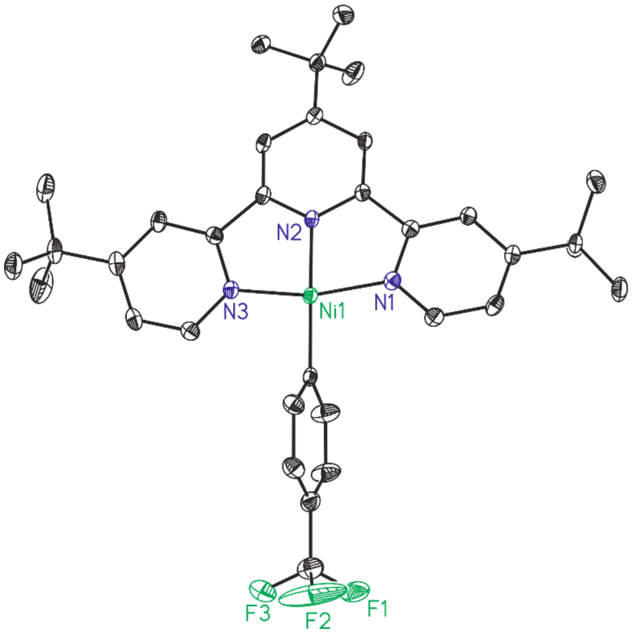

Supplementary Figure 34. ORTEP diagram of **Ni-I**.

Supplementary Table 3. Crystallographic Data

Empirical formula	C35 H41 Br Cl2 F3 N3 Ni	
Formula weight	770.23	
Temperature	100(2)K	
Wavelength	0.71073 Å	
Crystal system	monoclinic	
Space group	C 2/c	
Unit cell dimensions	a = 17.5031(16)Å	a= 90°.
	b = 22.317(2)Å	b = 91.787(3)°.
	c = 17.5404(16)Å	g = 90°.
Volume	6848.3(11) $Å^3$	
Z	8	
Density (calculated)	1.494 Mg/m^3	
Absorption coefficient	1.935 mm^{-1}	
F(000)	3168	
Crystal size	0.400 x 0.250 x 0.250 mm^3	
Theta range for data collection	1.825 to 26.464°.	
Index ranges	-21<=h<=21, -26<=k<=27, -21<=l<=21	
Reflections collected	33394	
Independent reflections	7029[R(int) = 0.0420]	

Completeness to theta =26.464°	99.5%
Absorption correction	Multi-scan
Max. and min. transmission	0.74 and 0.59
Refinement method	Full-matrix least-squares on F^2
Data / restraints / parameters	7029/ 46/ 456
Goodness-of-fit on F^2	0.916
Final R indices [I>2sigma(I)]	R1 = 0.0350, wR2 = 0.1112
R indices (all data)	R1 = 0.0482, wR2 = 0.1216
Largest diff. peak and hole	0.932 and -0.940 e.Å^{-3}

Supplementary Table 4. Bond lengths [Å] and angles [°] for Ni-I

Bond lengths----

F1	C34	1.320(3)
C1	N1	1.335(3)
C1	C2	1.384(4)
C1	H1	0.9500
N1	C5	1.373(3)
N1	Ni1	1.917(2)
Ni1	N2	1.859(2)
Ni1	C28	1.882(3)
Ni1	N3	1.909(2)
F2	C34	1.315(4)
C2	C3	1.390(4)
C2	H2	0.9500
N2	C10	1.334(3)
N2	C6	1.341(3)
F3	C34	1.335(4)
C3	C4	1.400(3)
C3	C16	1.526(3)
N3	C15	1.345(3)
N3	C11	1.367(3)
C4	C5	1.372(3)
C4	H4	0.9500
C5	C6	1.478(3)
C6	C7	1.383(3)
C7	C8	1.411(3)
C7	H7	0.9500

C8	C9	1.399(3)
C8	C20	1.520(4)
C9	C10	1.377(3)
C9	H9	0.9500
C10	C11	1.482(3)
C11	C12	1.379(3)
C12	C13	1.394(4)
C12	H12	0.9500
C13	C14	1.396(4)
C13	C24	1.524(4)
C14	C15	1.379(4)
C14	H14	0.9500
C15	H15	0.9500
C16	C17	1.524(3)
C16	C18	1.531(4)
C16	C19	1.534(4)
C17	H17A	0.9800
C17	H17B	0.9800
C17	H17C	0.9800
C18	H18A	0.9800
C18	H18B	0.9800
C18	H18C	0.9800
C19	H19A	0.9800
C19	H19B	0.9800
C19	H19C	0.9800
C20	C23	1.527(4)
C20	C21	1.528(4)
C20	C22	1.541(4)
C21	H21A	0.9800
C21	H21B	0.9800
C21	H21C	0.9800
C22	H22A	0.9800
C22	H22B	0.9800
C22	H22C	0.9800
C23	H23A	0.9800
C23	H23B	0.9800
C23	H23C	0.9800
C24	C26	1.523(4)

C24	C27	1.526(4)	
C24	C25	1.542(4)	
C25	H25A	0.9800	
C25	H25B	0.9800	
C25	H25C	0.9800	
C26	H26A	0.9800	
C26	H26B	0.9800	
C26	H26C	0.9800	
C27	H27A	0.9800	
C27	H27B	0.9800	
C27	H27C	0.9800	
C28	C33	1.391(4)	
C28	C29	1.398(4)	
C29	C30	1.390(4)	
C29	H29	0.9500	
C30	C31	1.384(4)	
C30	H30	0.9500	
C31	C32	1.382(4)	
C31	C34	1.482(4)	
C32	C33	1.380(4)	
C32	H32	0.9500	
C33	H33	0.9500	
C1B	Cl1B	1.753(3)	
C1B	Cl1B#	1.754(3)	2_655
C1B	H1BA	0.9900	
C1B	H1BB	0.9900	
C1C	Cl1C	1.786(7)	
C1C	Cl2C	1.799(8)	
C1C	H1CA	0.9900	
C1C	H1CB	0.9900	
C1D	Cl1D	1.775(9)	
C1D	Cl2D	1.781(9)	
C1D	H1DA	0.9900	
C1D	H1DB	0.9900	

Angles----------

N1	C1	C2	123.4(2)
N1	C1	H1	118.3

C2	C1	H1	118.3
C1	N1	C5	117.2(2)
C1	N1	Ni1	128.63(17)
C5	N1	Ni1	113.84(16)
N2	Ni1	C28	174.43(10)
N2	Ni1	N3	82.66(9)
C28	Ni1	N3	96.84(10)
N2	Ni1	N1	82.33(9)
C28	Ni1	N1	98.00(9)
N3	Ni1	N1	164.97(9)
C1	C2	C3	119.7(2)
C1	C2	H2	120.1
C3	C2	H2	120.1
C10	N2	C6	121.4(2)
C10	N2	Ni1	119.02(17)
C6	N2	Ni1	119.08(17)
C2	C3	C4	116.8(2)
C2	C3	C16	123.5(2)
C4	C3	C16	119.7(2)
C15	N3	C11	117.0(2)
C15	N3	Ni1	128.87(18)
C11	N3	Ni1	114.00(16)
C5	C4	C3	120.5(2)
C5	C4	H4	119.7
C3	C4	H4	119.7
C4	C5	N1	121.9(2)
C4	C5	C6	124.7(2)
N1	C5	C6	113.3(2)
N2	C6	C7	120.5(2)
N2	C6	C5	110.3(2)
C7	C6	C5	129.2(2)
C6	C7	C8	119.4(2)
C6	C7	H7	120.3
C8	C7	H7	120.3
C9	C8	C7	118.1(2)
C9	C8	C20	121.9(2)
C7	C8	C20	120.0(2)
C10	C9	C8	119.4(2)

C10	C9	H9	120.3
C8	C9	H9	120.3
N2	C10	C9	121.2(2)
N2	C10	C11	110.4(2)
C9	C10	C11	128.2(2)
N3	C11	C12	122.9(2)
N3	C11	C10	113.0(2)
C12	C11	C10	124.0(2)
C11	C12	C13	120.0(2)
C11	C12	H12	120.0
C13	C12	H12	120.0
C12	C13	C14	116.5(2)
C12	C13	C24	123.0(2)
C14	C13	C24	120.6(2)
C15	C14	C13	121.0(2)
C15	C14	H14	119.5
C13	C14	H14	119.5
N3	C15	C14	122.4(2)
N3	C15	H15	118.8
C14	C15	H15	118.8
C17	C16	C3	112.1(2)
C17	C16	C18	109.3(2)
C3	C16	C18	109.8(2)
C17	C16	C19	108.6(2)
C3	C16	C19	107.3(2)
C18	C16	C19	109.6(2)
C16	C17	H17A	109.5
C16	C17	H17B	109.5
H17A	C17	H17B	109.5
C16	C17	H17C	109.5
H17A	C17	H17C	109.5
H17B	C17	H17C	109.5
C16	C18	H18A	109.5
C16	C18	H18B	109.5
H18A	C18	H18B	109.5
C16	C18	H18C	109.5
H18A	C18	H18C	109.5
H18B	C18	H18C	109.5

C16	C19	H19A	109.5
C16	C19	H19B	109.5
H19A	C19	H19B	109.5
C16	C19	H19C	109.5
H19A	C19	H19C	109.5
H19B	C19	H19C	109.5
C8	C20	C23	112.4(2)
C8	C20	C21	108.6(2)
C23	C20	C21	109.1(2)
C8	C20	C22	109.0(2)
C23	C20	C22	108.3(2)
C21	C20	C22	109.4(2)
C20	C21	H21A	109.5
C20	C21	H21B	109.5
H21A	C21	H21B	109.5
C20	C21	H21C	109.5
H21A	C21	H21C	109.5
H21B	C21	H21C	109.5
C20	C22	H22A	109.5
C20	C22	H22B	109.5
H22A	C22	H22B	109.5
C20	C22	H22C	109.5
H22A	C22	H22C	109.5
H22B	C22	H22C	109.5
C20	C23	H23A	109.5
C20	C23	H23B	109.5
H23A	C23	H23B	109.5
C20	C23	H23C	109.5
H23A	C23	H23C	109.5
H23B	C23	H23C	109.5
C26	C24	C13	108.5(2)
C26	C24	C27	110.9(3)
C13	C24	C27	109.0(2)
C26	C24	C25	108.5(3)
C13	C24	C25	112.2(2)
C27	C24	C25	107.9(2)
C24	C25	H25A	109.5
C24	C25	H25B	109.5

H25A	C25	H25B	109.5
C24	C25	H25C	109.5
H25A	C25	H25C	109.5
H25B	C25	H25C	109.5
C24	C26	H26A	109.5
C24	C26	H26B	109.5
H26A	C26	H26B	109.5
C24	C26	H26C	109.5
H26A	C26	H26C	109.5
H26B	C26	H26C	109.5
C24	C27	H27A	109.5
C24	C27	H27B	109.5
H27A	C27	H27B	109.5
C24	C27	H27C	109.5
H27A	C27	H27C	109.5
H27B	C27	H27C	109.5
C33	C28	C29	116.7(2)
C33	C28	Ni1	118.5(2)
C29	C28	Ni1	124.78(19)
C30	C29	C28	121.6(2)
C30	C29	H29	119.2
C28	C29	H29	119.2
C31	C30	C29	120.2(2)
C31	C30	H30	119.9
C29	C30	H30	119.9
C32	C31	C30	119.1(3)
C32	C31	C34	119.4(3)
C30	C31	C34	121.5(2)
C33	C32	C31	120.4(3)
C33	C32	H32	119.8
C31	C32	H32	119.8
C32	C33	C28	122.1(3)
C32	C33	H33	119.0
C28	C33	H33	119.0
F2	C34	F1	105.0(3)
F2	C34	F3	106.0(3)
F1	C34	F3	103.8(3)
F2	C34	C31	114.1(3)

F1	C34	C31	114.1(2)	
F3	C34	C31	112.9(3)	
Cl1B	C1B	Cl1B#	112.0(2)	2_655
Cl1B	C1B	H1BA	109.2	
Cl1B#	C1B	H1BA	109.2	2_655
Cl1B	C1B	H1BB	109.2	
Cl1B#	C1B	H1BB	109.2	2_655
H1BA	C1B	H1BB	107.9	
Cl1C	C1C	Cl2C	105.0(4)	
Cl1C	C1C	H1CA	110.7	
Cl2C	C1C	H1CA	110.7	
Cl1C	C1C	H1CB	110.7	
Cl2C	C1C	H1CB	110.7	
H1CA	C1C	H1CB	108.8	
Cl1D	C1D	Cl2D	118.0(10)	
Cl1D	C1D	H1DA	107.8	
Cl2D	C1D	H1DA	107.8	
Cl1D	C1D	H1DB	107.8	
Cl2D	C1D	H1DB	107.8	
H1DA	C1D	H1DB	107.2	

Supplementary Table 5. Torsion angles [°] for Ni-I

C2	C1	N1	C5	-4.3(4)
C2	C1	N1	Ni1	168.68(19)
N1	C1	C2	C3	-2.4(4)
N3	Ni1	N2	C10	2.72(18)
N1	Ni1	N2	C10	-178.23(19)
N3	Ni1	N2	C6	-169.72(19)
N1	Ni1	N2	C6	9.33(18)
C1	C2	C3	C4	7.0(4)
C1	C2	C3	C16	-170.3(2)
C2	C3	C4	C5	-5.2(4)
C16	C3	C4	C5	172.2(2)
C3	C4	C5	N1	-1.4(4)
C3	C4	C5	C6	-177.6(2)
C1	N1	C5	C4	6.2(3)
Ni1	N1	C5	C4	-167.84(19)
C1	N1	C5	C6	-177.3(2)
Ni1	N1	C5	C6	8.7(2)
C10	N2	C6	C7	-1.7(3)
Ni1	N2	C6	C7	170.59(18)
C10	N2	C6	C5	-178.9(2)
Ni1	N2	C6	C5	-6.7(3)
C4	C5	C6	N2	174.8(2)
N1	C5	C6	N2	-1.7(3)
C4	C5	C6	C7	-2.2(4)
N1	C5	C6	C7	-178.6(2)
N2	C6	C7	C8	2.4(4)
C5	C6	C7	C8	179.1(2)
C6	C7	C8	C9	-0.3(4)
C6	C7	C8	C20	-178.7(2)
C7	C8	C9	C10	-2.3(4)
C20	C8	C9	C10	176.0(2)
C6	N2	C10	C9	-1.1(4)
Ni1	N2	C10	C9	-173.39(18)
C6	N2	C10	C11	174.4(2)
Ni1	N2	C10	C11	2.2(3)
C8	C9	C10	N2	3.1(4)

C8	C9	C10	C11	-171.6(2)
C15	N3	C11	C12	4.4(4)
Ni1	N3	C11	C12	-172.11(19)
C15	N3	C11	C10	-173.0(2)
Ni1	N3	C11	C10	10.5(3)
N2	C10	C11	N3	-8.2(3)
C9	C10	C11	N3	167.0(2)
N2	C10	C11	C12	174.4(2)
C9	C10	C11	C12	-10.4(4)
N3	C11	C12	C13	-3.0(4)
C10	C11	C12	C13	174.1(2)
C11	C12	C13	C14	-1.0(4)
C11	C12	C13	C24	178.3(2)
C12	C13	C14	C15	3.4(4)
C24	C13	C14	C15	-175.9(2)
C11	N3	C15	C14	-1.8(4)
Ni1	N3	C15	C14	174.09(19)
C13	C14	C15	N3	-2.1(4)
C2	C3	C16	C17	-5.6(3)
C4	C3	C16	C17	177.1(2)
C2	C3	C16	C18	-127.3(3)
C4	C3	C16	C18	55.5(3)
C2	C3	C16	C19	113.6(3)
C4	C3	C16	C19	-63.7(3)
C9	C8	C20	C23	-0.8(3)
C7	C8	C20	C23	177.5(2)
C9	C8	C20	C21	120.1(3)
C7	C8	C20	C21	-61.7(3)
C9	C8	C20	C22	-120.8(3)
C7	C8	C20	C22	57.4(3)
C12	C13	C24	C26	121.3(3)
C14	C13	C24	C26	-59.4(3)
C12	C13	C24	C27	-117.9(3)
C14	C13	C24	C27	61.3(3)
C12	C13	C24	C25	1.4(4)
C14	C13	C24	C25	-179.3(2)
N3	Ni1	C28	C33	92.9(2)
N1	Ni1	C28	C33	-84.7(2)

N3	Ni1	C28	C29	-85.7(2)
N1	Ni1	C28	C29	96.7(2)
C33	C28	C29	C30	0.2(4)
Ni1	C28	C29	C30	178.8(2)
C28	C29	C30	C31	0.4(4)
C29	C30	C31	C32	-0.6(4)
C29	C30	C31	C34	-179.7(3)
C30	C31	C32	C33	0.1(5)
C34	C31	C32	C33	179.2(3)
C31	C32	C33	C28	0.5(5)
C29	C28	C33	C32	-0.7(4)
Ni1	C28	C33	C32	-179.4(3)
C32	C31	C34	F2	160.3(3)
C30	C31	C34	F2	-20.7(4)
C32	C31	C34	F1	39.5(4)
C30	C31	C34	F1	-141.4(3)
C32	C31	C34	F3	-78.7(4)
C30	C31	C34	F3	100.4(4)

Symmetry operations

1 'x, y, z'
2 '-x, y, -z+1/2'
3 'x+1/2, y+1/2, z'
4 '-x+1/2, y+1/2, -z+1/2'
5 '-x, -y, -z'
6 'x, -y, z-1/2'
7 '-x+1/2, -y+1/2, -z'
8 'x+1/2, -y+1/2, z-1/2'

8. NMR Spectra

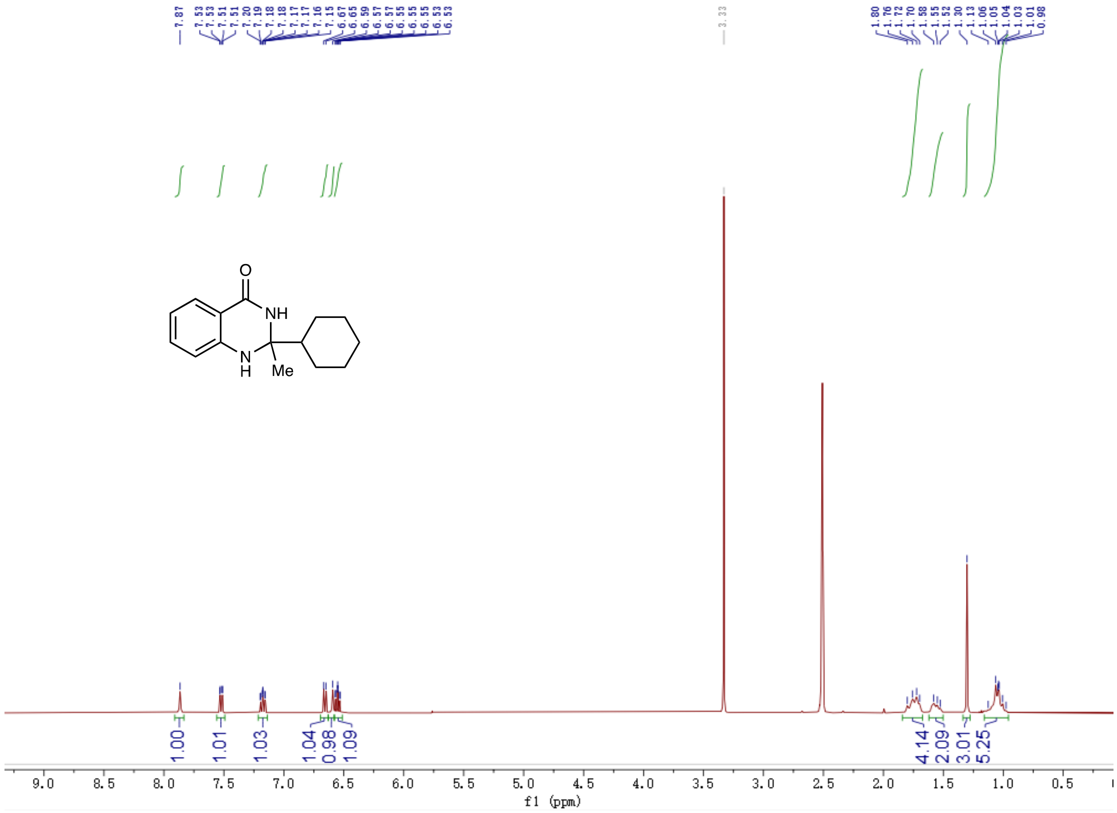

Supplementary Figure 35. ^{1}H NMR spectra of compound **2a**

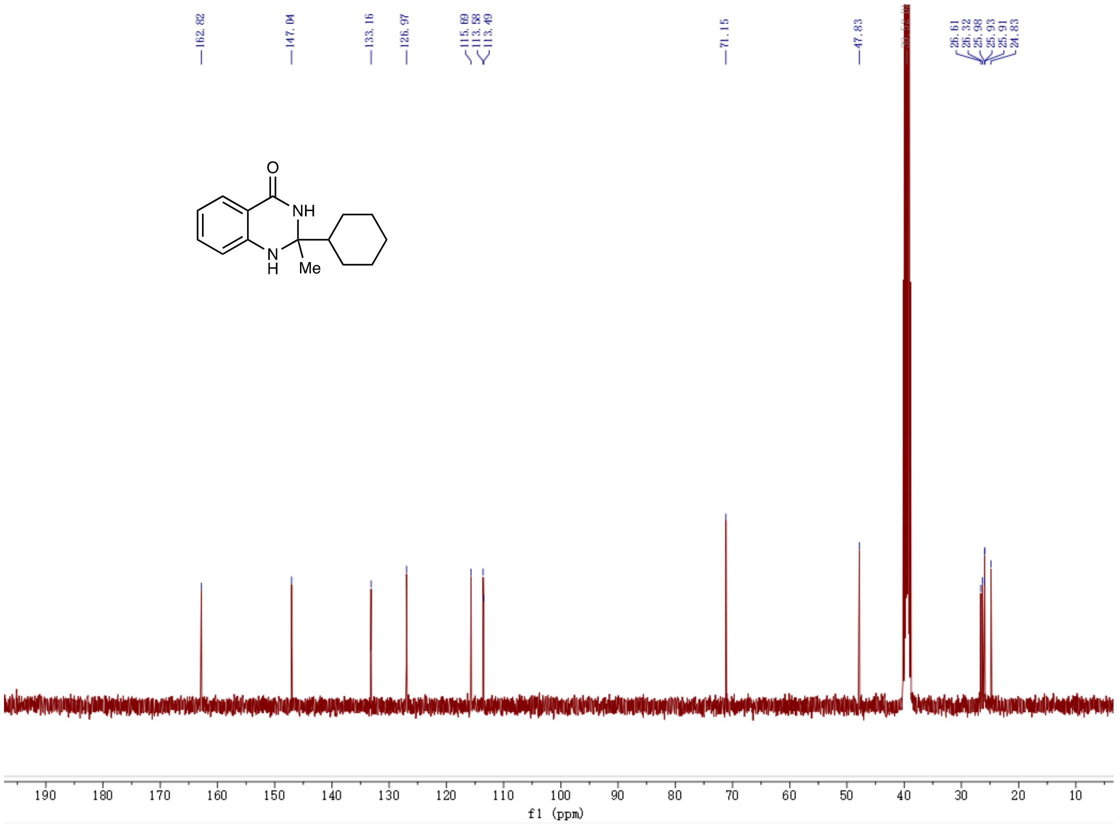

Supplementary Figure 36. ^{13}C NMR spectra of compound **2a**

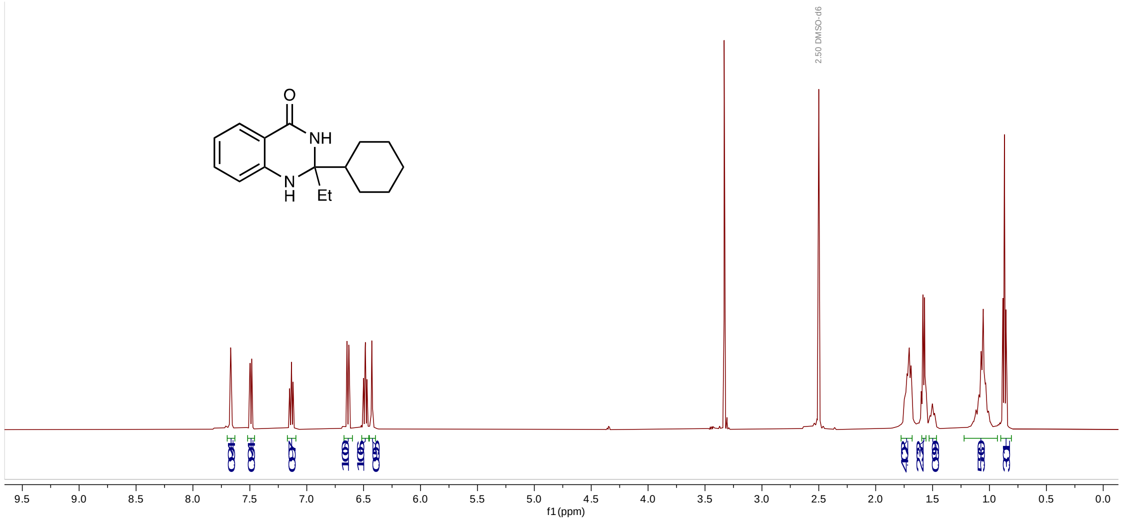

Supplementary Figure 37. ^{1}H NMR spectra of compound **2a'**

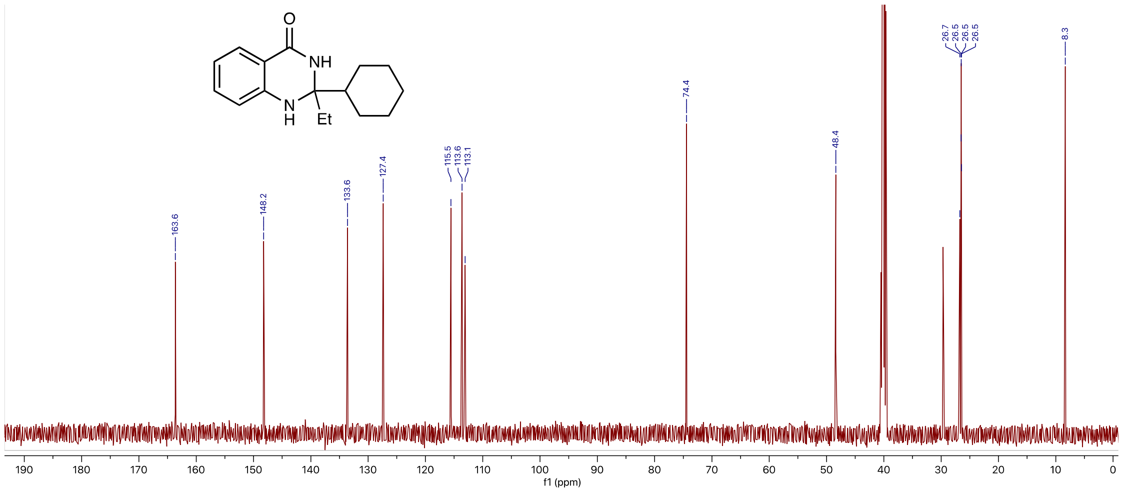

Supplementary Figure 38. ^{13}C NMR spectra of compound **2a'**

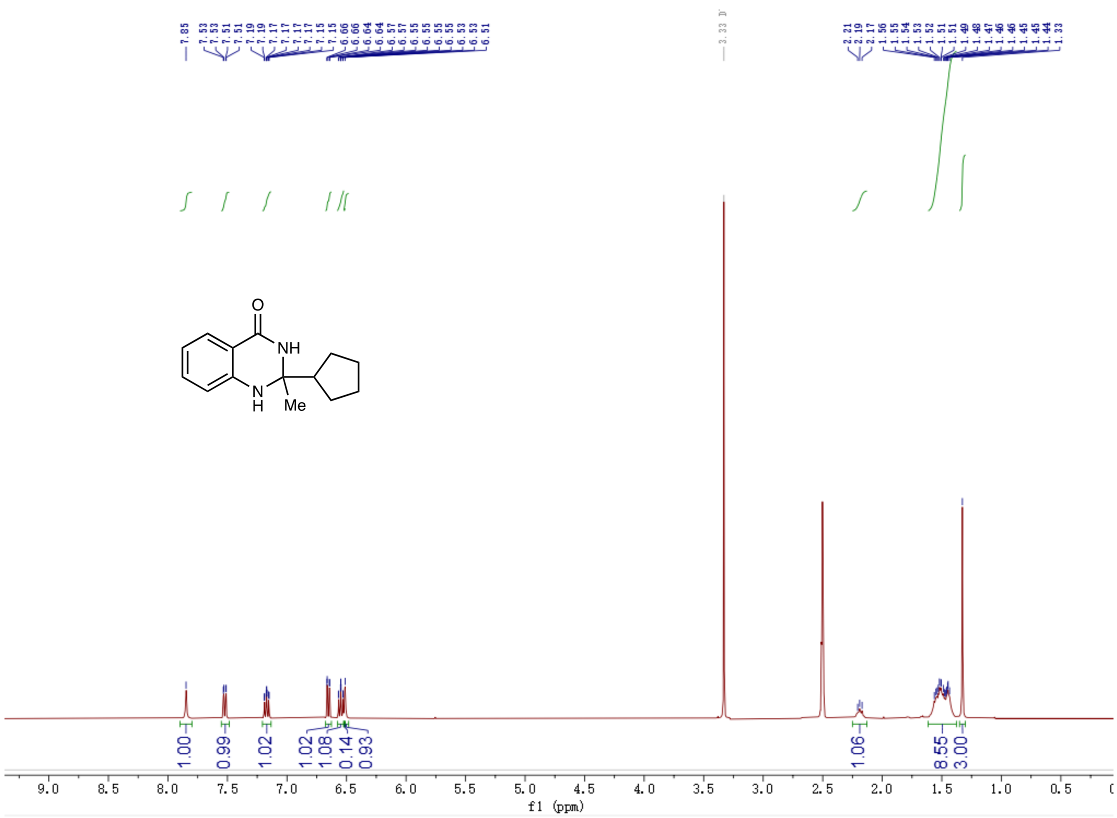

Supplementary Figure 39. ^{1}H NMR spectra of compound **2b**

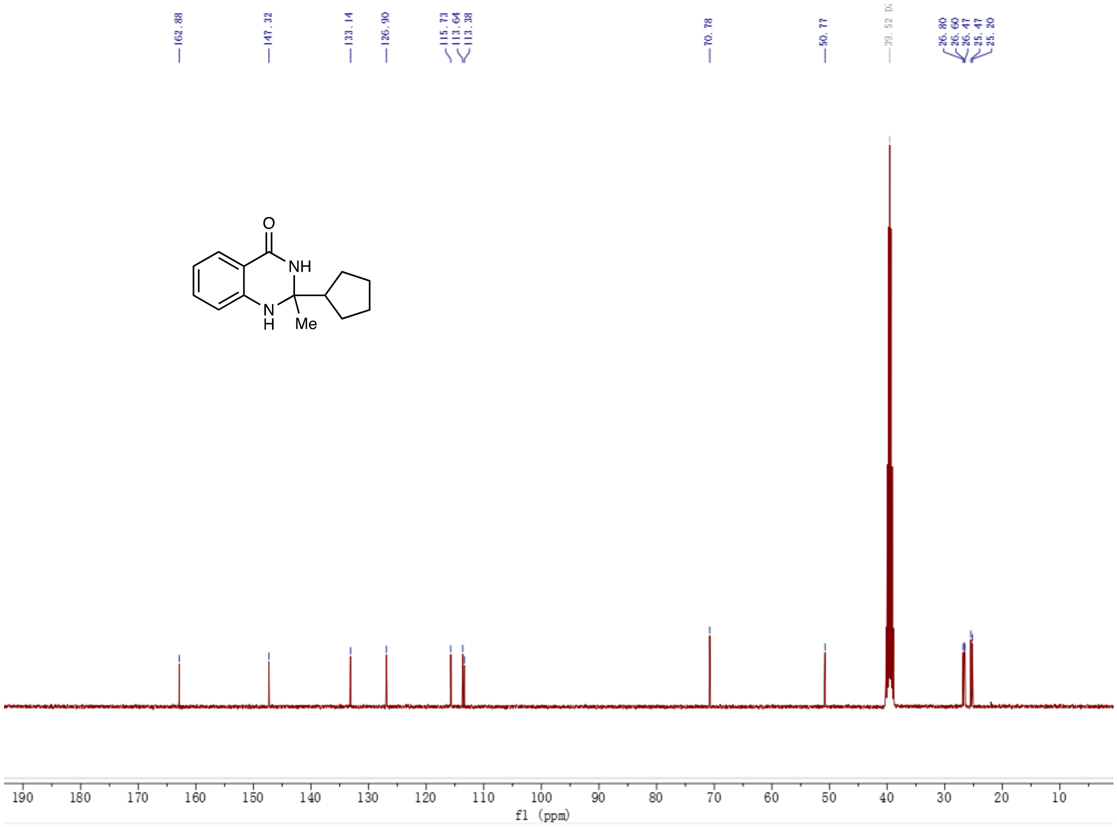

Supplementary Figure 40. ^{13}C NMR spectra of compound **2b**

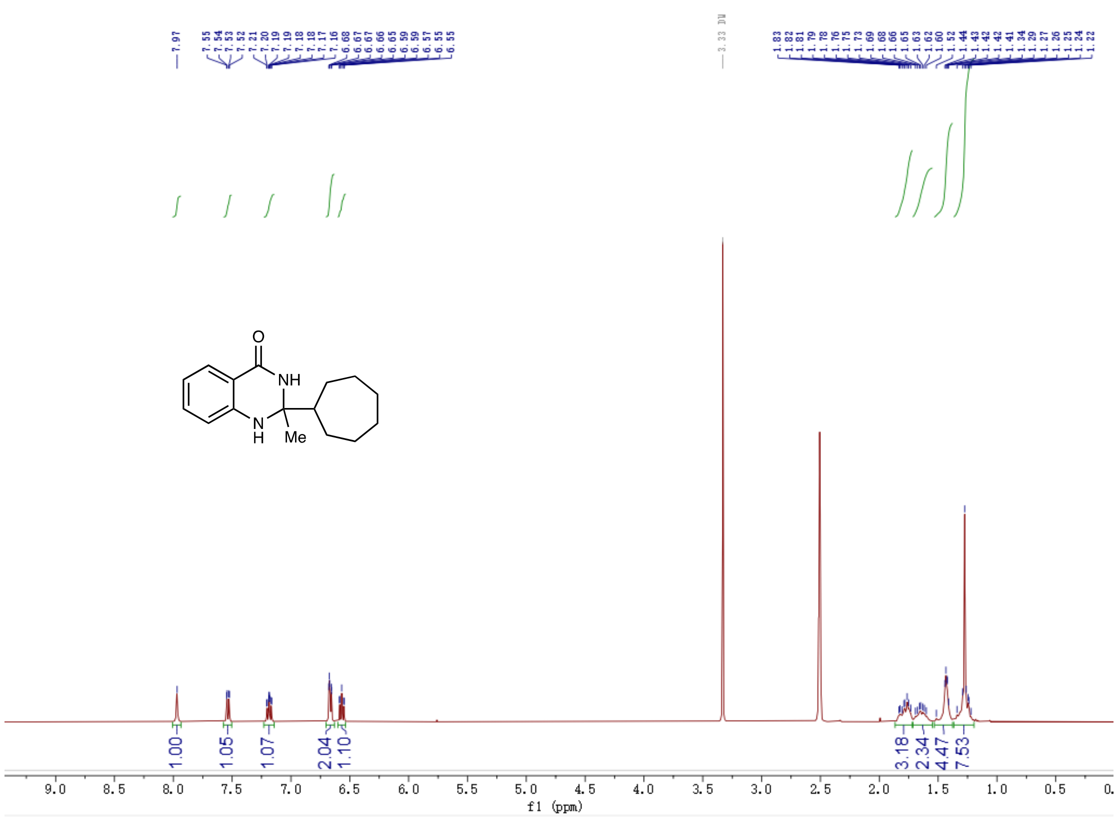

Supplementary Figure 41. ^{1}H NMR spectra of compound **2c**

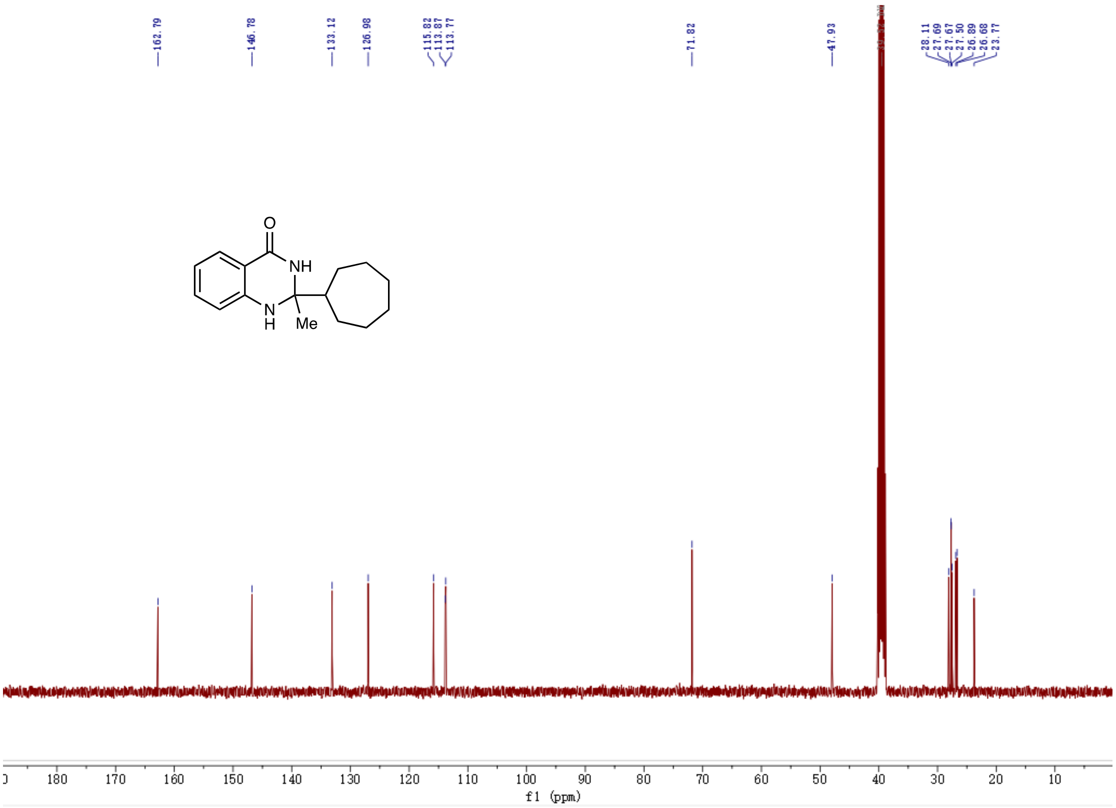

Supplementary Figure 42. ^{13}C NMR spectra of compound **2c**

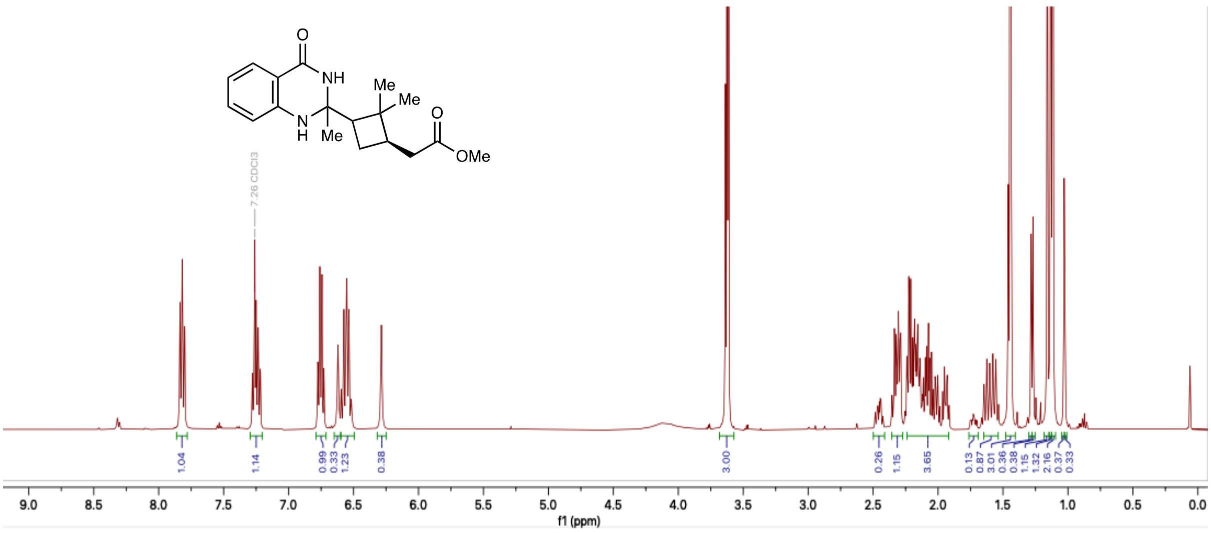

Supplementary Figure 43. ^{1}H NMR spectra of compound **2d**

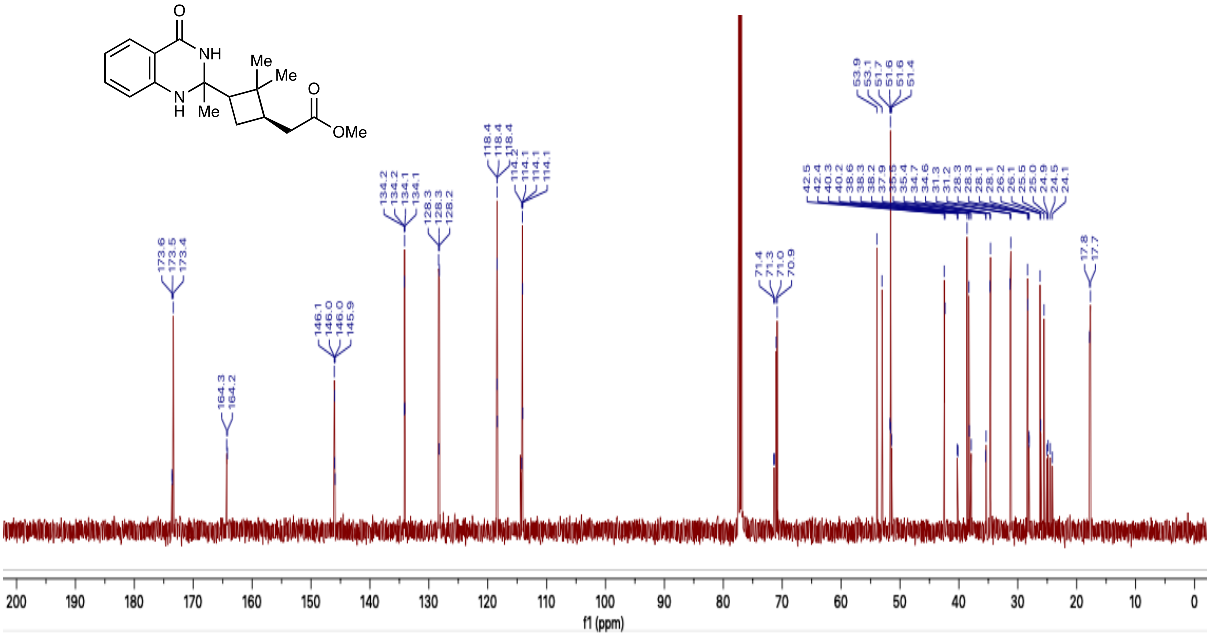

Supplementary Figure 44. ^{13}C NMR spectra of compound **2d**

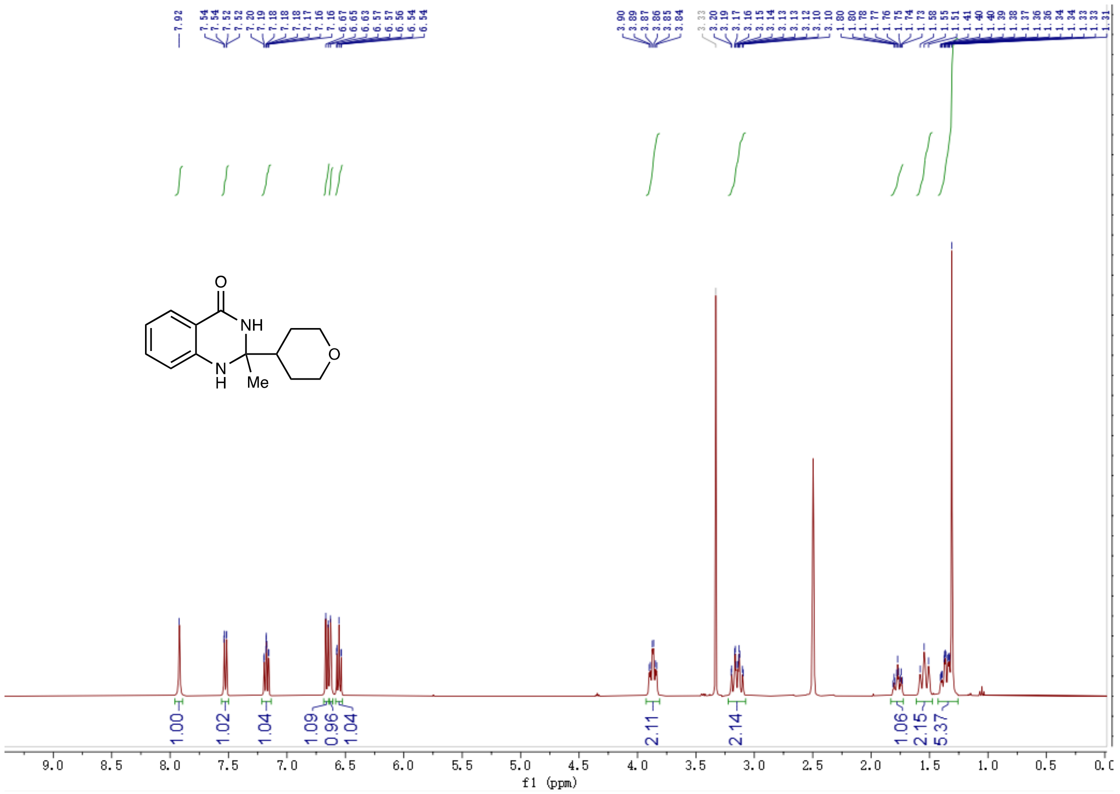

Supplementary Figure 45. ^{1}H NMR spectra of compound **2e**

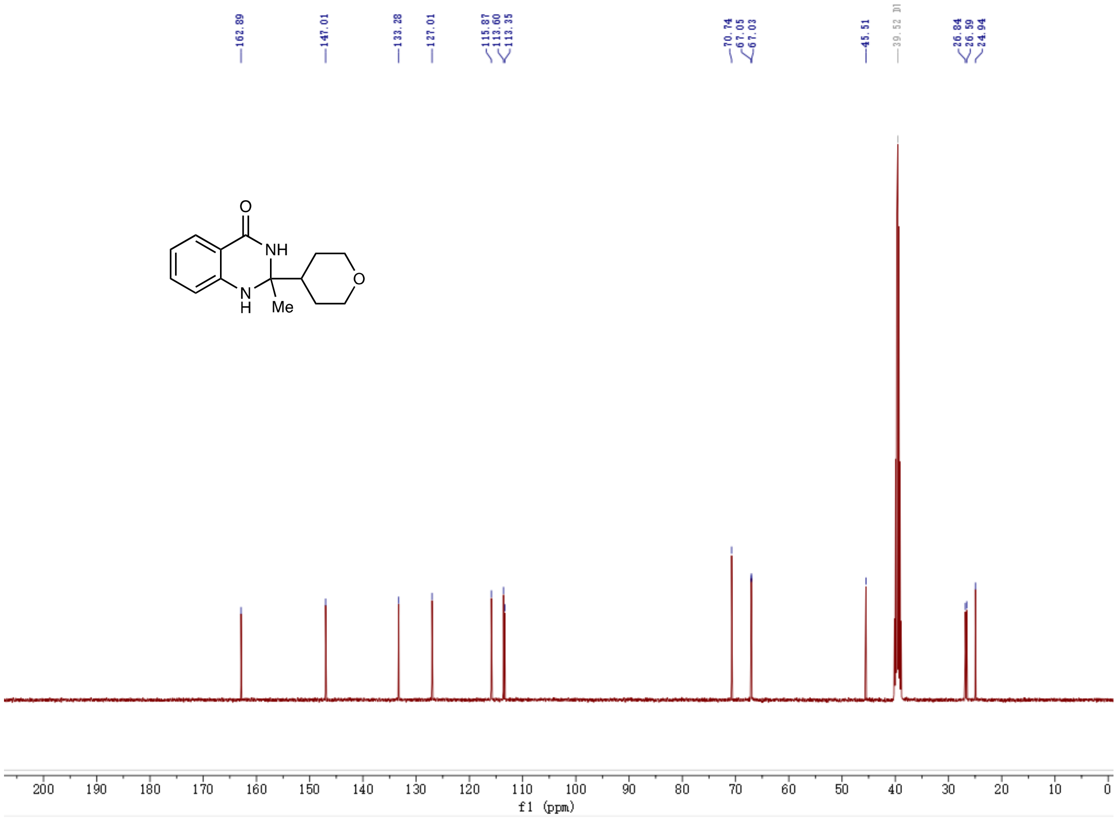

Supplementary Figure 46. ^{13}C NMR spectra of compound **2e**

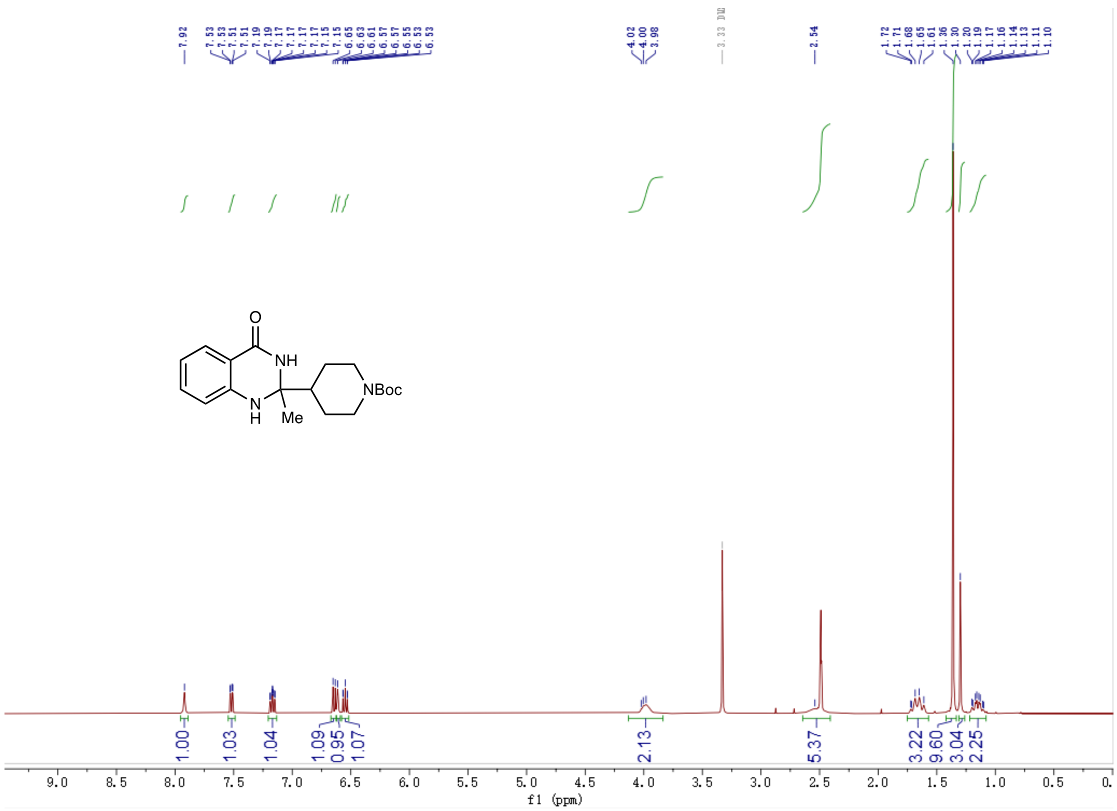

Supplementary Figure 47. ^{1}H NMR spectra of compound**2f**

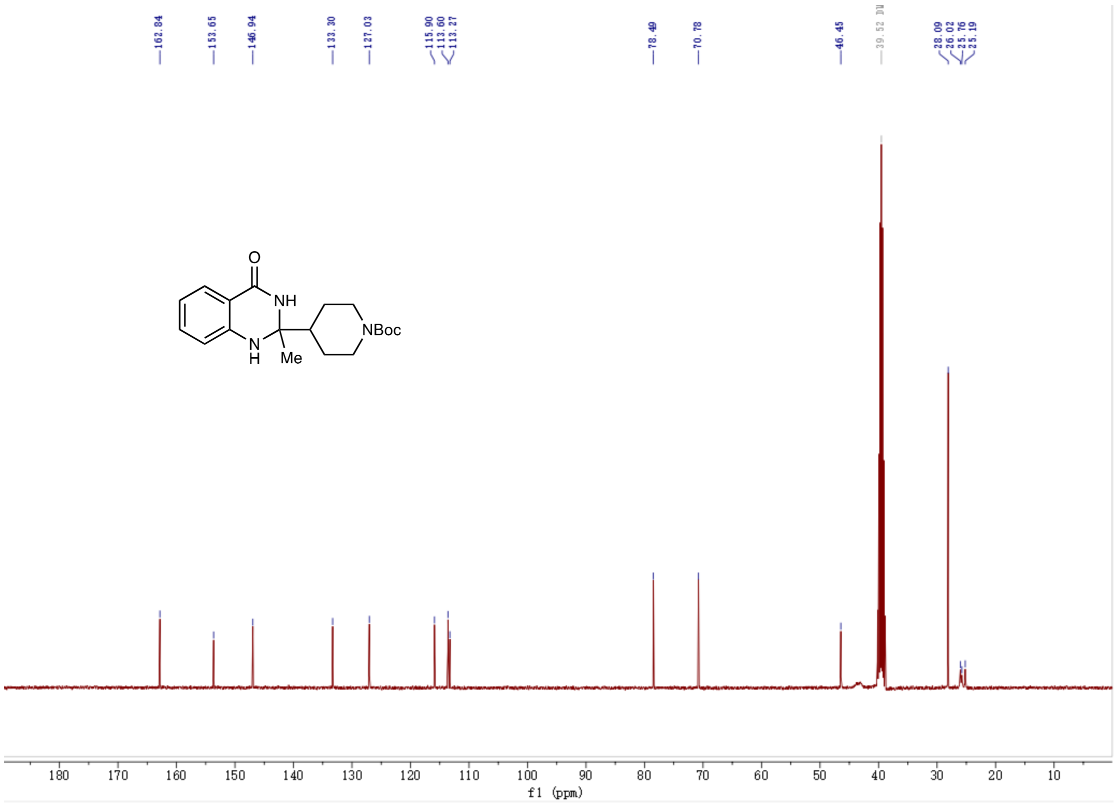

Supplementary Figure 48. ^{13}C NMR spectra of compound **2f**

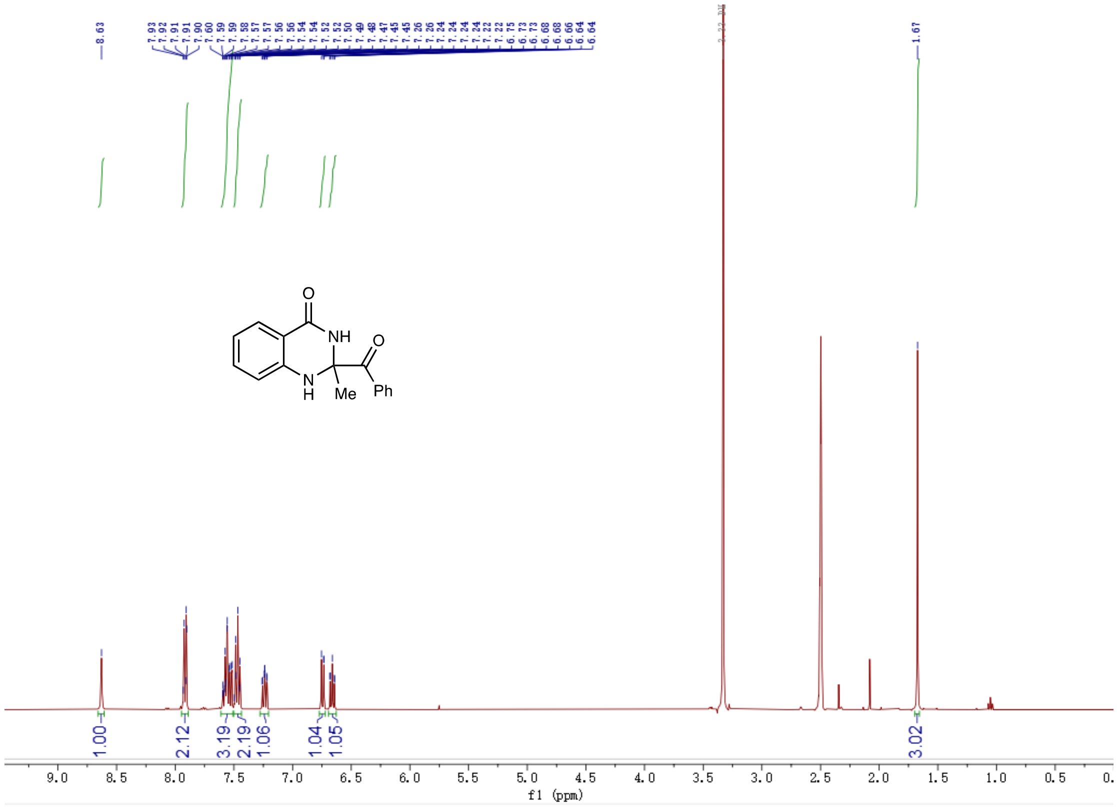

Supplementary Figure 49. ^{1}H NMR spectra of compound **2g**

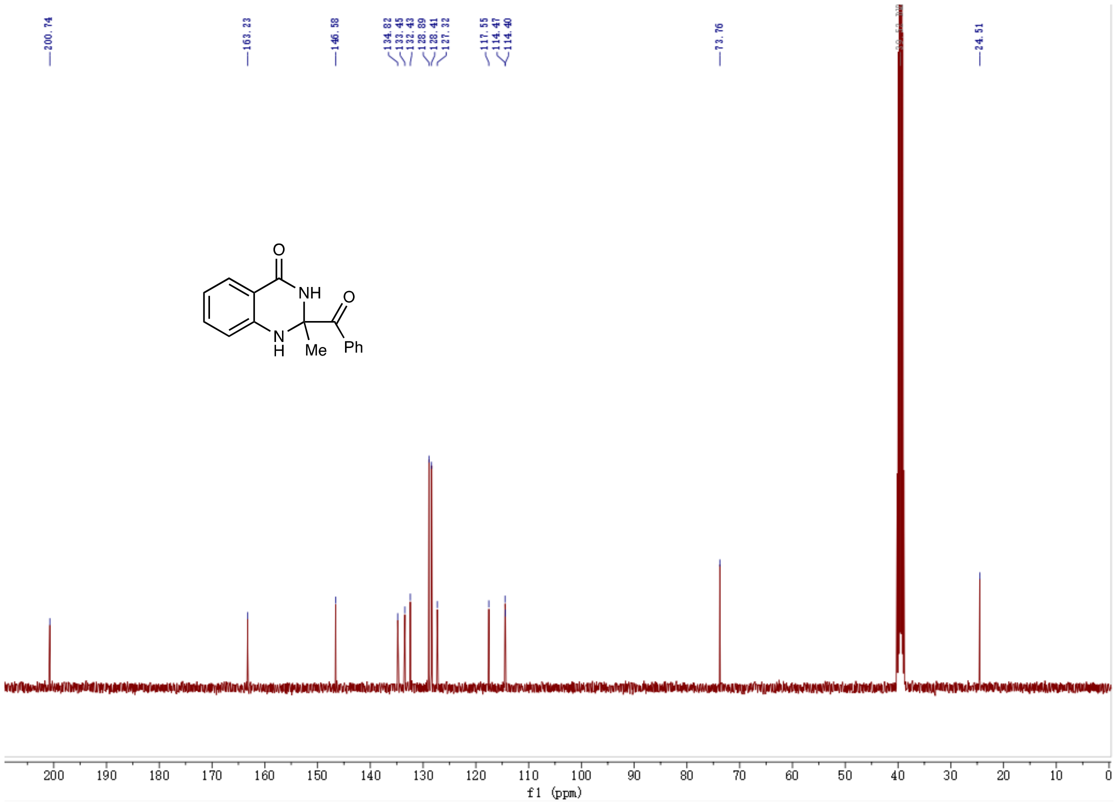

Supplementary Figure 50. ^{13}C NMR spectra of compound **2g**

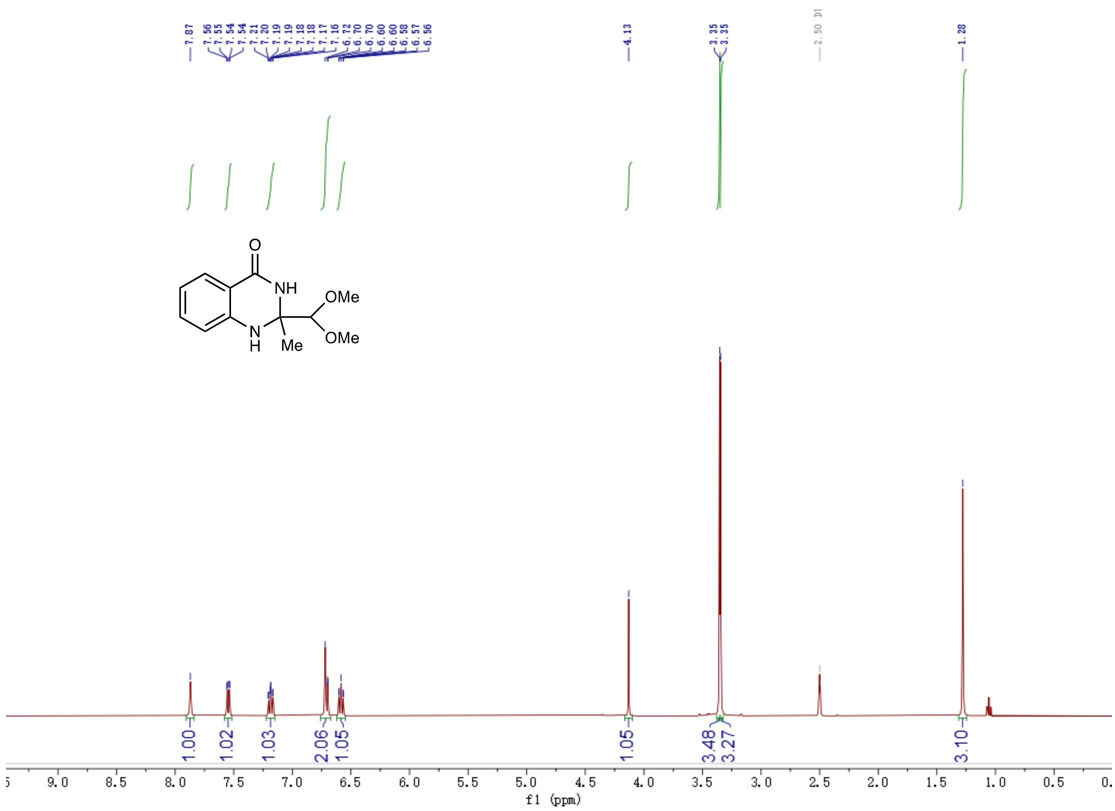

Supplementary Figure 51. ^{1}H NMR spectra of compound **2h**

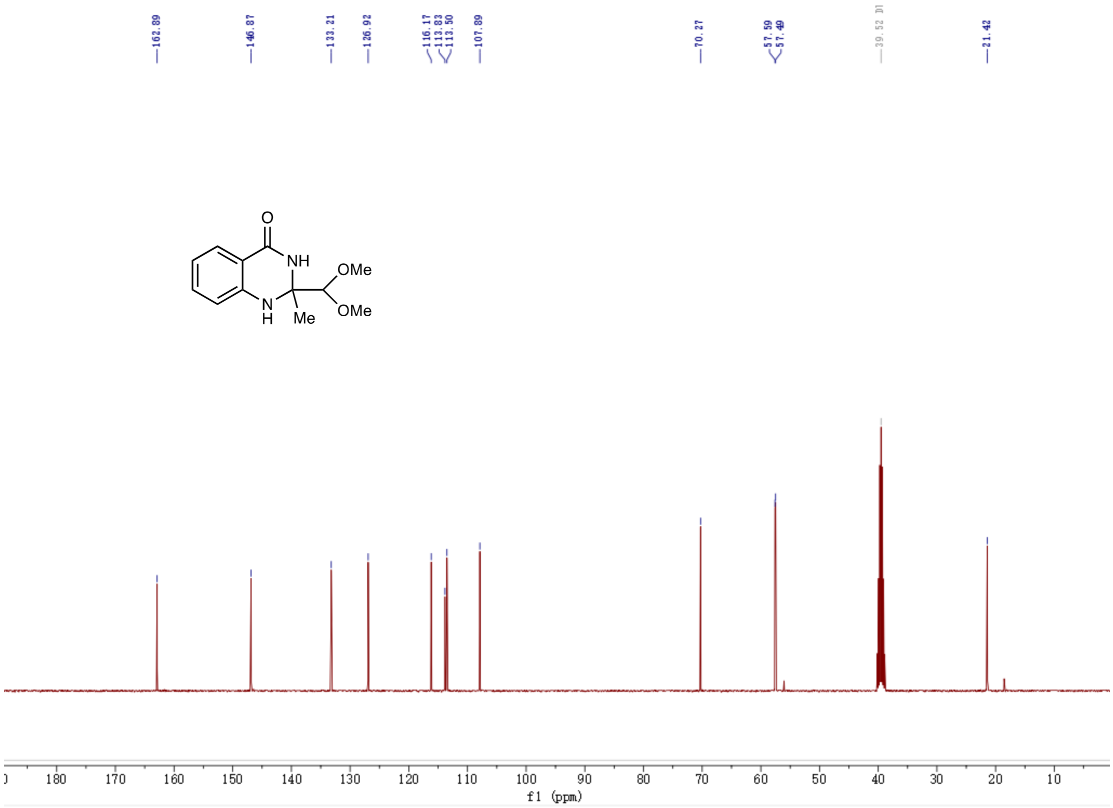

Supplementary Figure 52. ^{13}C NMR spectra of compound **2h**

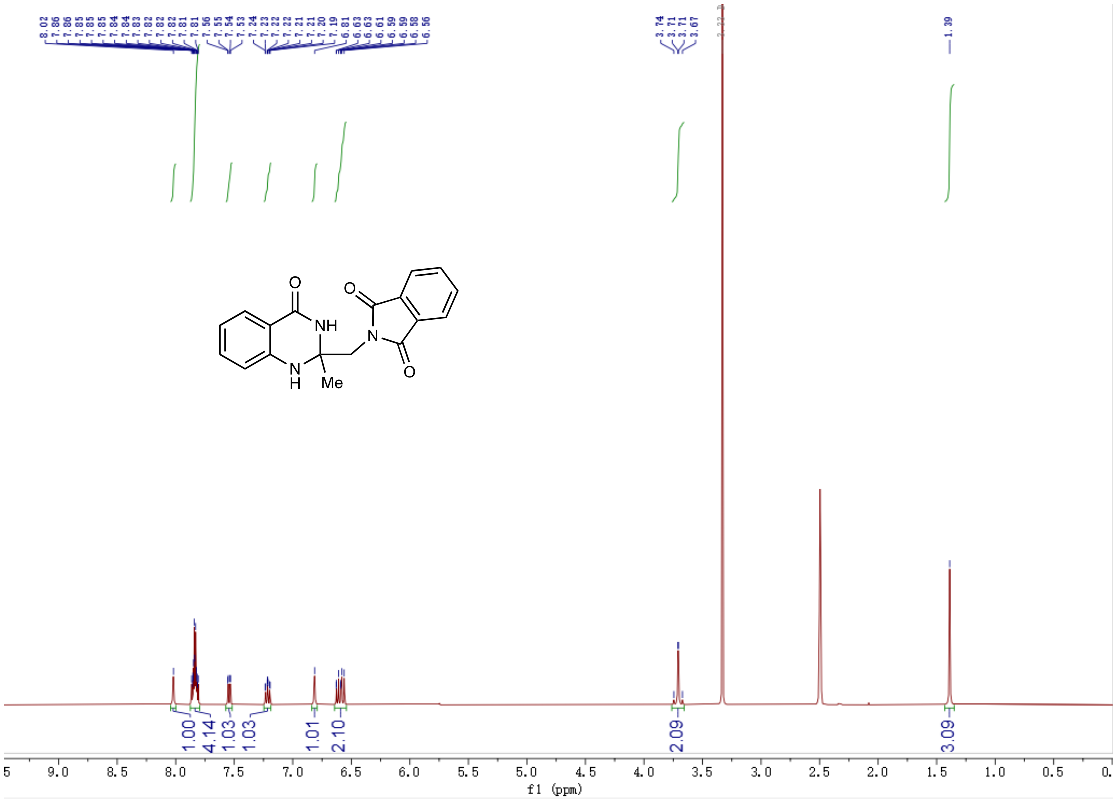

Supplementary Figure 53. ^{1}H NMR spectra of compound **2i**

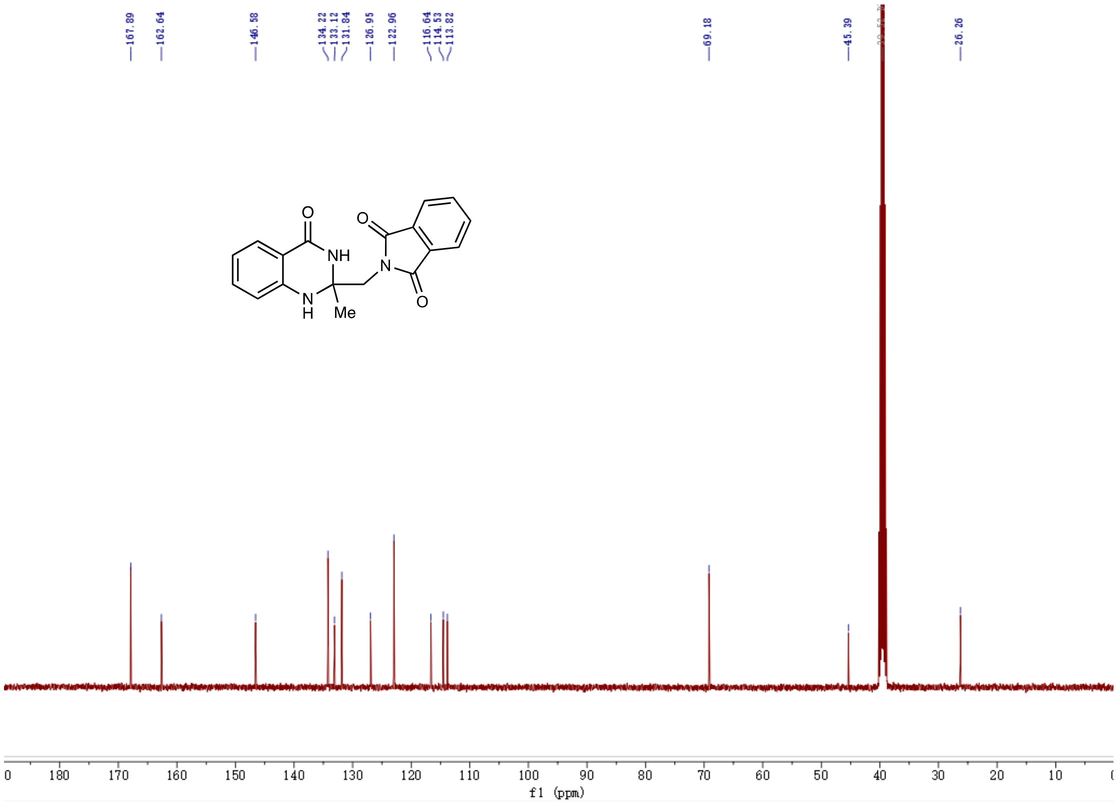

Supplementary Figure 54. ^{13}C NMR spectra of compound **2i**

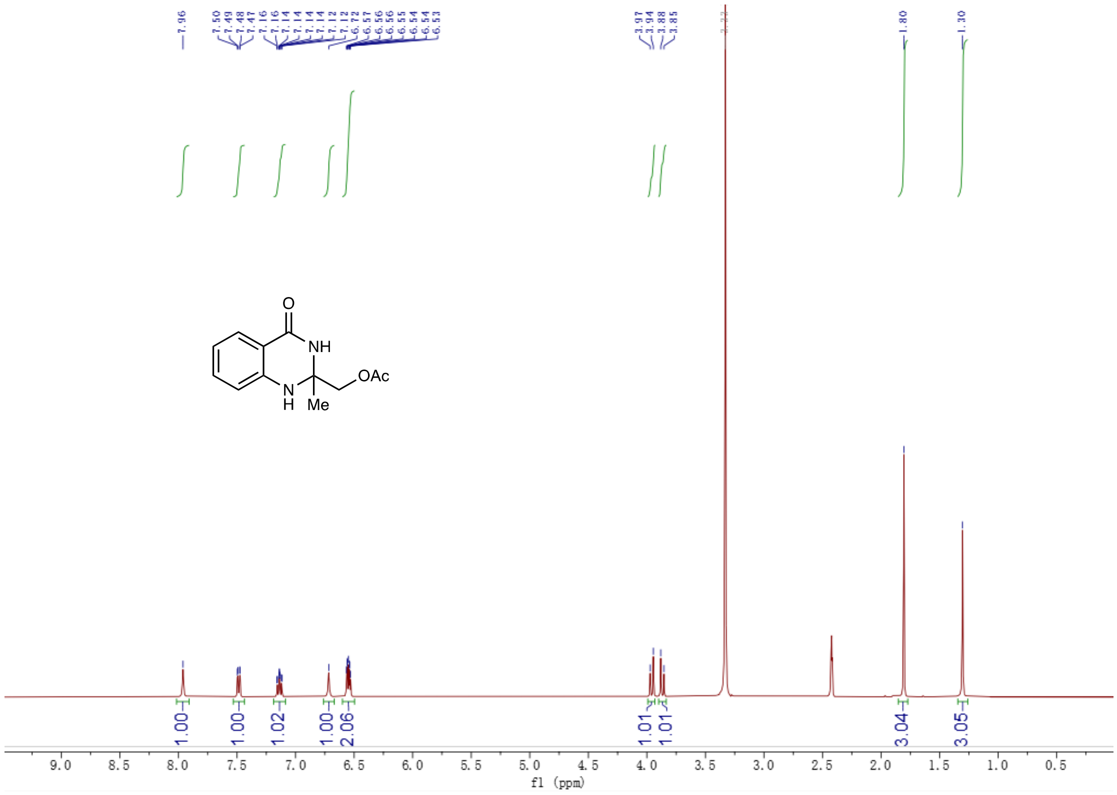

Supplementary Figure 55. ^{1}H NMR spectra of compound **2j**

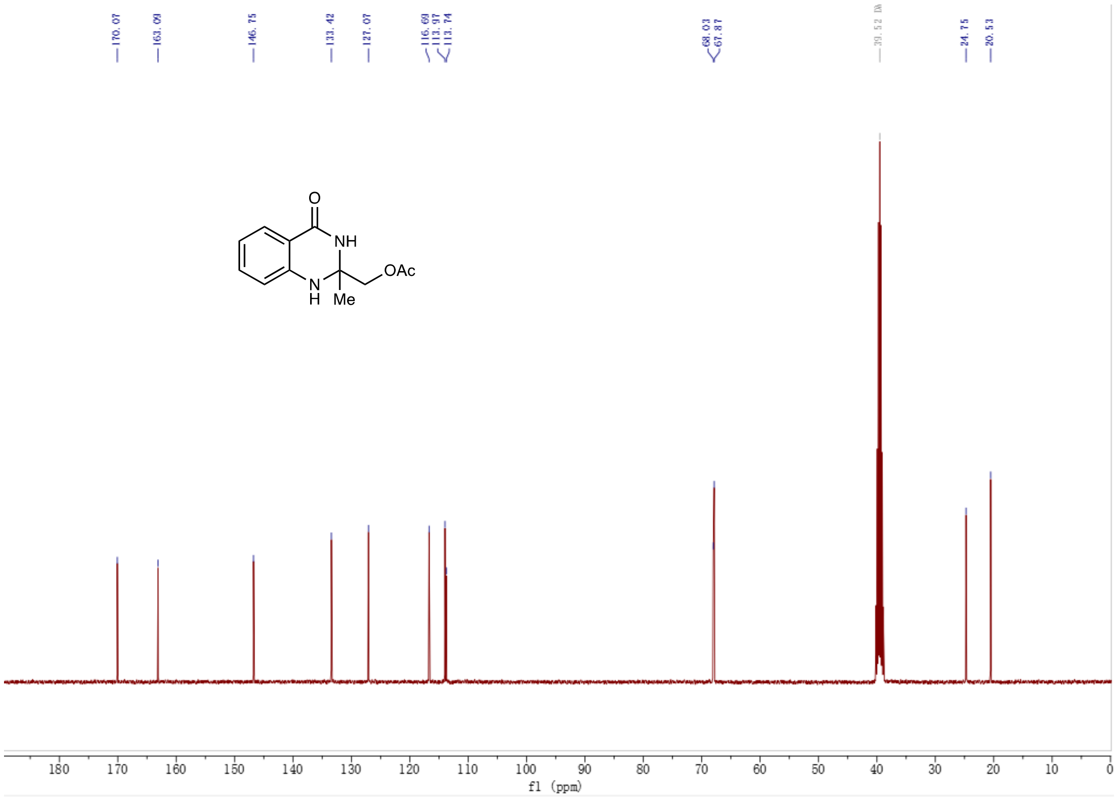

Supplementary Figure 56. ^{13}C NMR spectra of compound **2j**

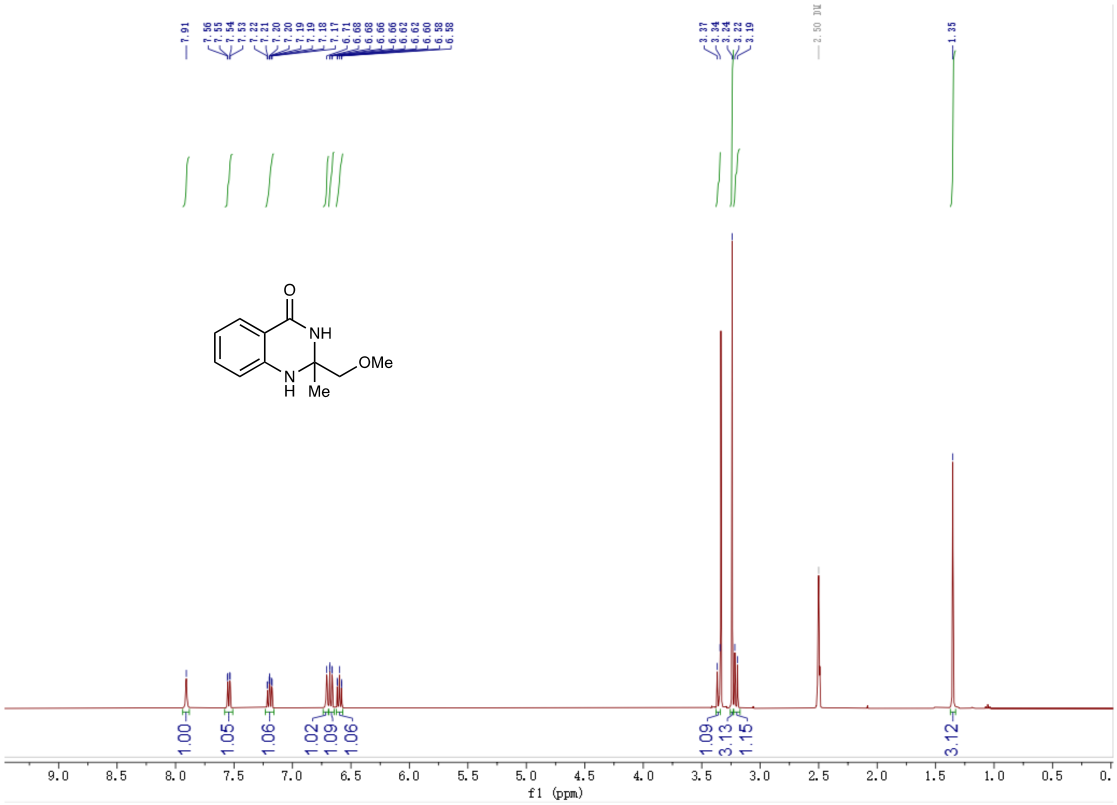

Supplementary Figure 57. ^{1}H NMR spectra of compound **2k**

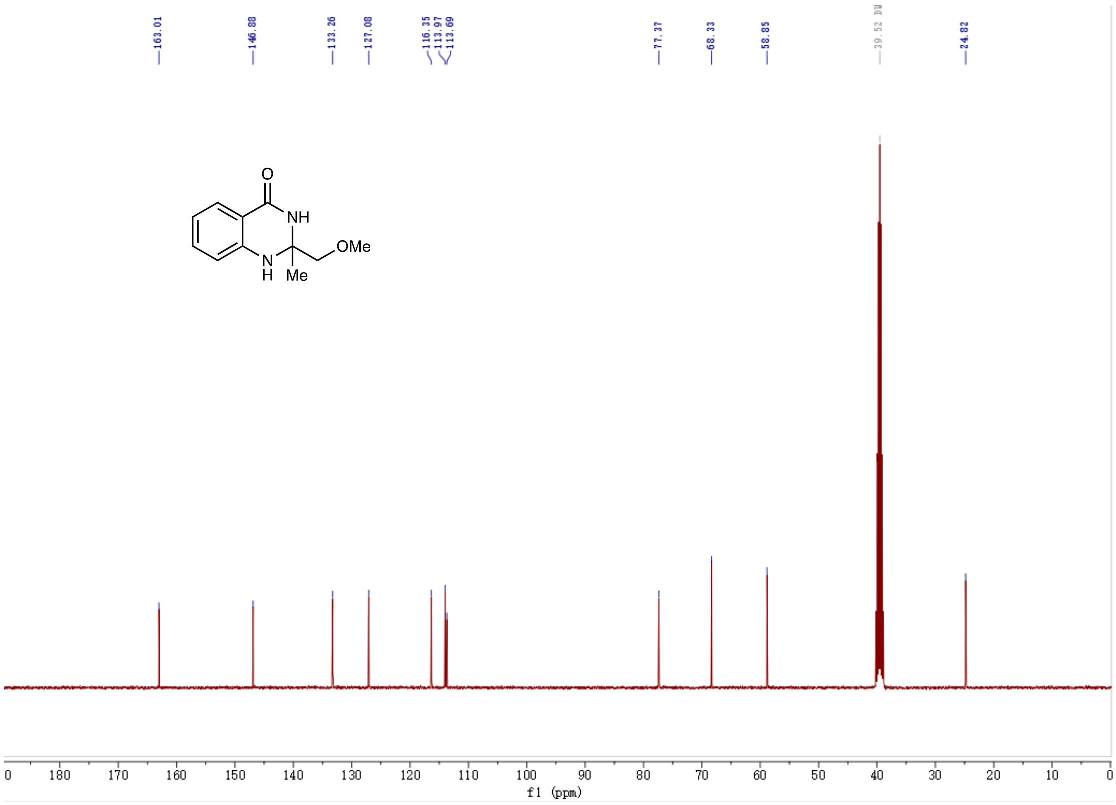

Supplementary Figure 58. ^{13}C NMR spectra of compound **2k**

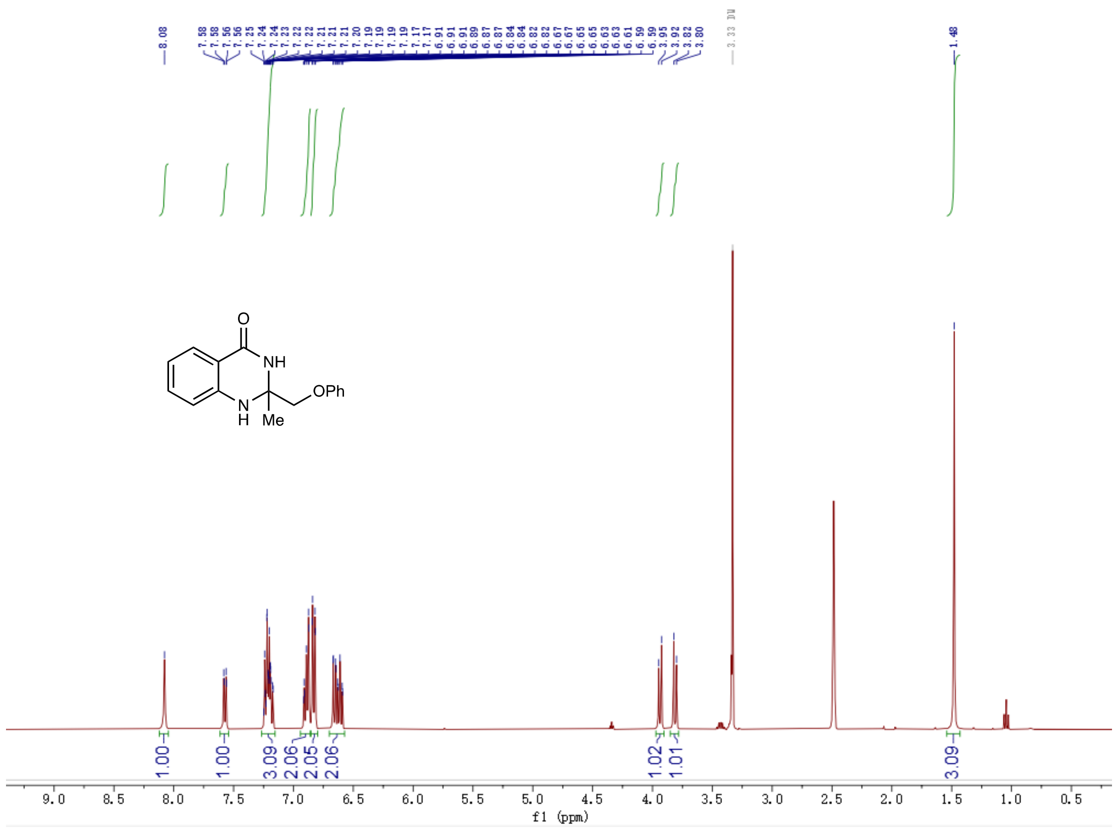

Supplementary Figure 59. ^{1}H NMR spectra of compound **2l**

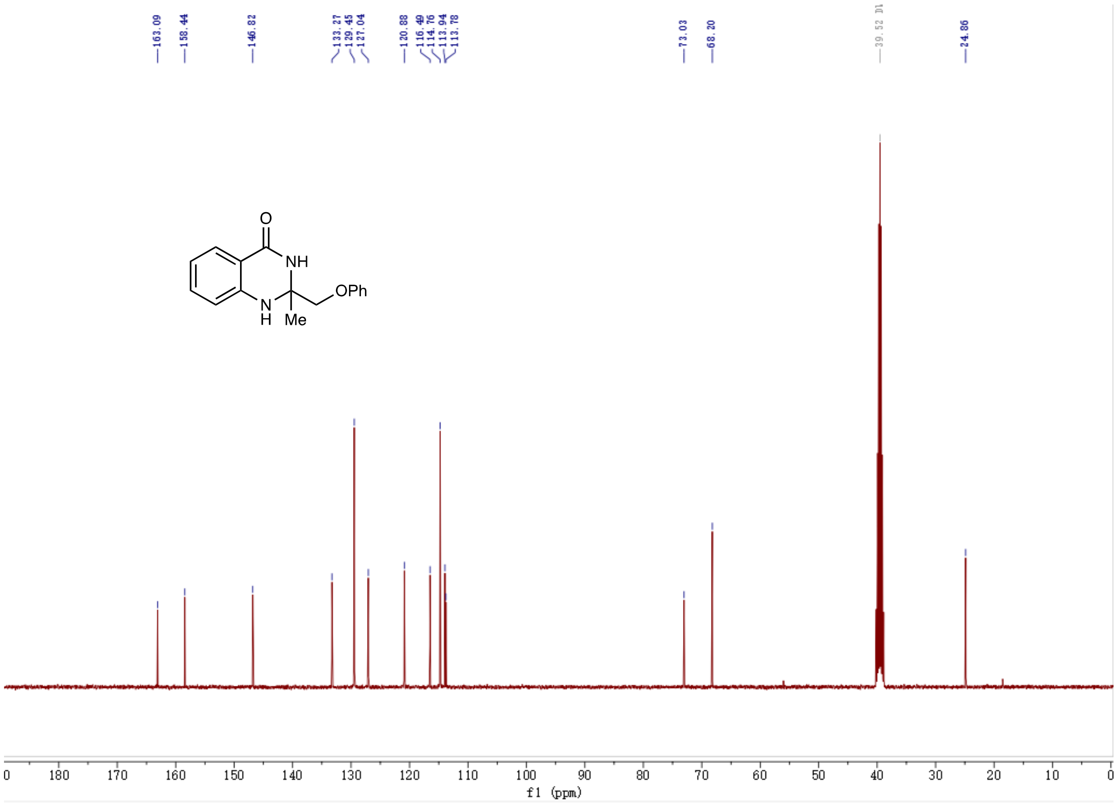

Supplementary Figure 60. ^{13}C NMR spectra of compound **2l**

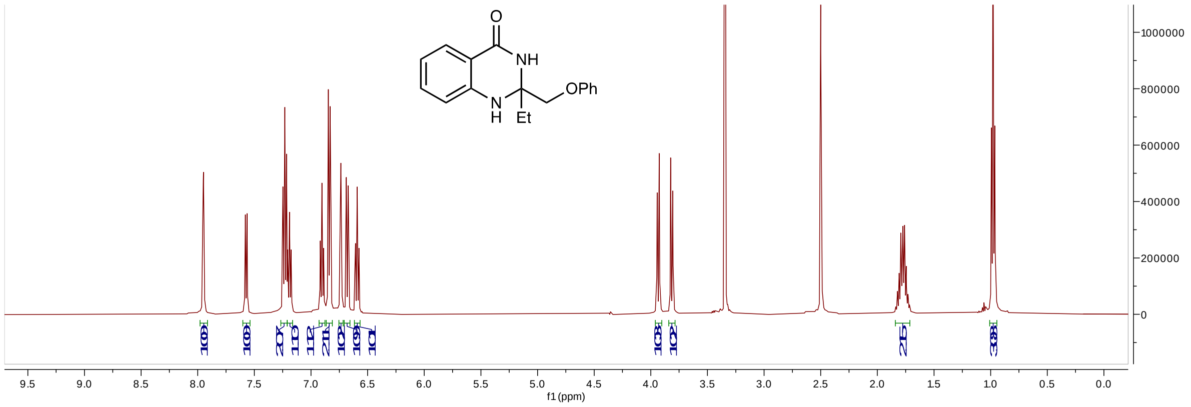

Supplementary Figure 61. ^{1}H NMR spectra of compound **2l’**

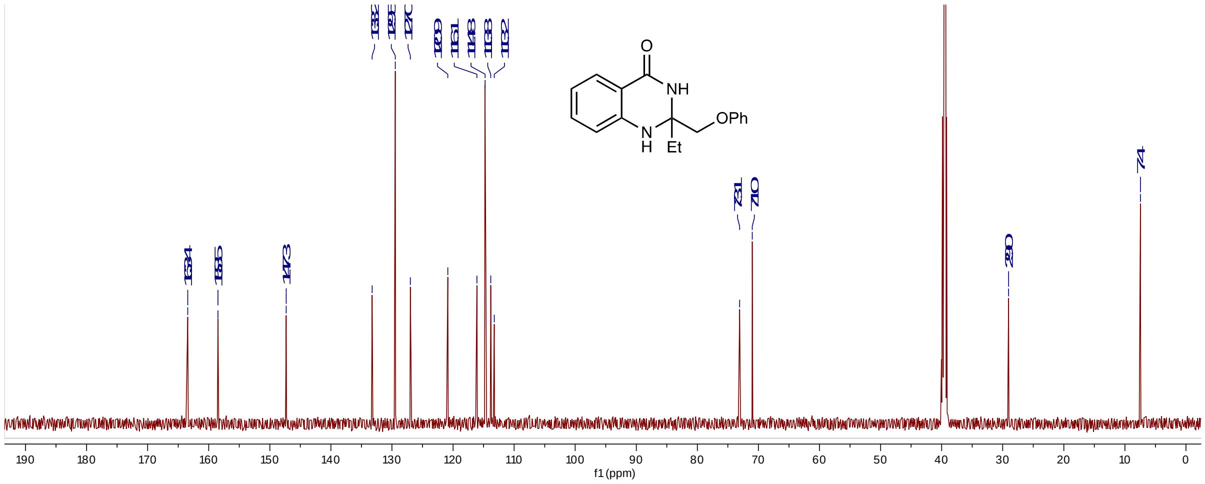

Supplementary Figure 62. ^{13}C NMR spectra of compound **2l’**

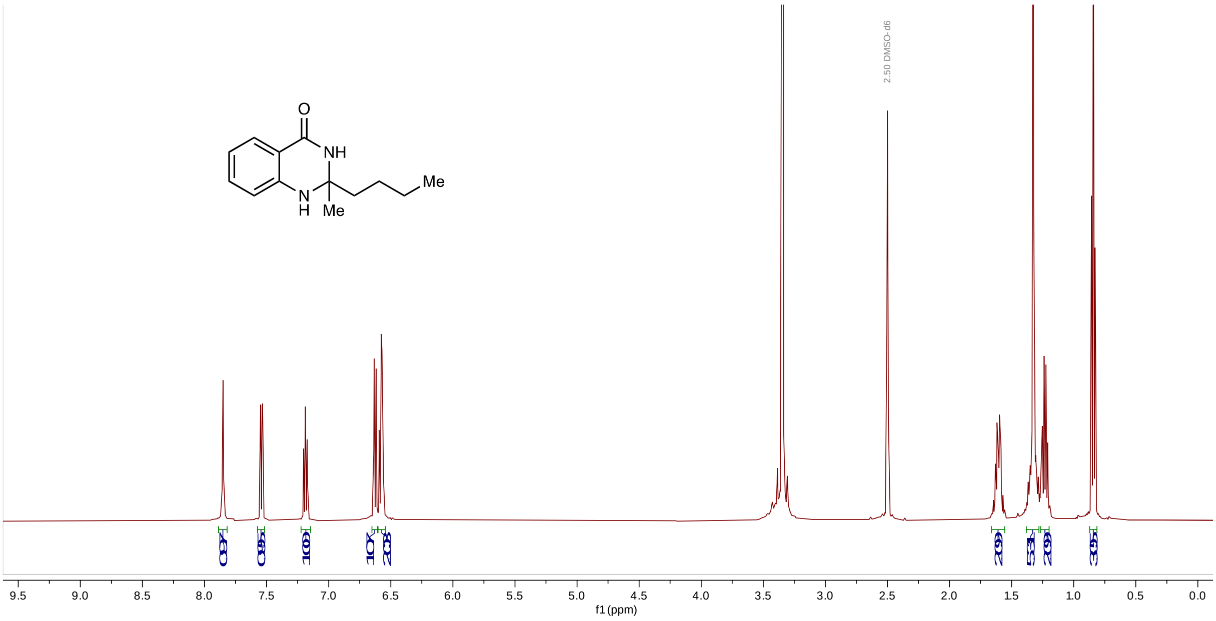

Supplementary Figure 63. ^{1}H NMR spectra of compound **2m**

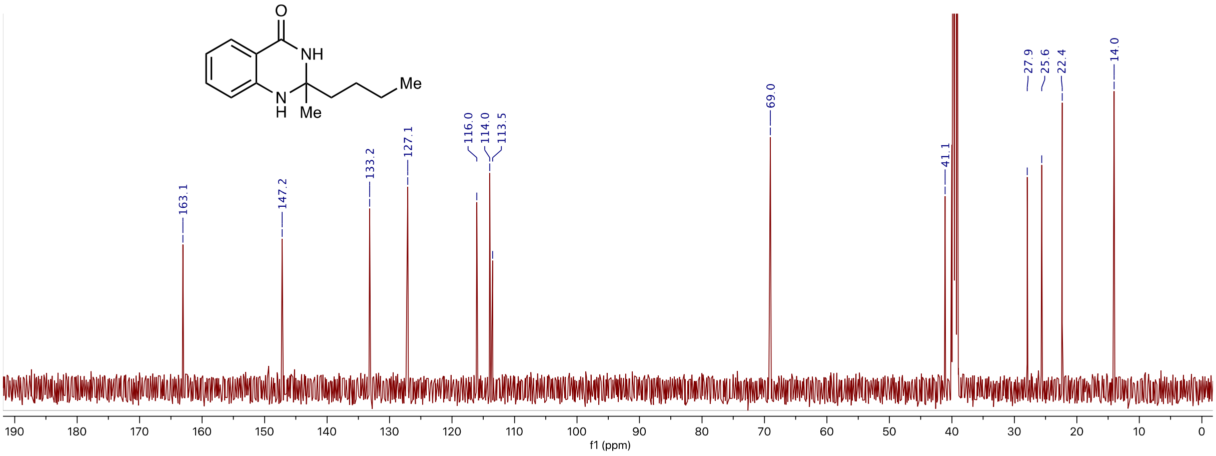

Supplementary Figure 64. ^{13}C NMR spectra of compound **2m**

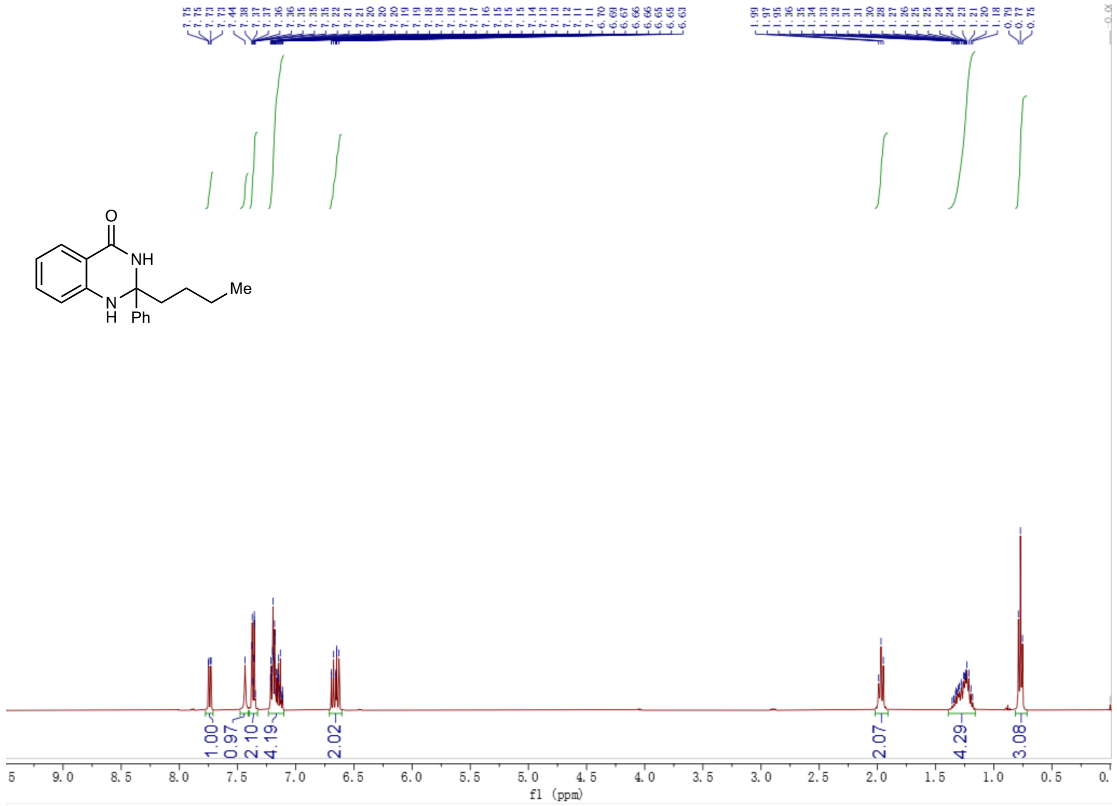

Supplementary Figure 65. ^{1}H NMR spectra of compound **2m'**

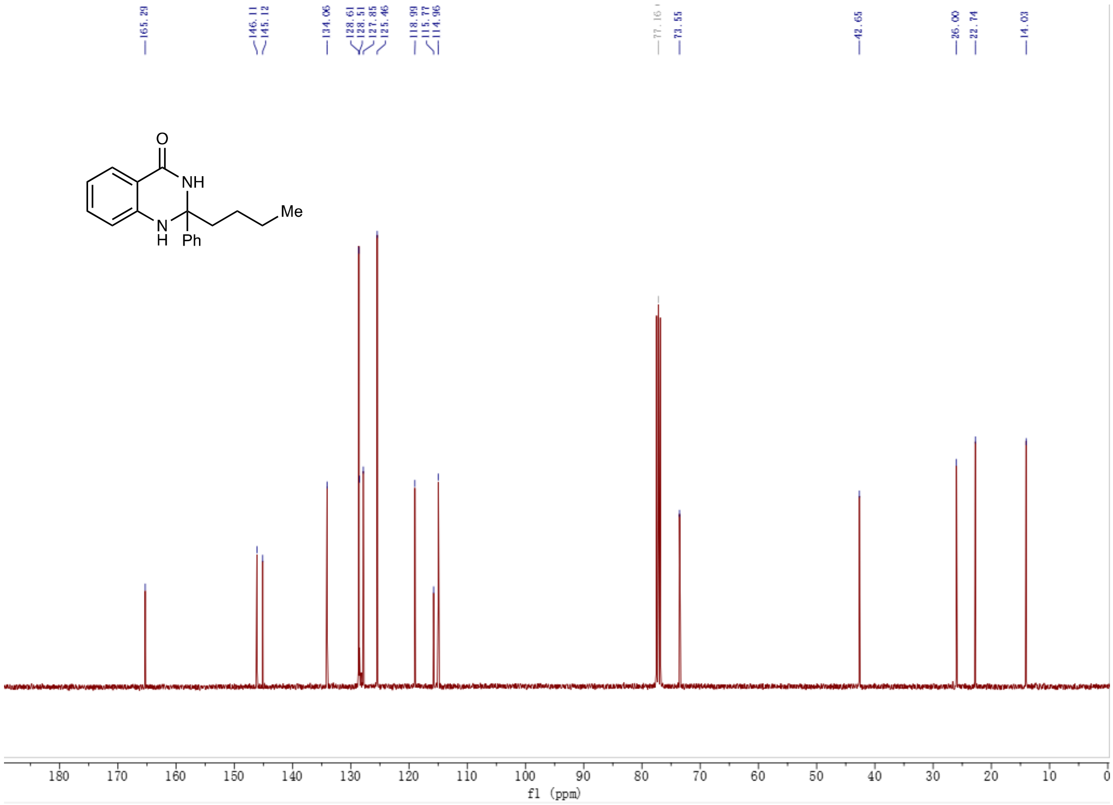

Supplementary Figure 66. ^{13}C NMR spectra of compound **2m'**

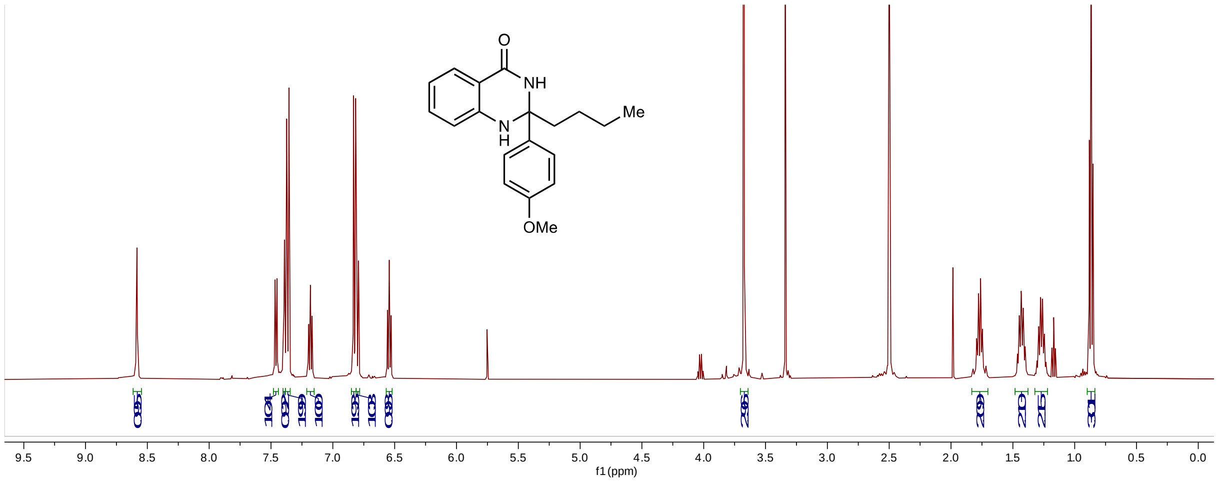

Supplementary Figure 67. ^{1}H NMR spectra of compound **2m''**

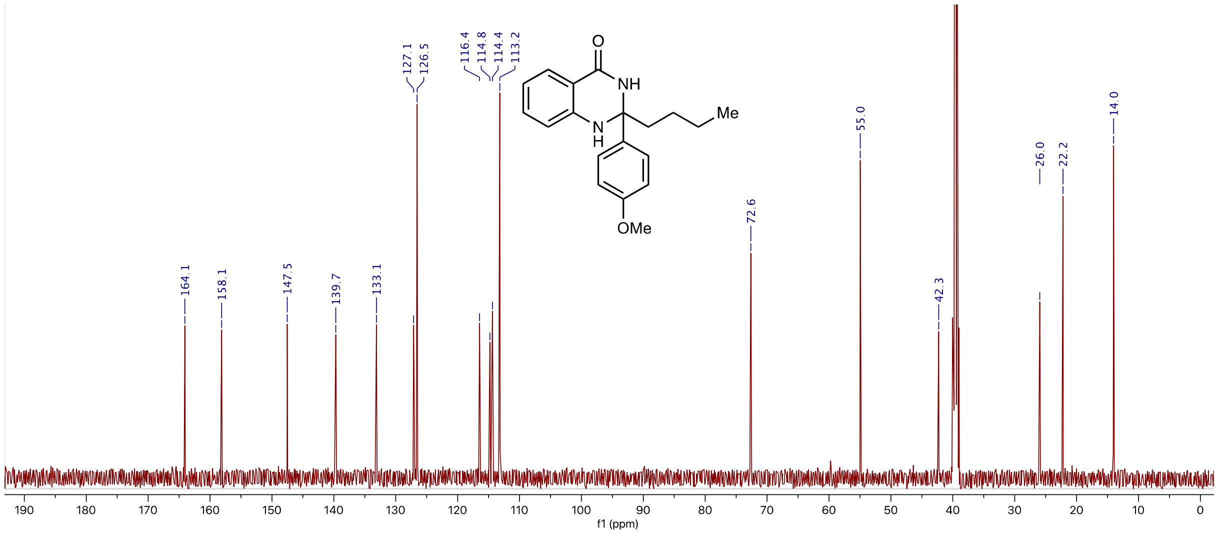

Supplementary Figure 68. ^{13}C NMR spectra of compound **2m''**

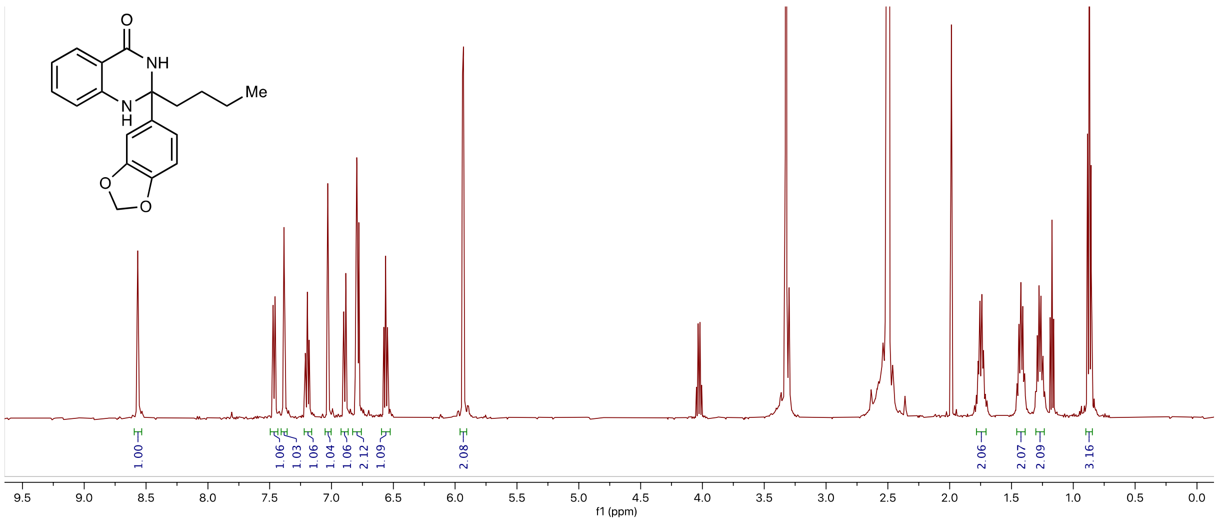

Supplementary Figure 69. ^{1}H NMR spectra of compound **2m’’’**

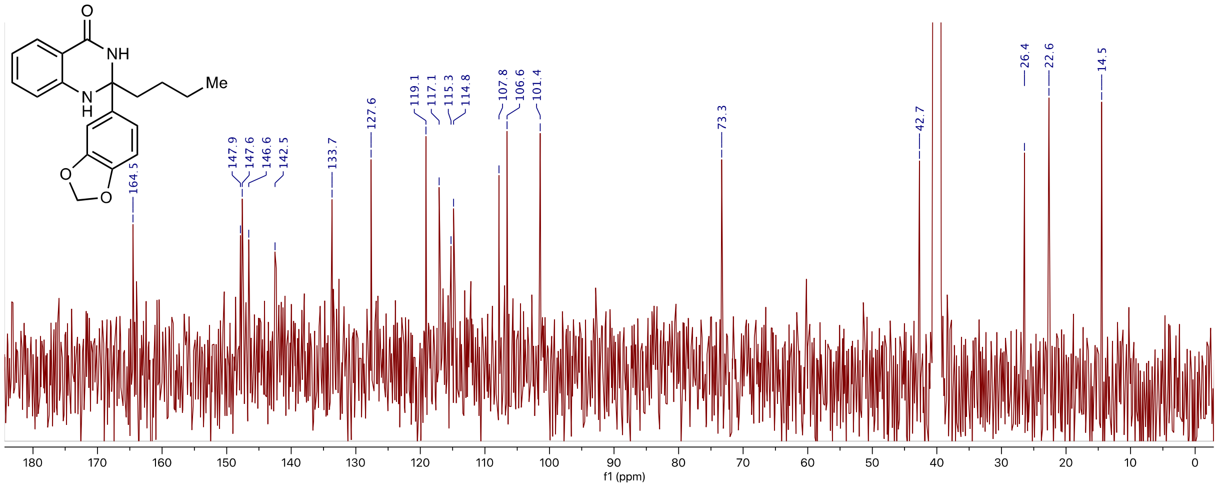

Supplementary Figure 70. ^{13}C NMR spectra of compound **2m’’’**

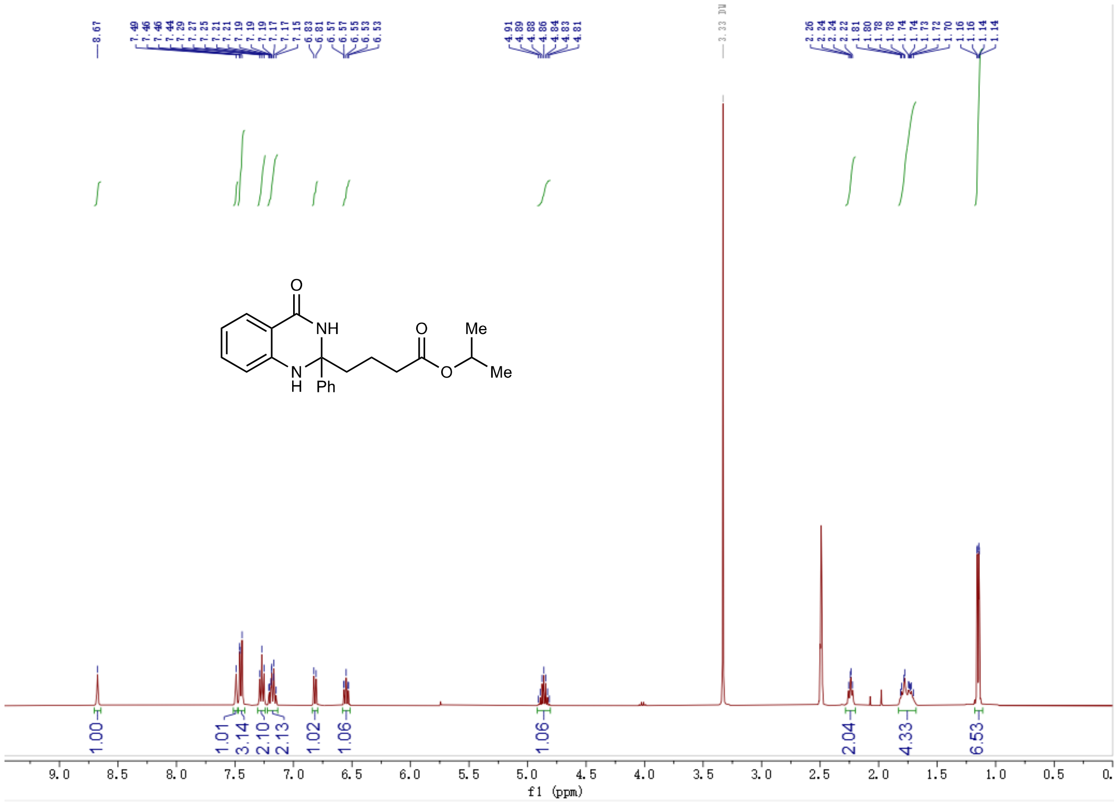

Supplementary Figure 71. ^{1}H NMR spectra of compound **2n**

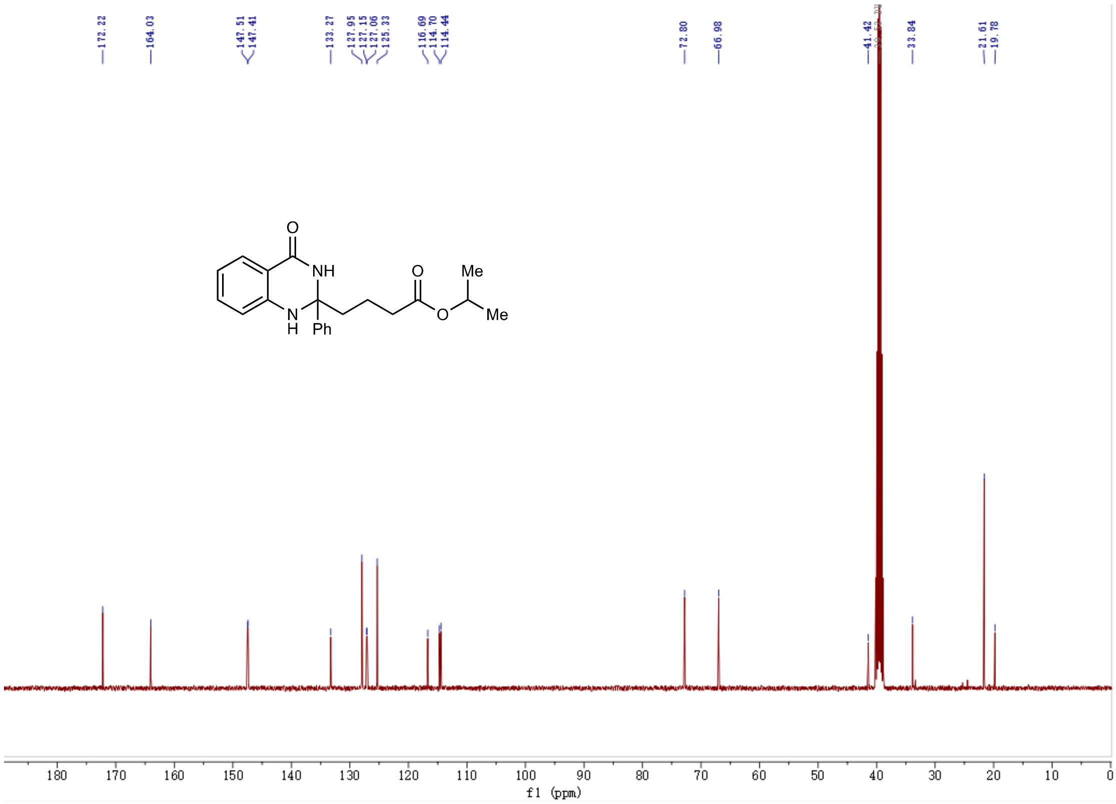

Supplementary Figure 72. ^{13}C NMR spectra of compound **2n**

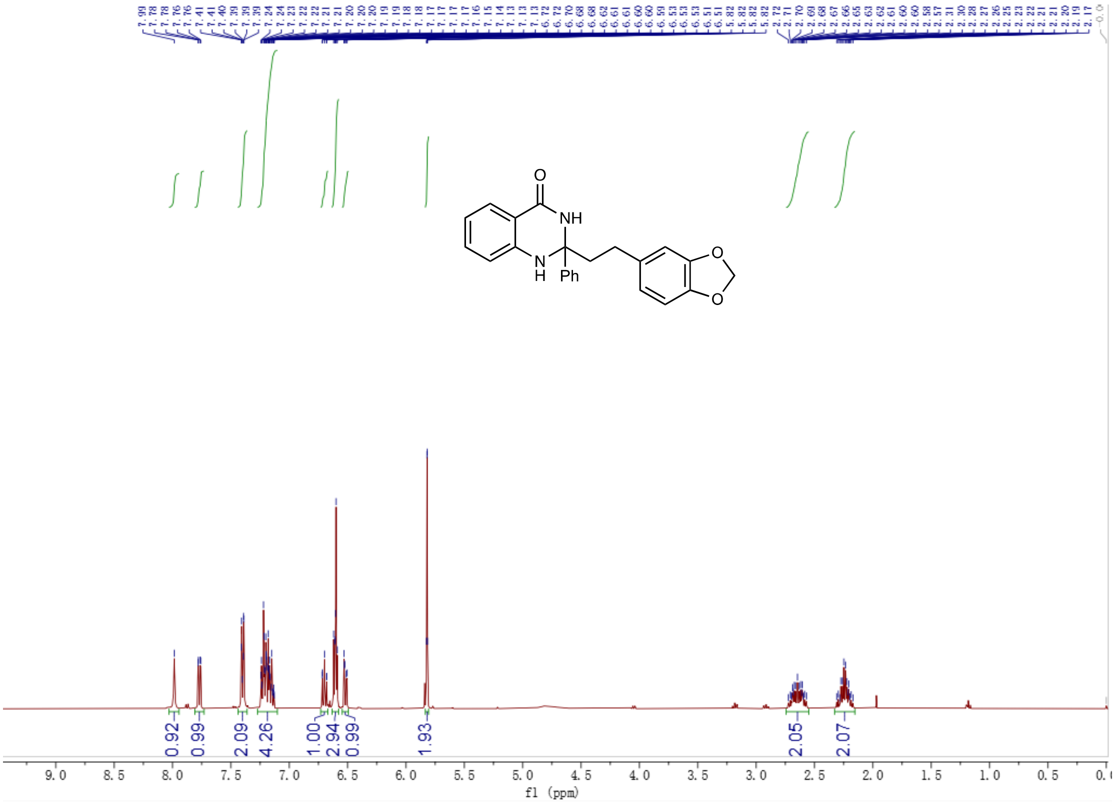

Supplementary Figure 73. ^{1}H NMR spectra of compound **2o**

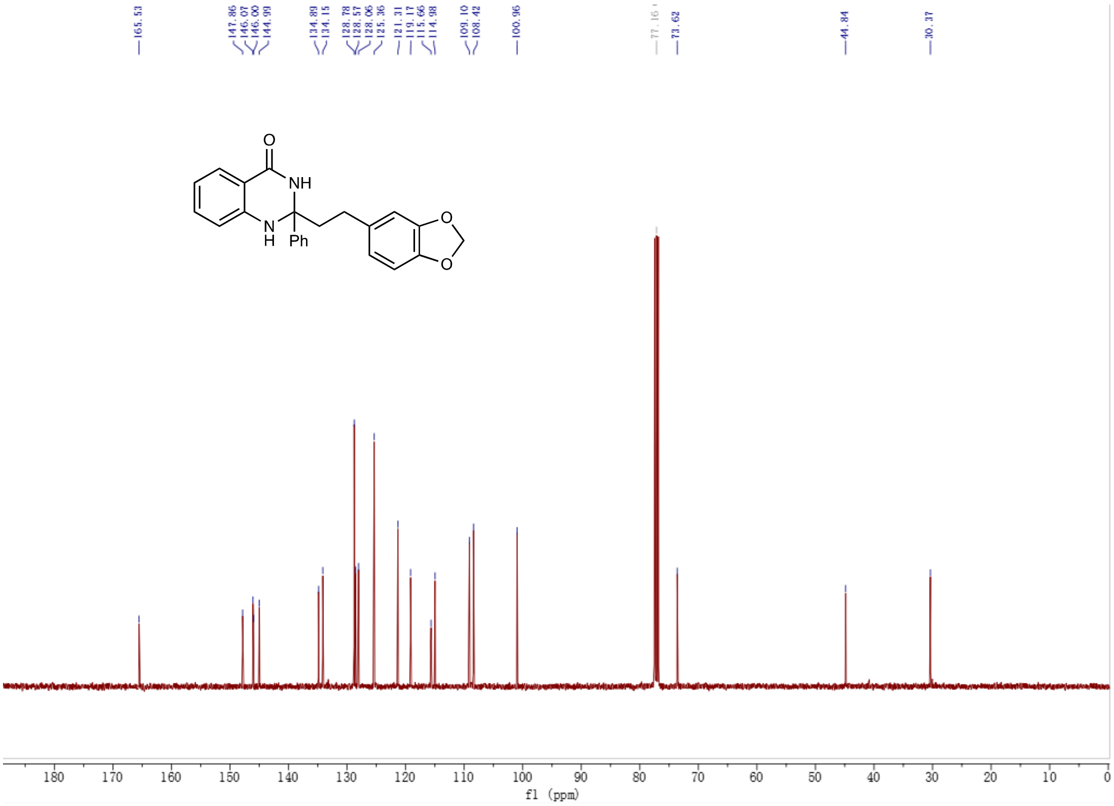

Supplementary Figure 74. ^{13}C NMR spectra of compound **2o**

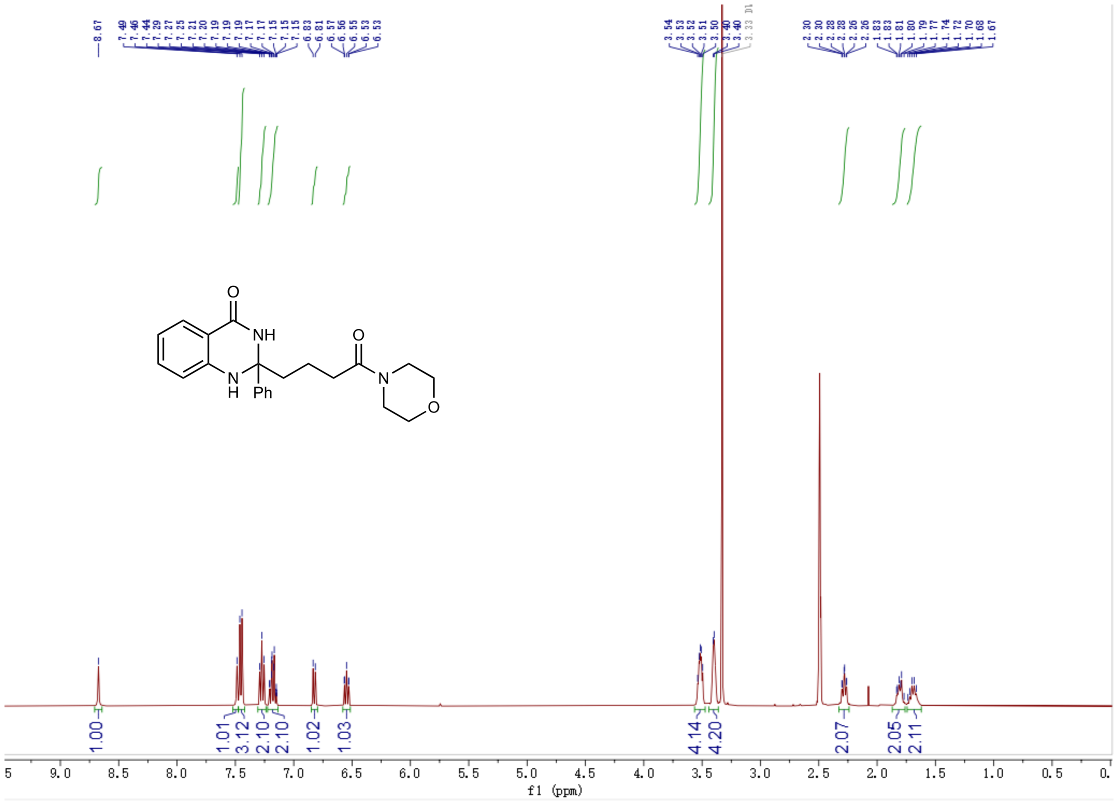

Supplementary Figure 75. ^{1}H NMR spectra of compound **2p**

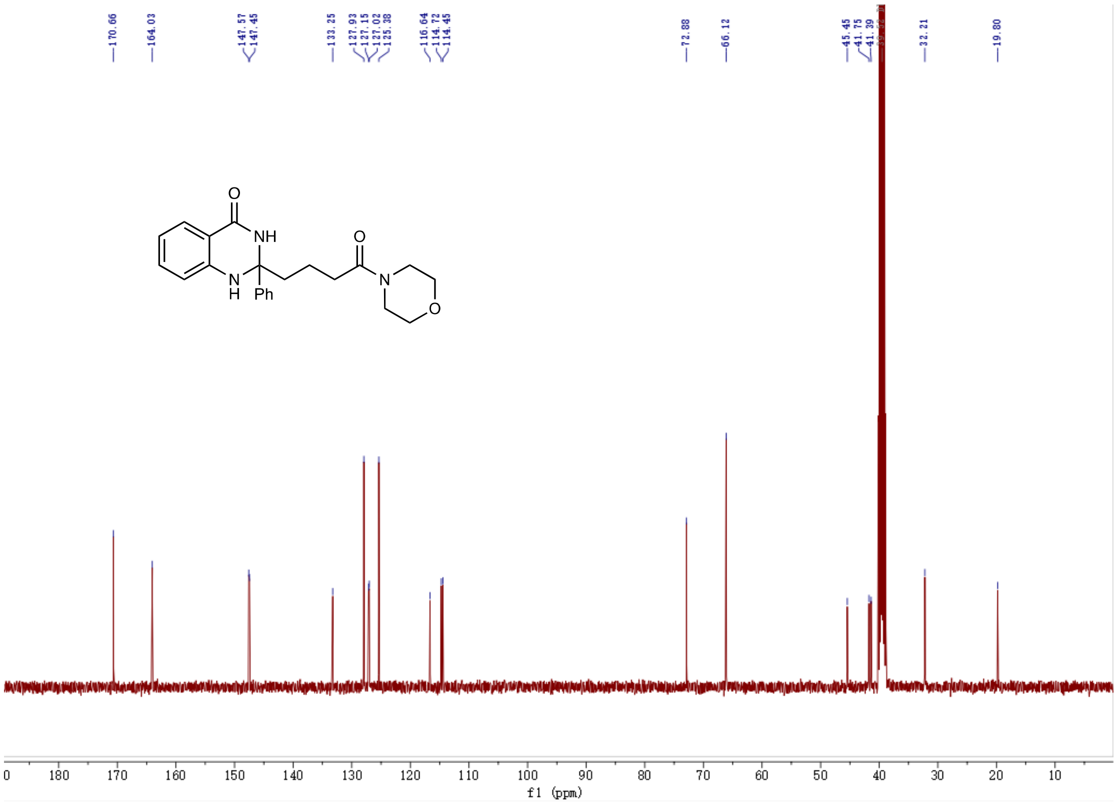

Supplementary Figure 76. ^{13}C NMR spectra of compound **2p**

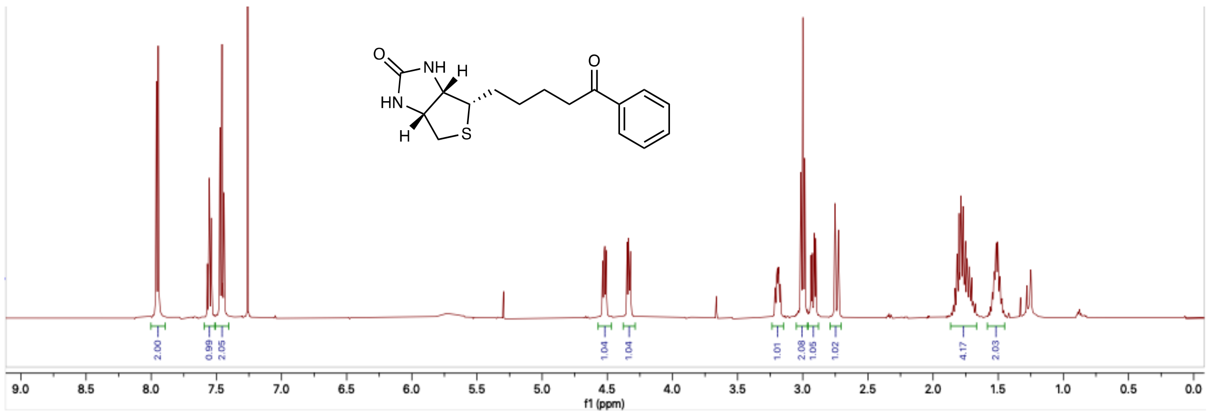

Supplementary Figure 77. ^{1}H NMR spectra of compound **S1**

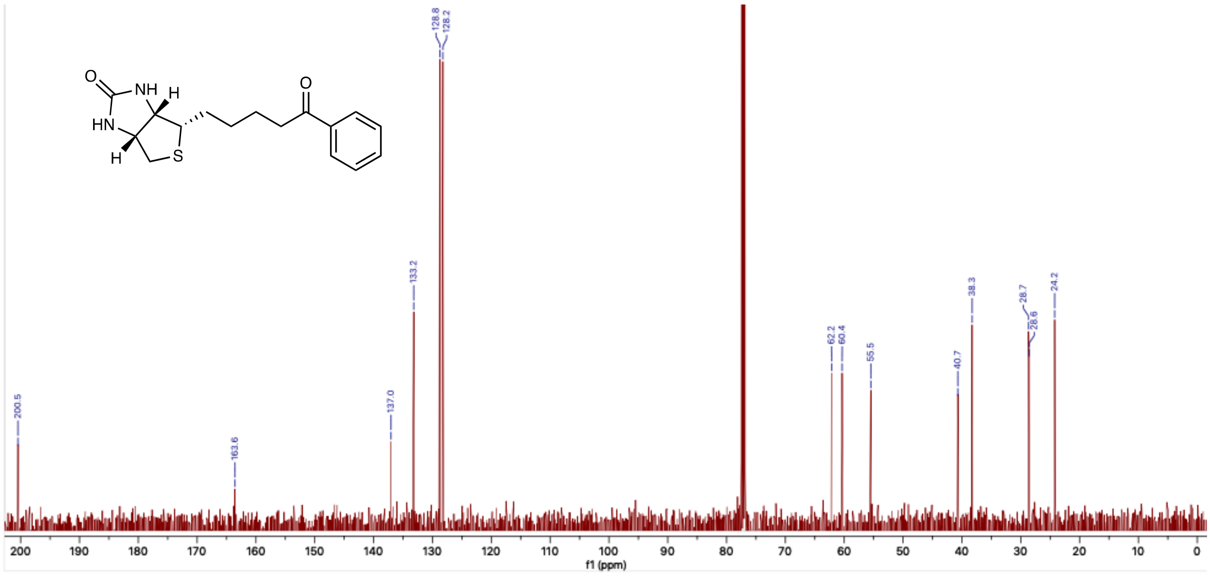

Supplementary Figure 78. ^{13}C NMR spectra of compound **S1**

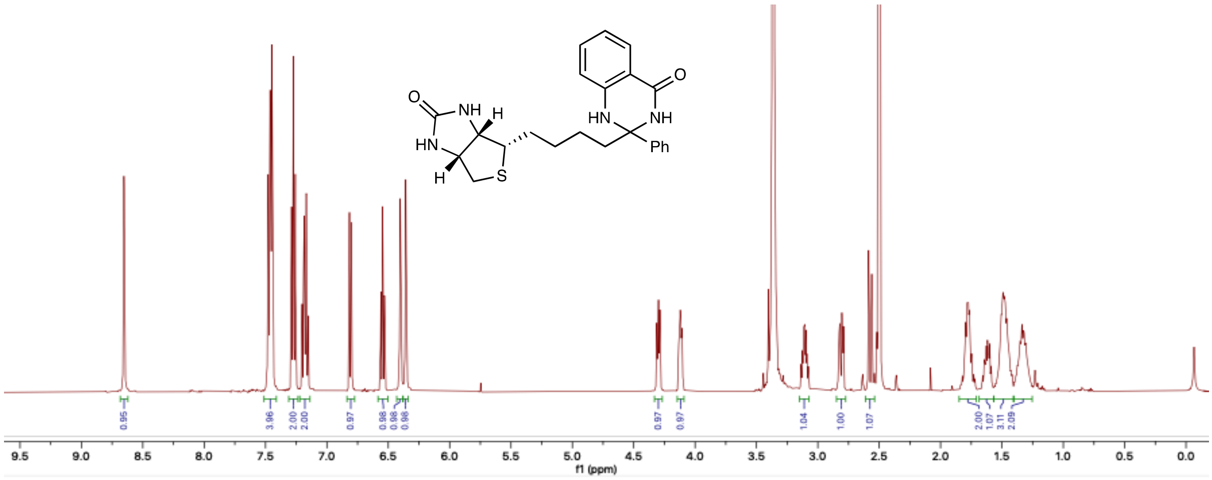

Supplementary Figure 79. ^{1}H NMR spectra of compound **2q**

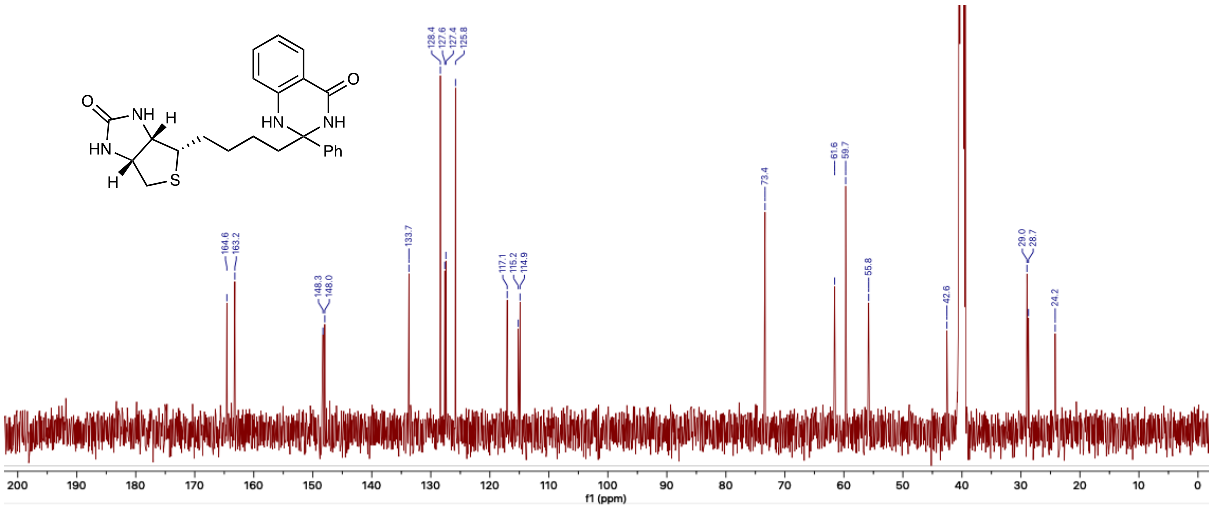

Supplementary Figure 80. ^{13}C NMR spectra of compound **2q**

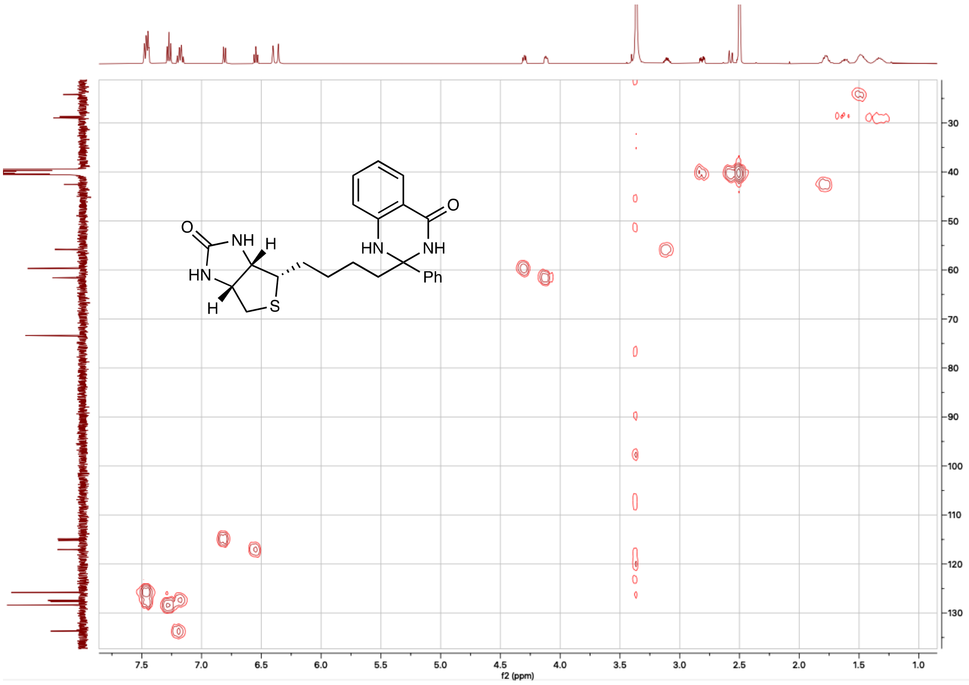

Supplementary Figure 81. HMQC NMR spectra of compound **2q**

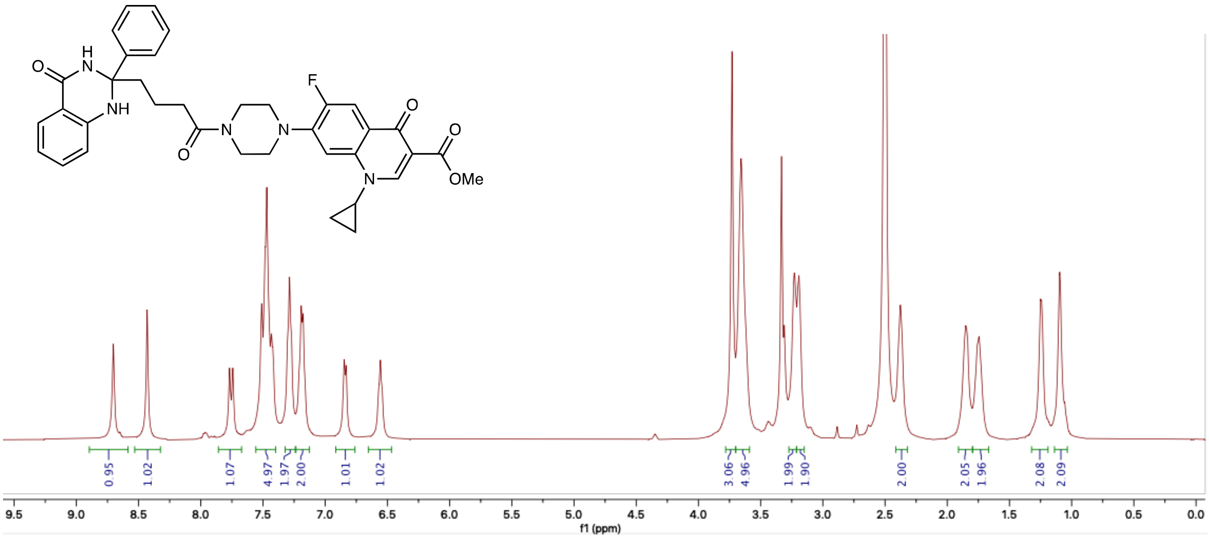

Supplementary Figure 82. ^{1}H NMR spectra of compound **2r**

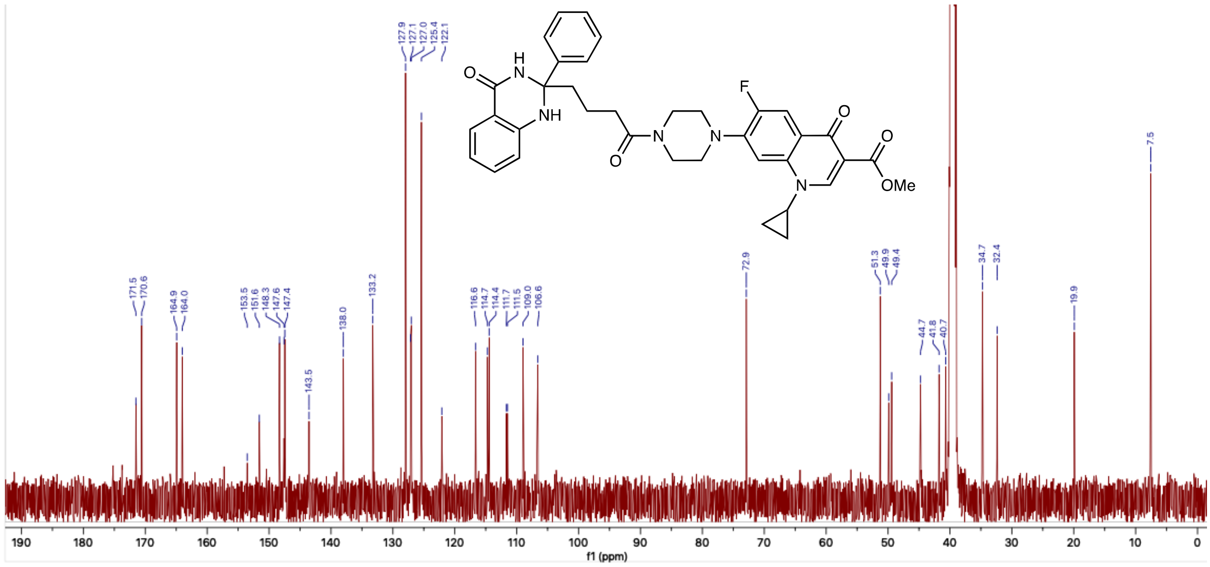

Supplementary Figure 83. ^{13}C NMR spectra of compound **2r**

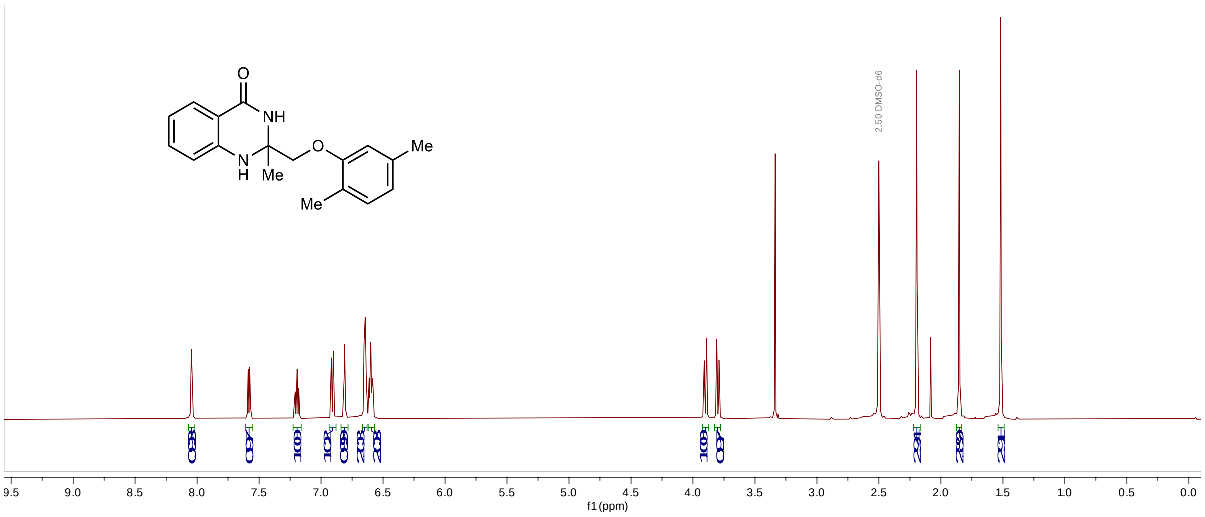

Supplementary Figure 84. ^{1}H NMR spectra of compound **2s**

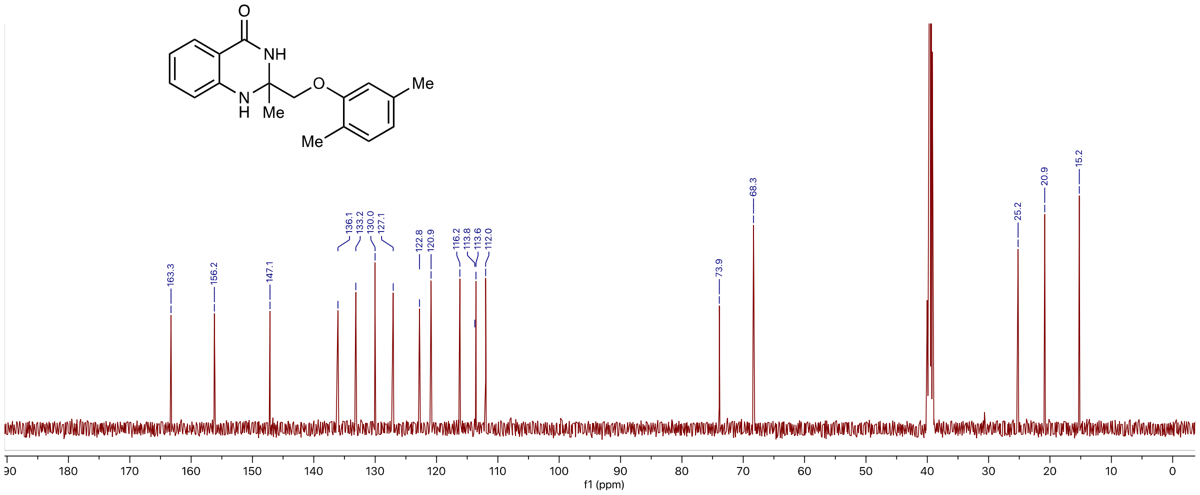

Supplementary Figure 85. ^{13}C NMR spectra of compound **2s**

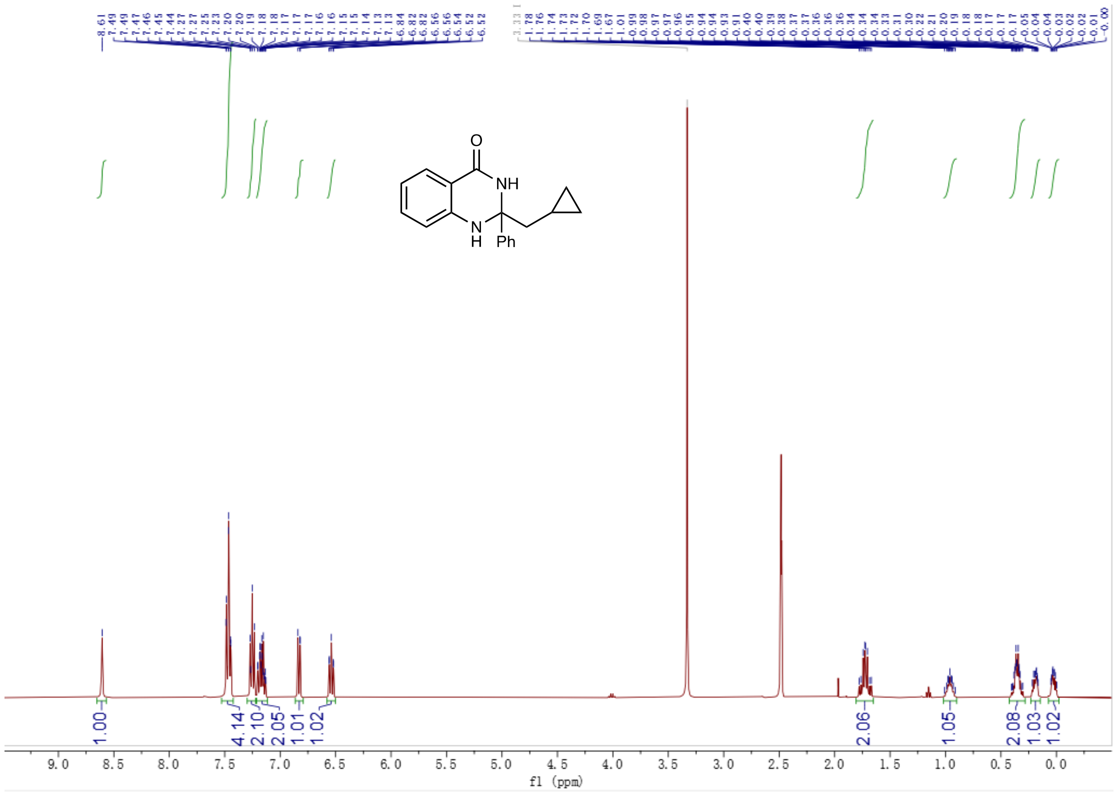

Supplementary Figure 86. ^{1}H NMR spectra of compound **2t**

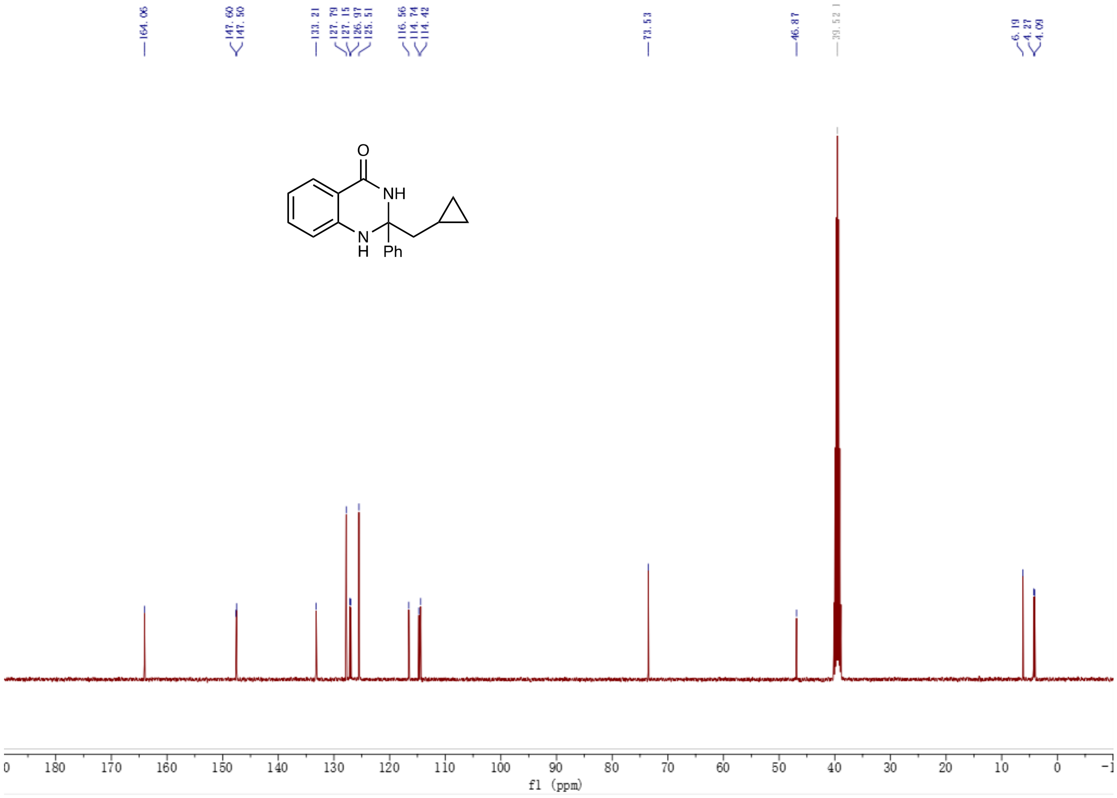

Supplementary Figure 87. ^{13}C NMR spectra of compound **2t**

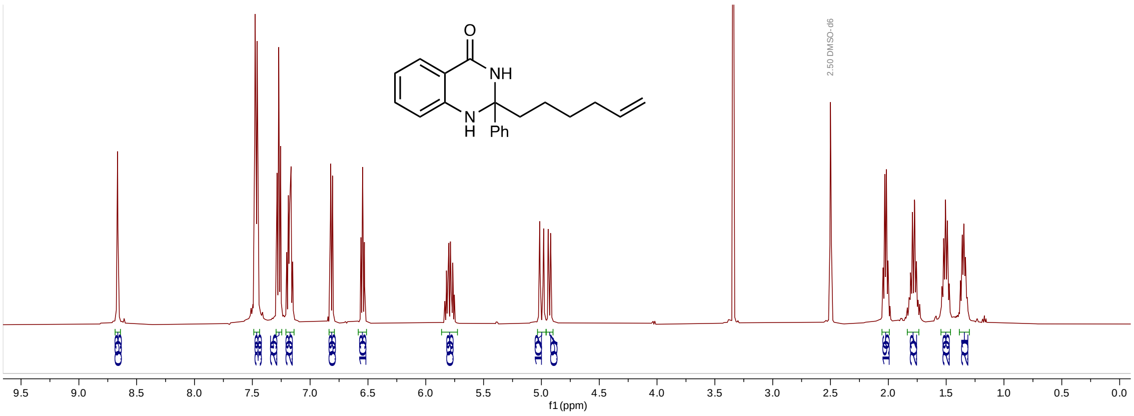

Supplementary Figure 88. ^{1}H NMR spectra of compound **2u**

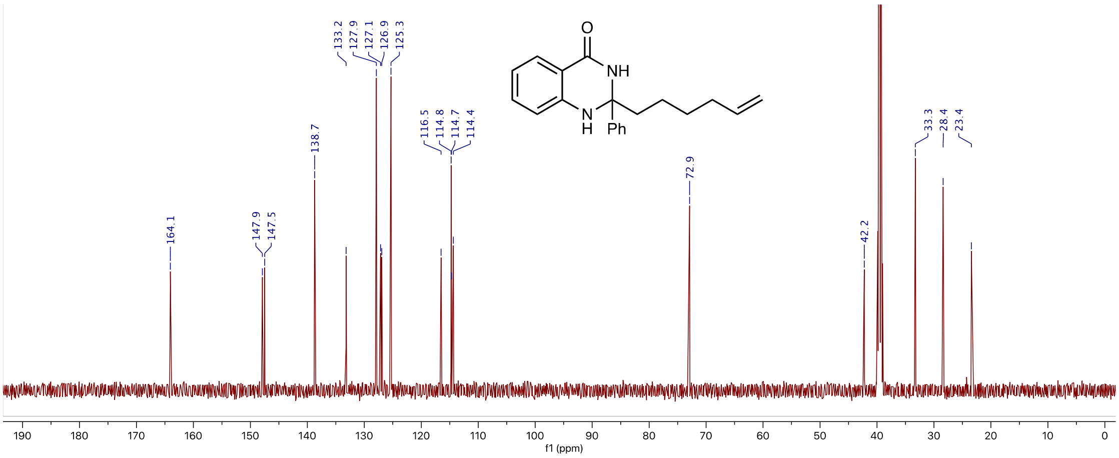

Supplementary Figure 89. ^{13}C NMR spectra of compound **2u**

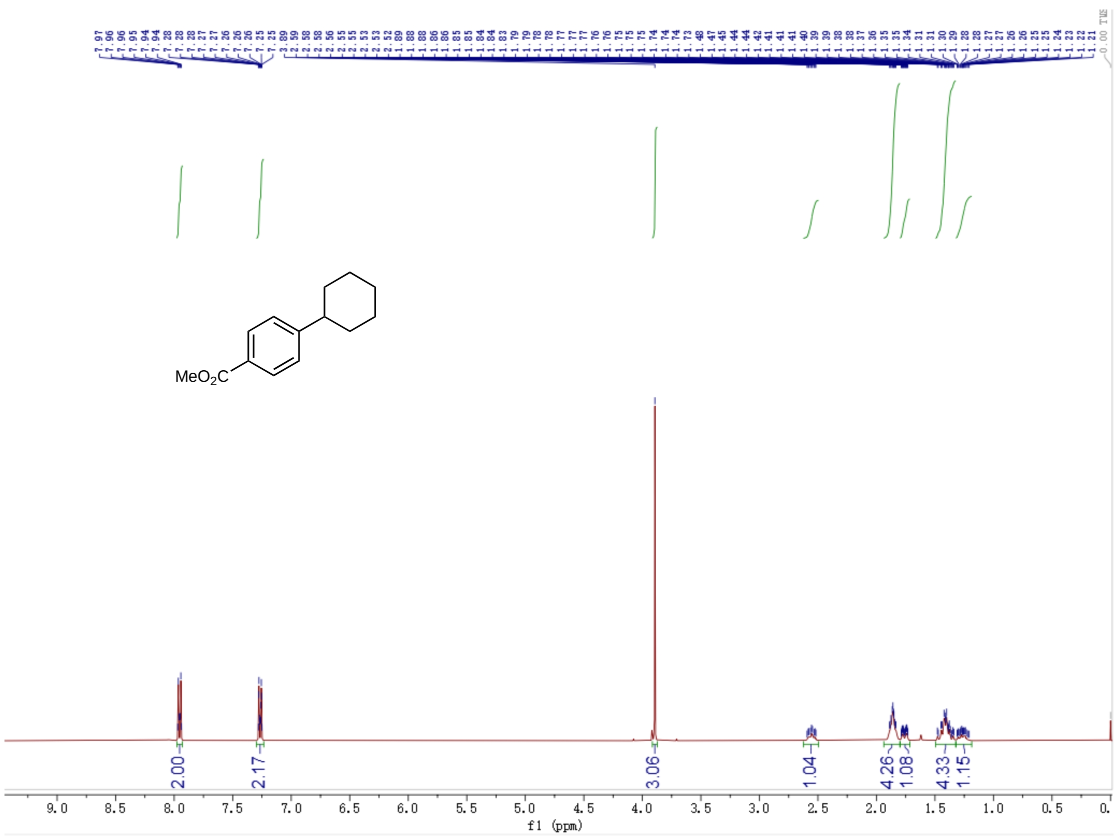

Supplementary Figure 90. ^{1}H NMR spectra of compound **3a**

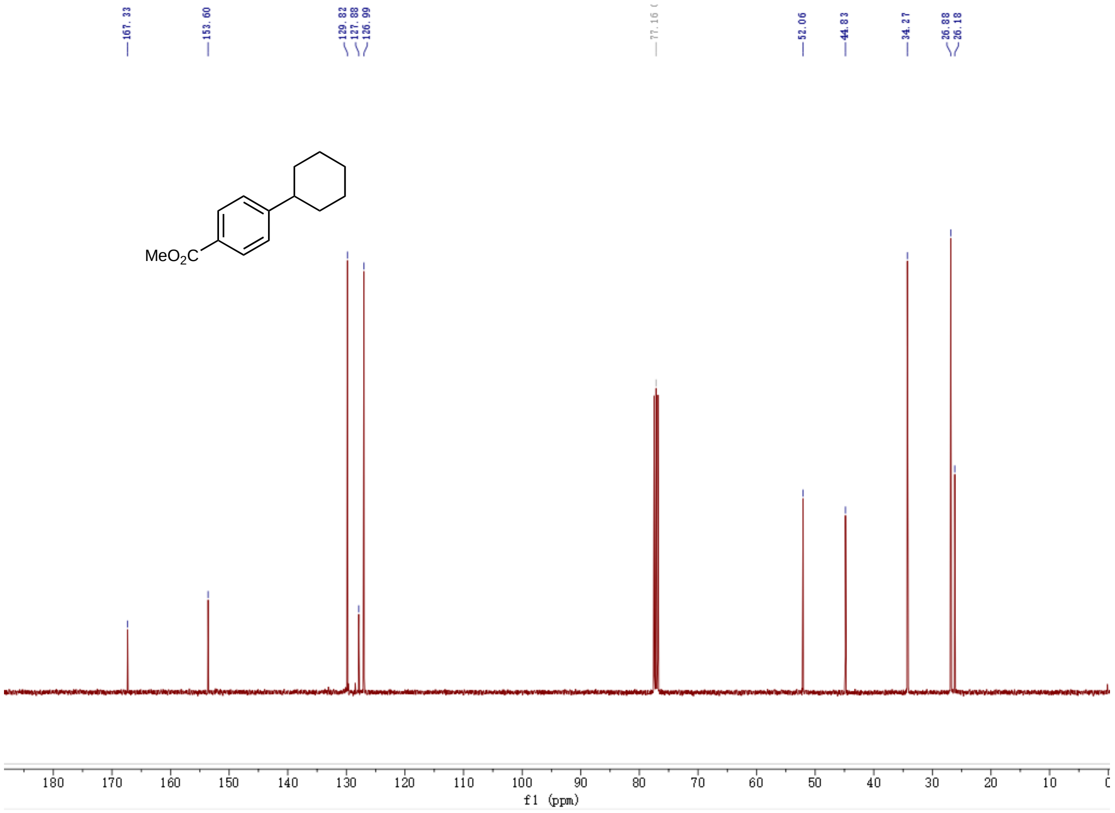

Supplementary Figure 91. ^{13}C NMR spectra of compound **3a**

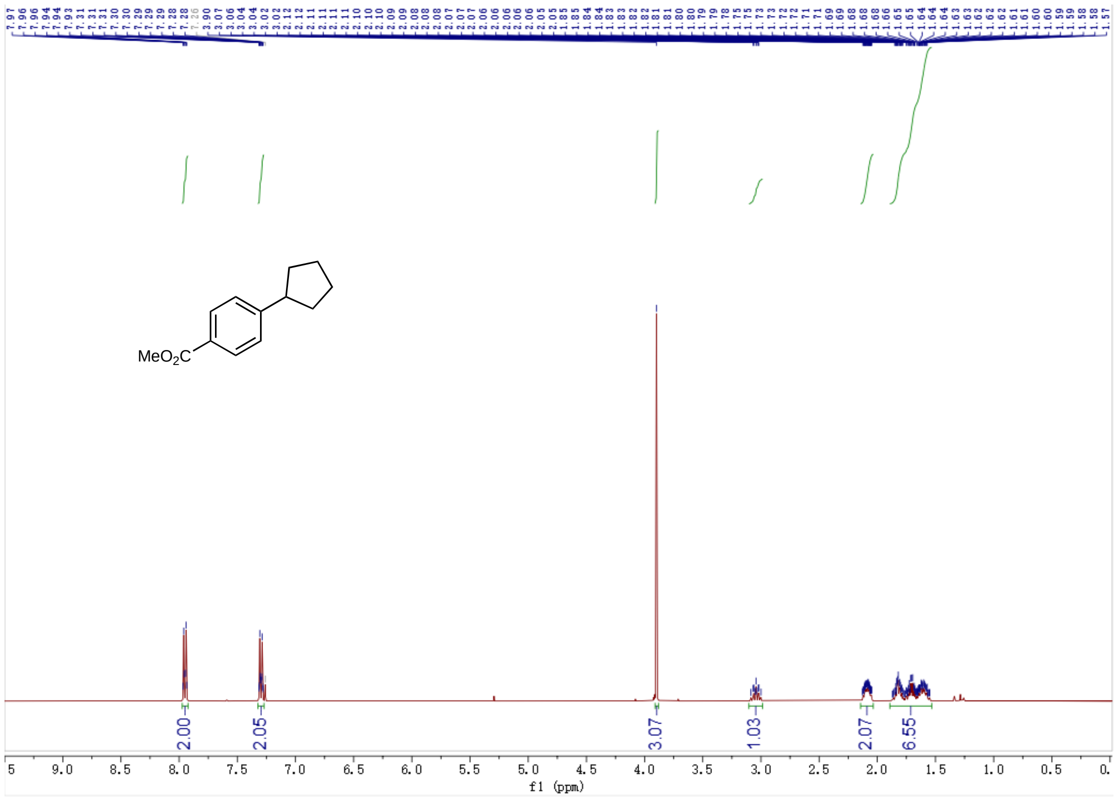

Supplementary Figure 92. ^{1}H NMR spectra of compound **3b**

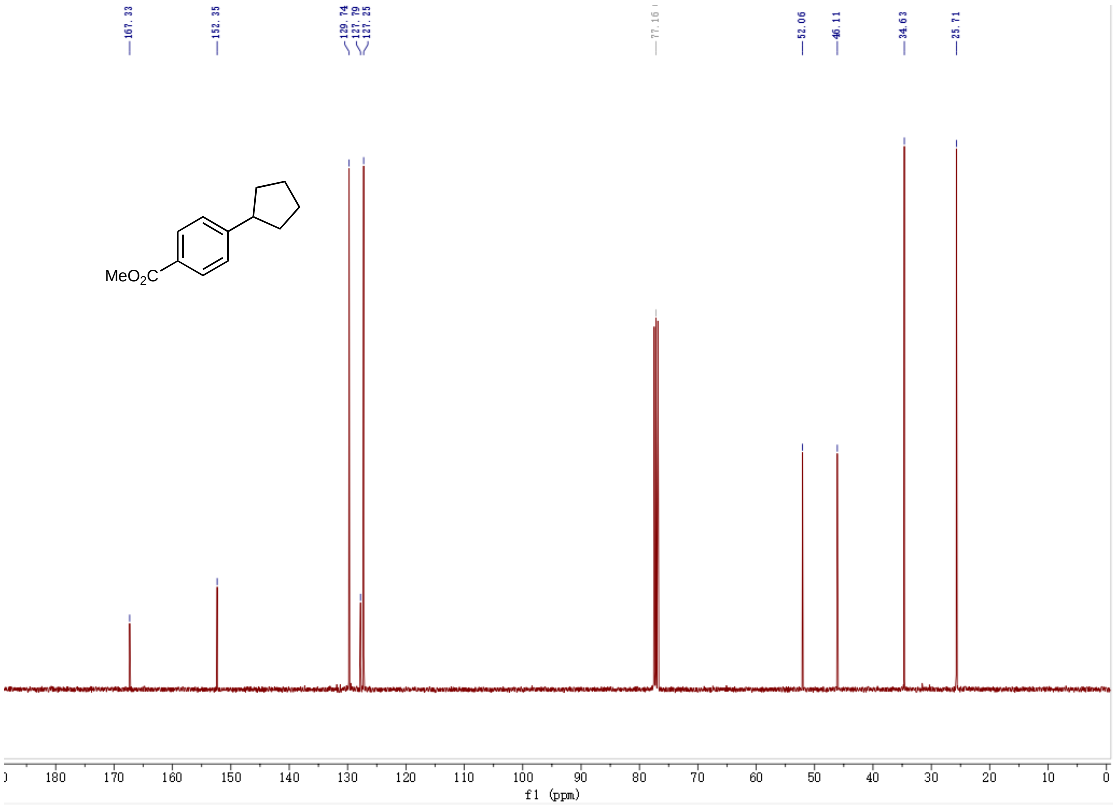

Supplementary Figure 93. ^{13}C NMR spectra of compound **3b**

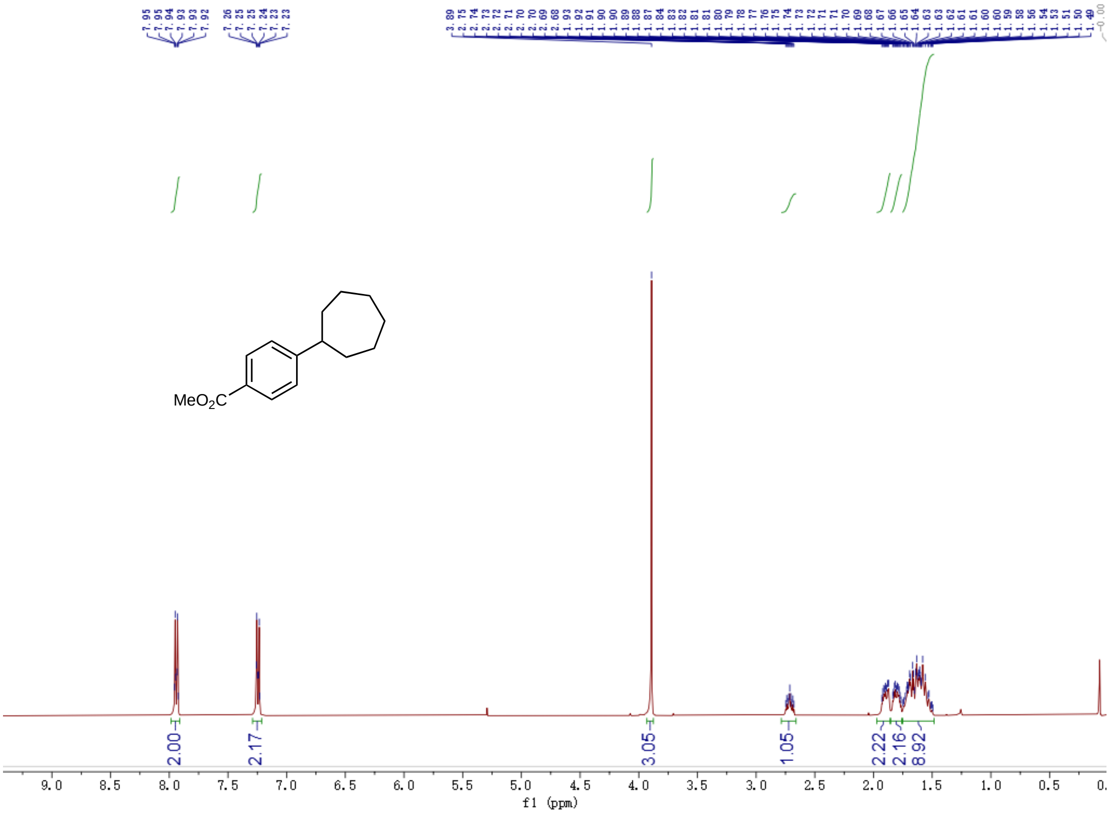

Supplementary Figure 94. ^{1}H NMR spectra of compound **3c**

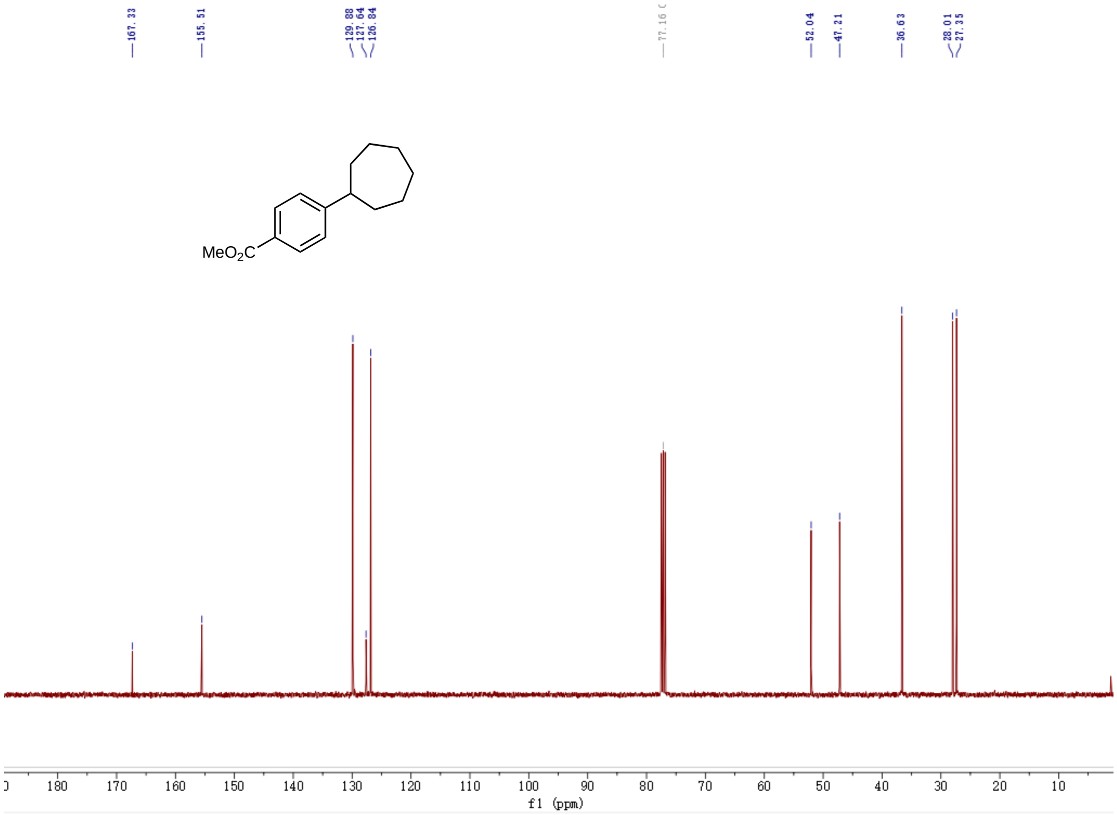

Supplementary Figure 95. ^{13}C NMR spectra of compound **3c**

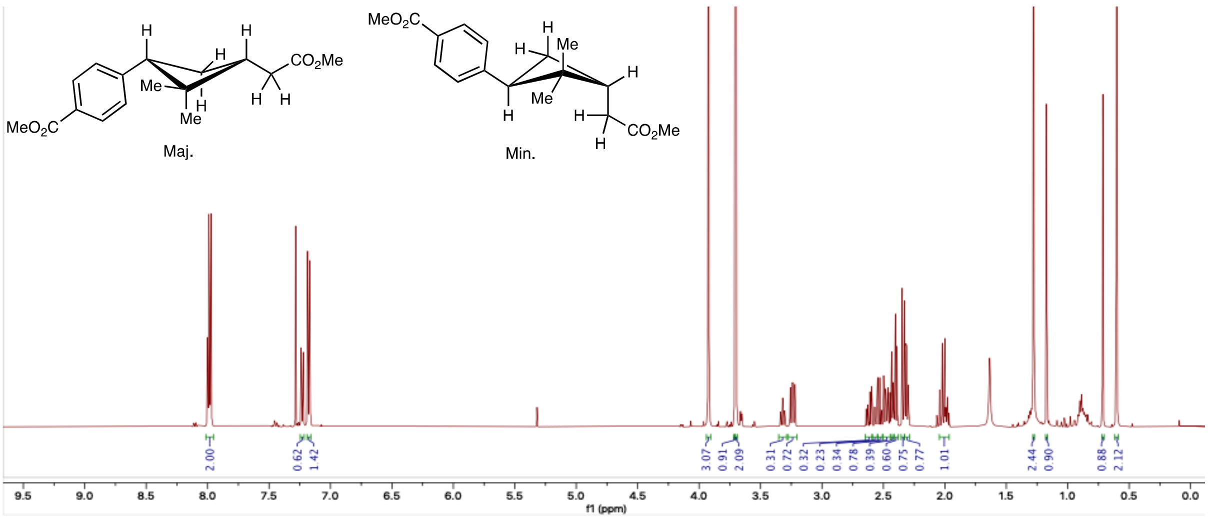

Supplementary Figure 96. ^{1}H NMR spectra of compound **3d**

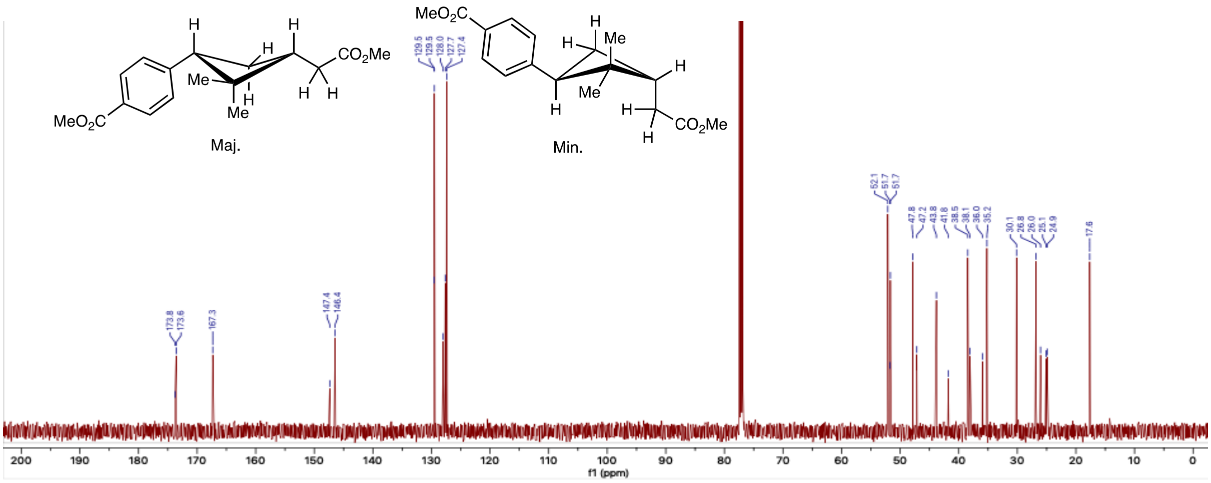

Supplementary Figure 97. ^{13}C NMR spectra of compound **3d**

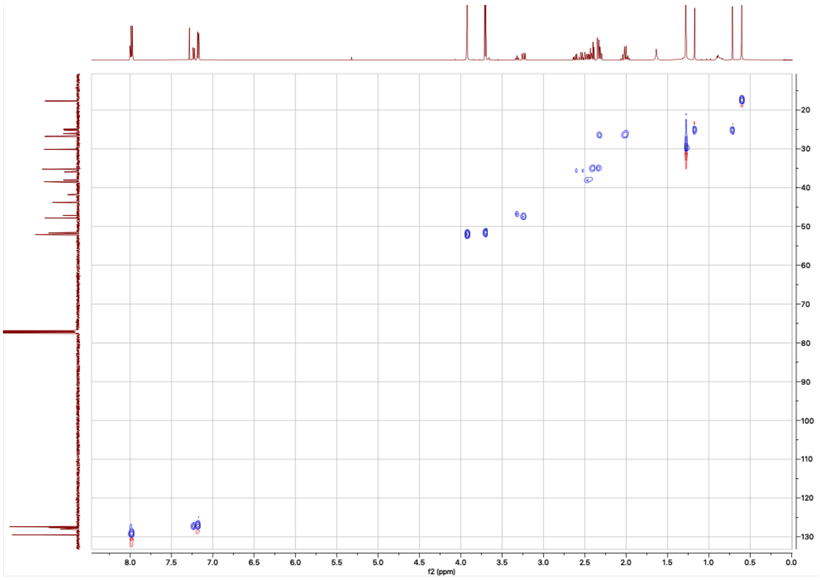

Supplementary Figure 98. HMQC NMR spectra of compound **3d**

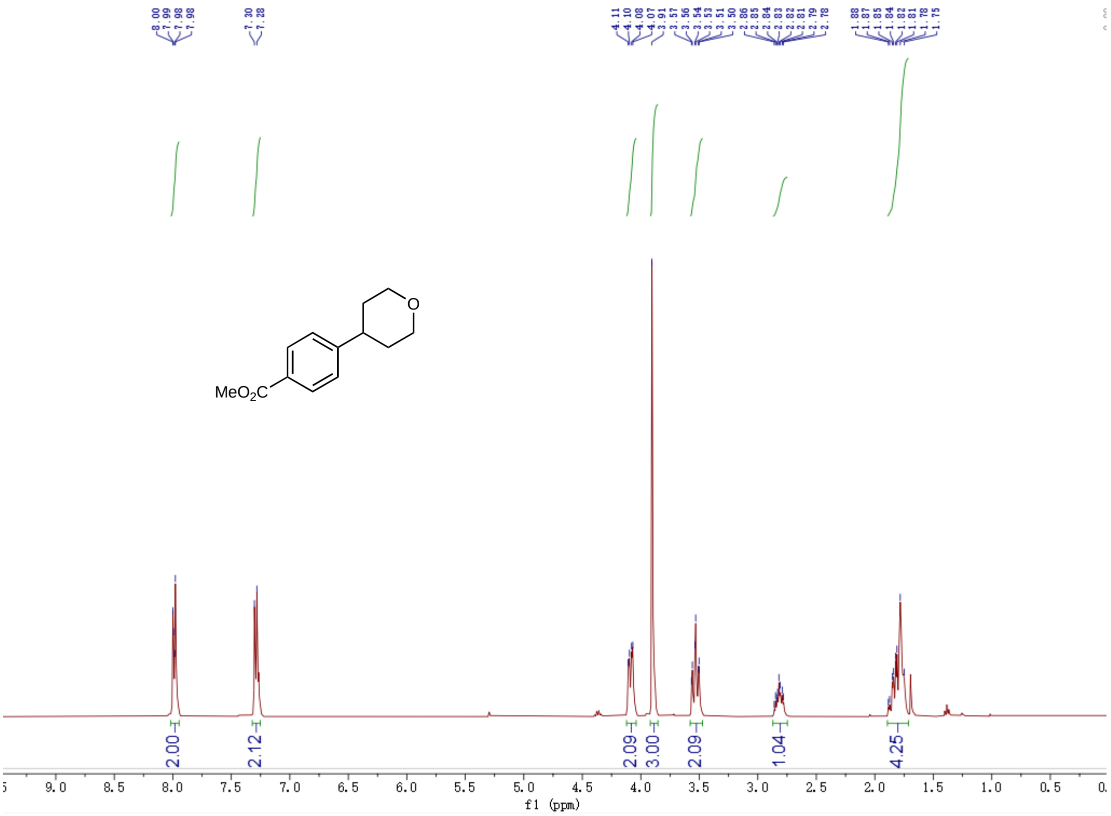

Supplementary Figure 99. ^{1}H NMR spectra of compound **3e**

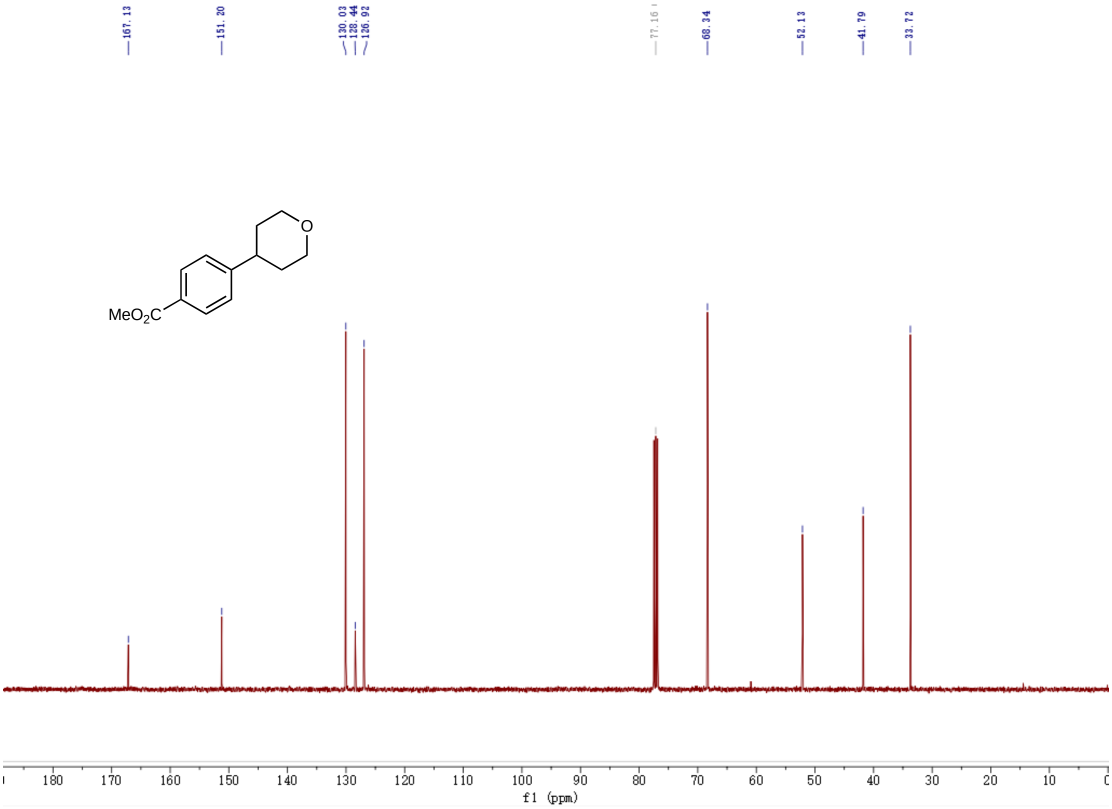

Supplementary Figure 100. ^{13}C NMR spectra of compound **3e**

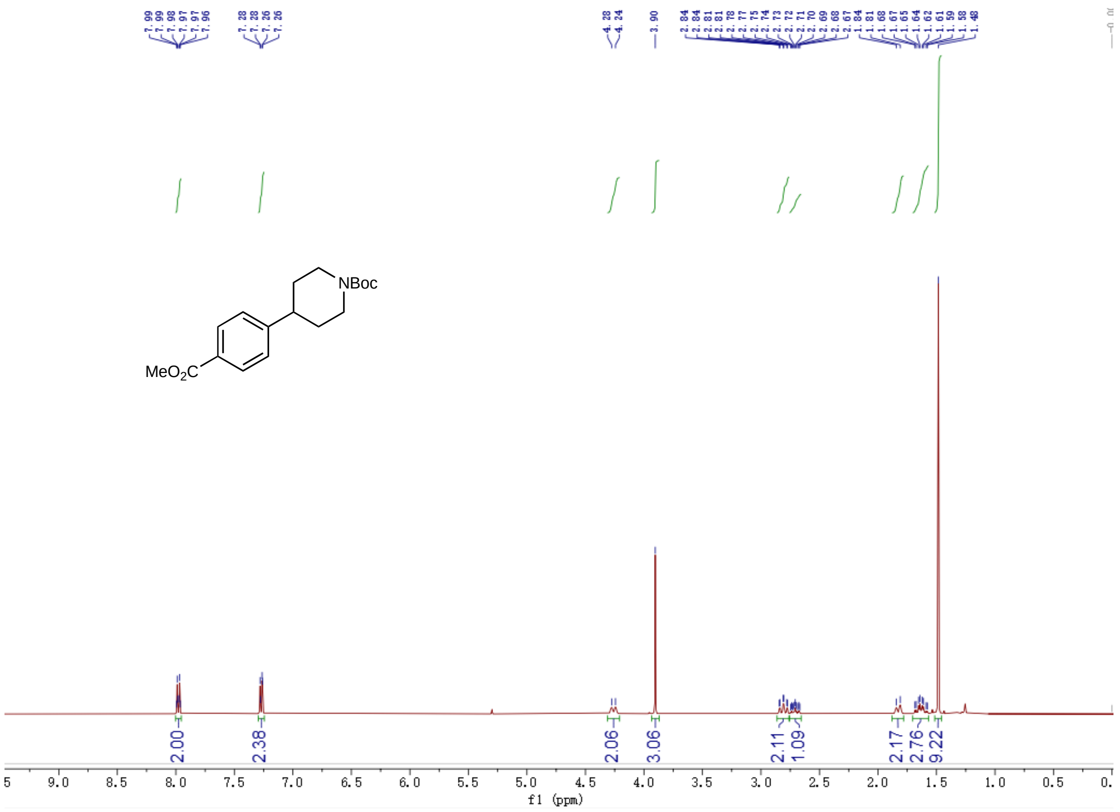

Supplementary Figure 101. ^{1}H NMR spectra of compound **3f**

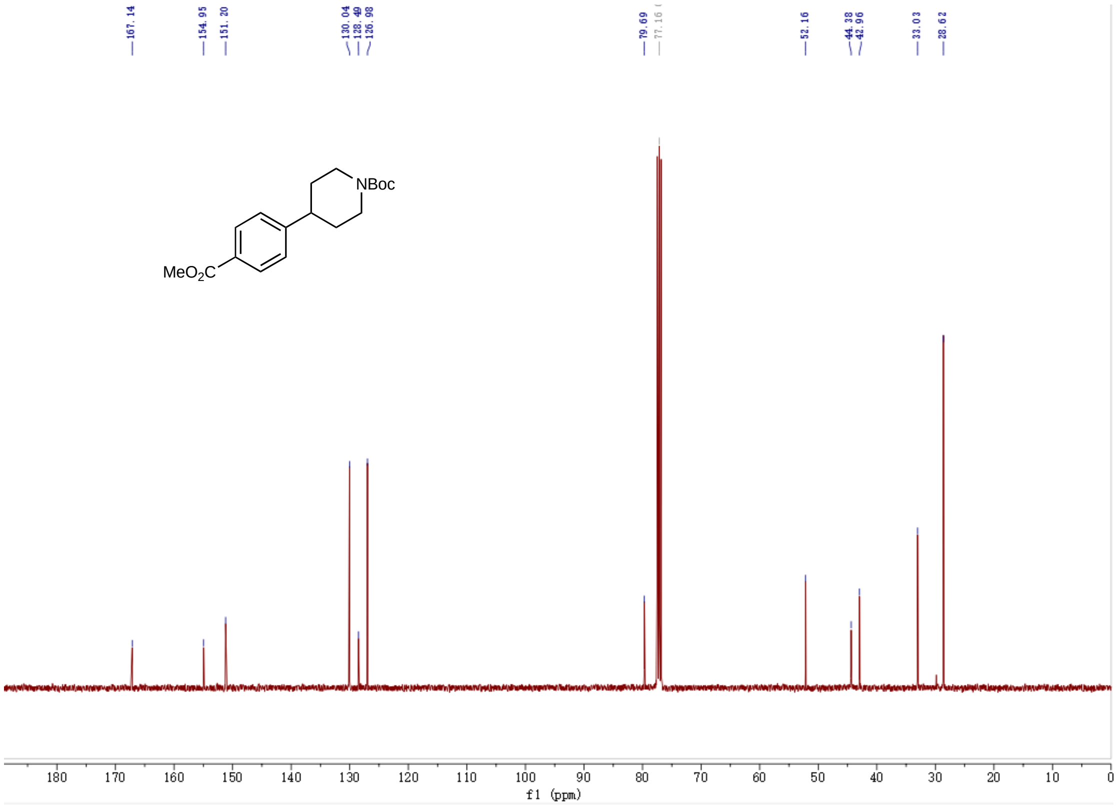

Supplementary Figure 102. ^{13}C NMR spectra of compound **3f**

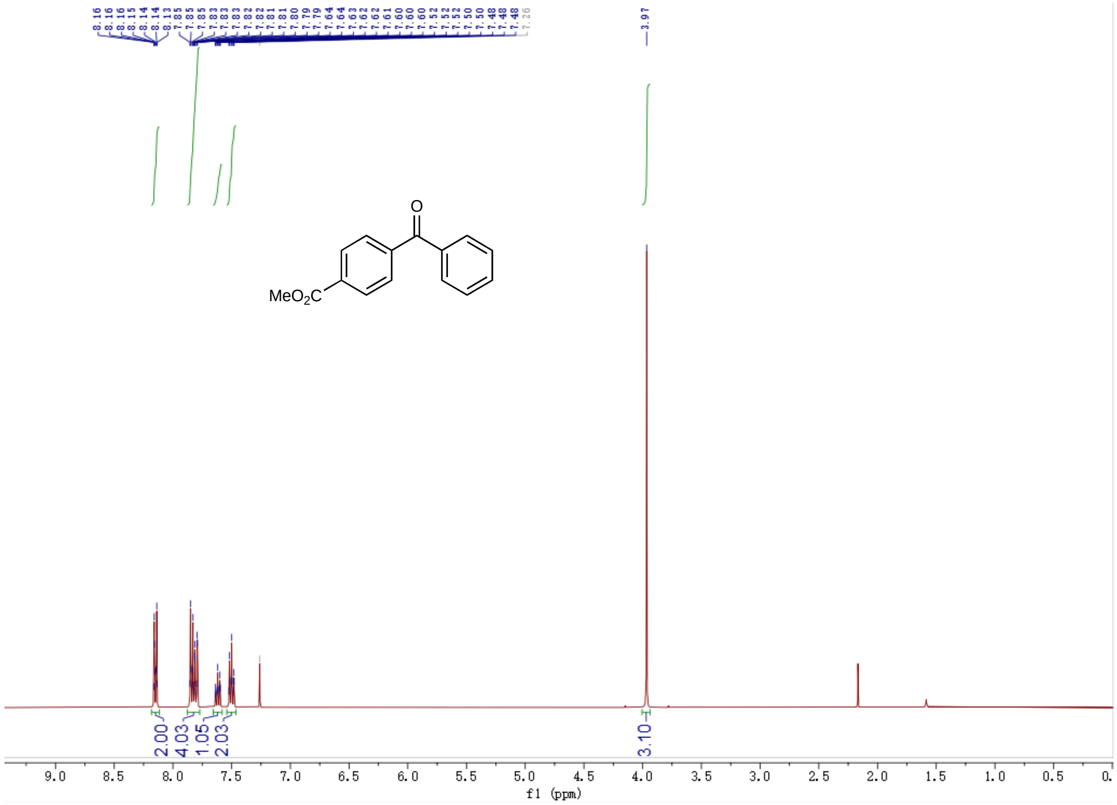

Supplementary Figure 103. ^{1}H NMR spectra of compound **3g**

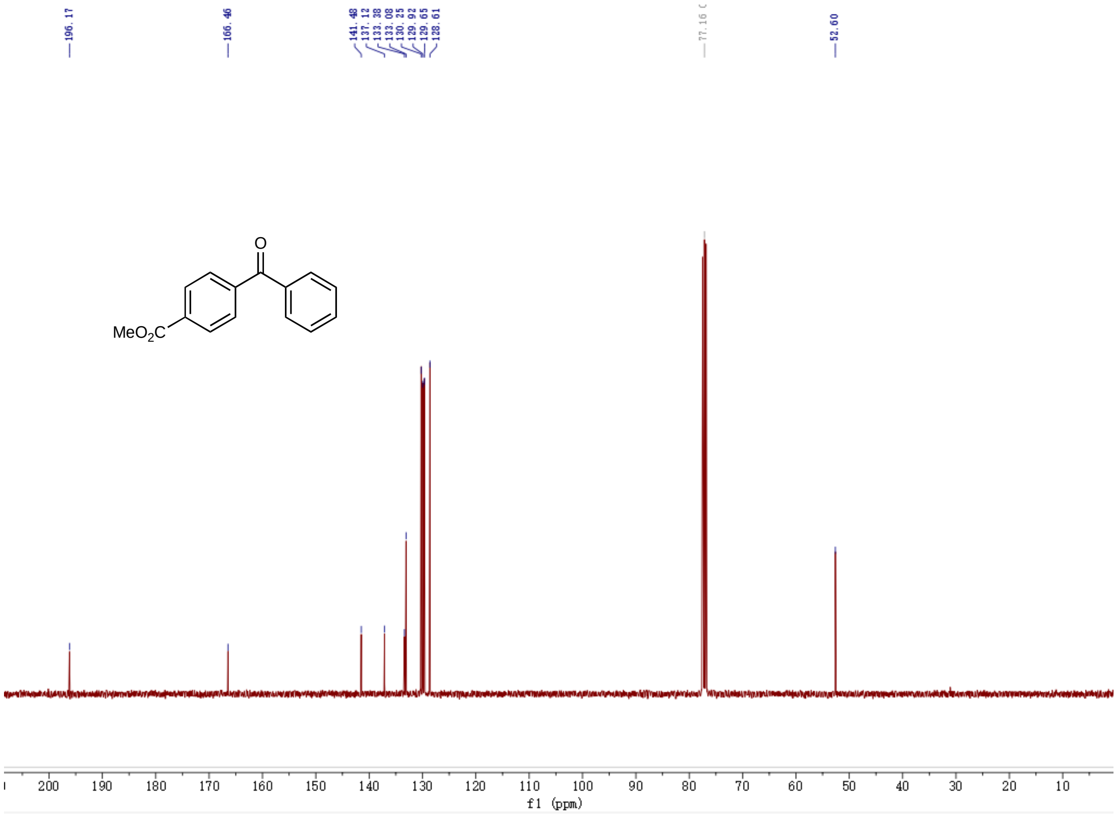

Supplementary Figure 104. ^{13}C NMR spectra of compound **3g**

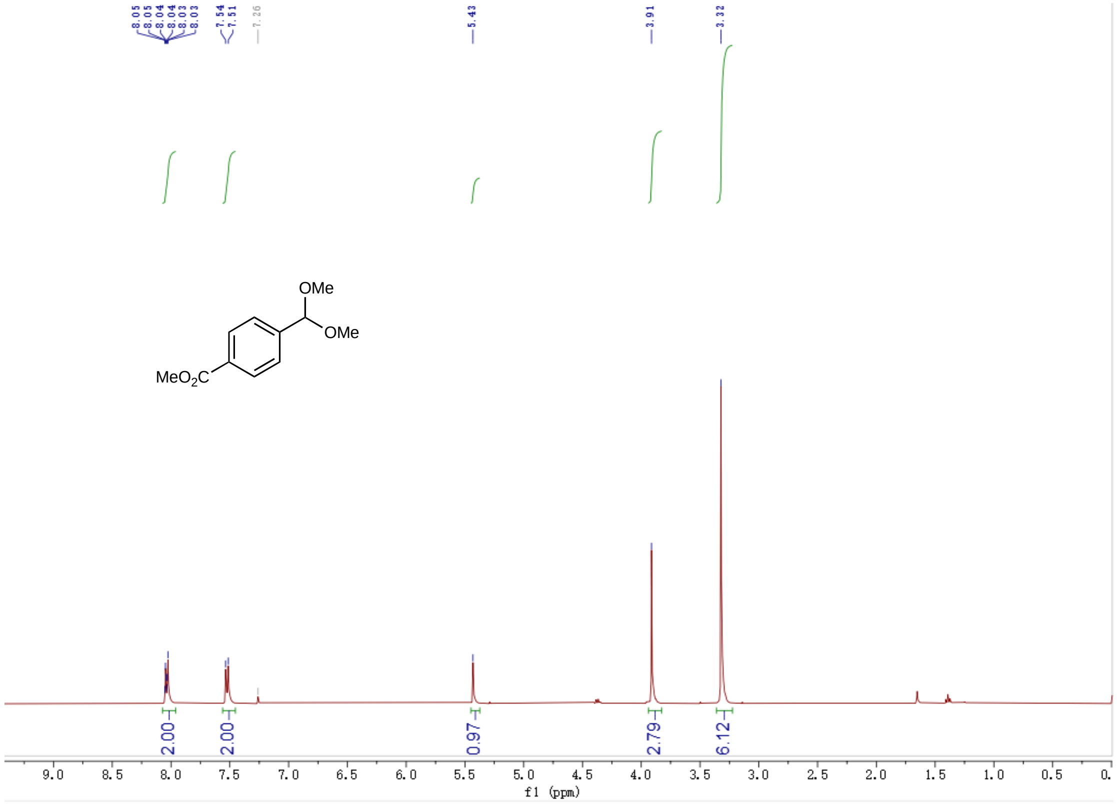

Supplementary Figure 105. ^{1}H NMR spectra of compound **3h**

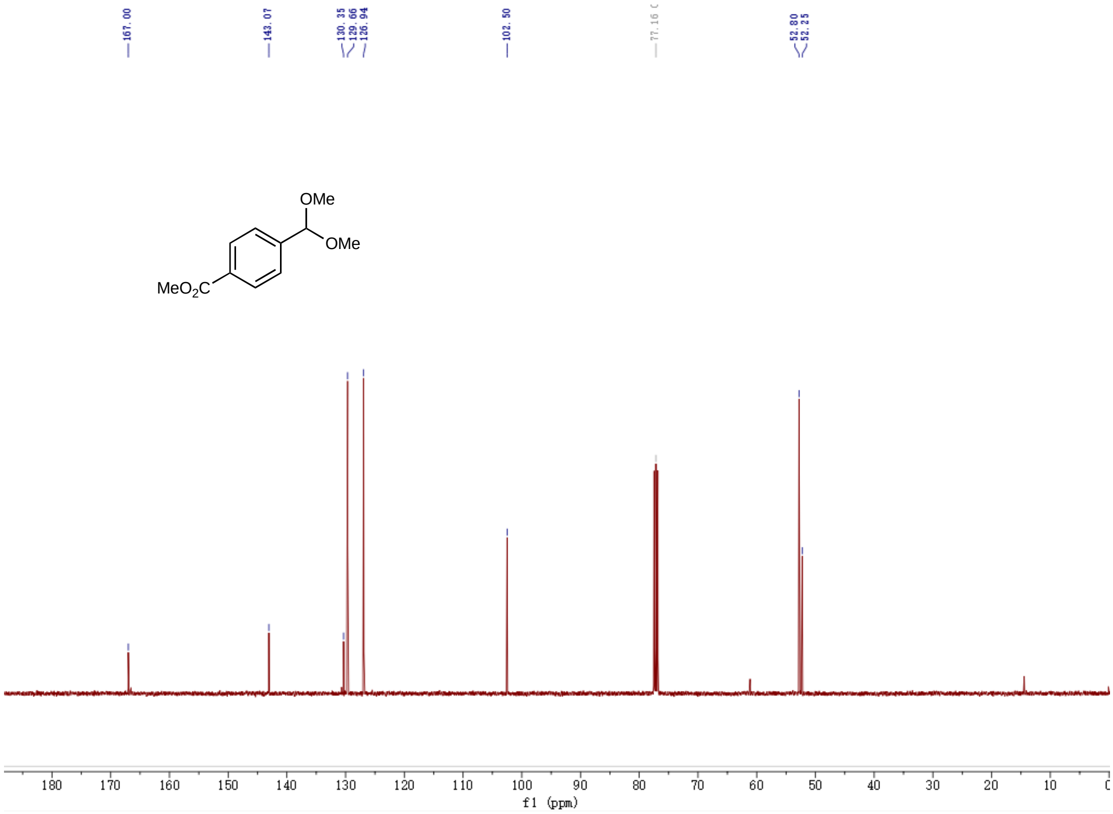

Supplementary Figure 106. ^{13}C NMR spectra of compound **3h**

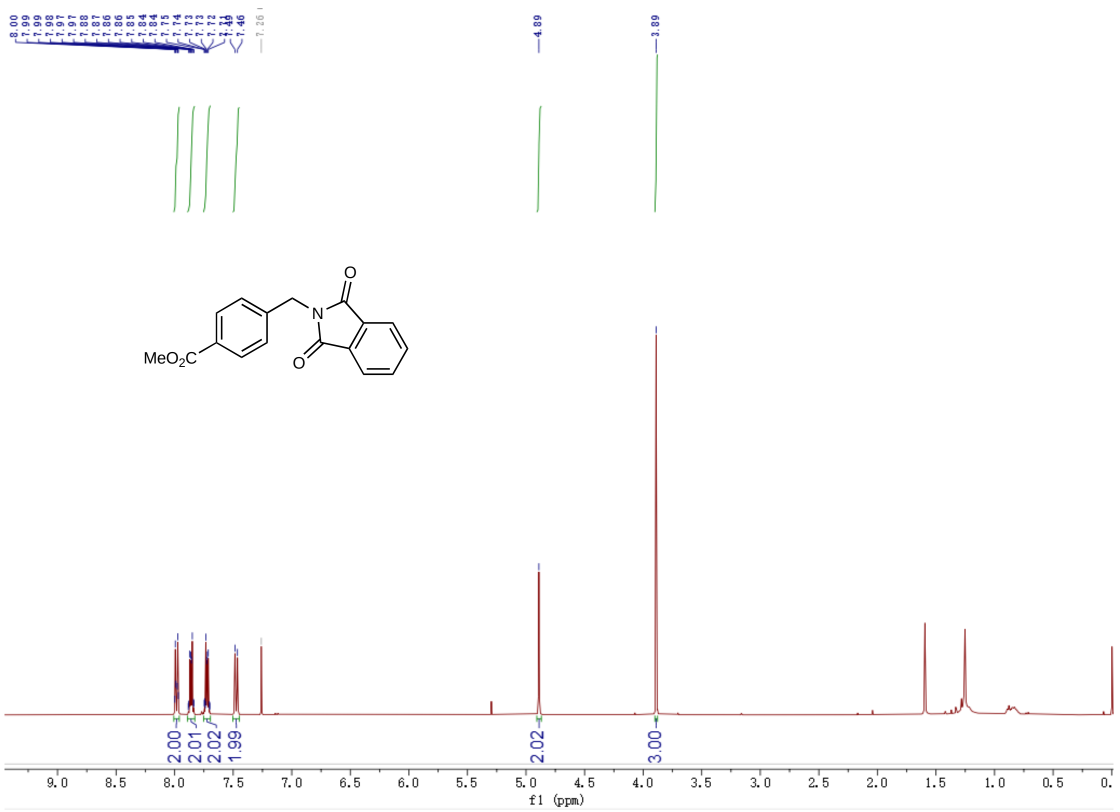

Supplementary Figure 107. ^{1}H NMR spectra of compound **3i**

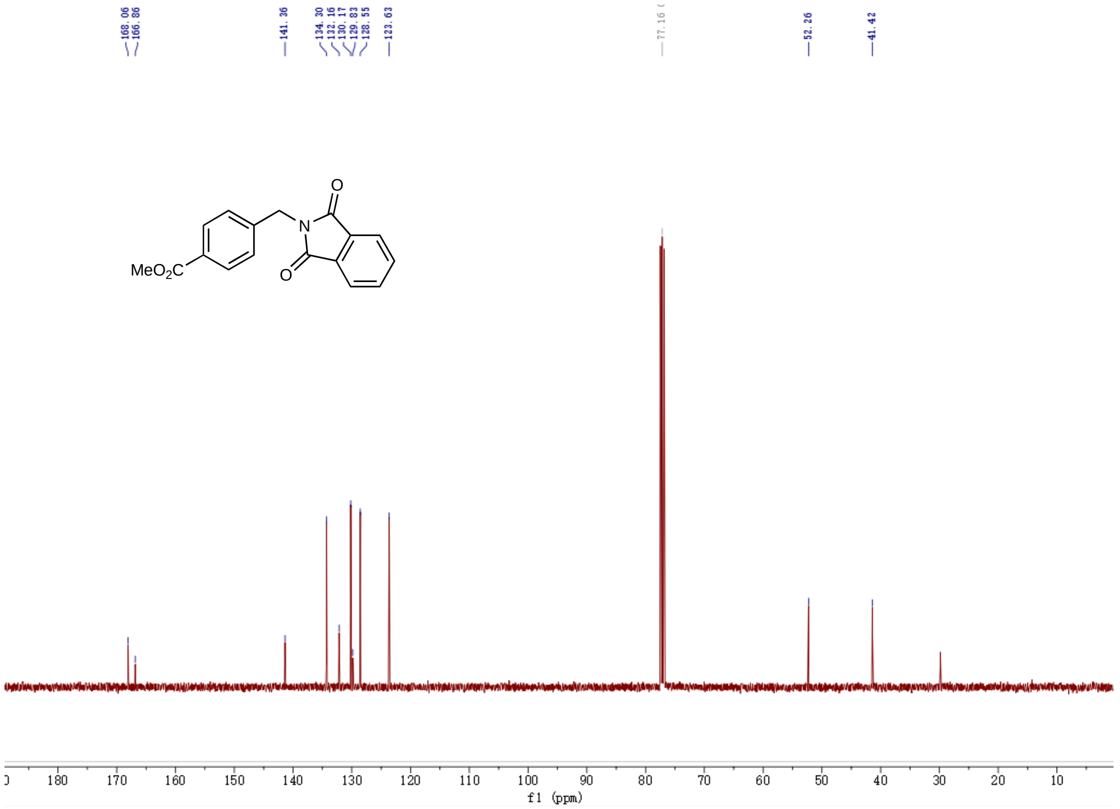

Supplementary Figure 108. ^{13}C NMR spectra of compound **3i**

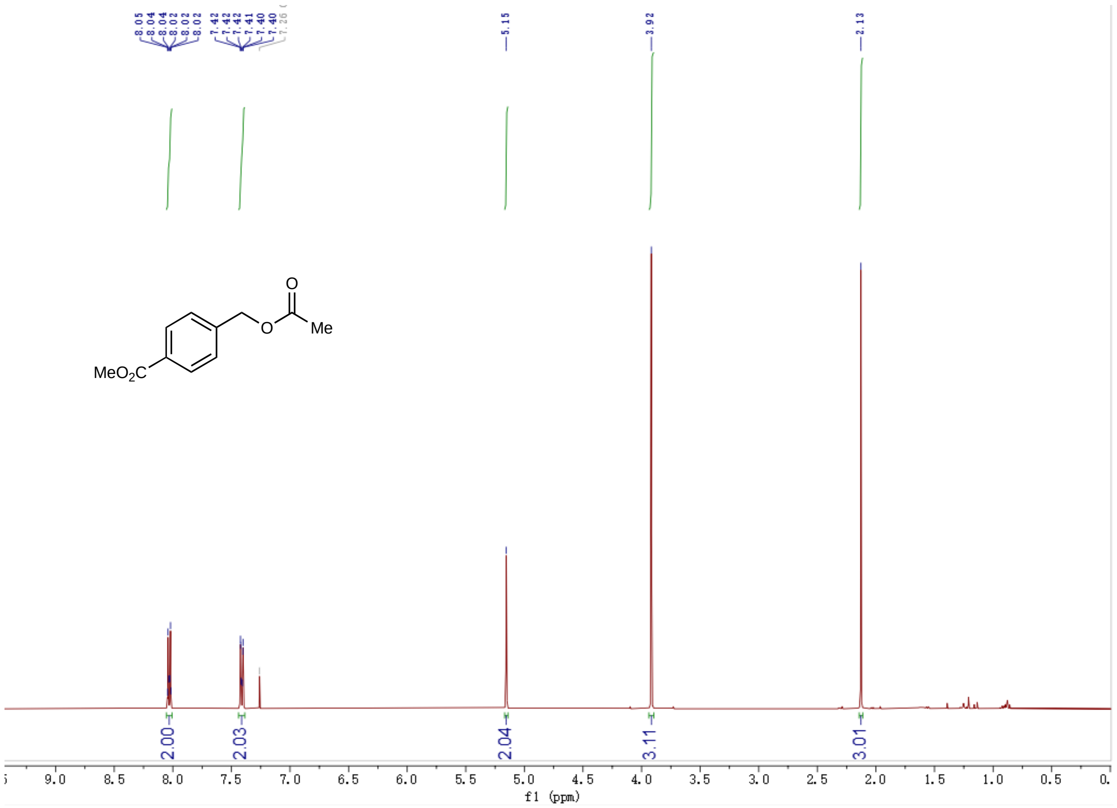

Supplementary Figure 109. ^{1}H NMR spectra of compound **3j**

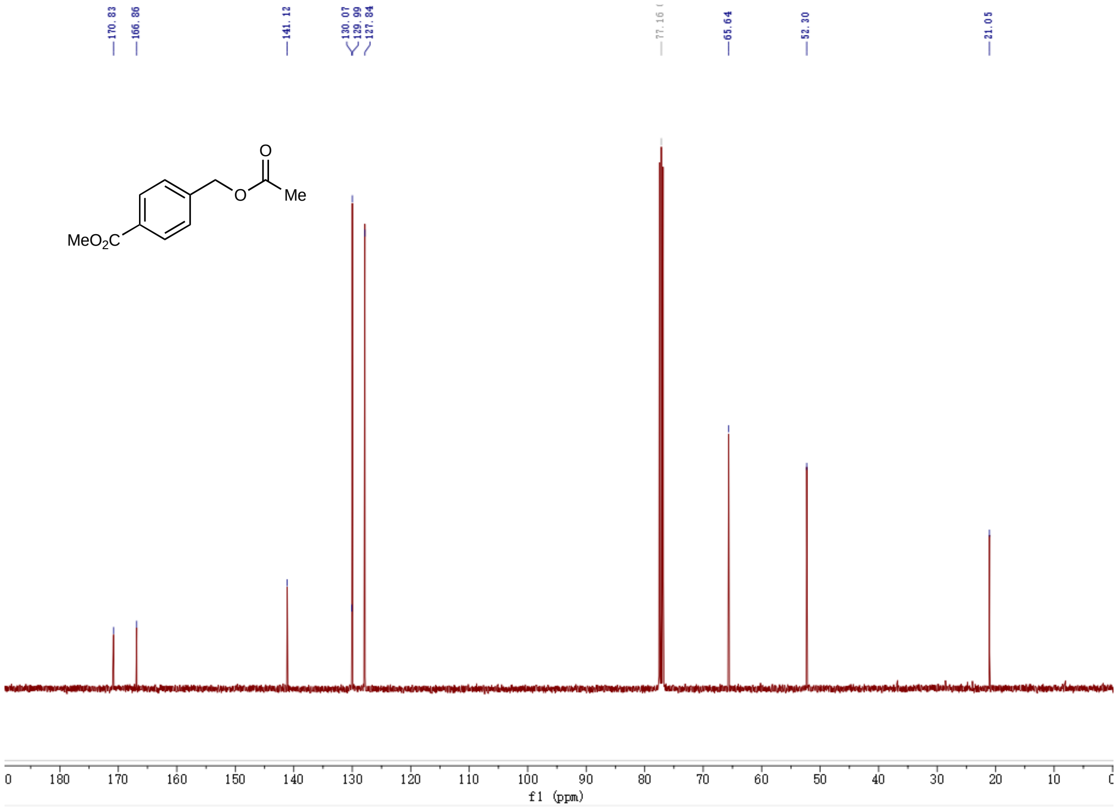

Supplementary Figure 110. ^{13}C NMR spectra of compound **3j**

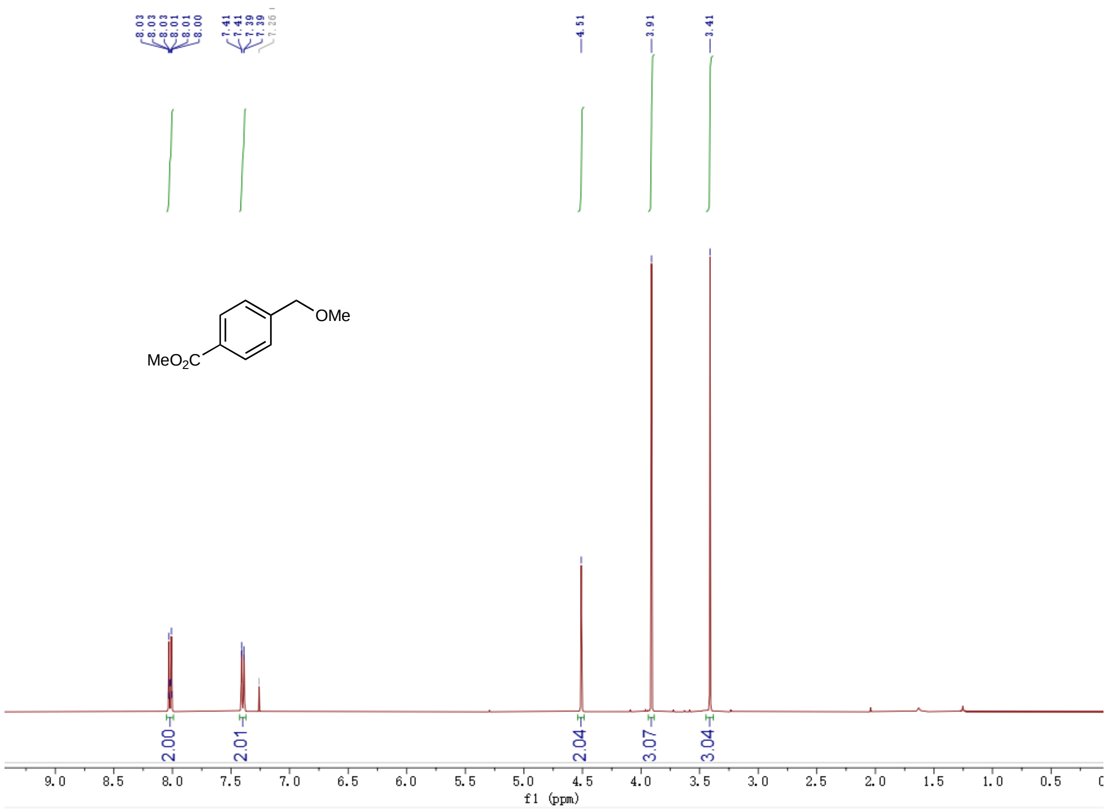

Supplementary Figure 111. ^{1}H NMR spectra of compound **3k**

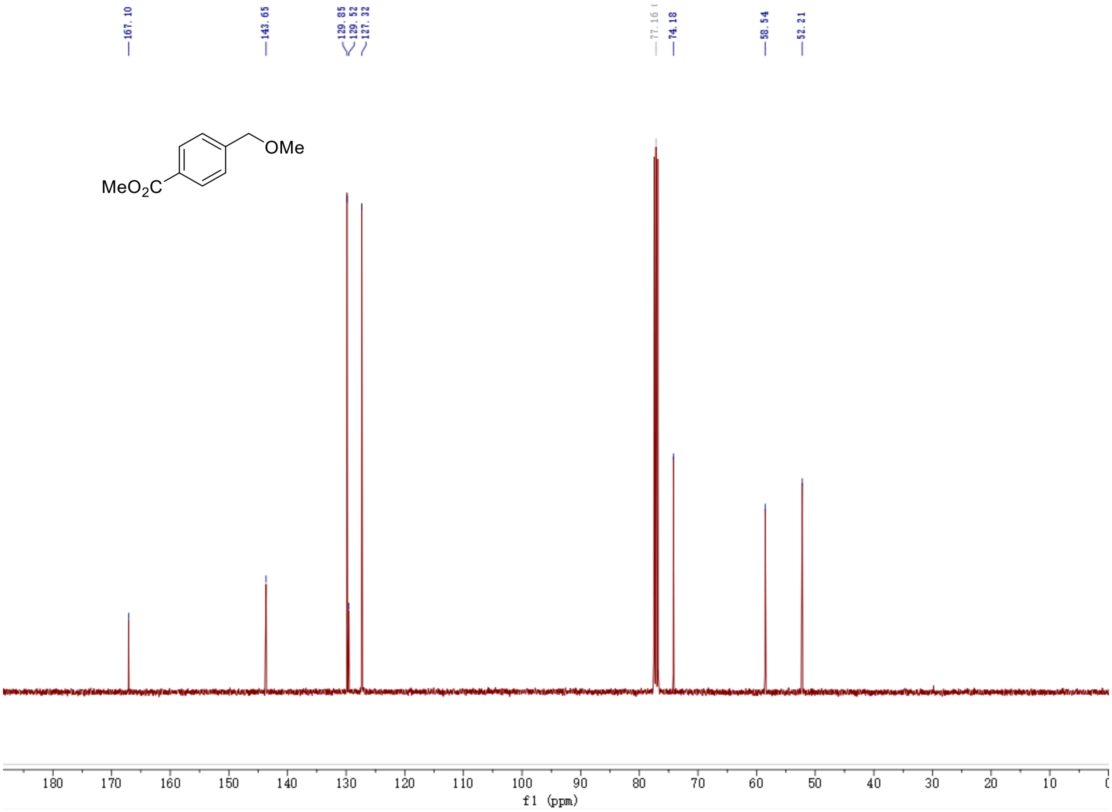

Supplementary Figure 112. ^{13}C NMR spectra of compound **3k**

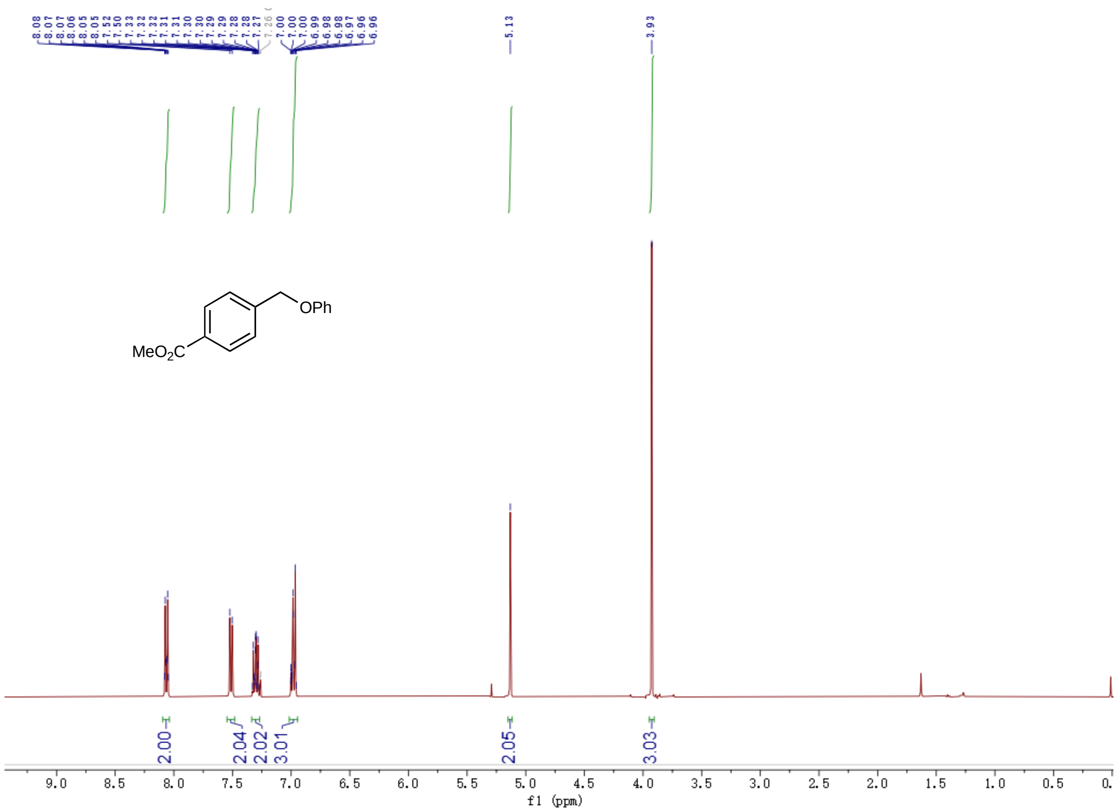

Supplementary Figure 113. ^{1}H NMR spectra of compound **3l**

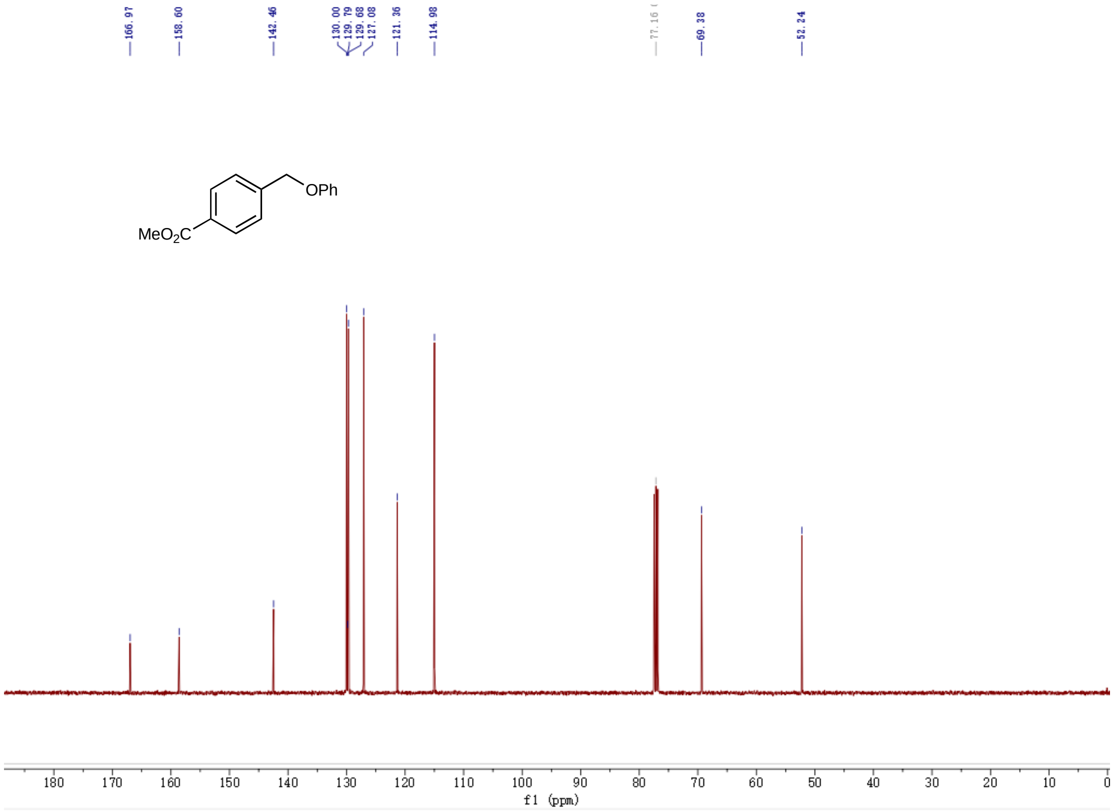

Supplementary Figure 114. ^{13}C NMR spectra of compound **3l**

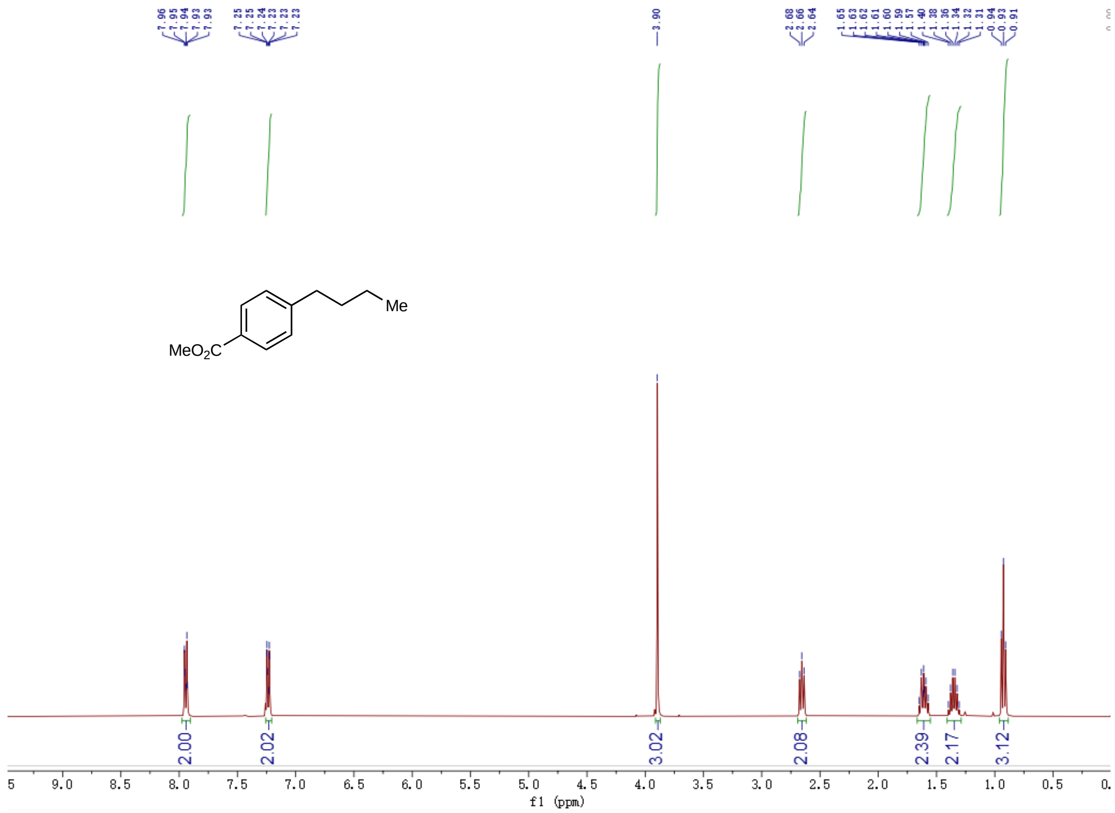

Supplementary Figure 115. ^{1}H NMR spectra of compound **3m**

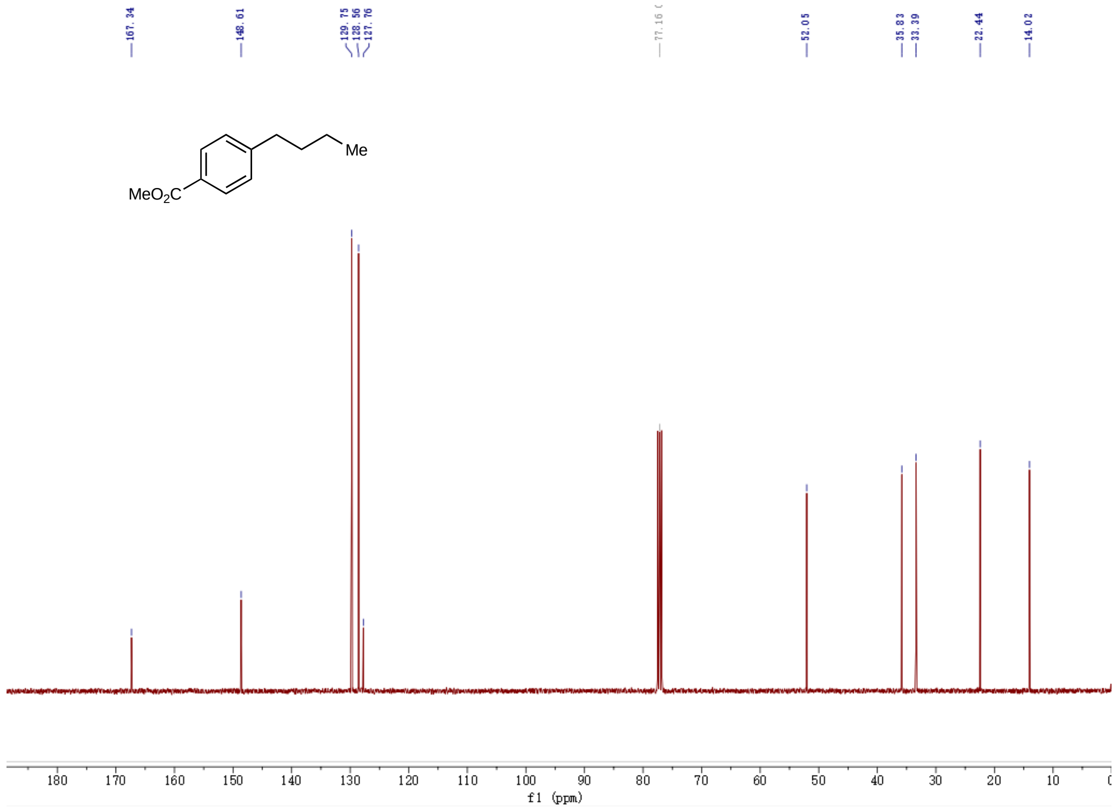

Supplementary Figure 116. ^{13}C NMR spectra of compound **3m**

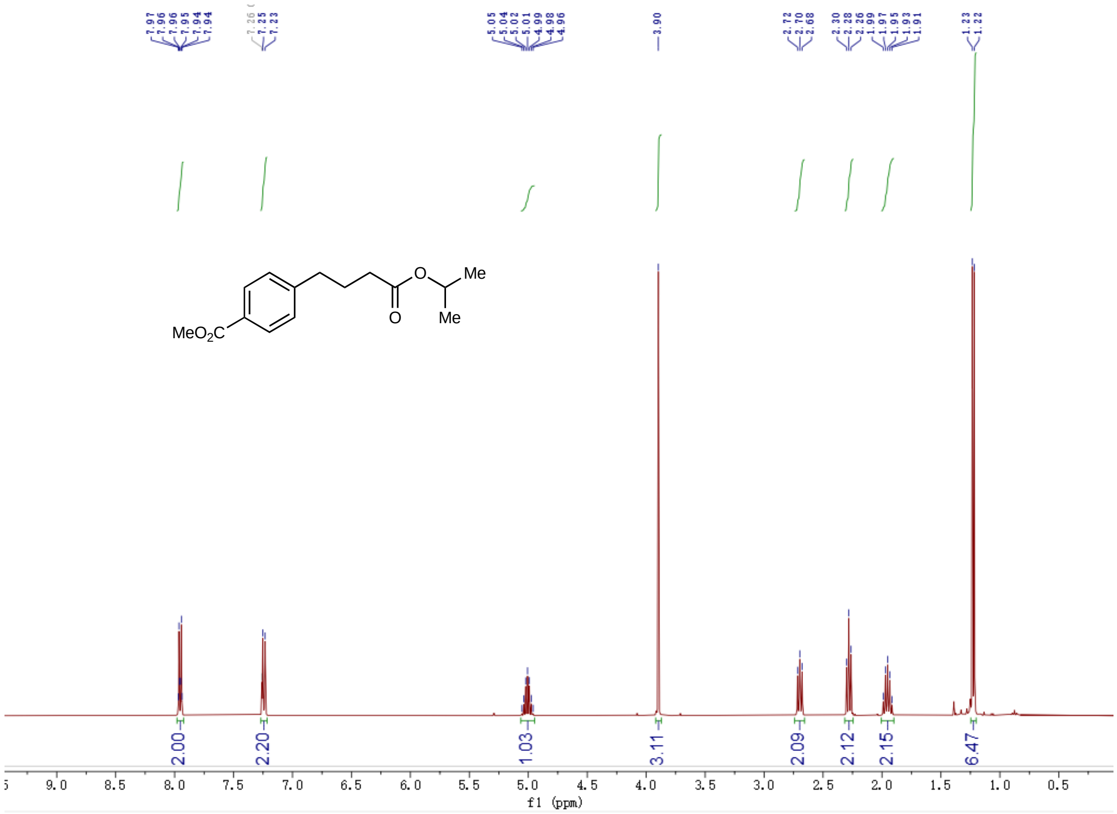

Supplementary Figure 117. ^{1}H NMR spectra of compound **3n**

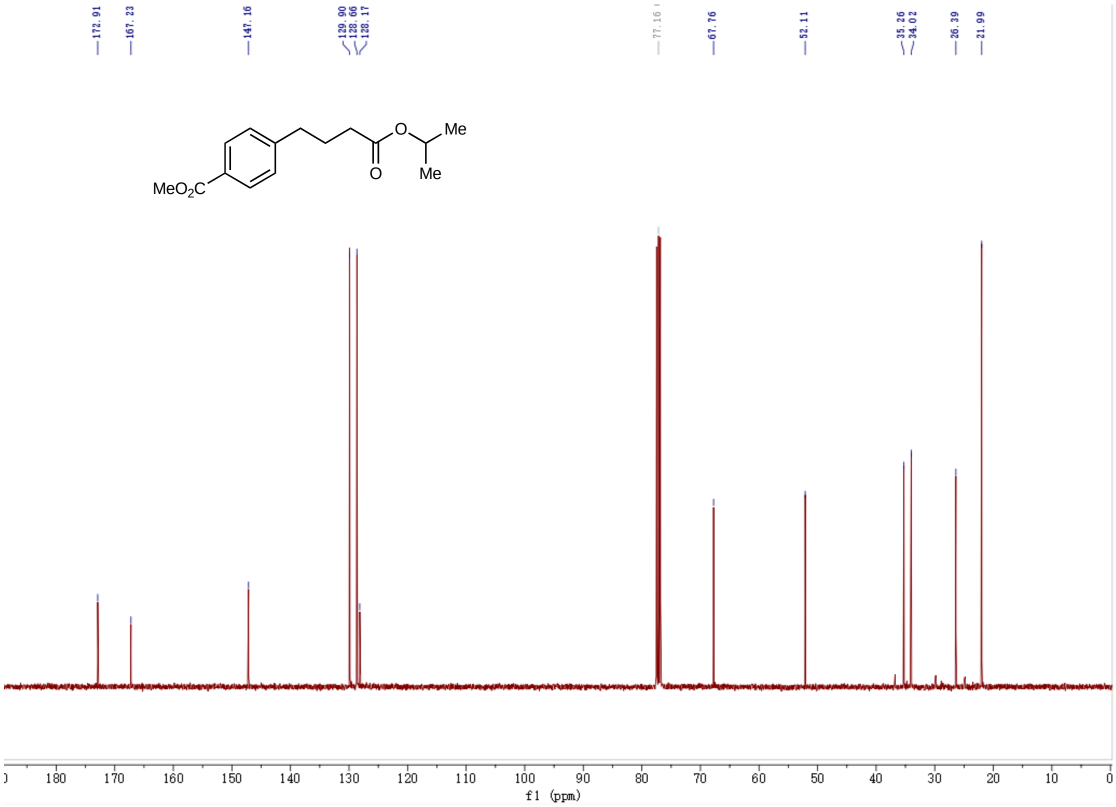

Supplementary Figure 118. ^{13}C NMR spectra of compound **3n**

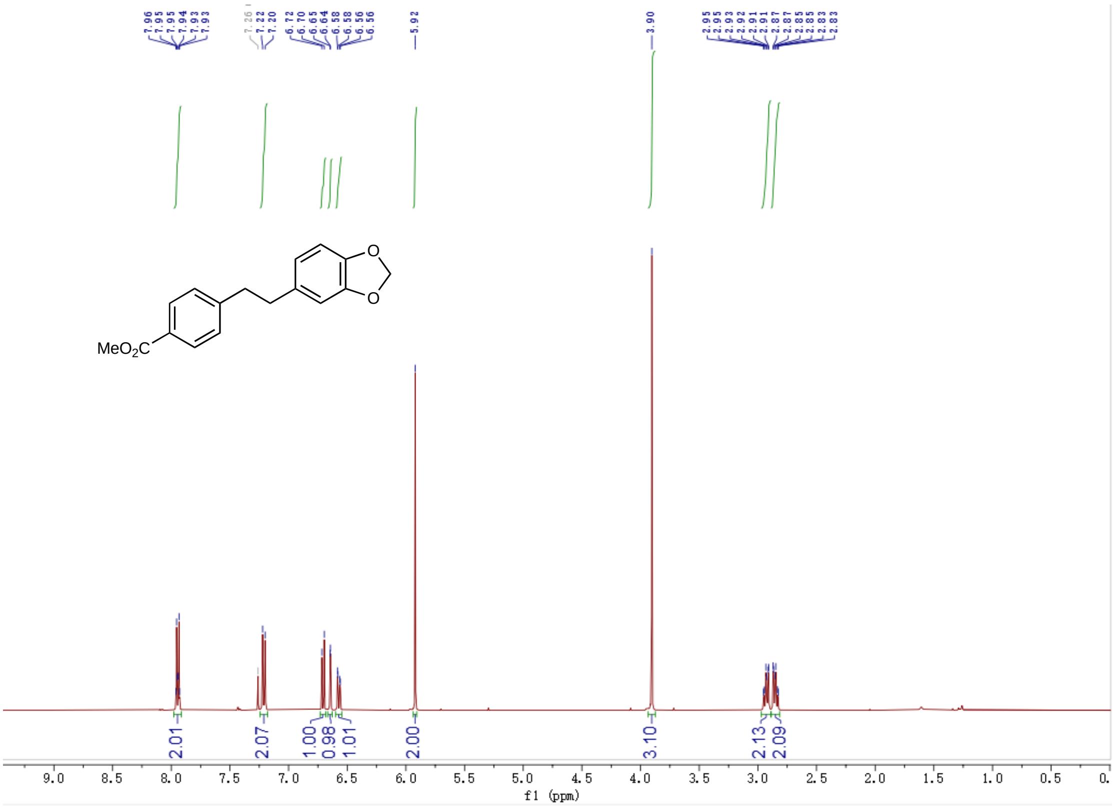

Supplementary Figure 119. ^{1}H NMR spectra of compound **3o**

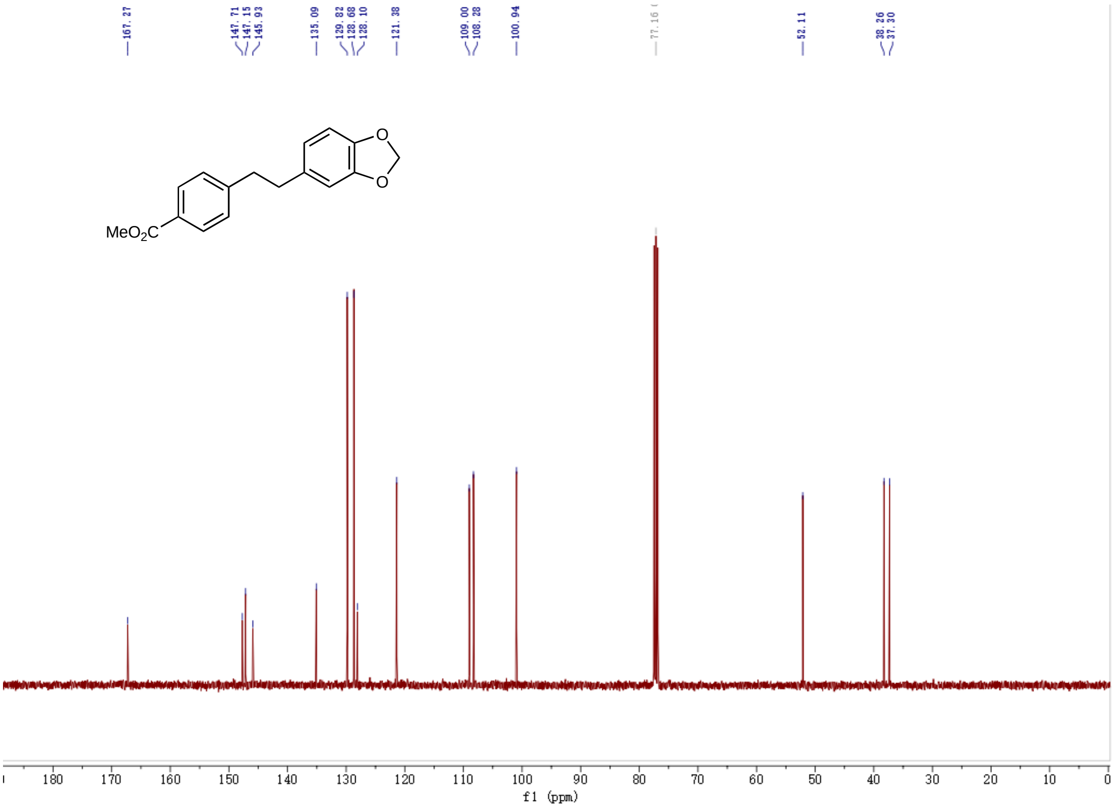

Supplementary Figure 120. ^{13}C NMR spectra of compound **3o**

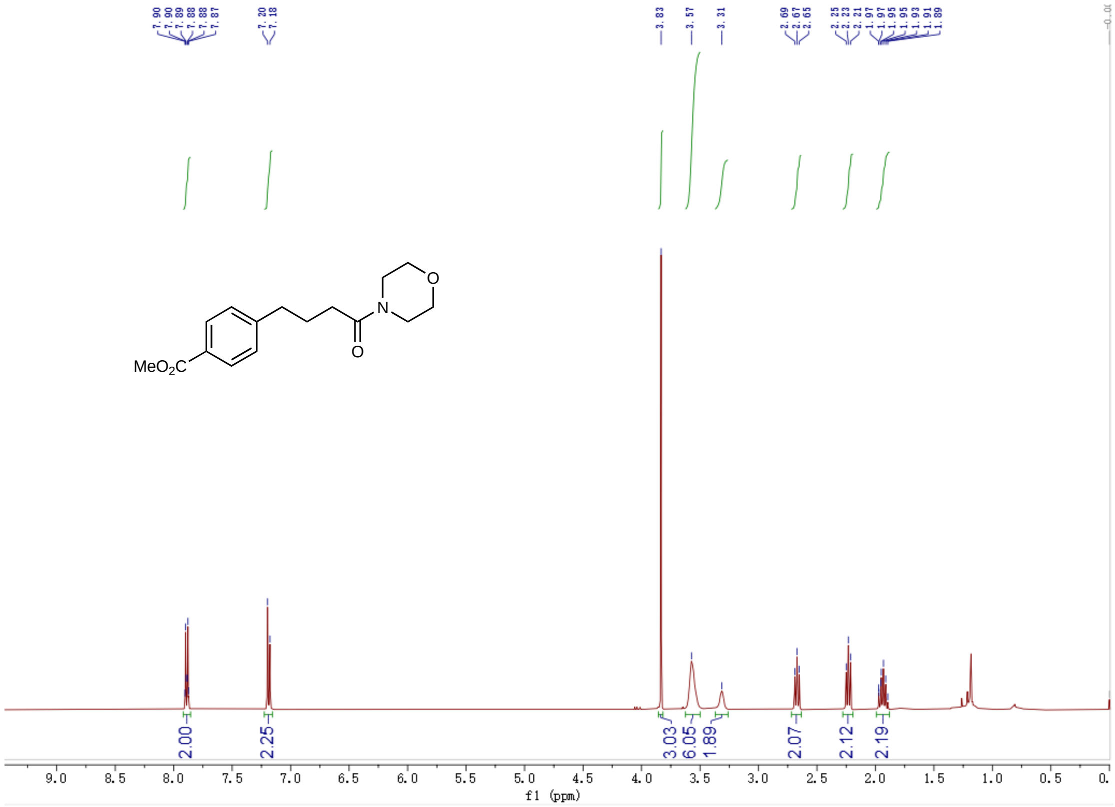

Supplementary Figure 121. ^{1}H NMR spectra of compound **3p**

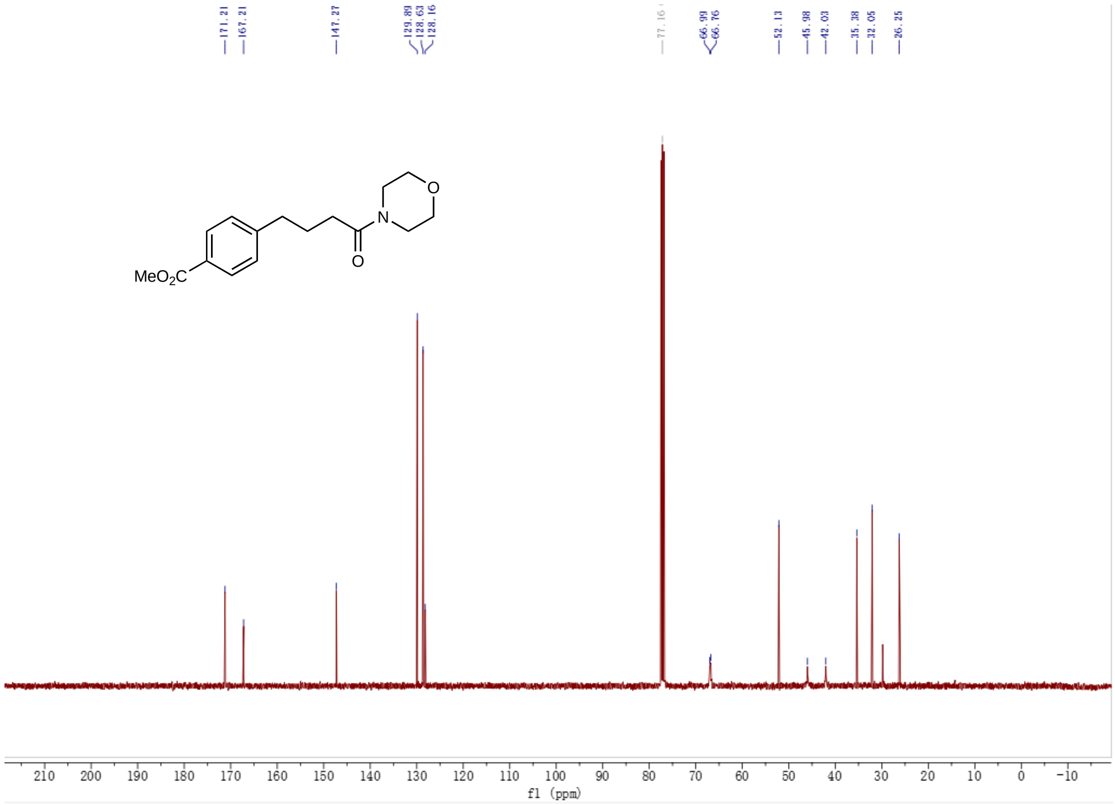

Supplementary Figure 122. ^{13}C NMR spectra of compound **3p**

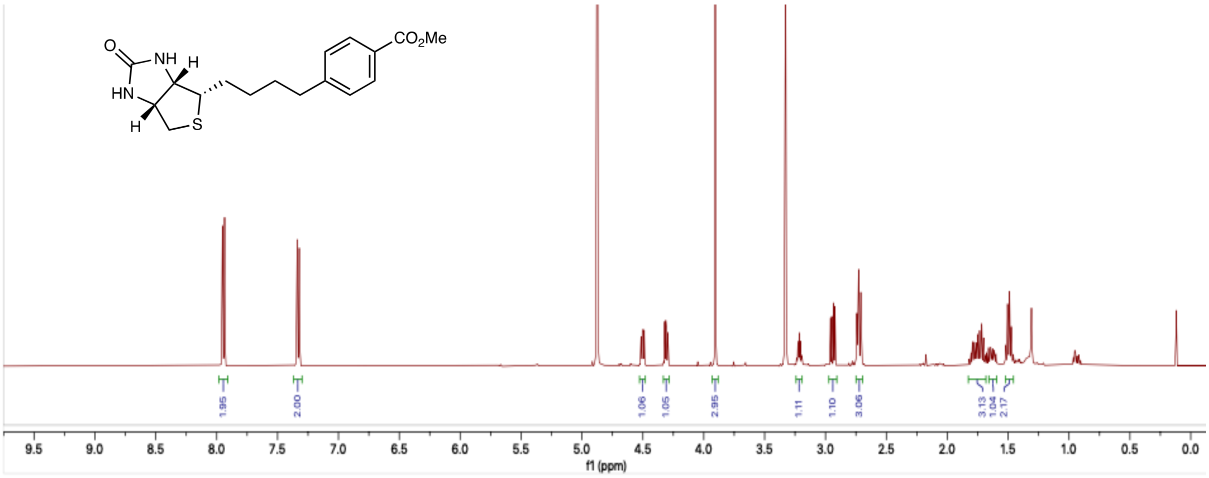

Supplementary Figure 123. ^{1}H NMR spectra of compound **3q**

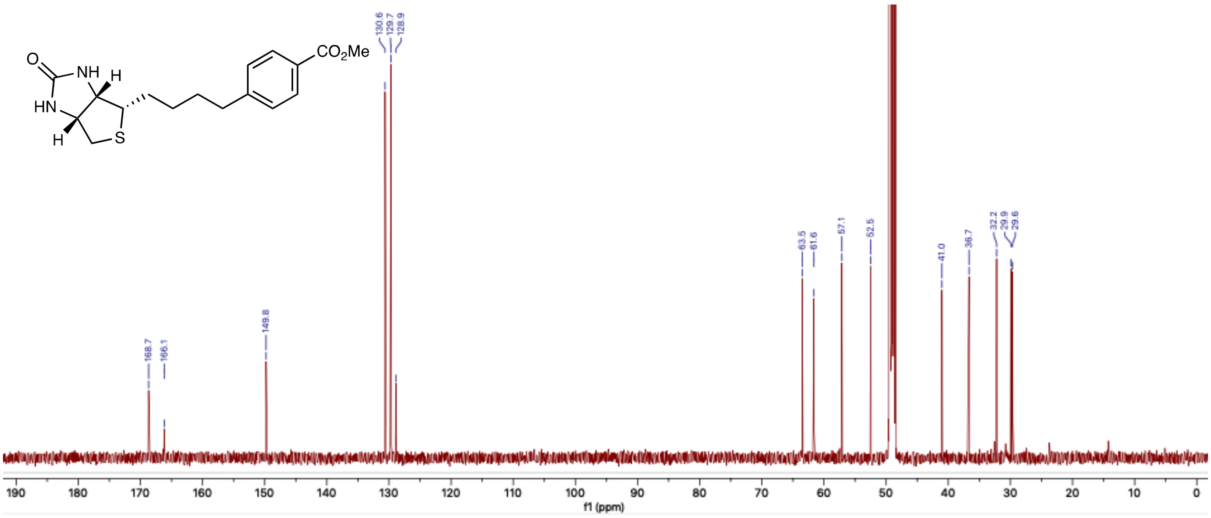

Supplementary Figure 124. ^{13}C NMR spectra of compound **3q**

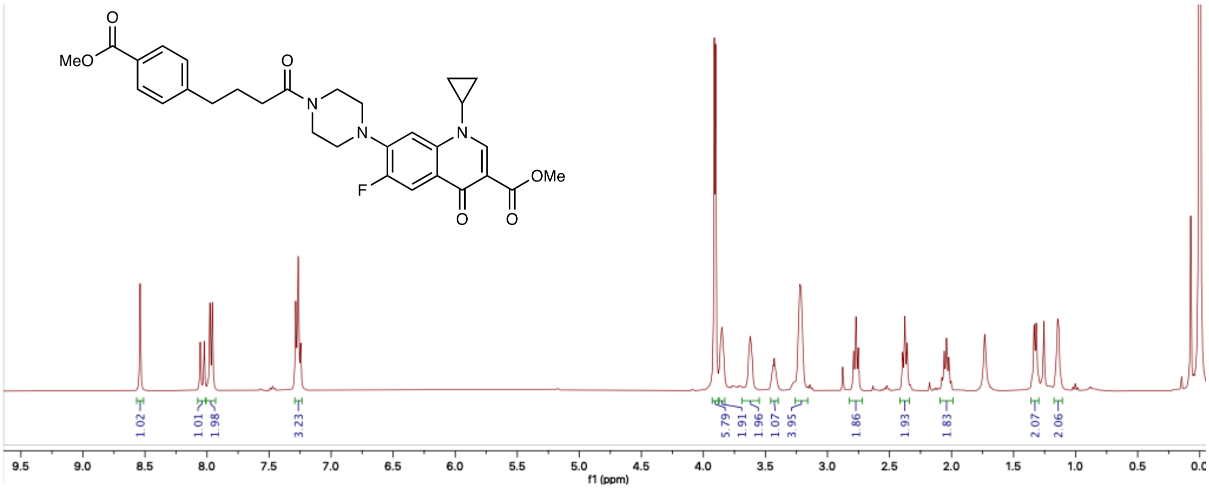

Supplementary Figure 125. ^{1}H NMR spectra of compound **3r**

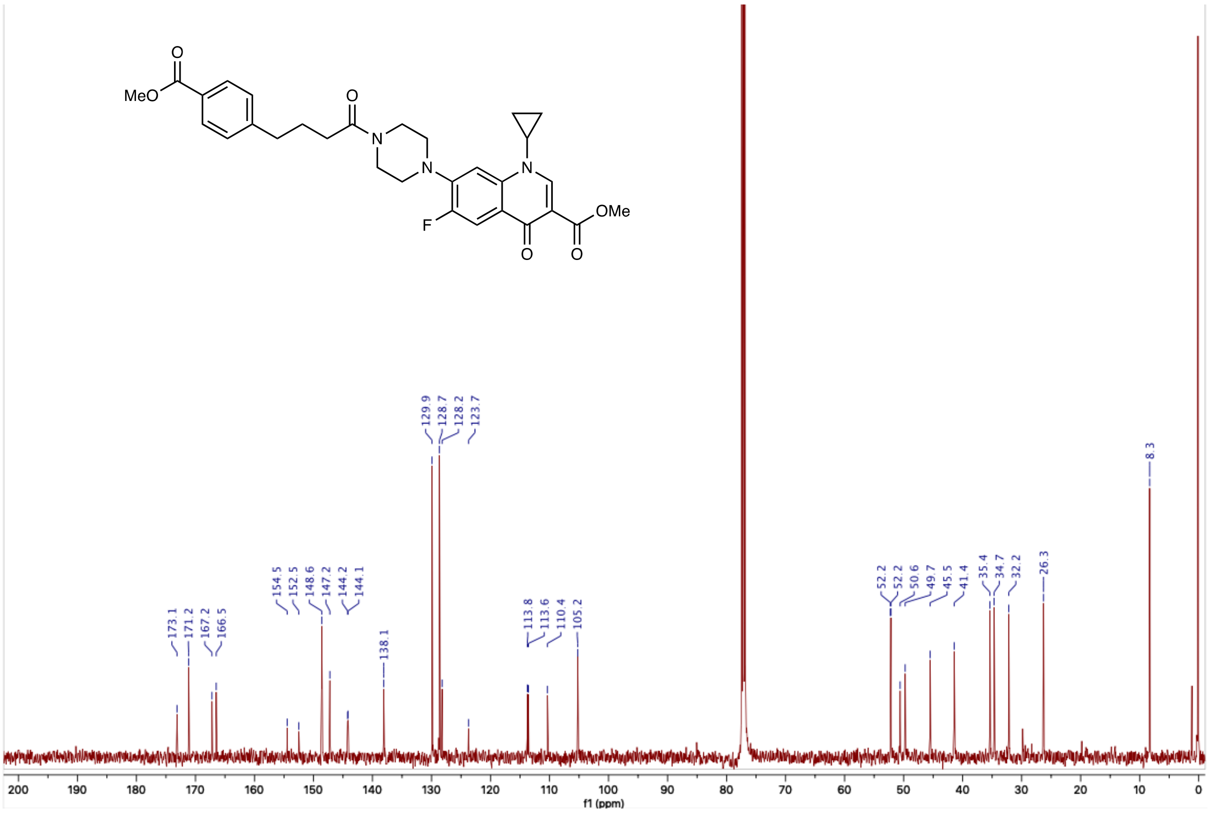

Supplementary Figure 126. ^{13}C NMR spectra of compound **3r**

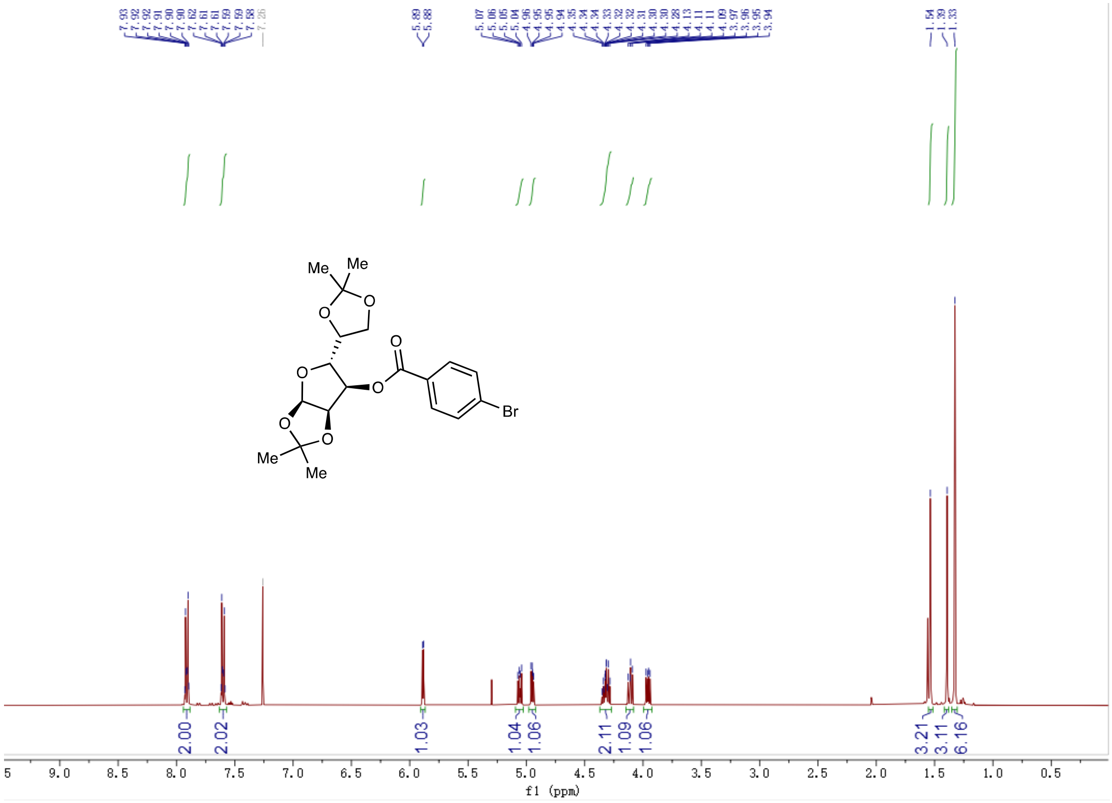

Supplementary Figure 127. ^{1}H NMR spectra of compound **4o**

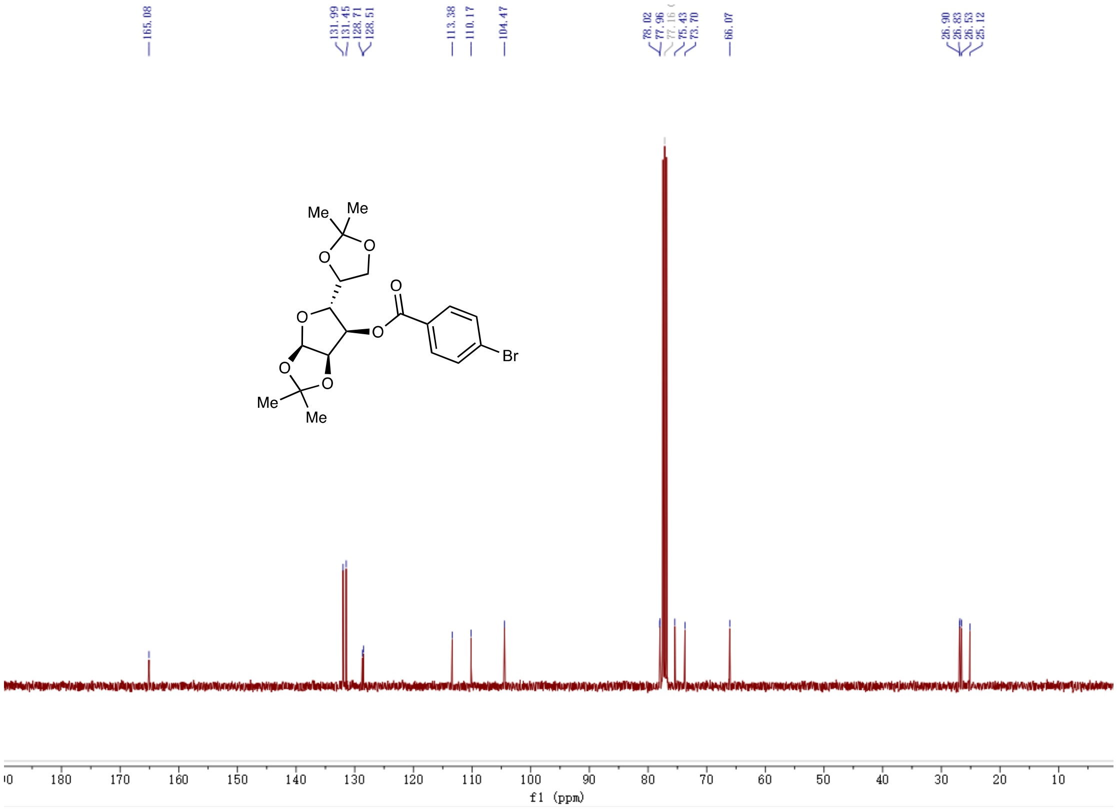

Supplementary Figure 128. ^{13}C NMR spectra of compound **4o**

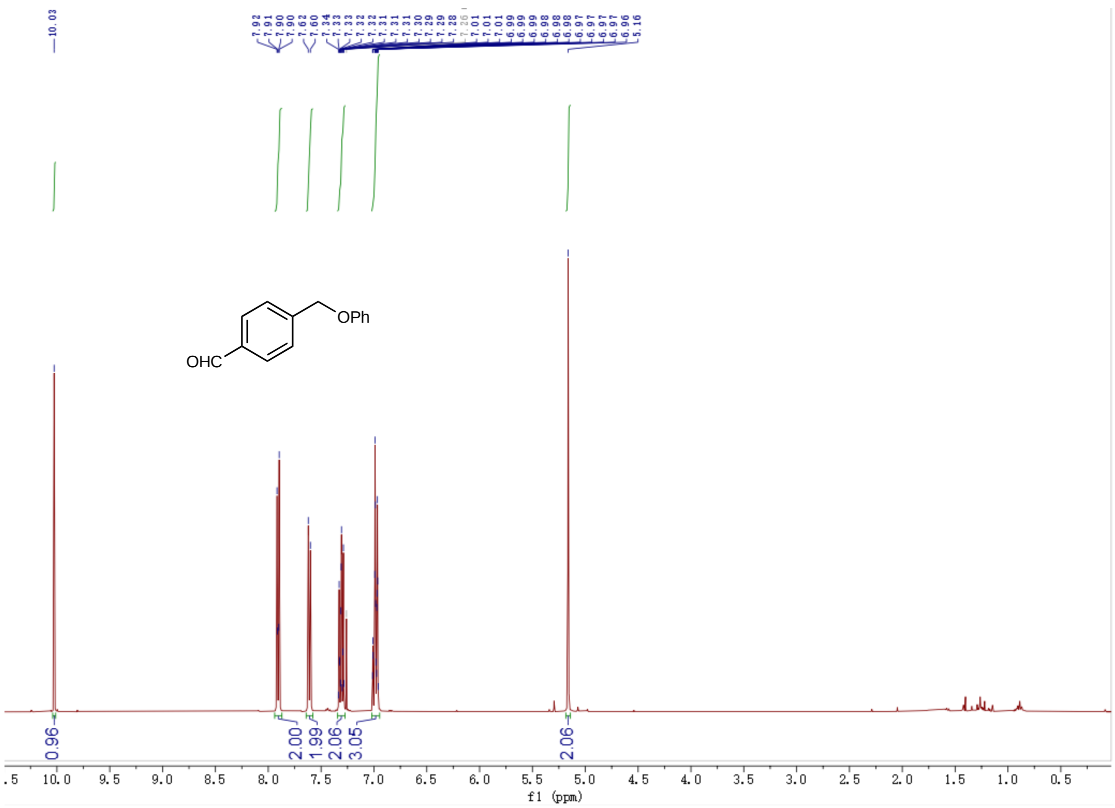

Supplementary Figure 129. ^{1}H NMR spectra of compound **5a**

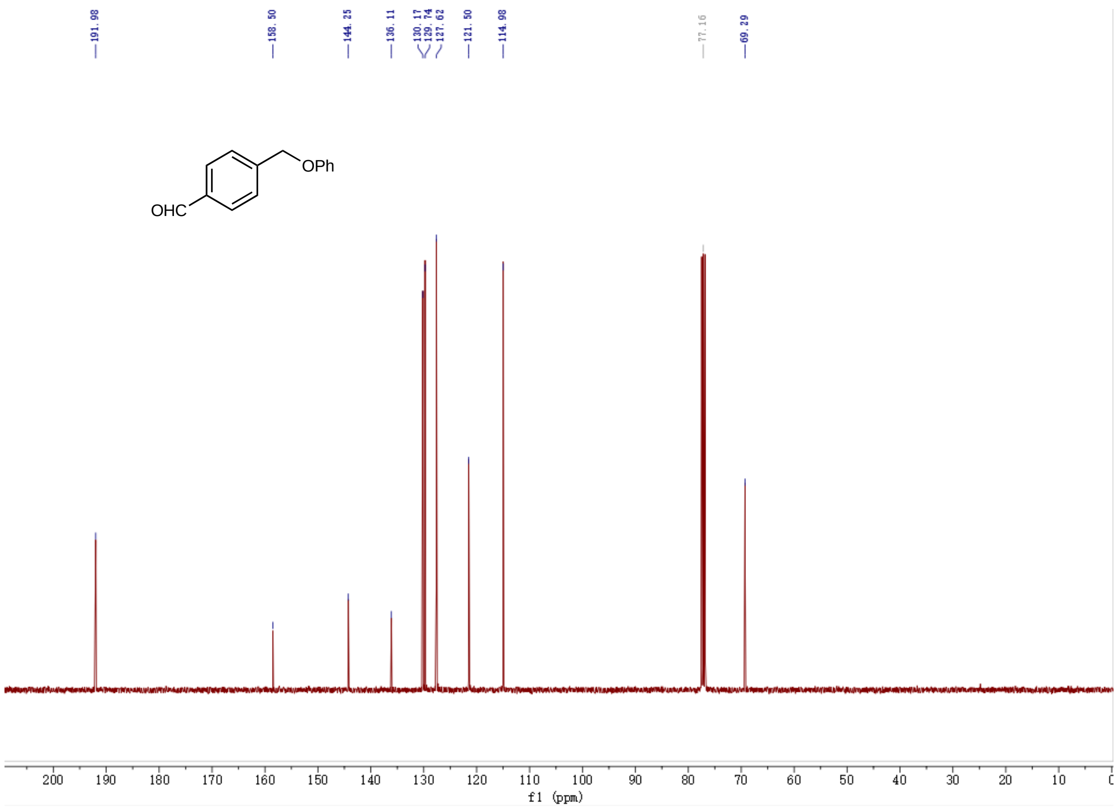

Supplementary Figure 130. ^{13}C NMR spectra of compound **5a**

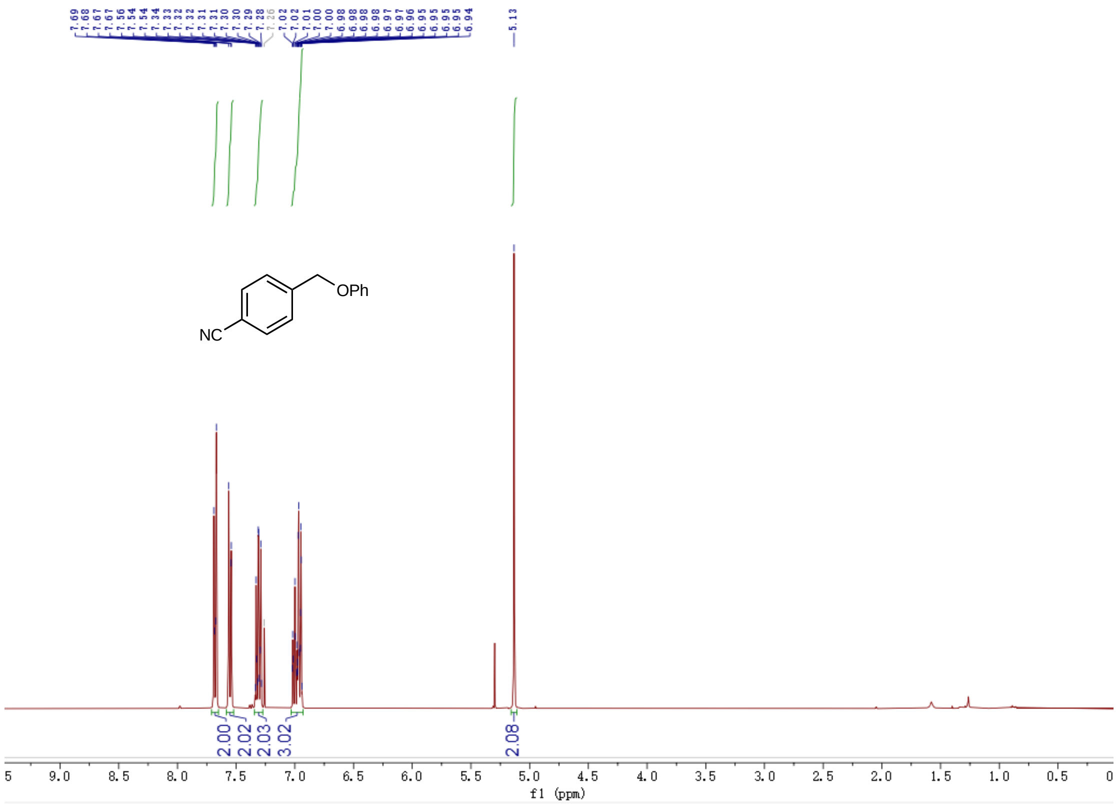

Supplementary Figure 131. ^{1}H NMR spectra of compound **5b**

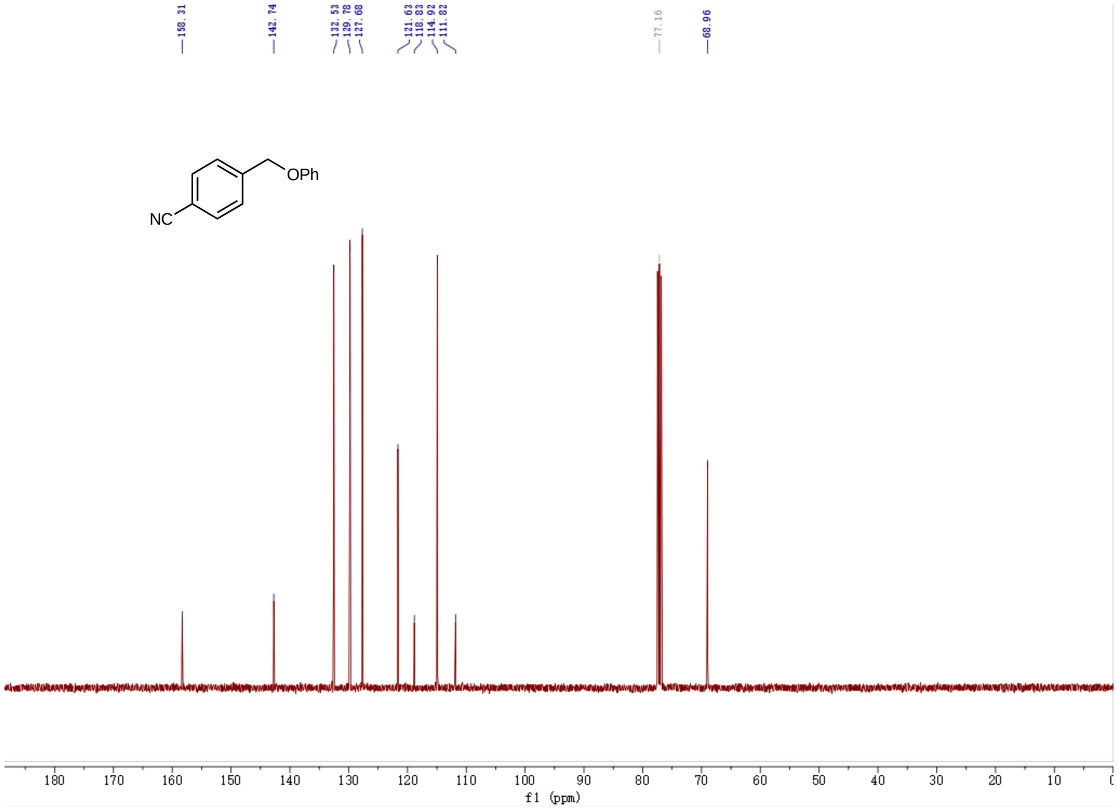

Supplementary Figure 132. ^{13}C NMR spectra of compound **5b**

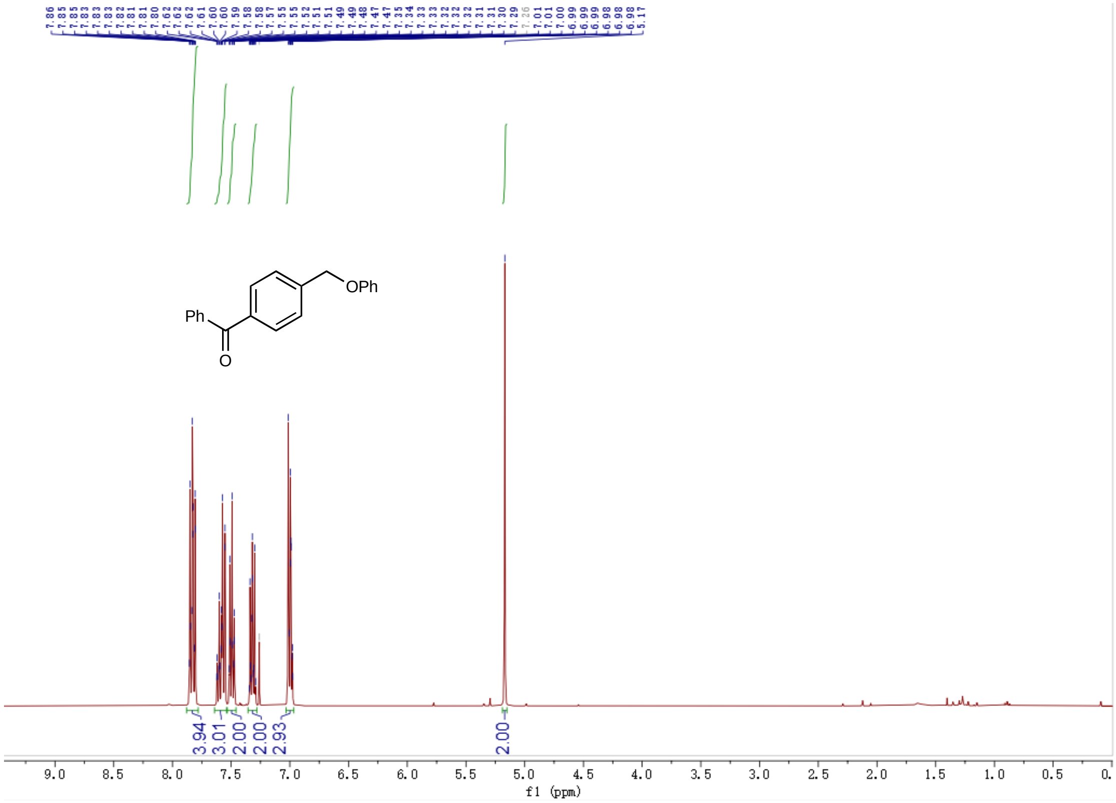

Supplementary Figure 133. ^{1}H NMR spectra of compound **5c**

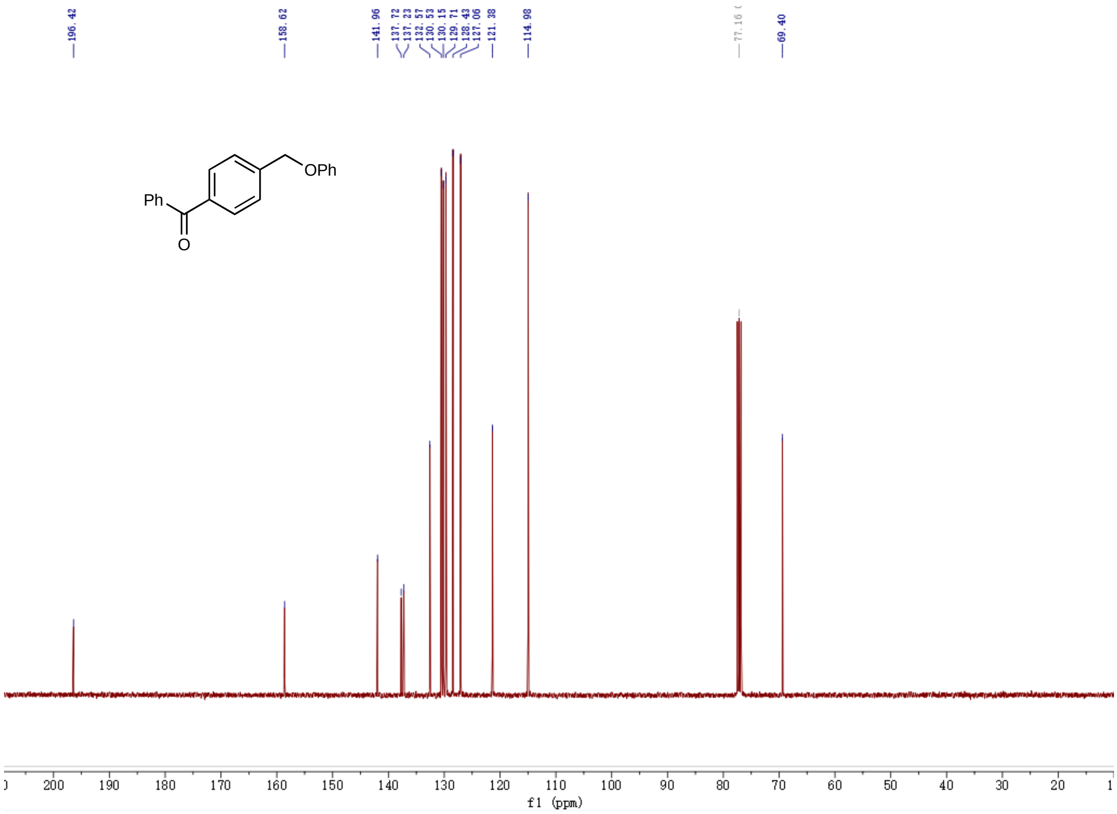

Supplementary Figure 134. ^{13}C NMR spectra of compound **5c**

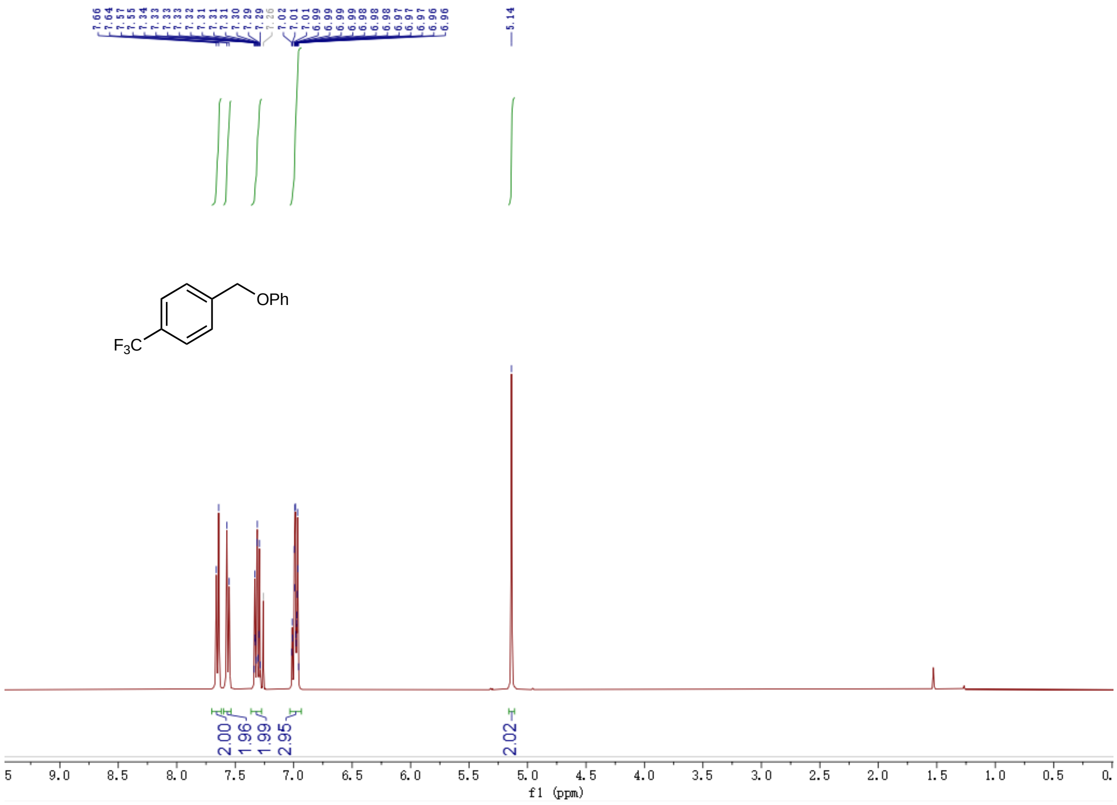

Supplementary Figure 135. ^{1}H NMR spectra of compound **5d**

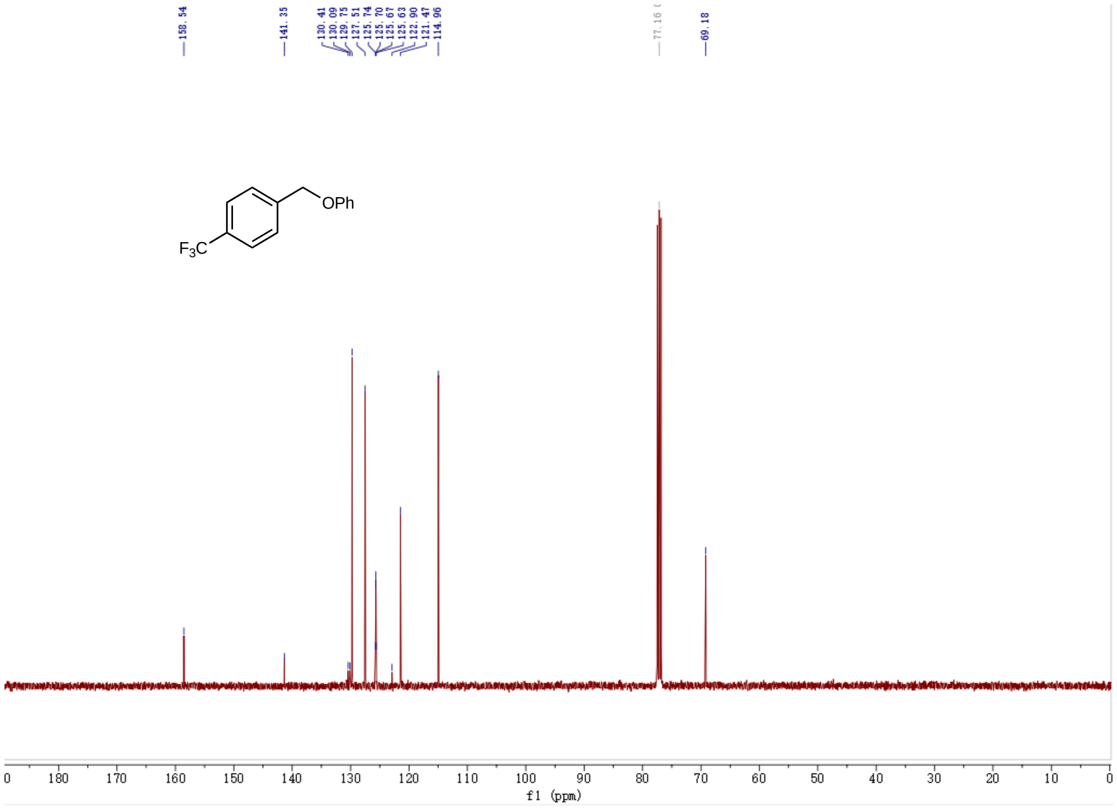

Supplementary Figure 136. ^{13}C NMR spectra of compound **5d**

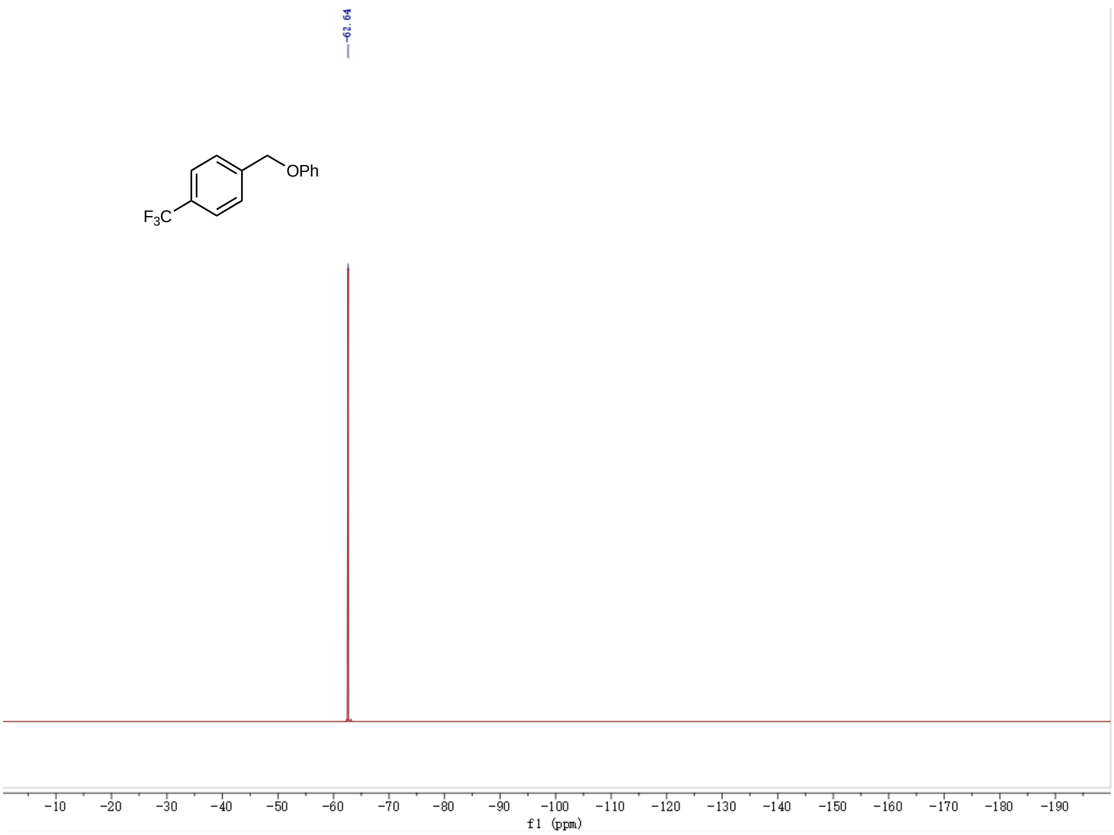

Supplementary Figure 137. ^{19}F NMR spectra of compound **5d**

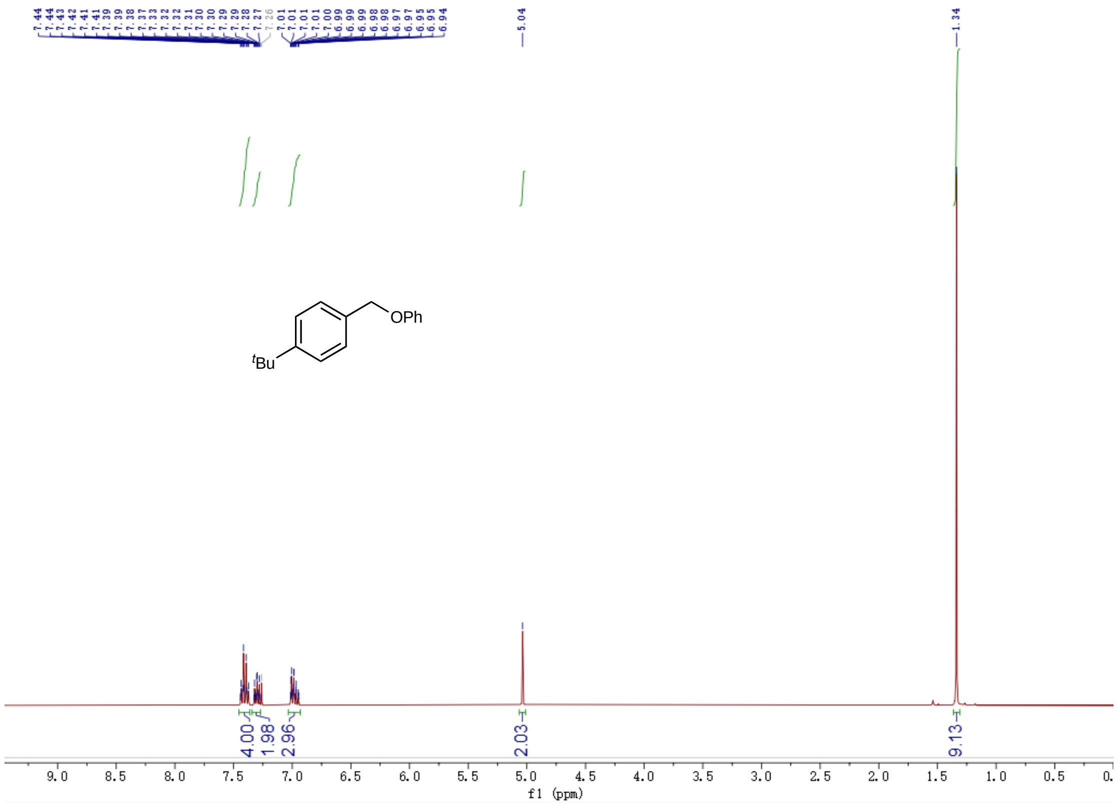

Supplementary Figure 138. ^{1}H NMR spectra of compound **5e**

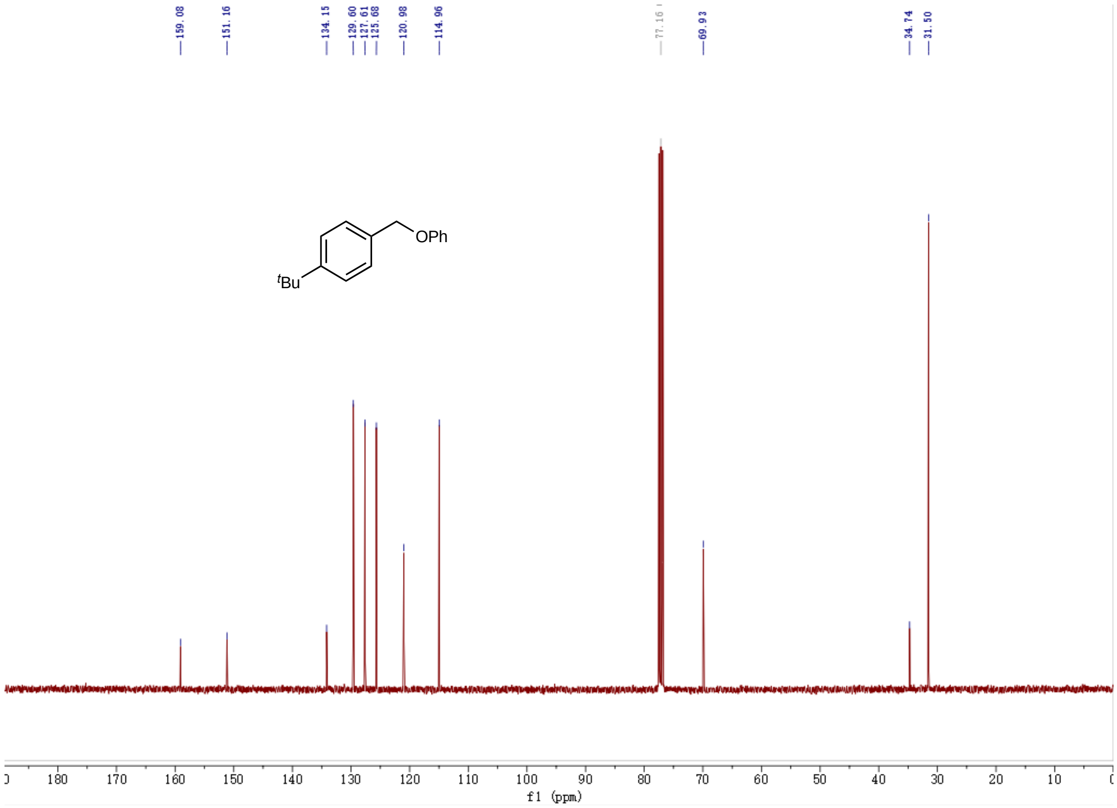

Supplementary Figure 139. ^{13}C NMR spectra of compound **5e**

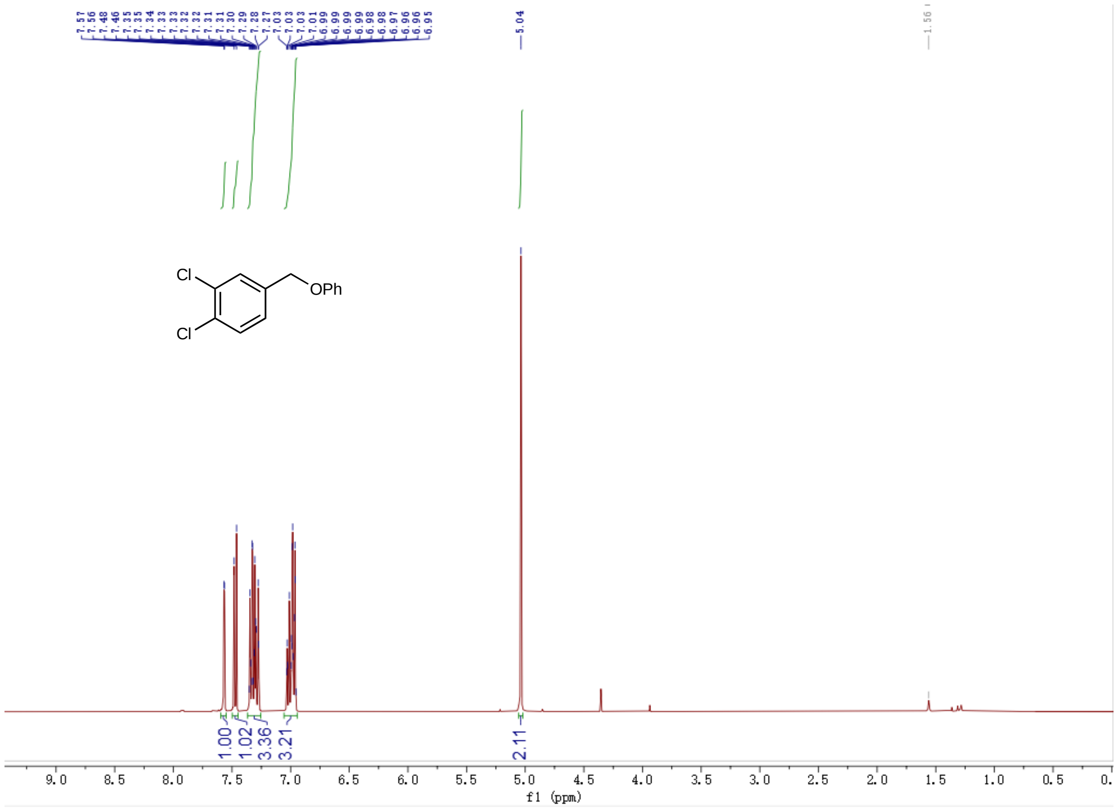

Supplementary Figure 140. ^{1}H NMR spectra of compound **5f**

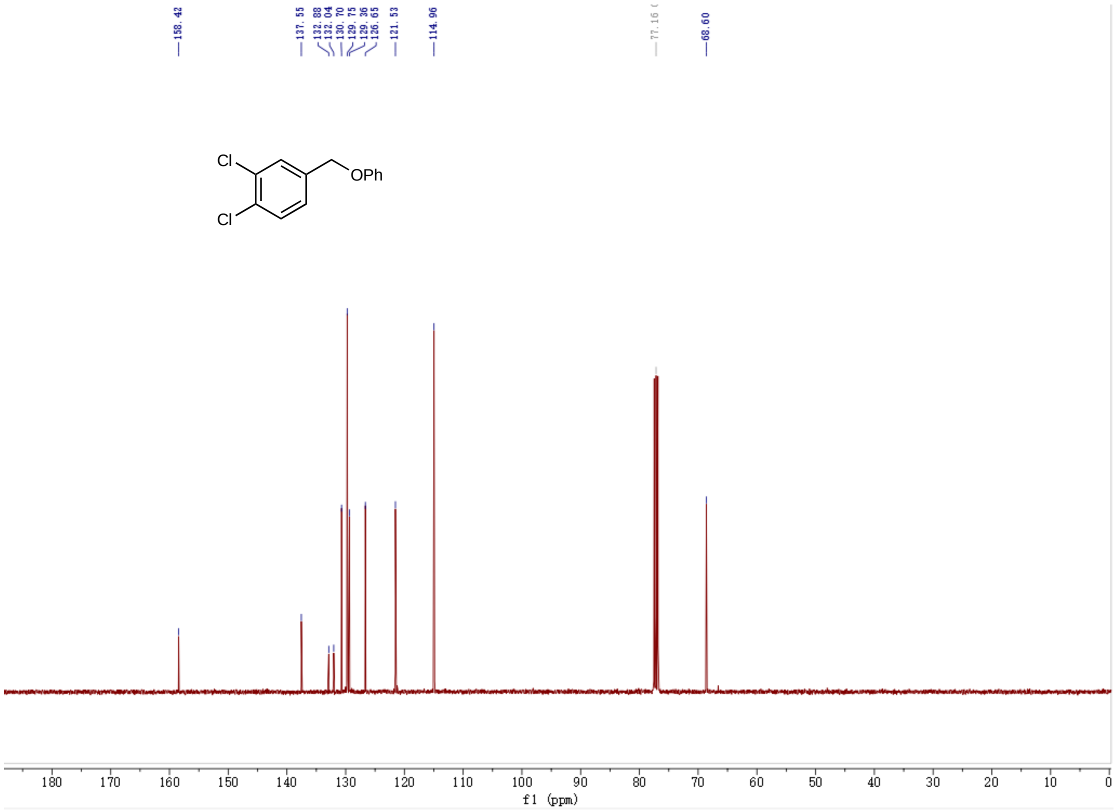

Supplementary Figure 141. ^{13}C NMR spectra of compound **5f**

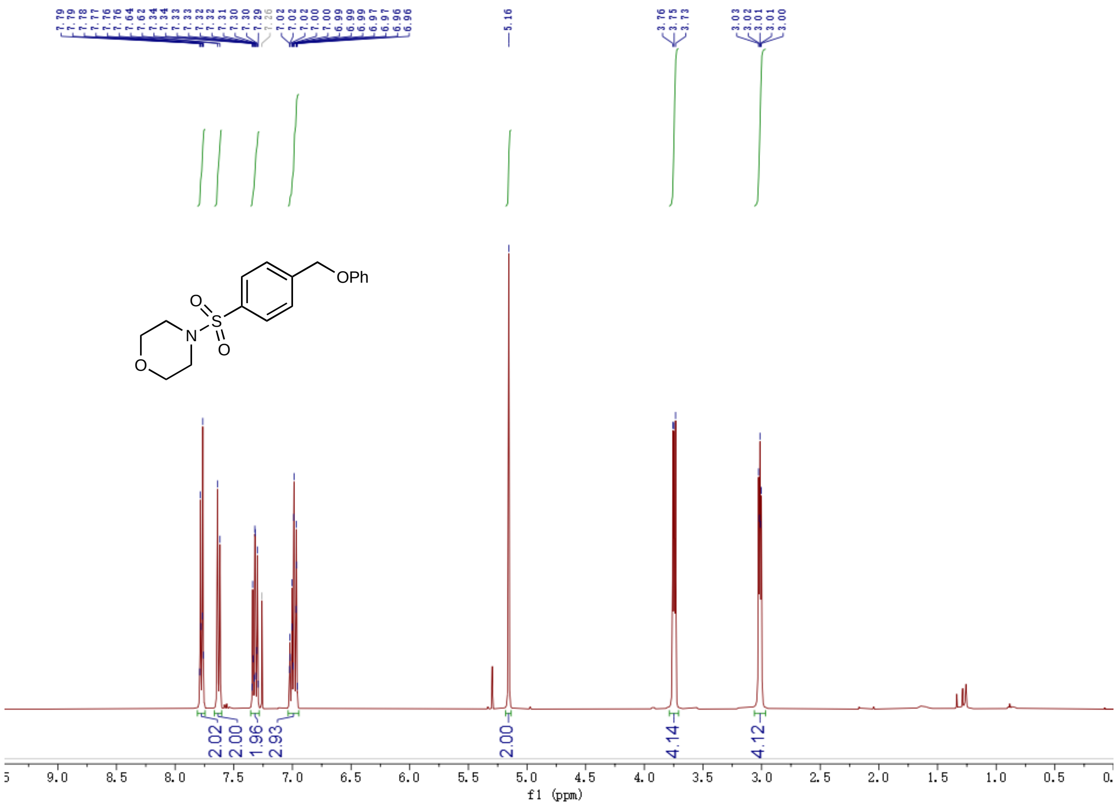

Supplementary Figure 142. ^{1}H NMR spectra of compound **5g**

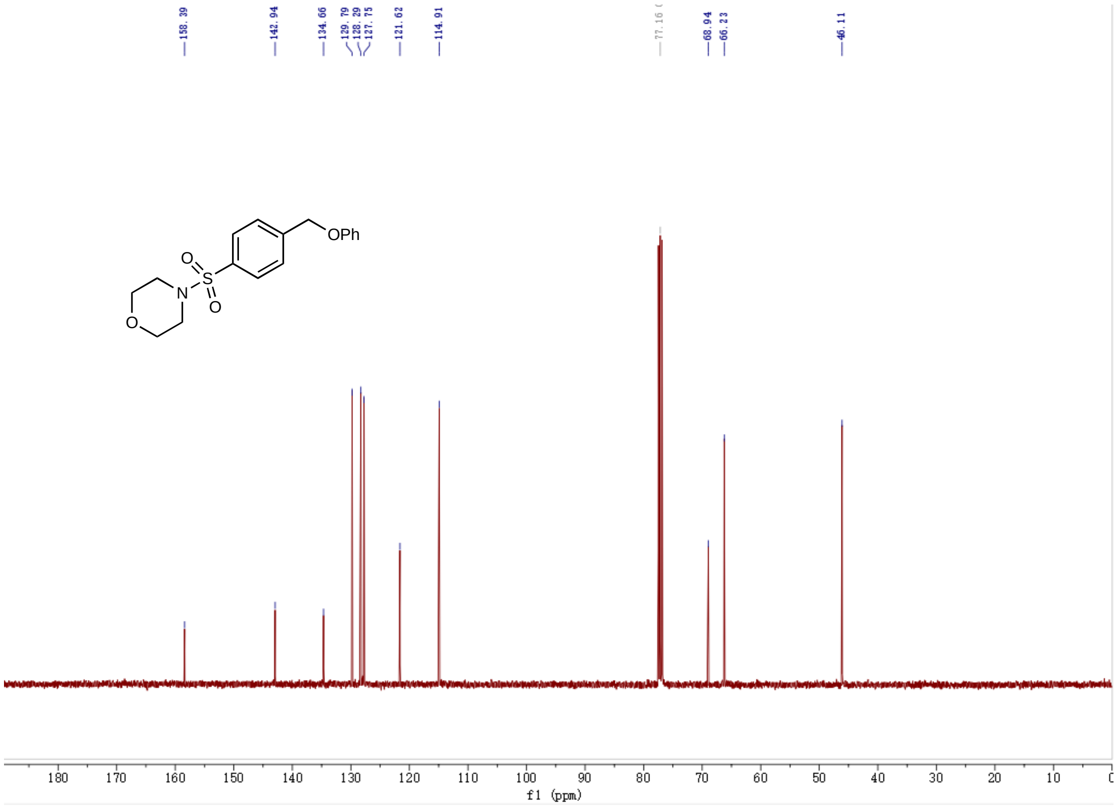

Supplementary Figure 143. ^{13}C NMR spectra of compound **5g**

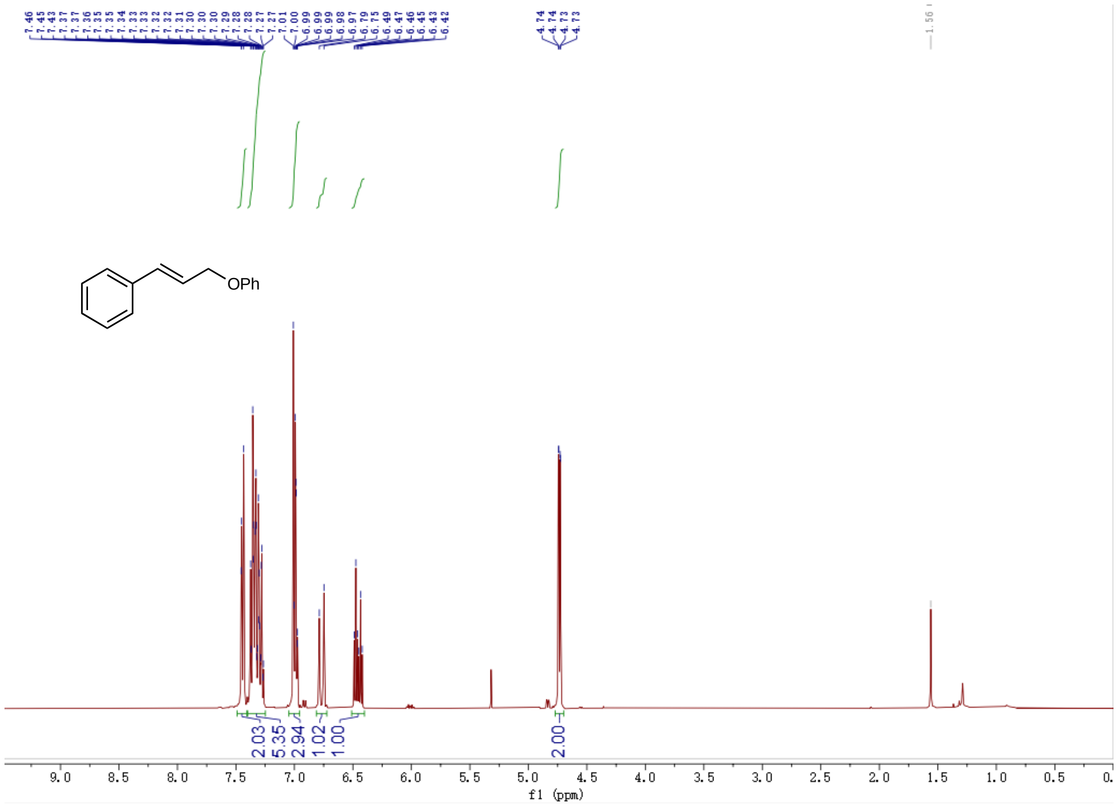

Supplementary Figure 144. ^{1}H NMR spectra of compound **5h**

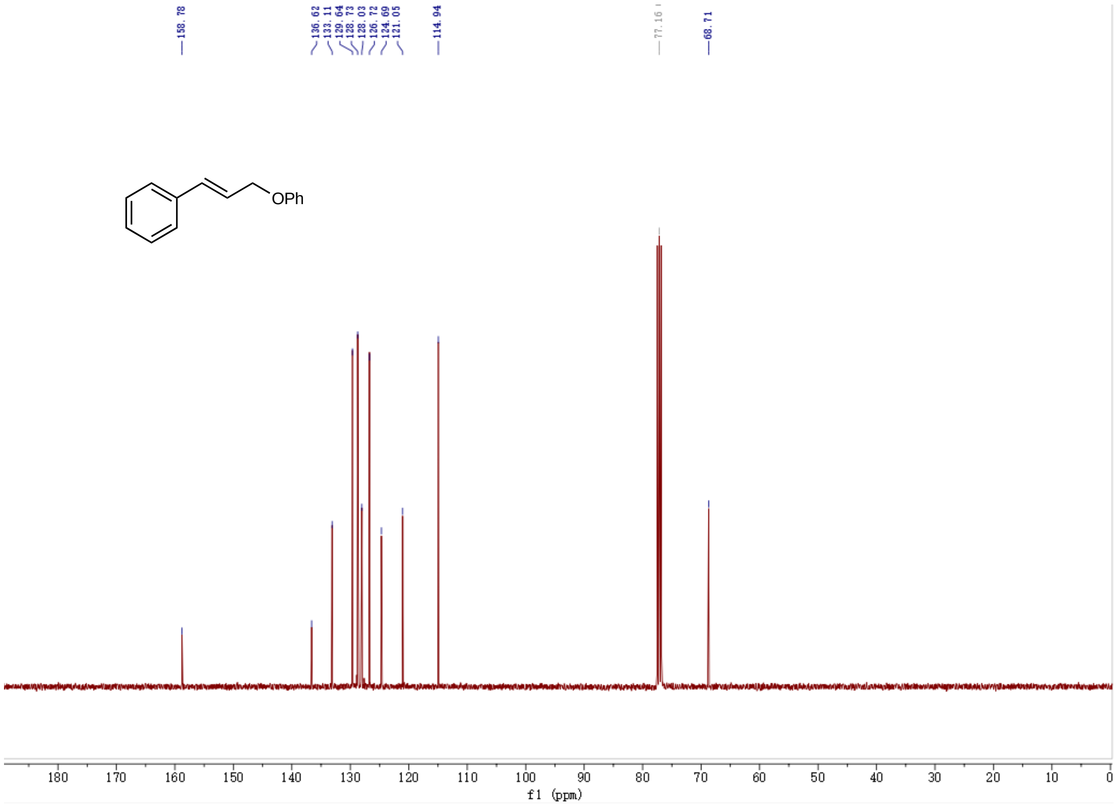

Supplementary Figure 145. ^{13}C NMR spectra of compound **5h**

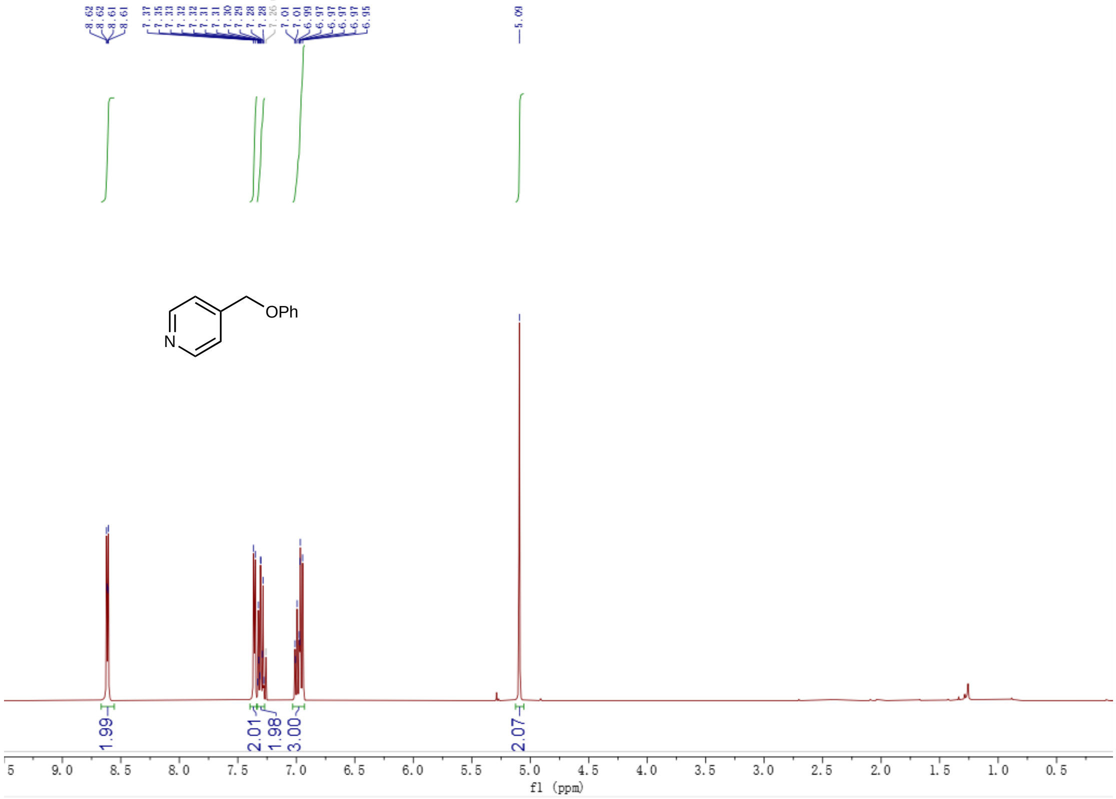

Supplementary Figure 146. ^{1}H NMR spectra of compound **5i**

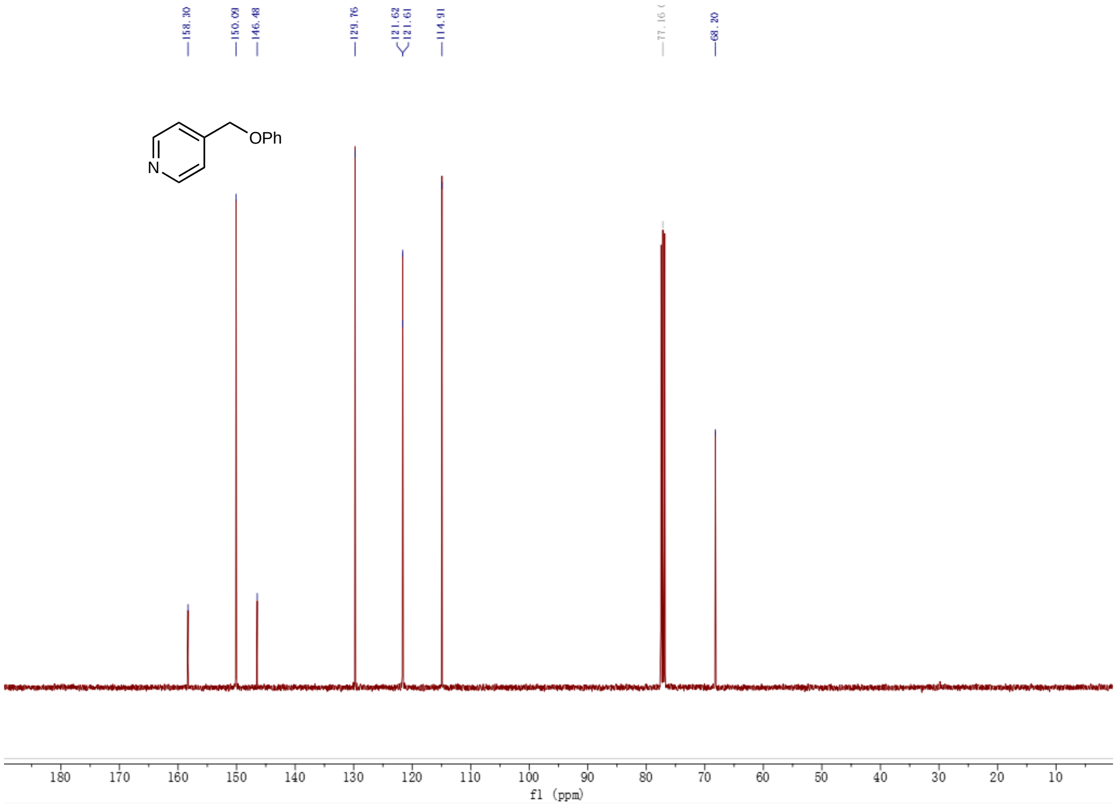

Supplementary Figure 147. ^{13}C NMR spectra of compound **5i**

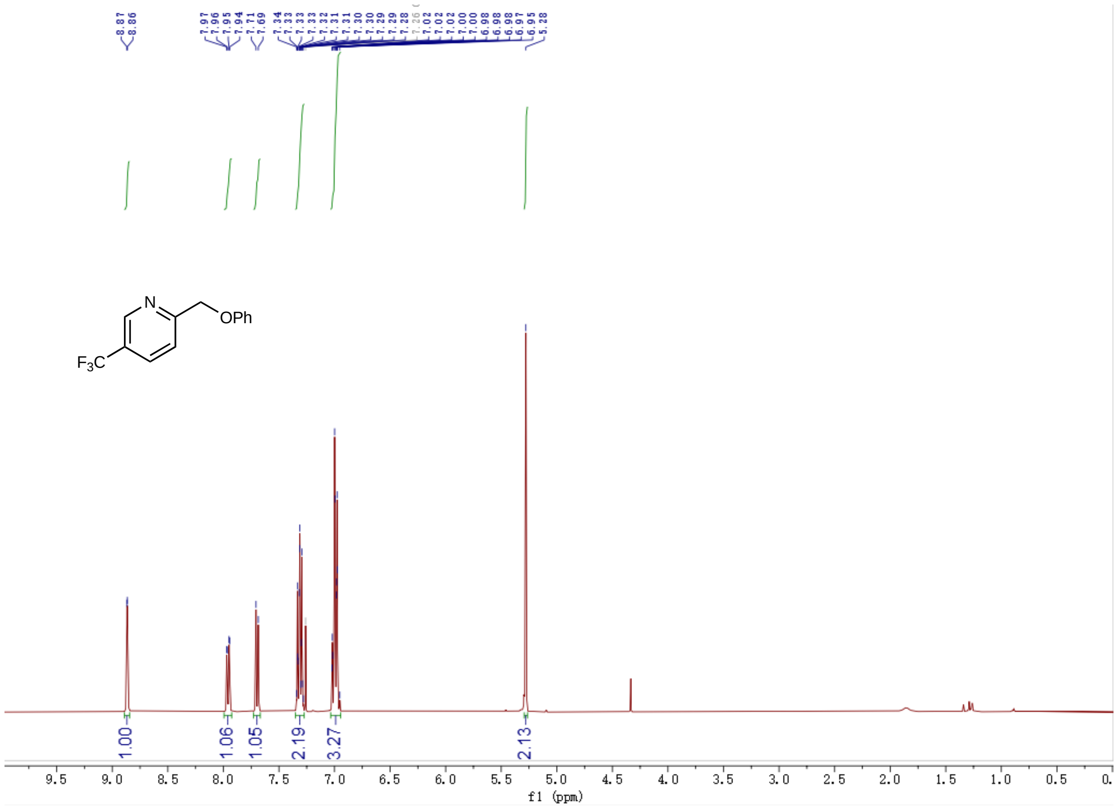

Supplementary Figure 148. ^{1}H NMR spectra of compound **5j**

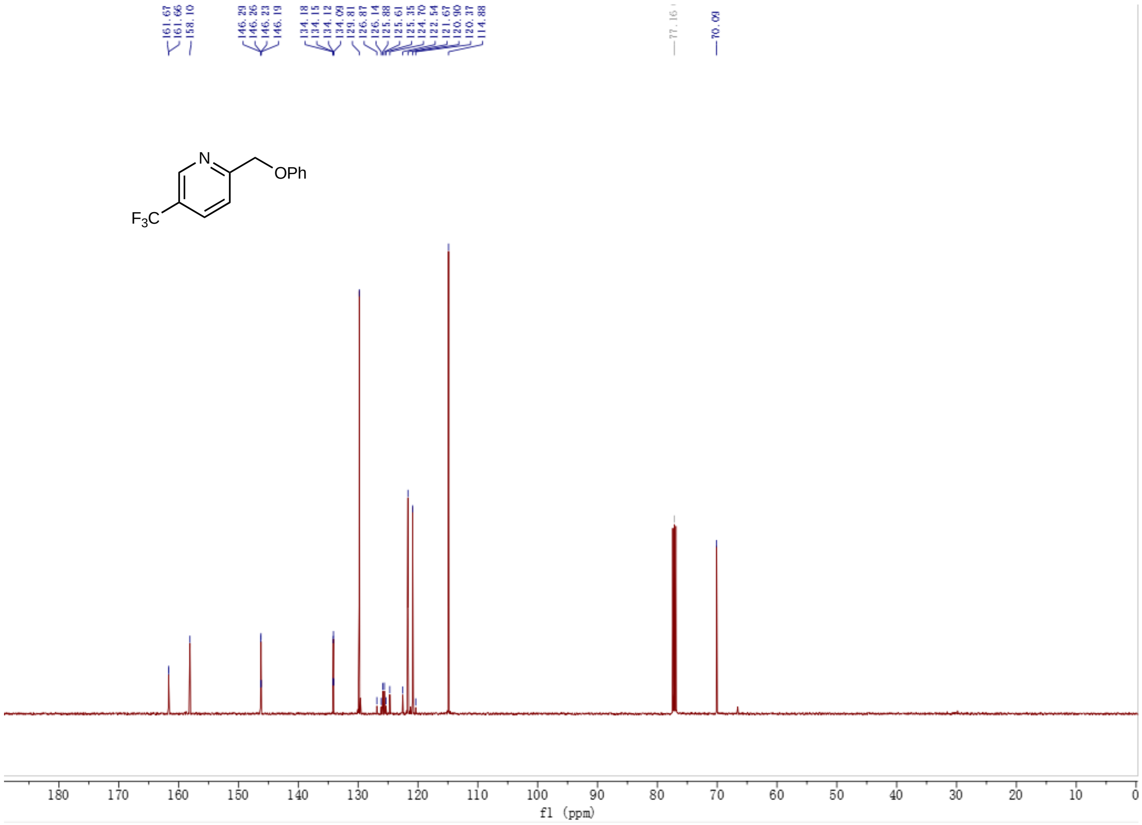

Supplementary Figure 149. ^{13}C NMR spectra of compound **5j**

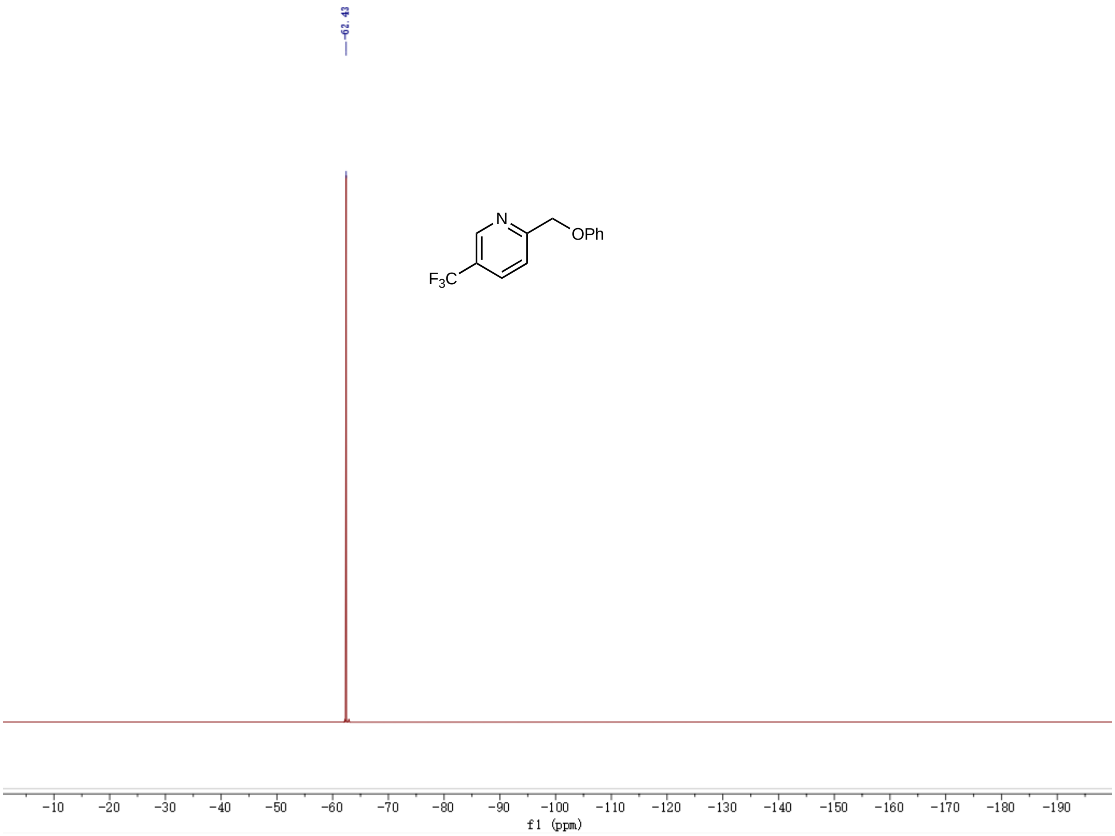

Supplementary Figure 150. ^{19}F NMR spectra of compound **5j**

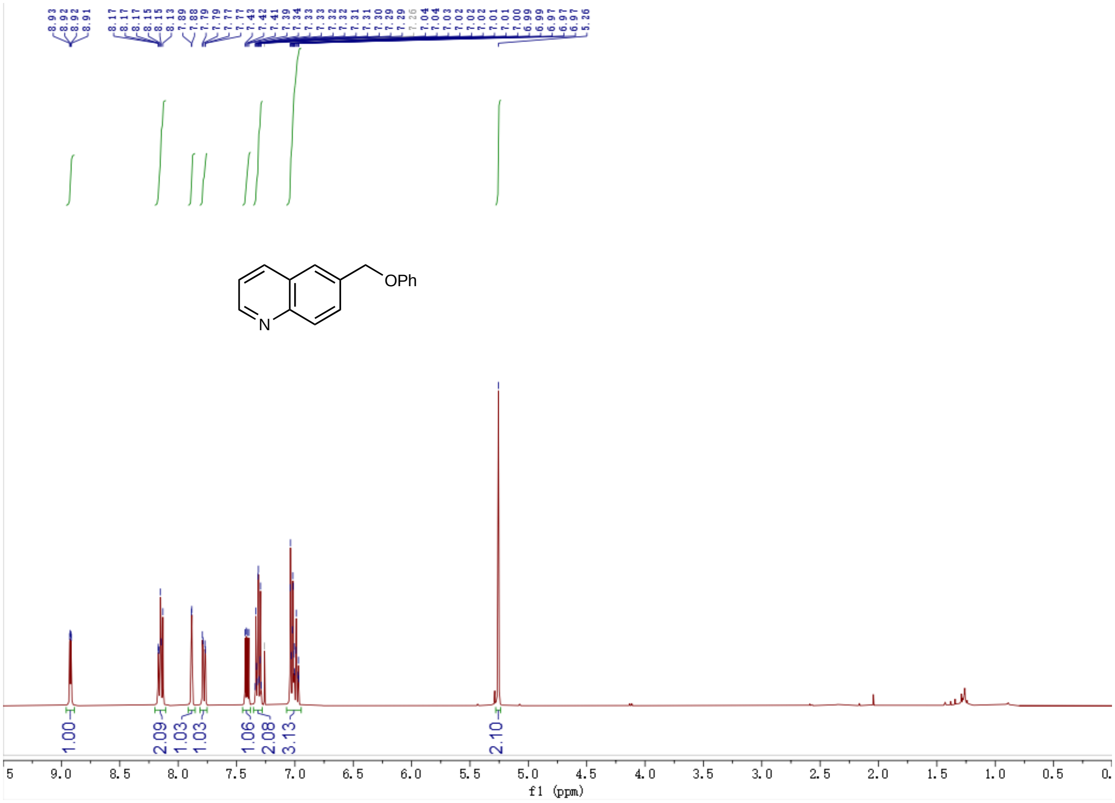

Supplementary Figure 151. ^{1}H NMR spectra of compound **5k**

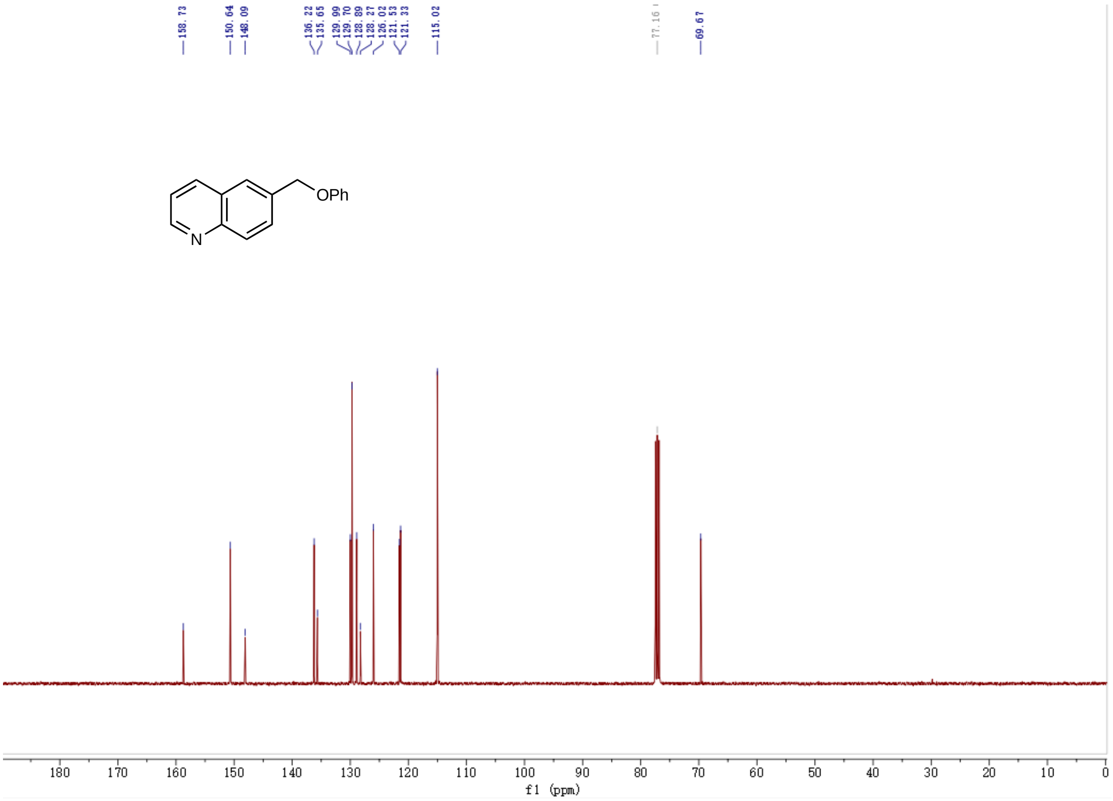

Supplementary Figure 152. ^{13}C NMR spectra of compound **5k**

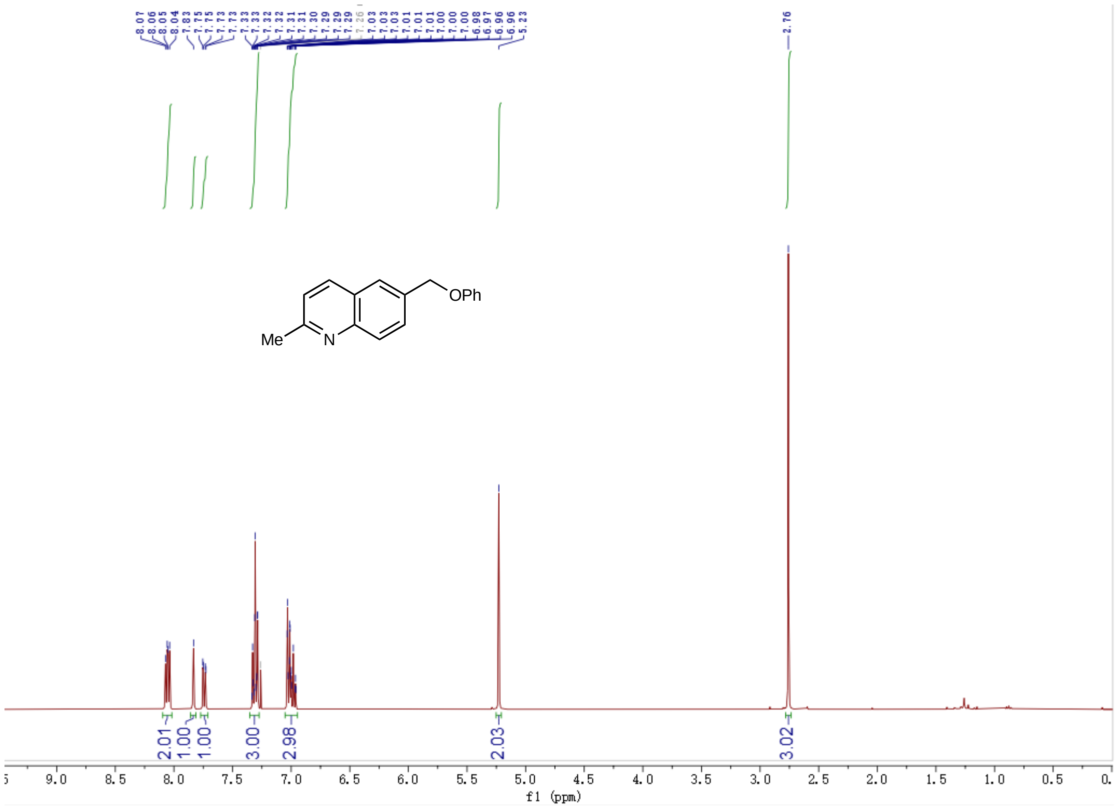

Supplementary Figure 153. ^{1}H NMR spectra of compound **5l**

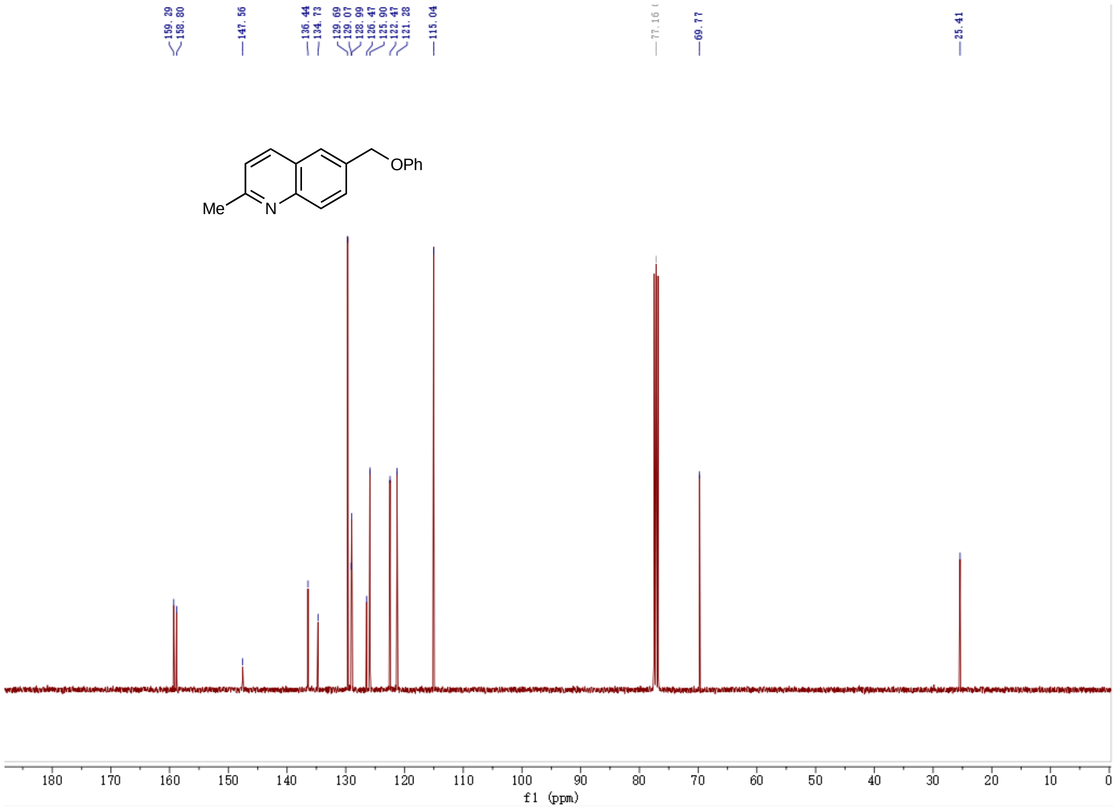

Supplementary Figure 154. ^{13}C NMR spectra of compound **5l**

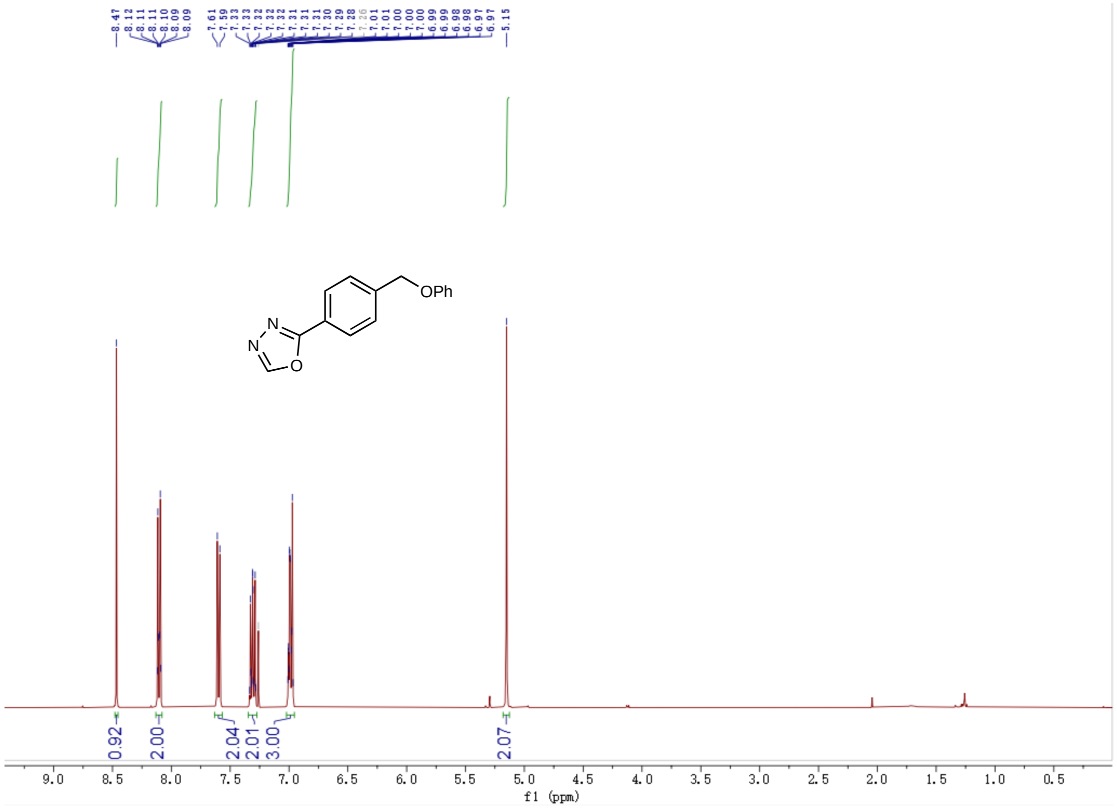

Supplementary Figure 155. ^{1}H NMR spectra of compound **5m**

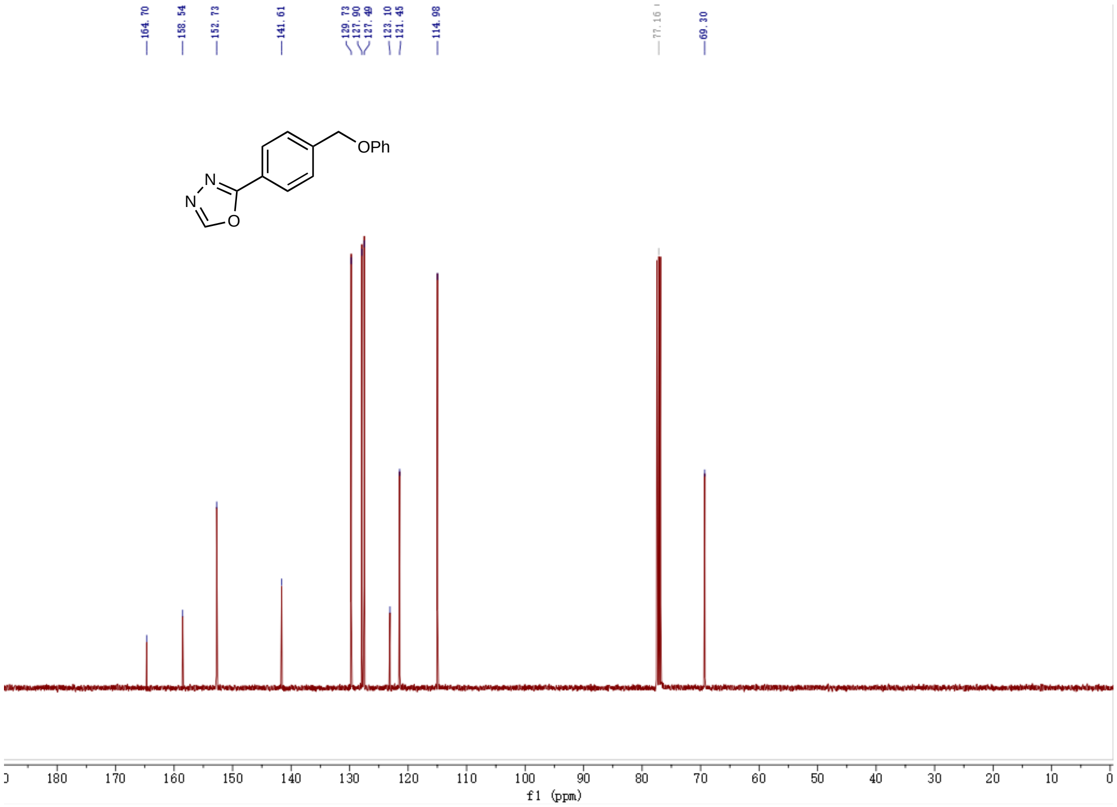

Supplementary Figure 156. ^{13}C NMR spectra of compound **5m**

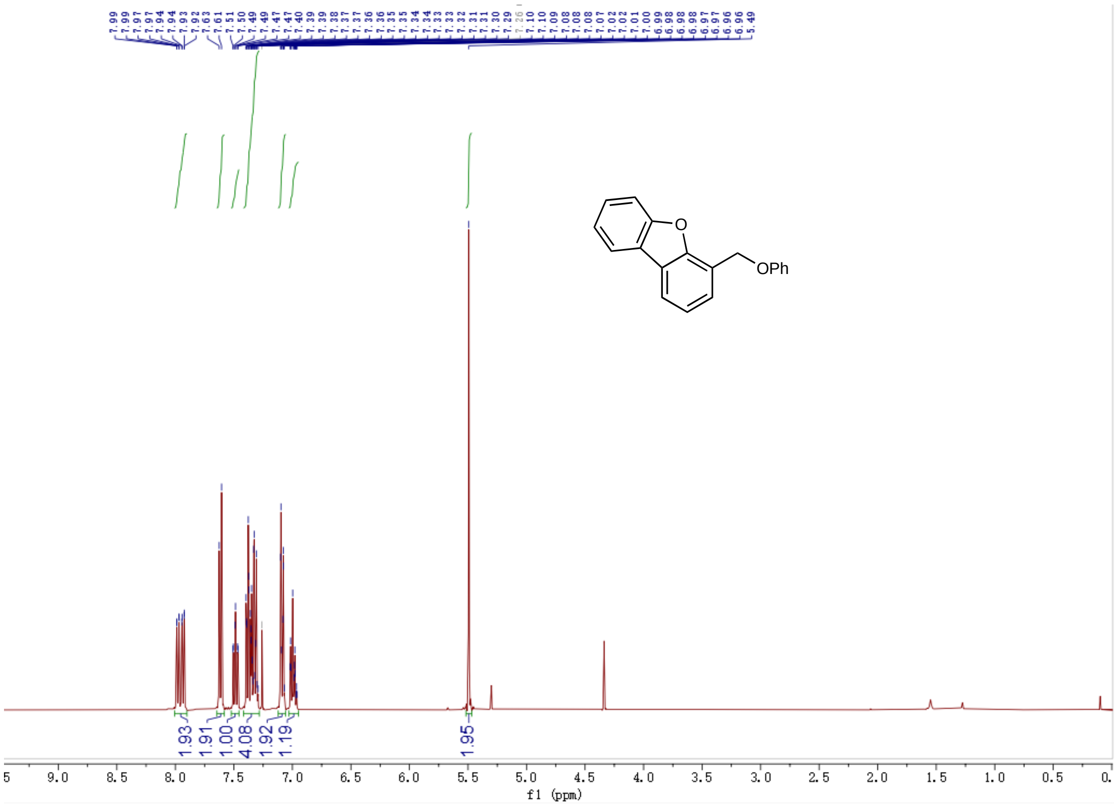

Supplementary Figure 157. ^{1}H NMR spectra of compound **5n**

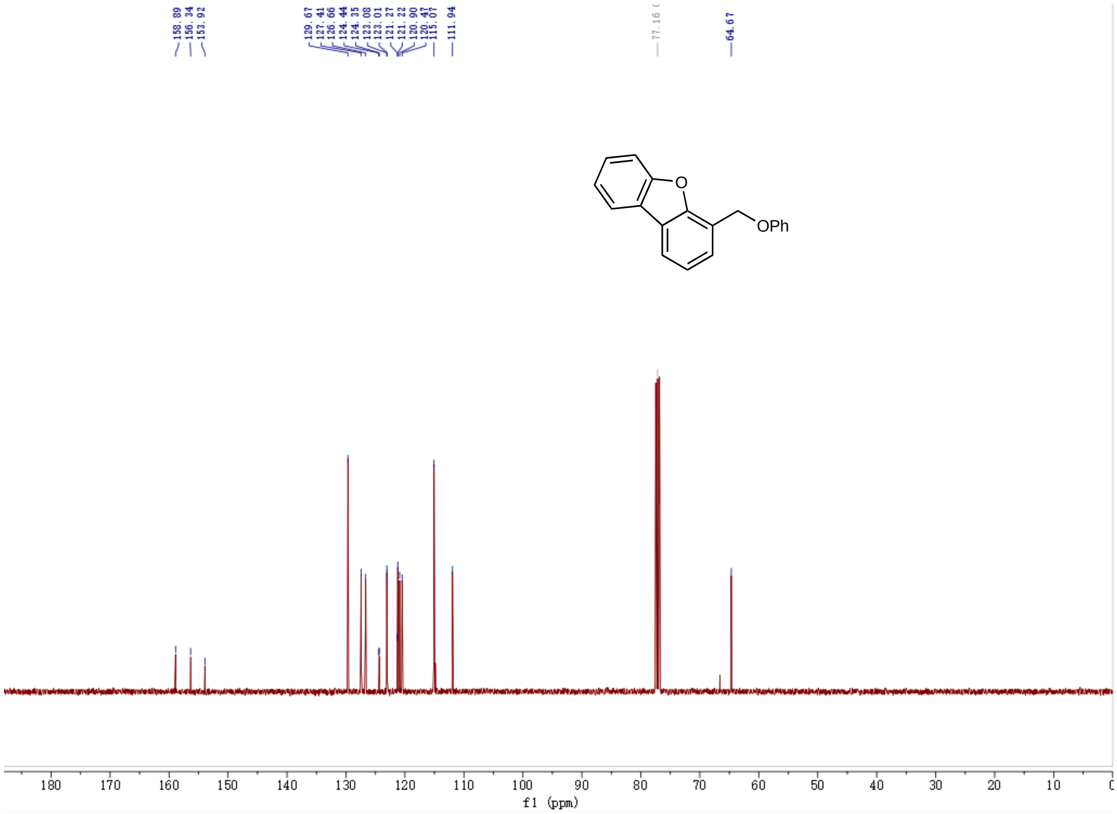

Supplementary Figure 158. ^{13}C NMR spectra of compound **5n**

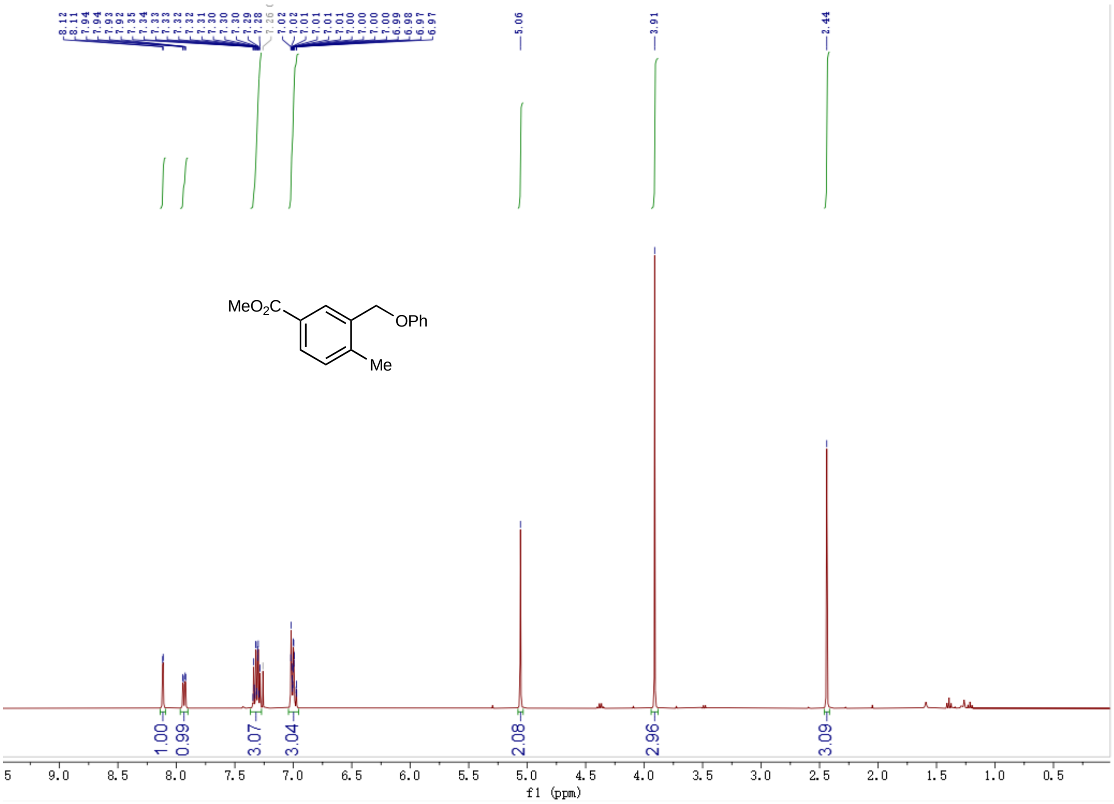

Supplementary Figure 159. ^{1}H NMR spectra of compound **5o**

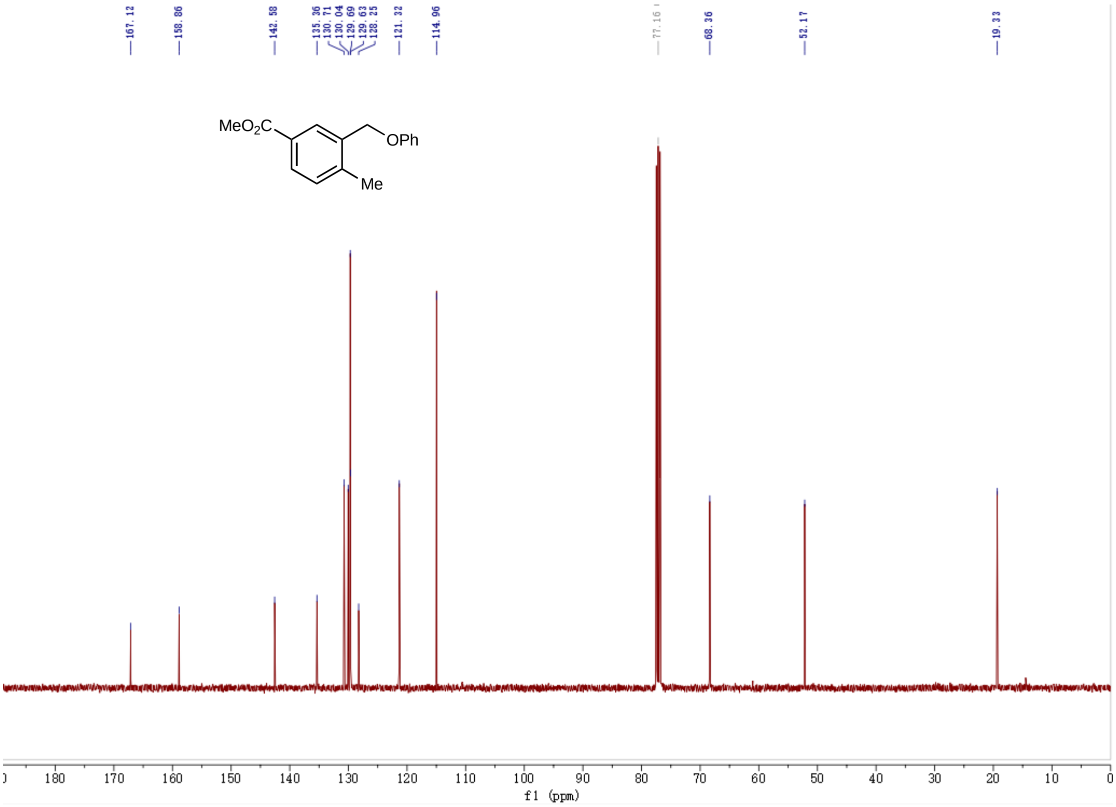

Supplementary Figure 160. ^{13}C NMR spectra of compound **5o**

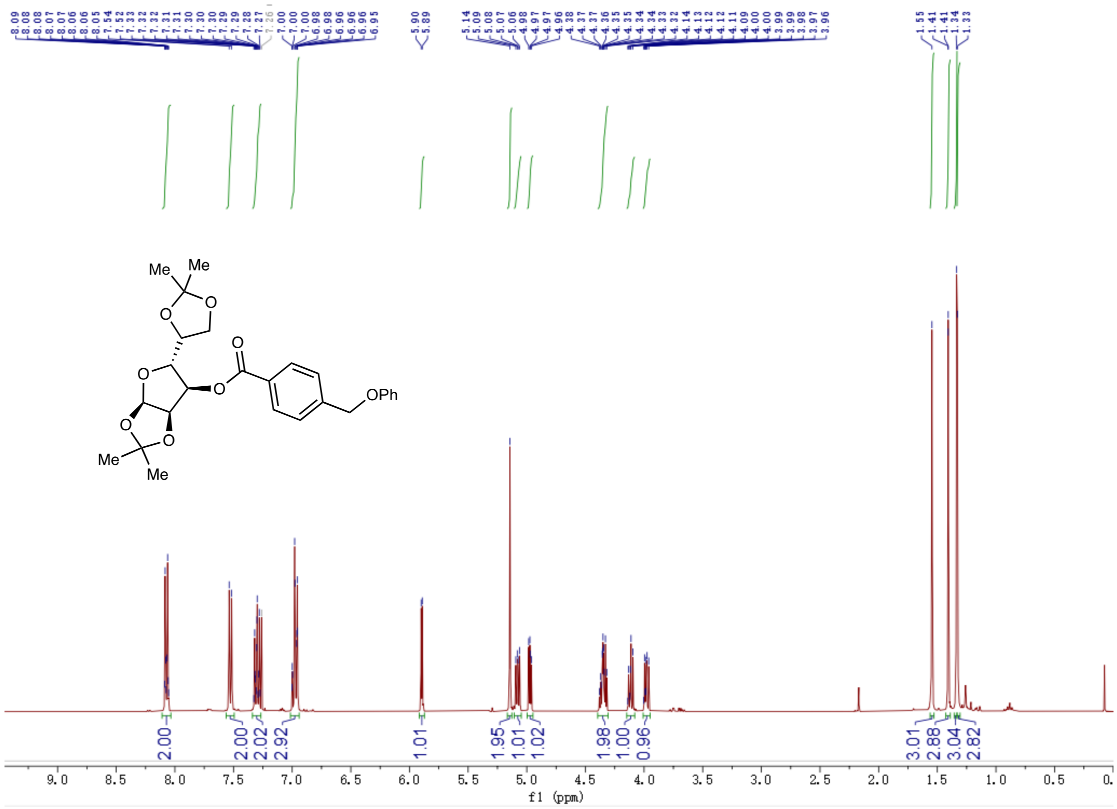

Supplementary Figure 161. ^{1}H NMR spectra of compound **5p**

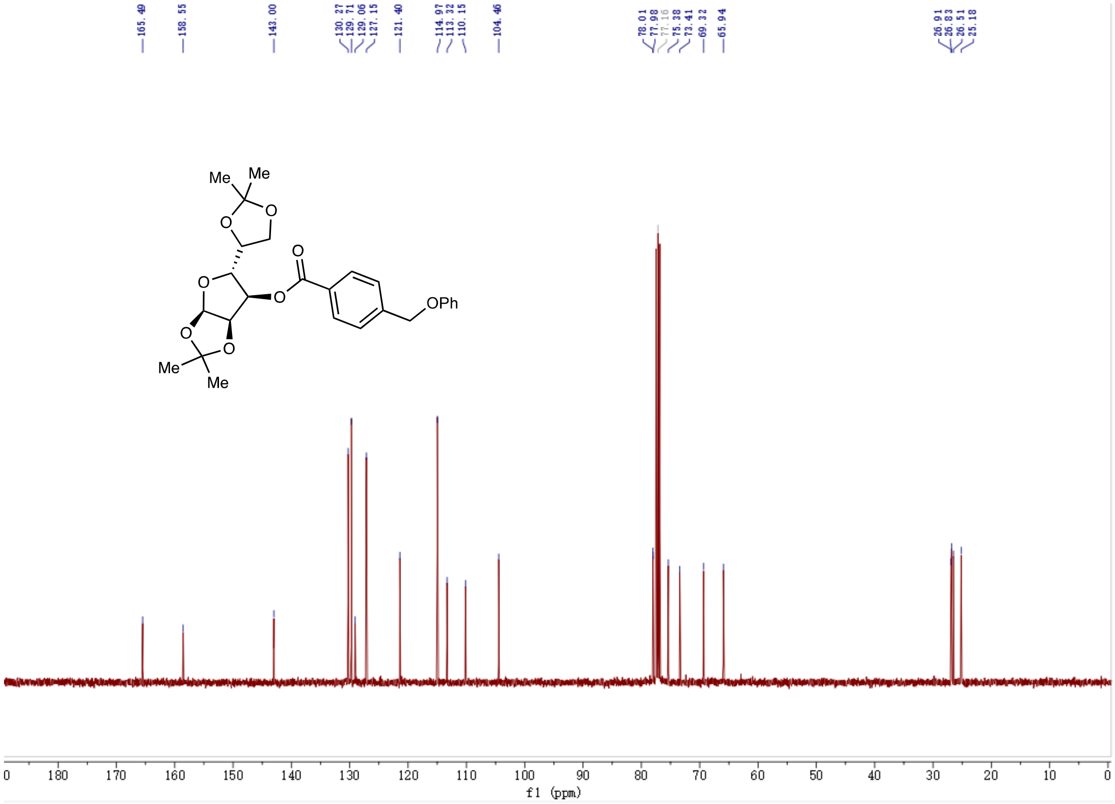

Supplementary Figure 162. ^{13}C NMR spectra of compound **5p**

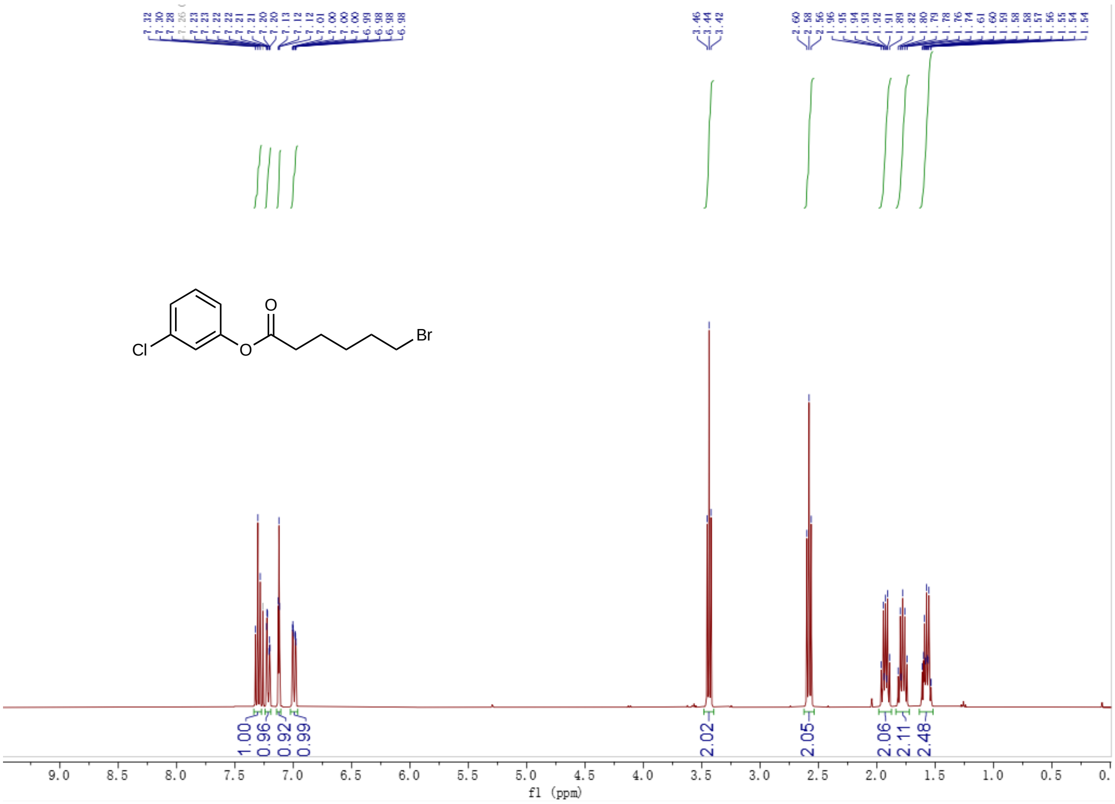

Supplementary Figure 163. ^{1}H NMR spectra of compound **7e**

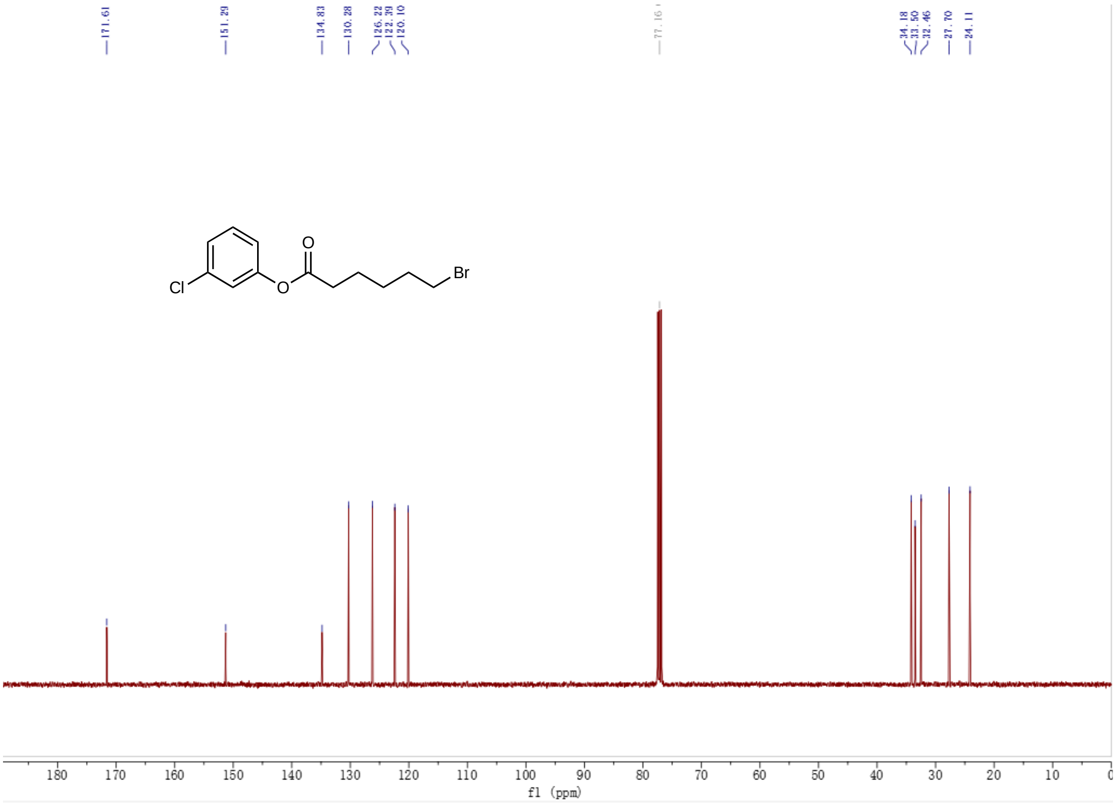

Supplementary Figure 164. ^{13}C NMR spectra of compound **7e**

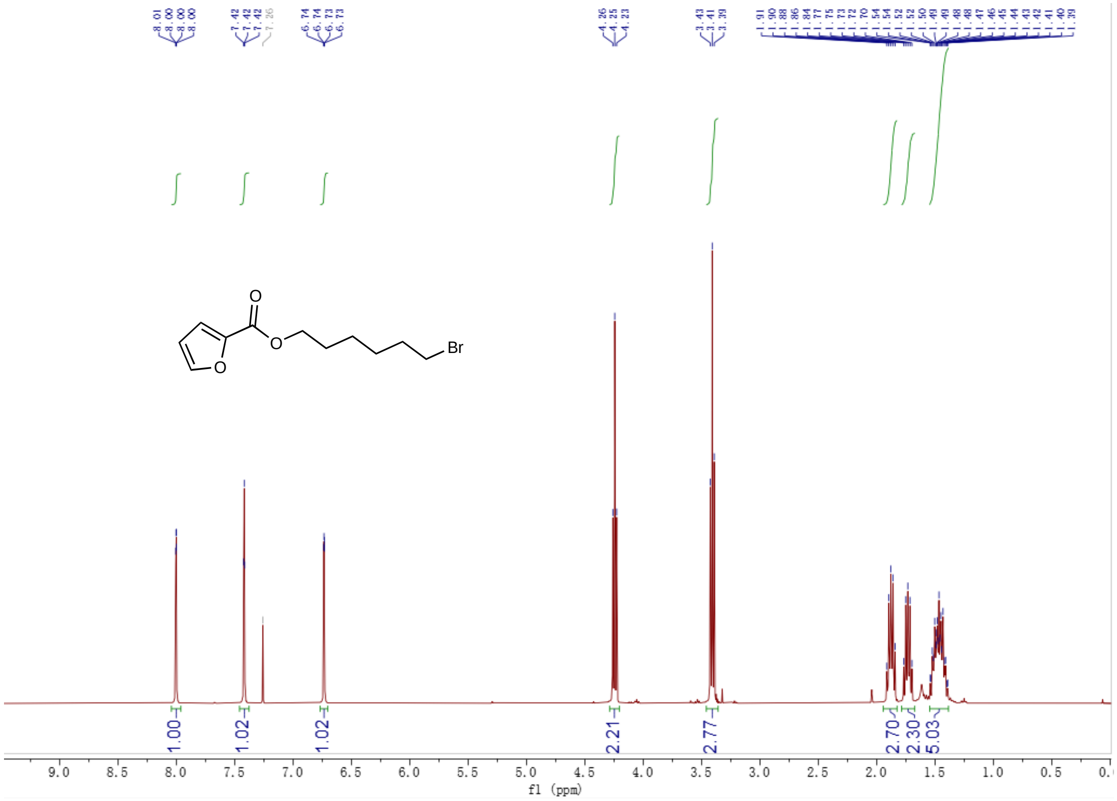

Supplementary Figure 165. ^{1}H NMR spectra of compound **7g**

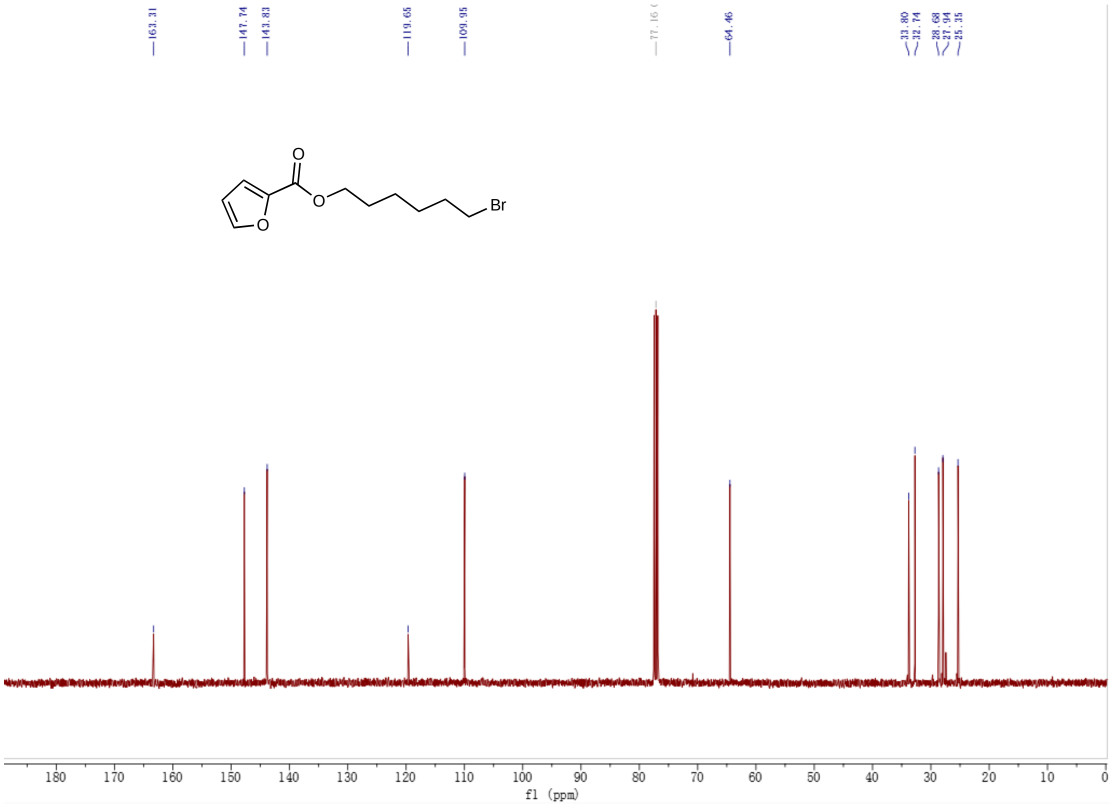

Supplementary Figure 166. ^{13}C NMR spectra of compound **7g**

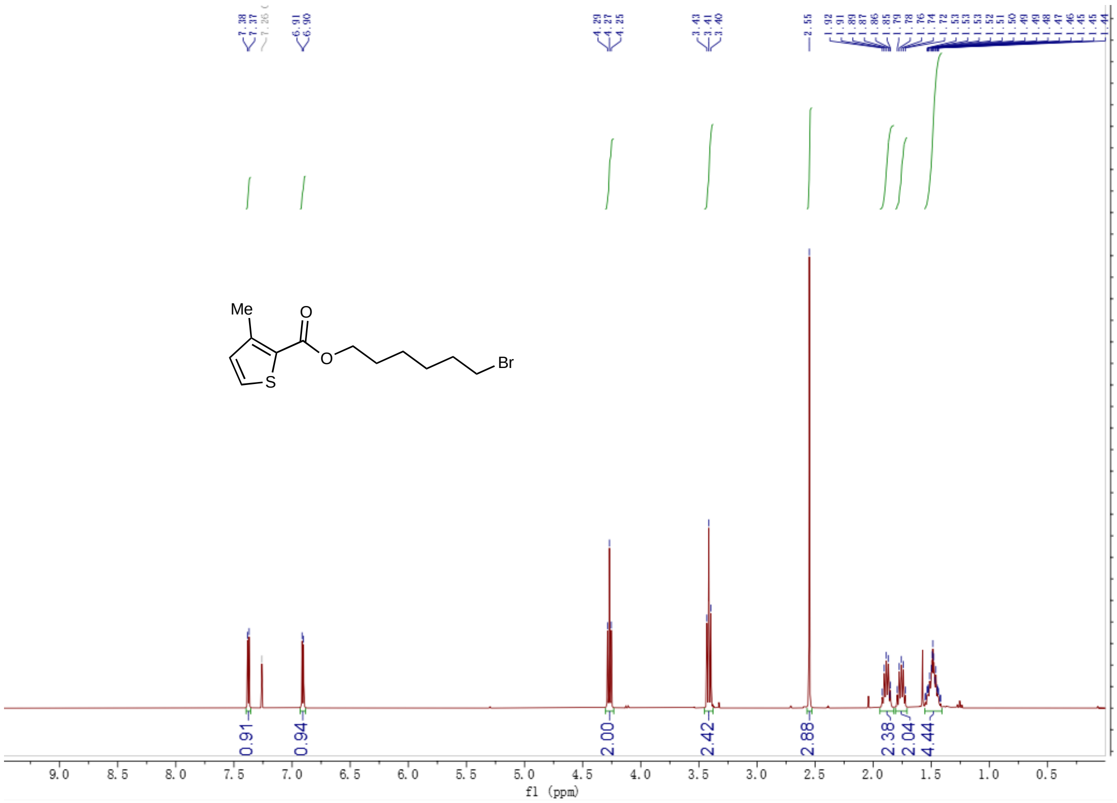

Supplementary Figure 167. ^{1}H NMR spectra of compound **7i**

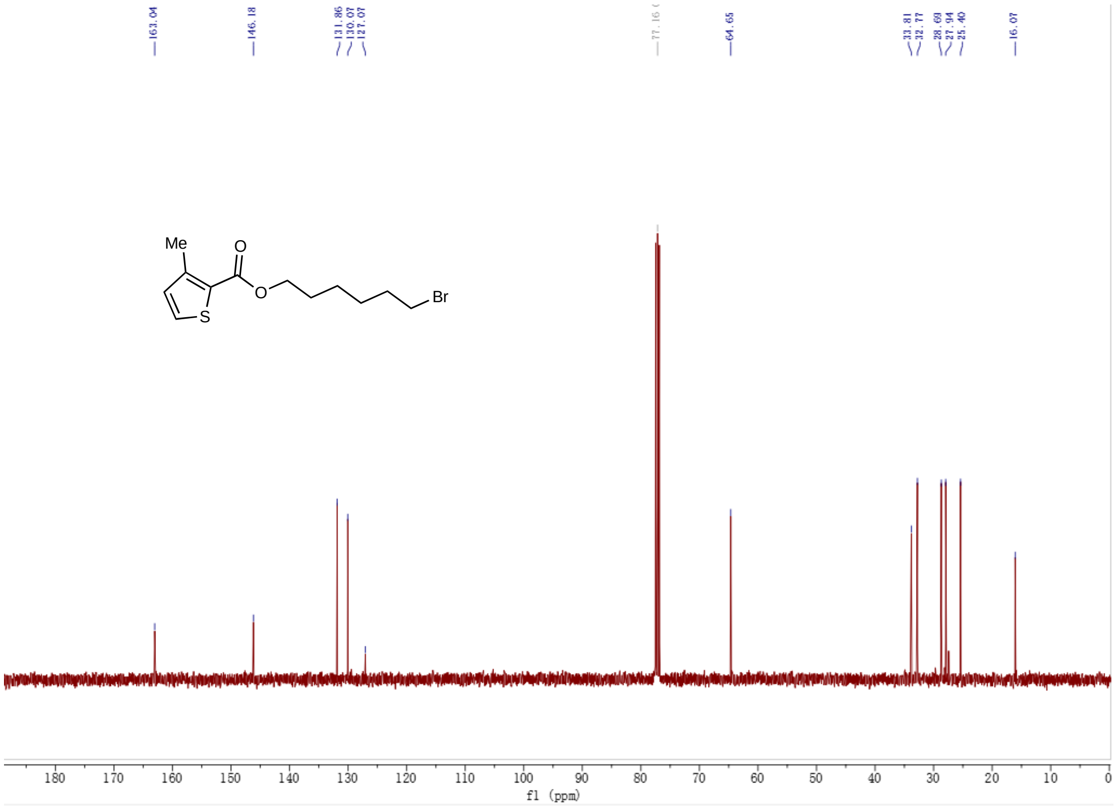

Supplementary Figure 168. ^{13}C NMR spectra of compound **7i**

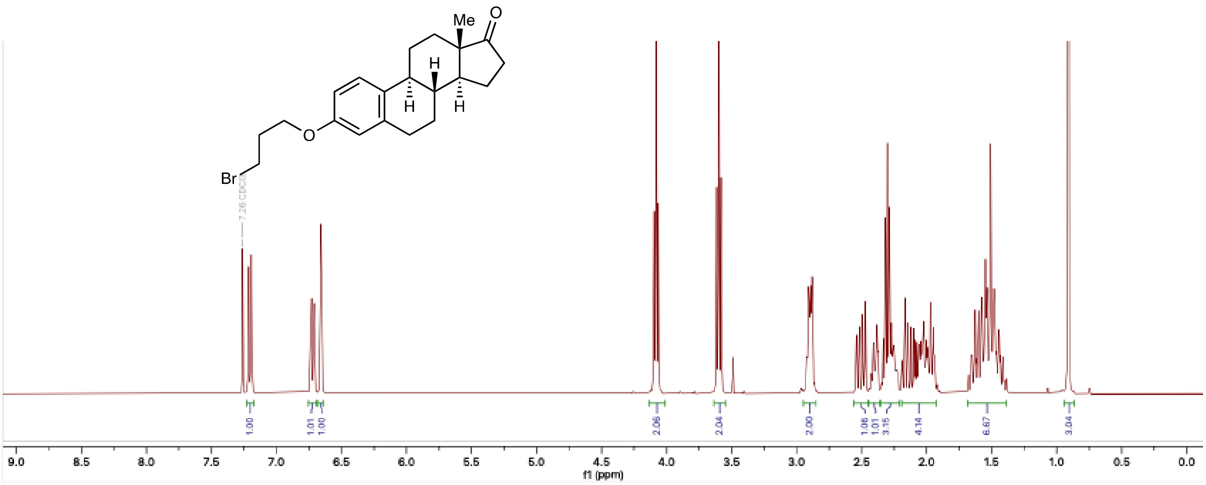

Supplementary Figure 169. ^{1}H NMR spectra of compound **7j**

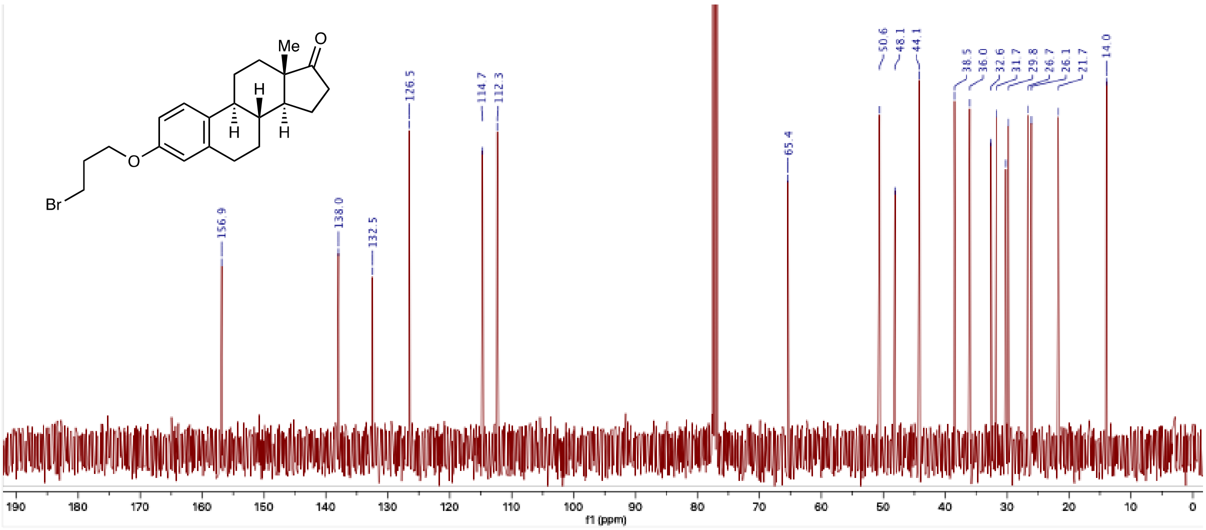

Supplementary Figure 170. ^{13}C NMR spectra of compound **7j**

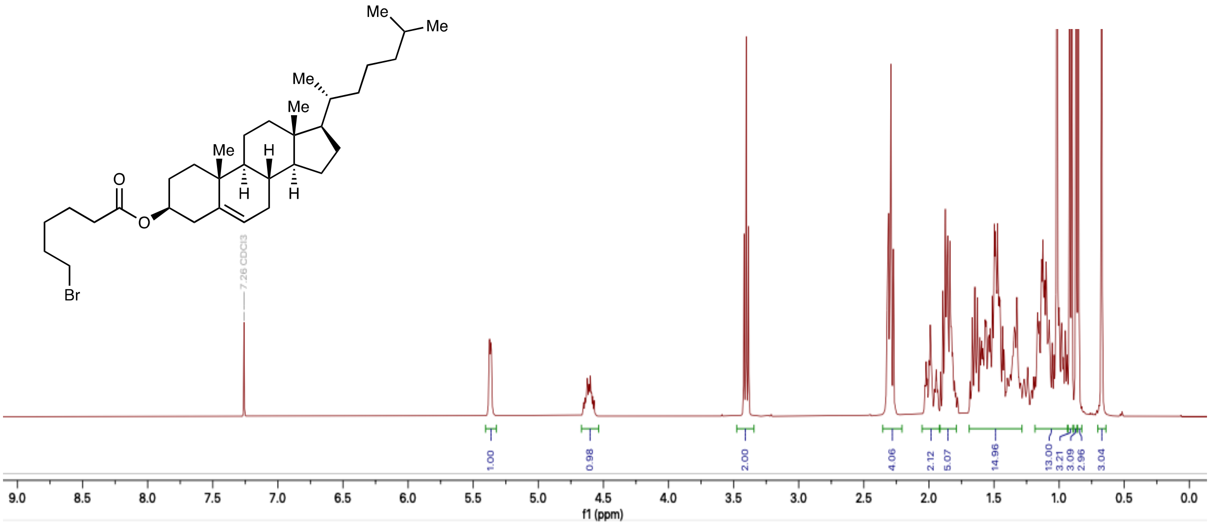

Supplementary Figure 171. ^{1}H NMR spectra of compound **7k**

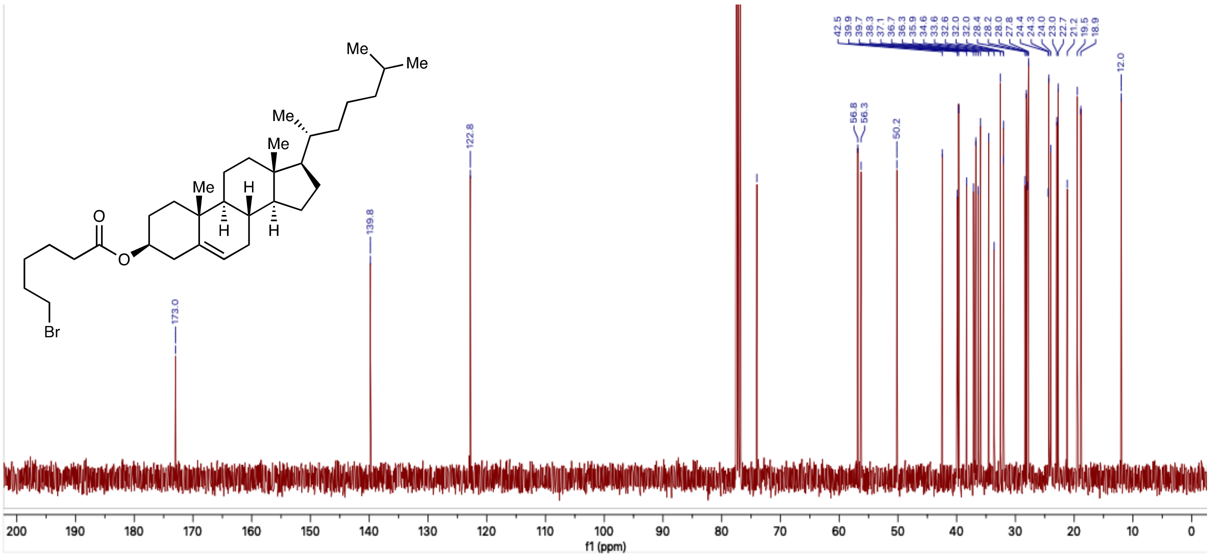

Supplementary Figure 172. ^{13}C NMR spectra of compound **7k**

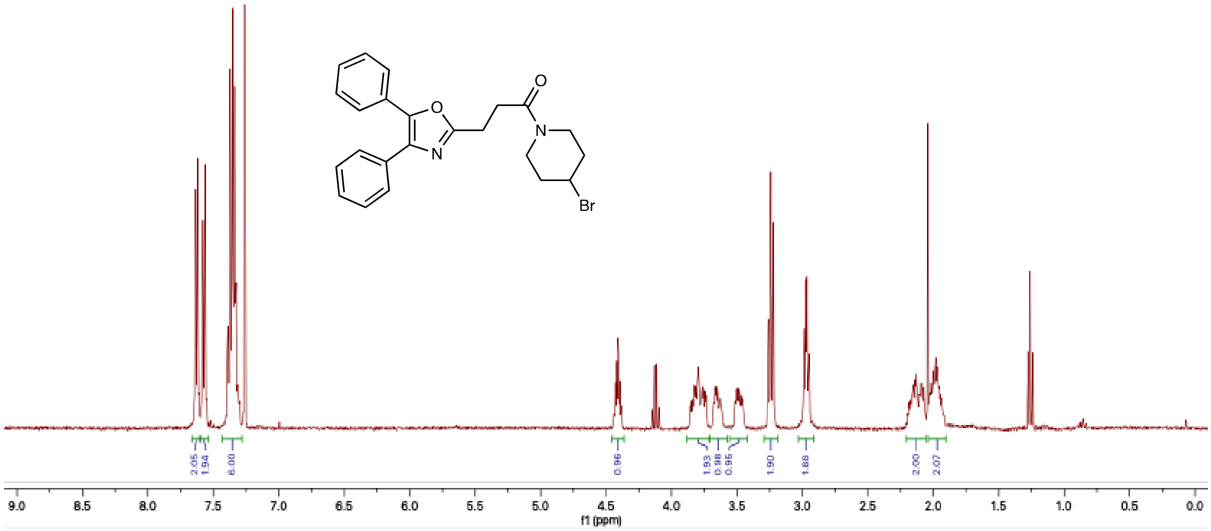

Supplementary Figure 173. ^{1}H NMR spectra of compound **7p**

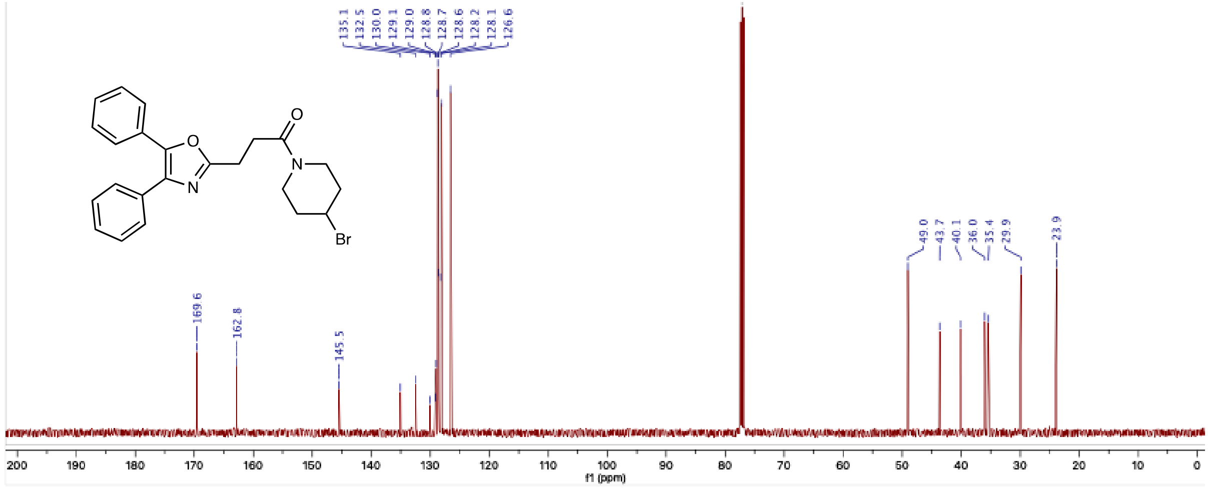

Supplementary Figure 174. ^{13}C NMR spectra of compound **7p**

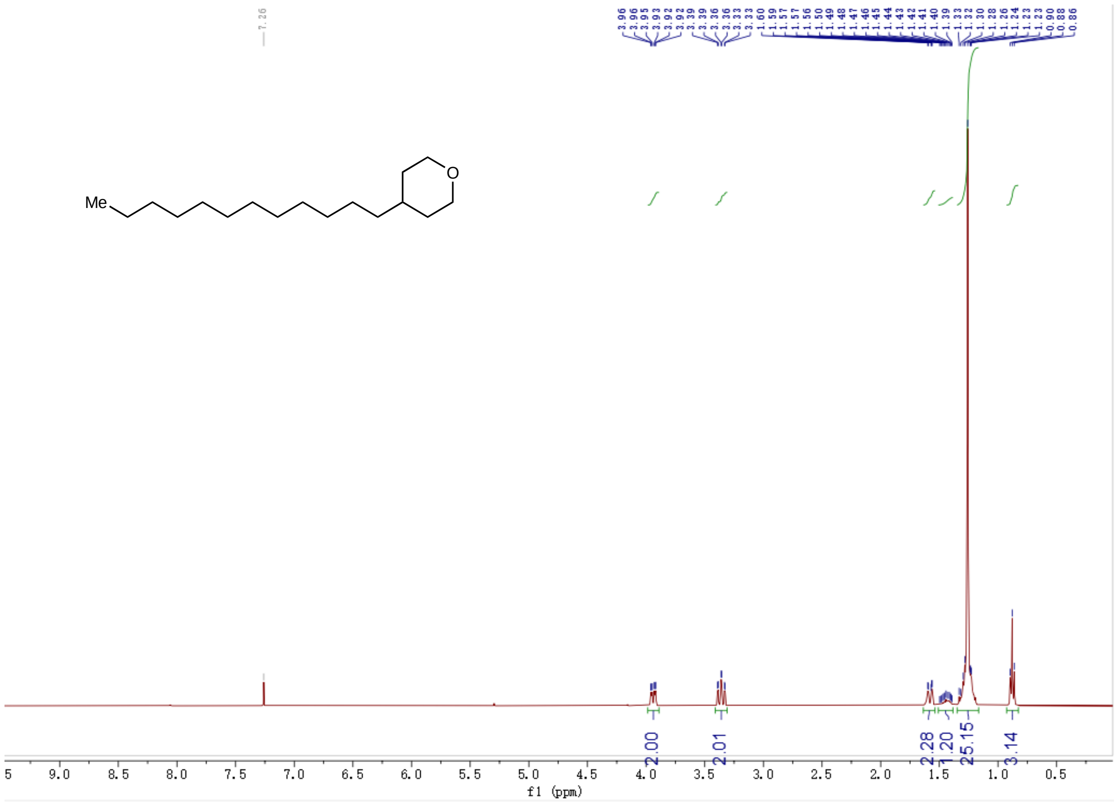

Supplementary Figure 175. ^{1}H NMR spectra of compound **8a**

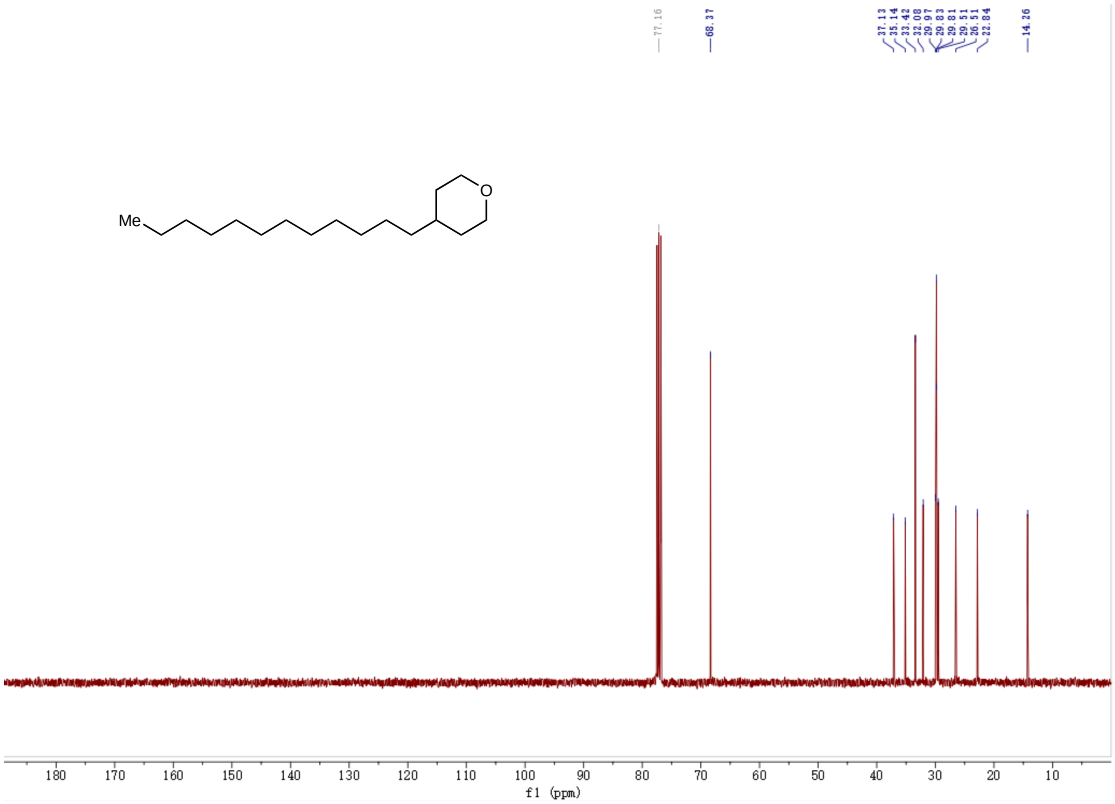

Supplementary Figure 176. ^{13}C NMR spectra of compound **8a**

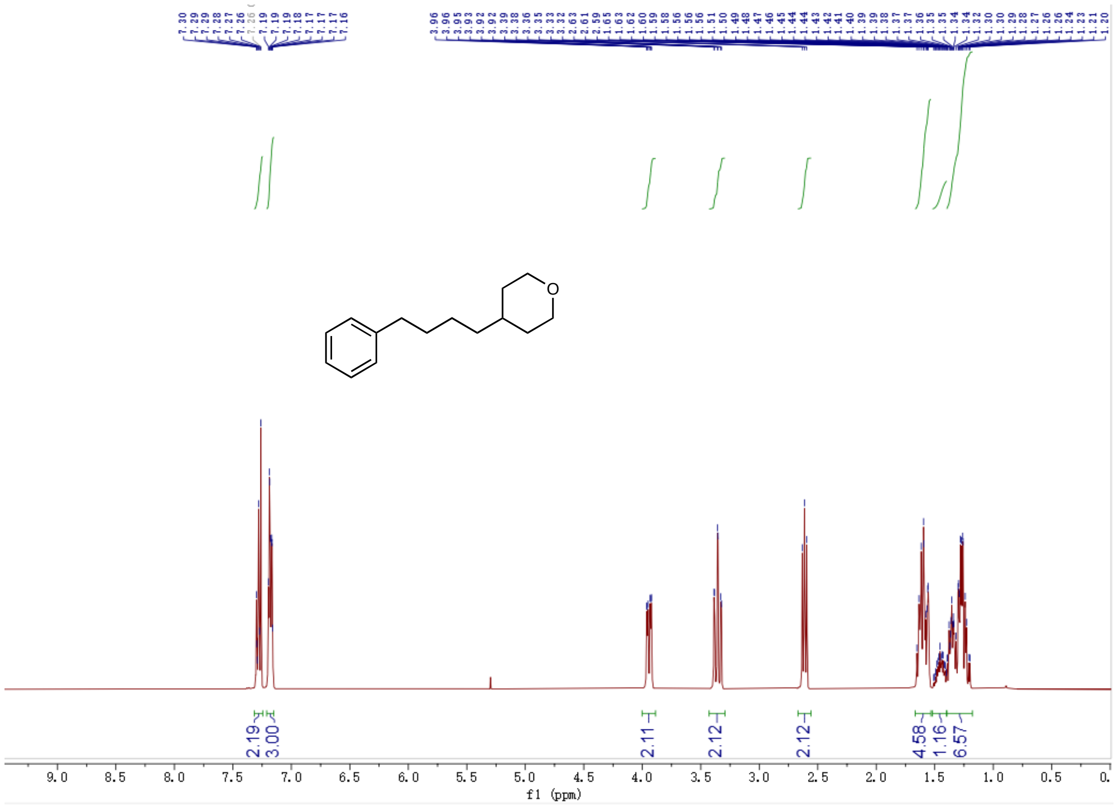

Supplementary Figure 177. ^{1}H NMR spectra of compound **8b**

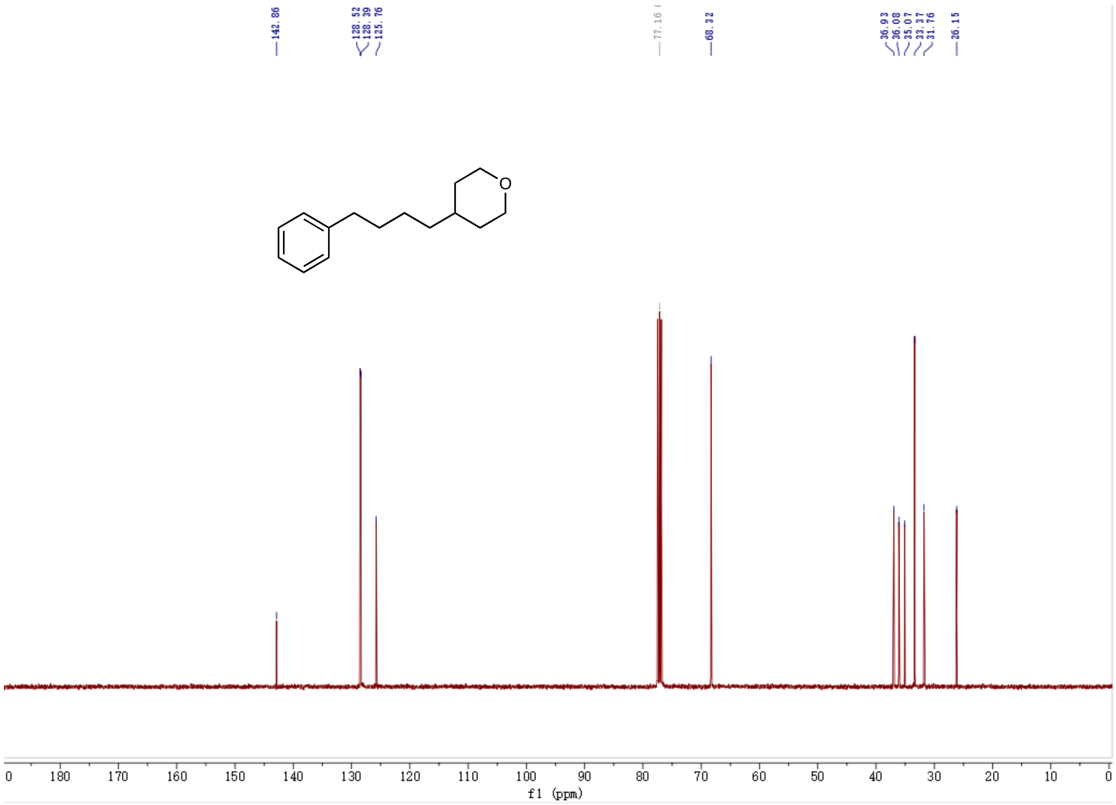

Supplementary Figure 178. ^{13}C NMR spectra of compound **8b**

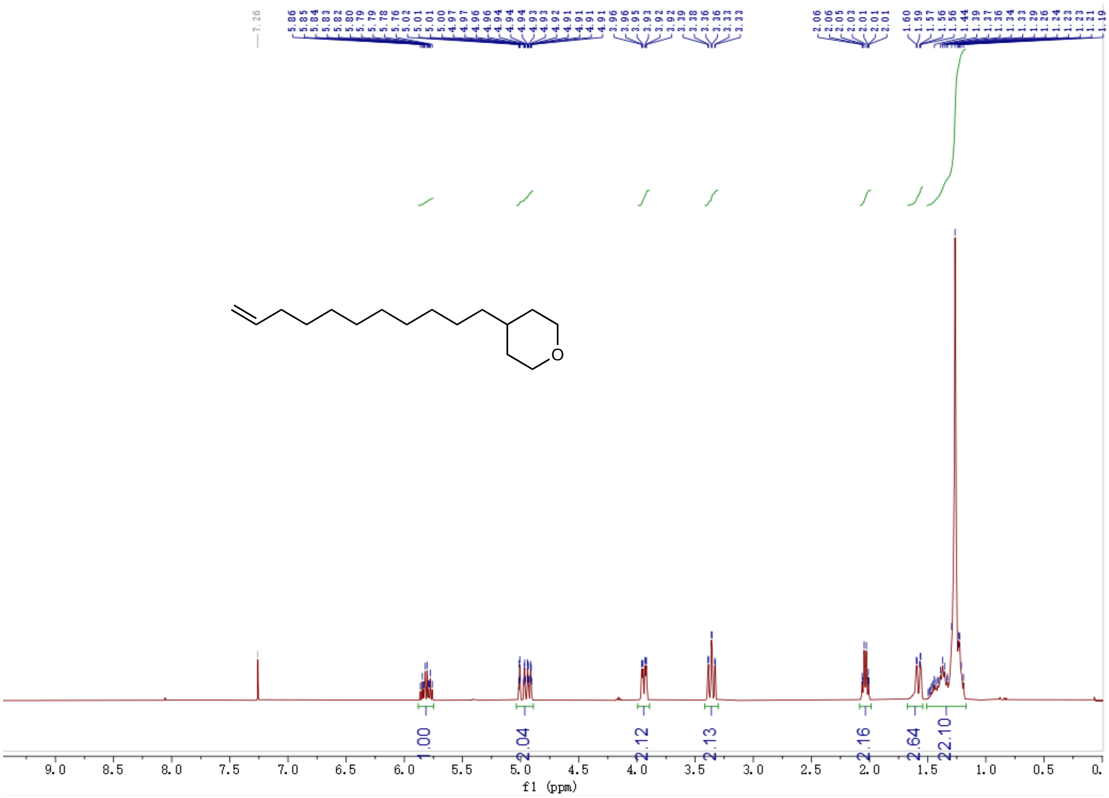

Supplementary Figure 179. ^{1}H NMR spectra of compound **8c**

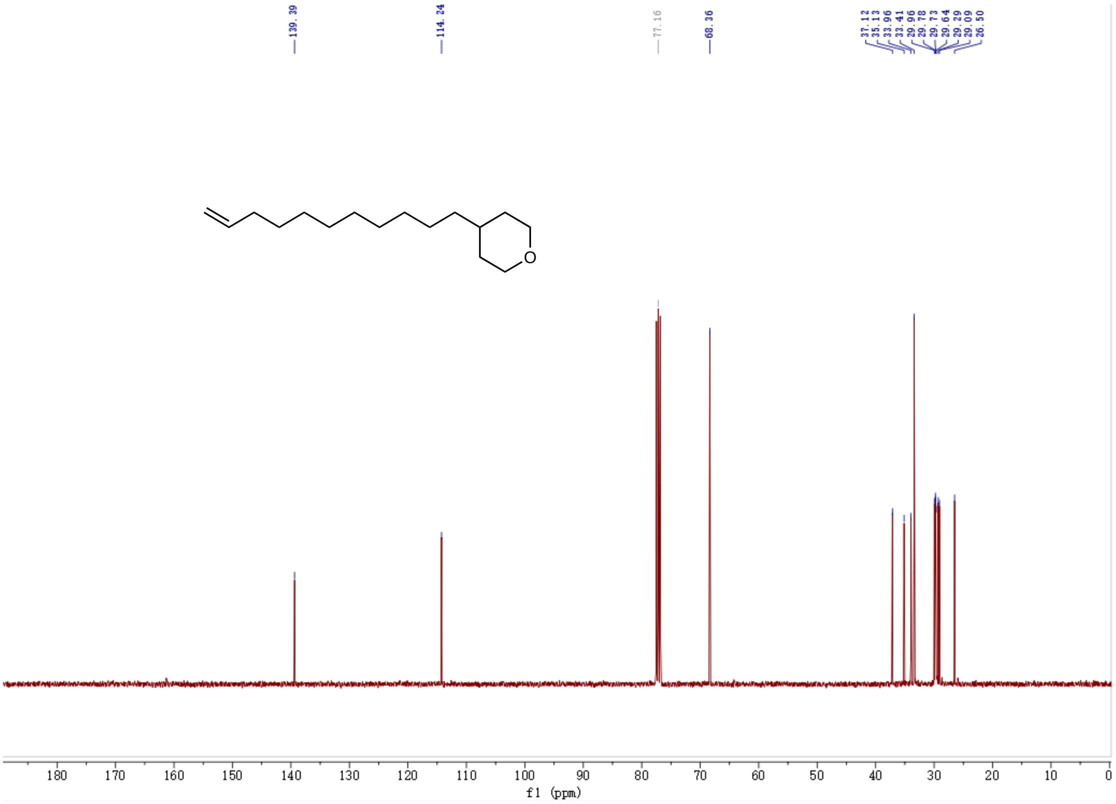

Supplementary Figure 180. ^{13}C NMR spectra of compound **8c**

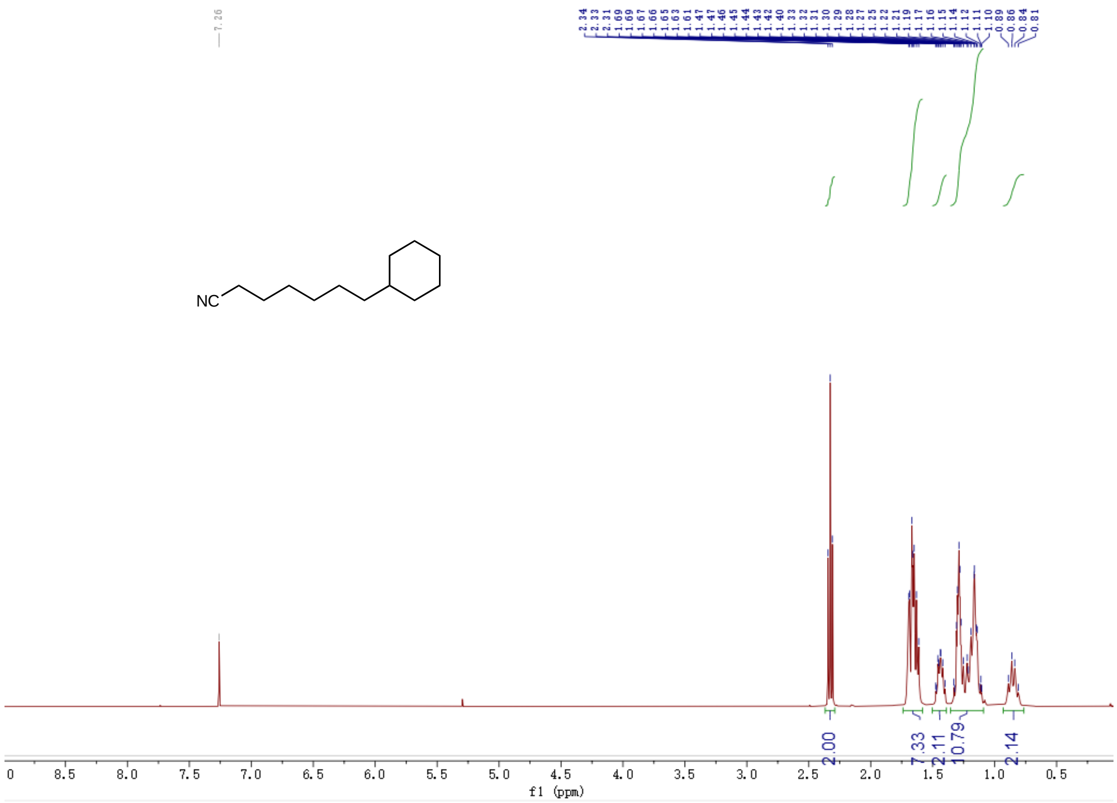

Supplementary Figure 181. ^{1}H NMR spectra of compound **8d**

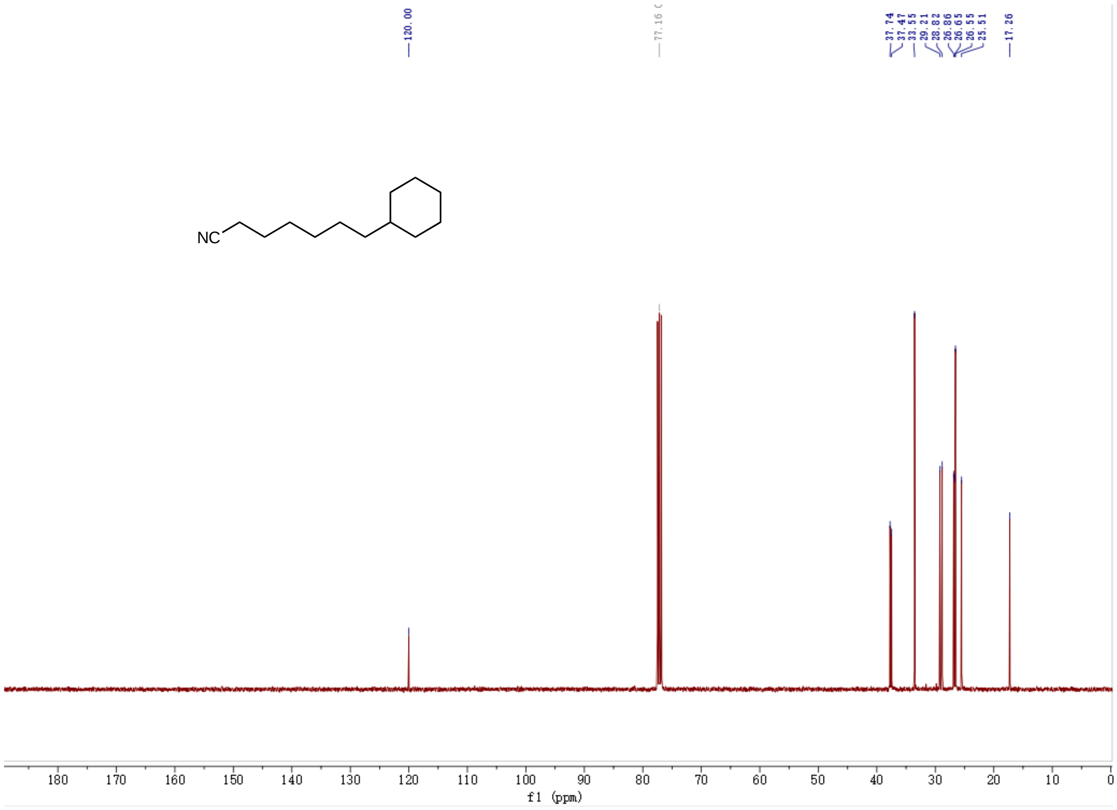

Supplementary Figure 182. ^{13}C NMR spectra of compound **8d**

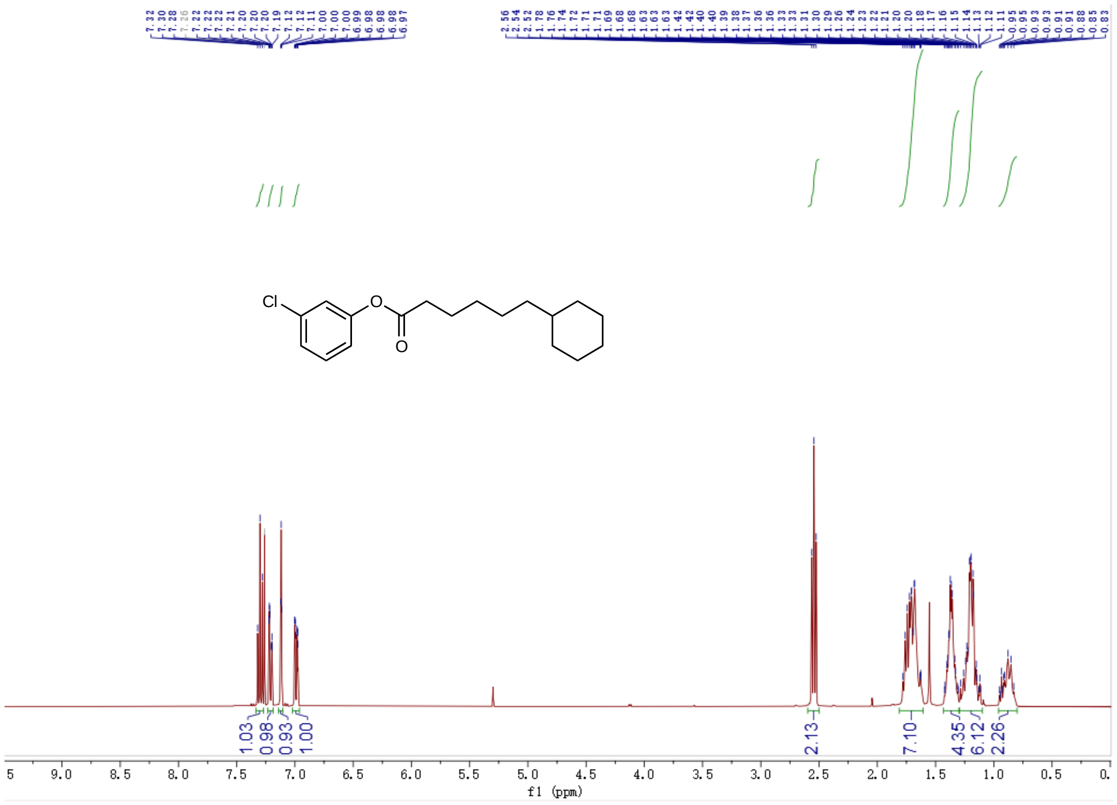

Supplementary Figure 183. ^{1}H NMR spectra of compound **8e**

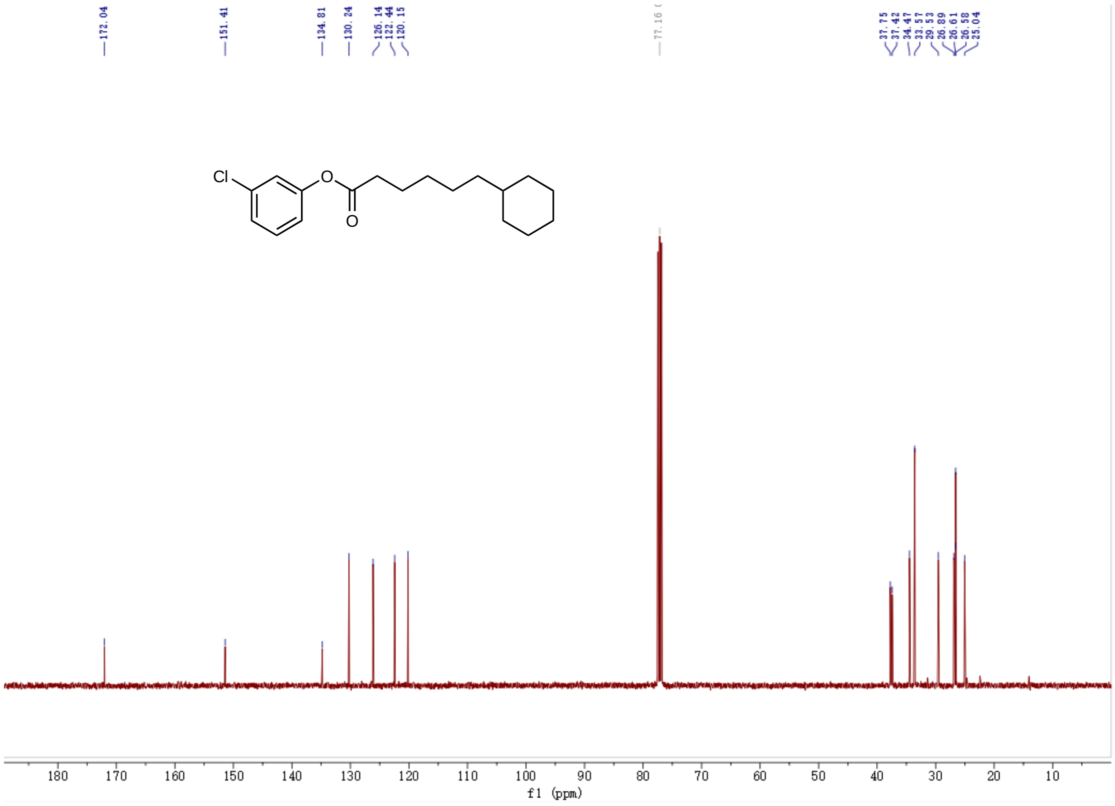

Supplementary Figure 184. ^{13}C NMR spectra of compound **8e**

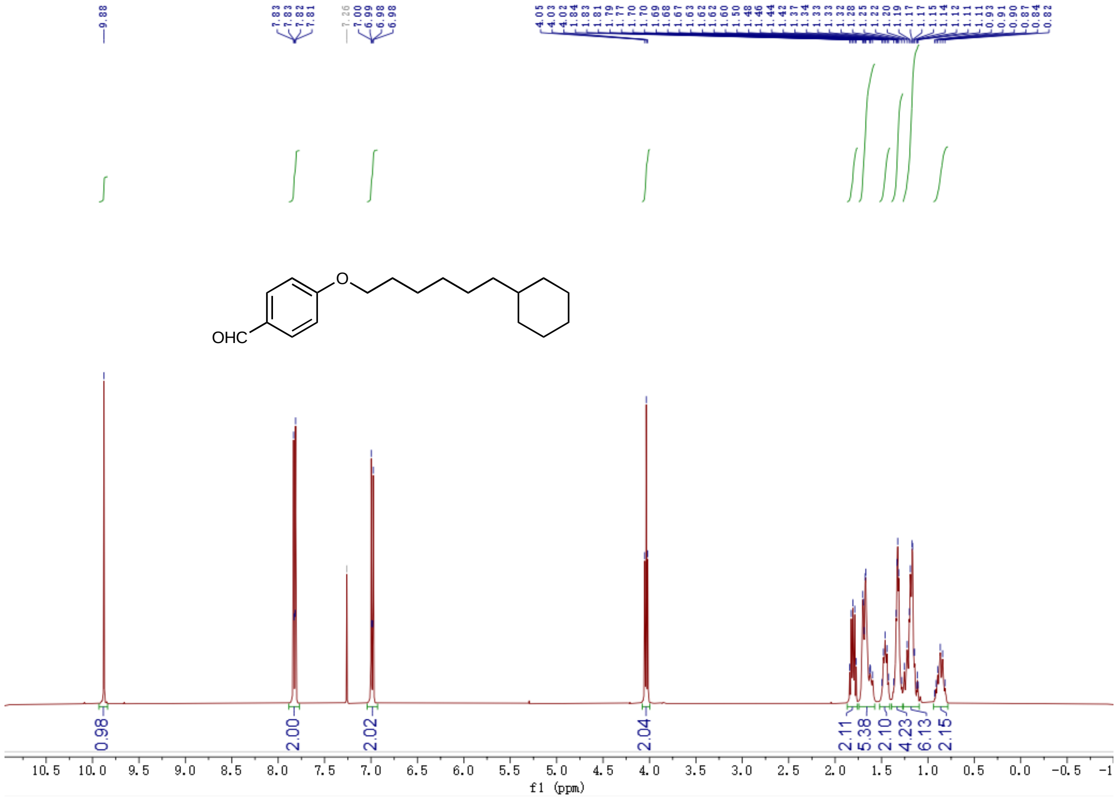

Supplementary Figure 185. ^{1}H NMR spectra of compound **8f**

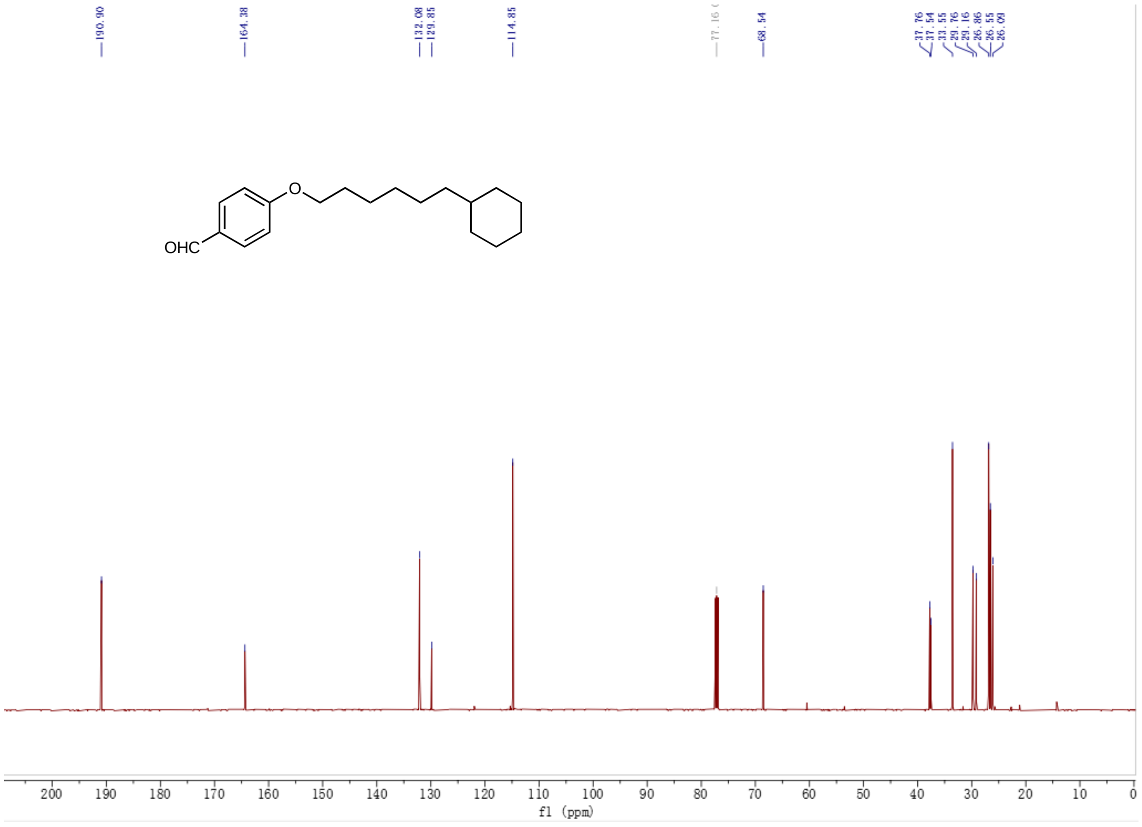

Supplementary Figure 186. ^{13}C NMR spectra of compound **8f**

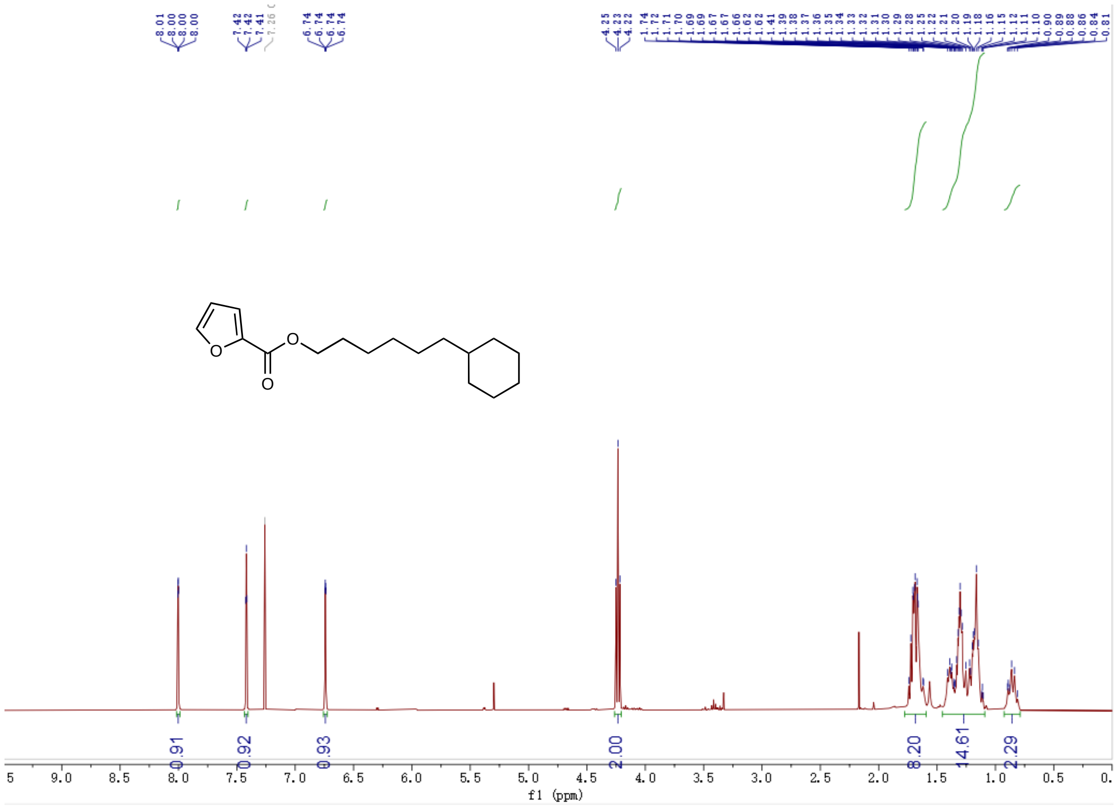

Supplementary Figure 187. ^{1}H NMR spectra of compound **8g**

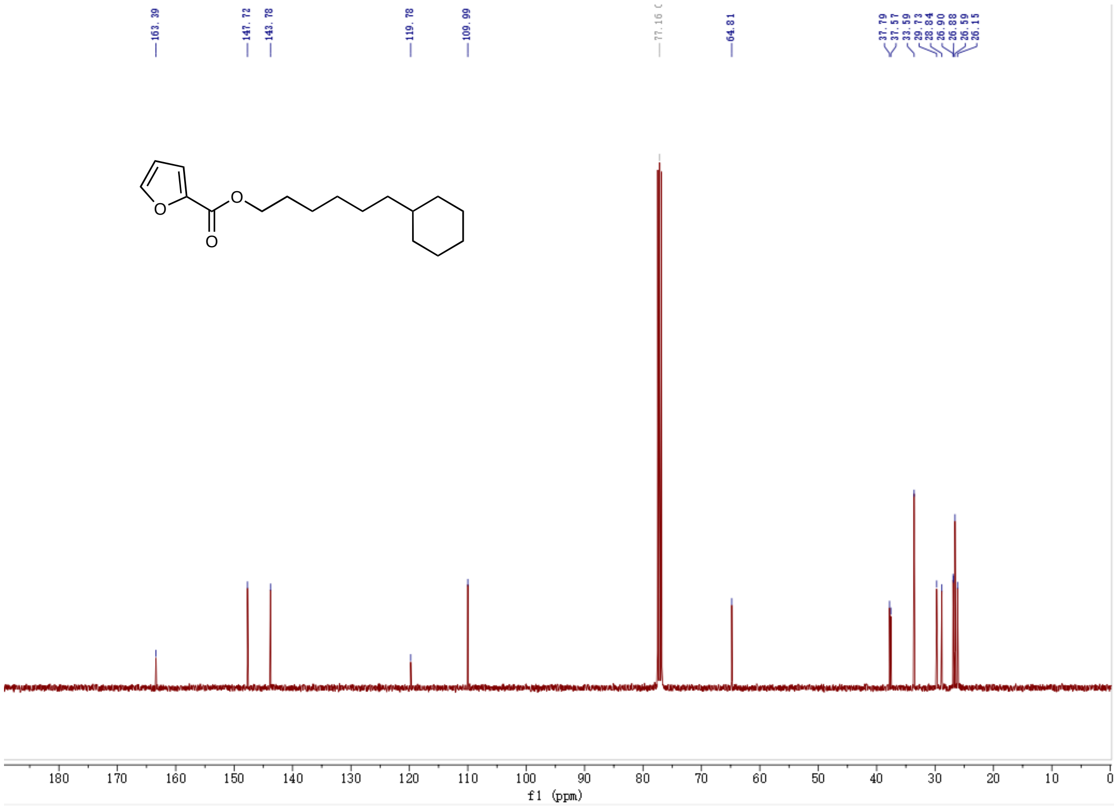

Supplementary Figure 188. ^{13}C NMR spectra of compound **8g**

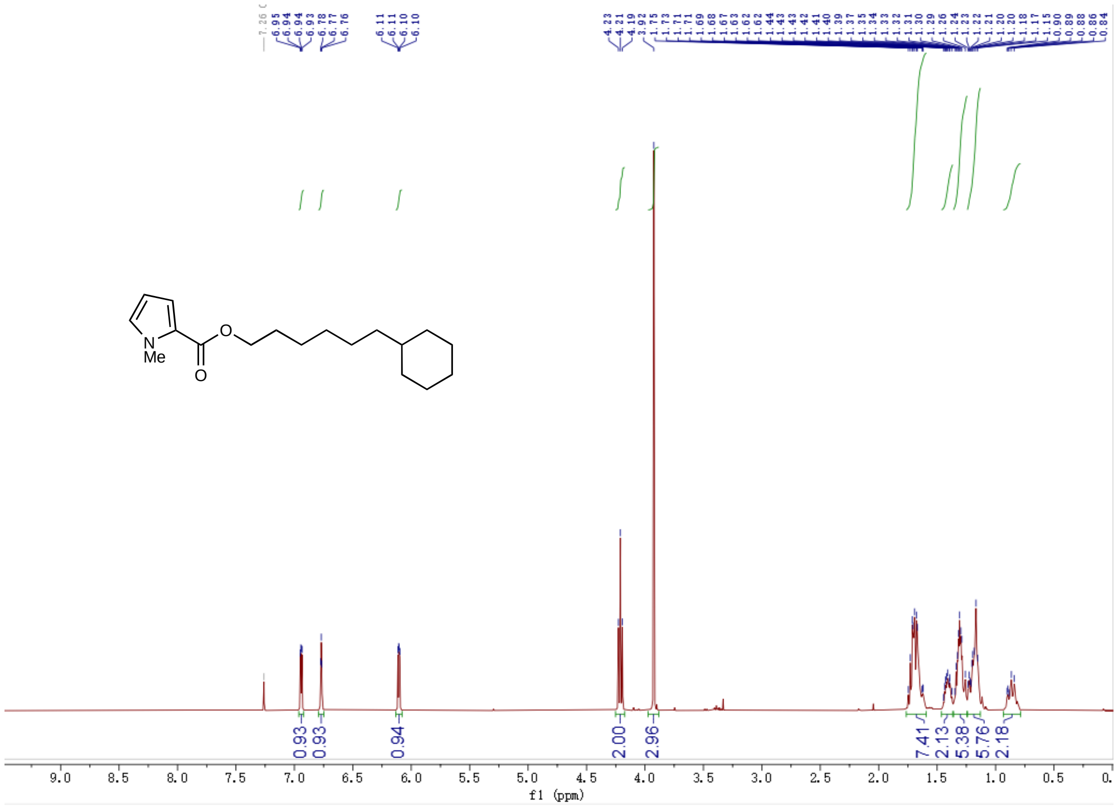

Supplementary Figure 189. ^{1}H NMR spectra of compound **8h**

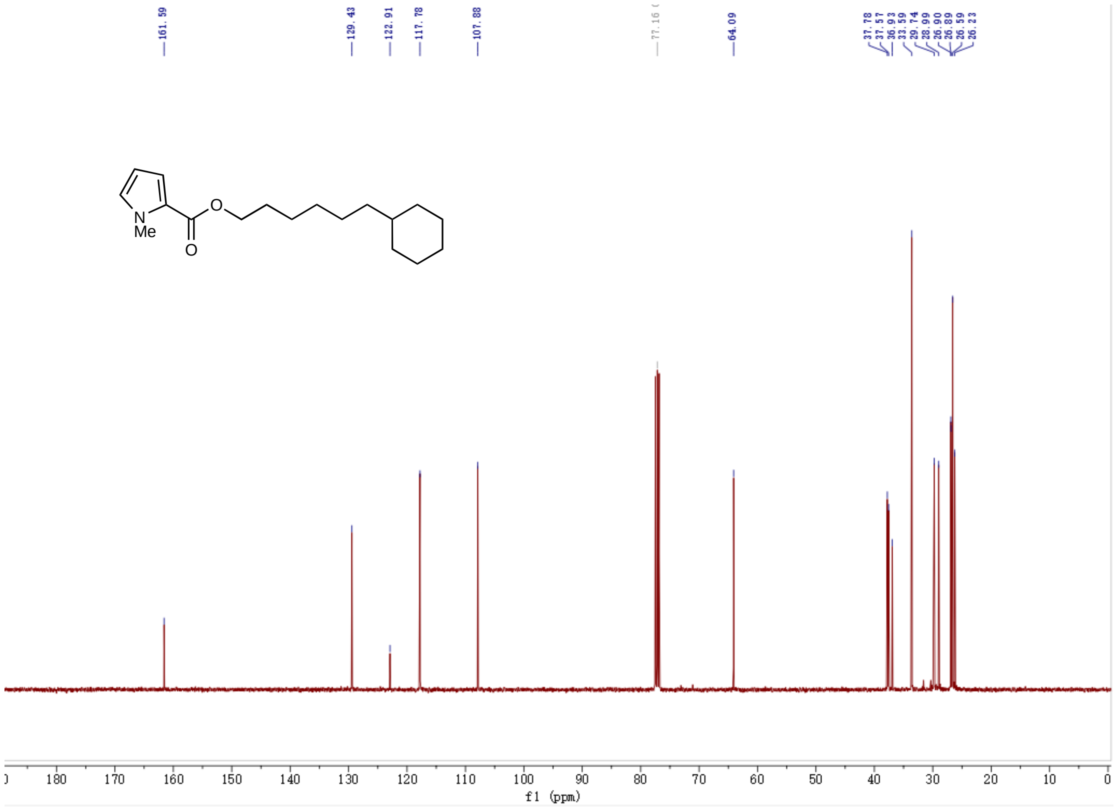

Supplementary Figure 190. ^{13}C NMR spectra of compound **8h**

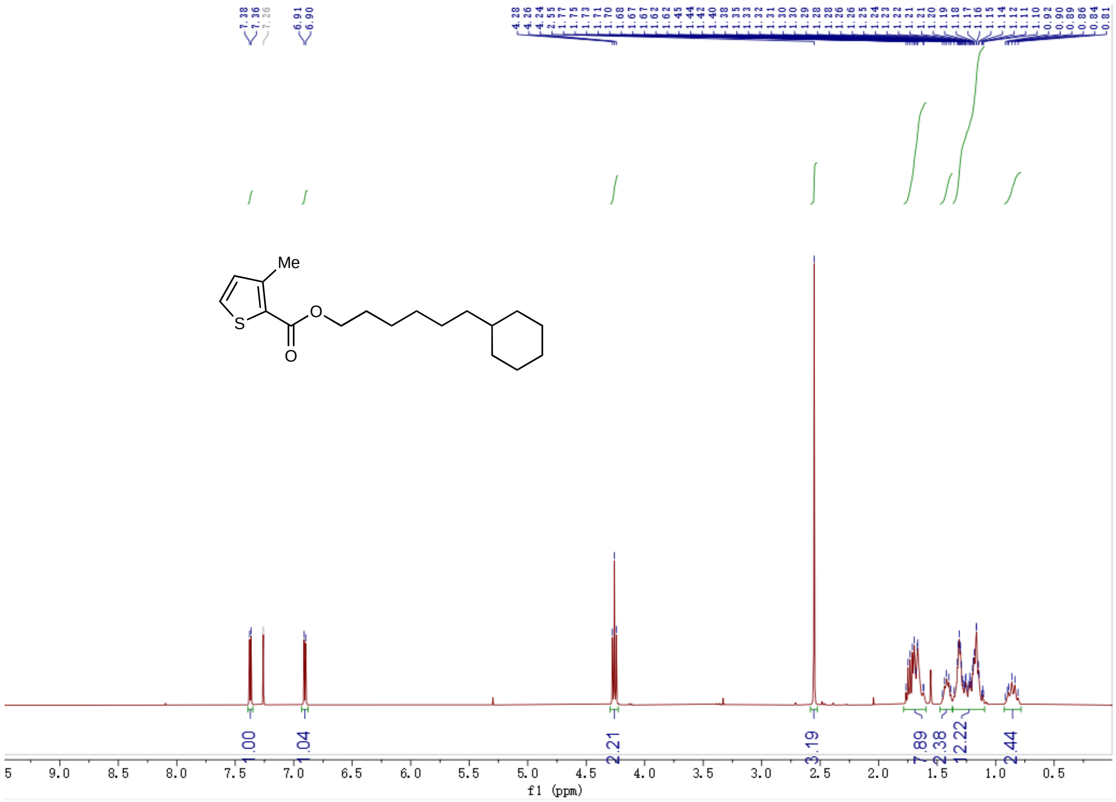

Supplementary Figure 191. ^{1}H NMR spectra of compound **8i**

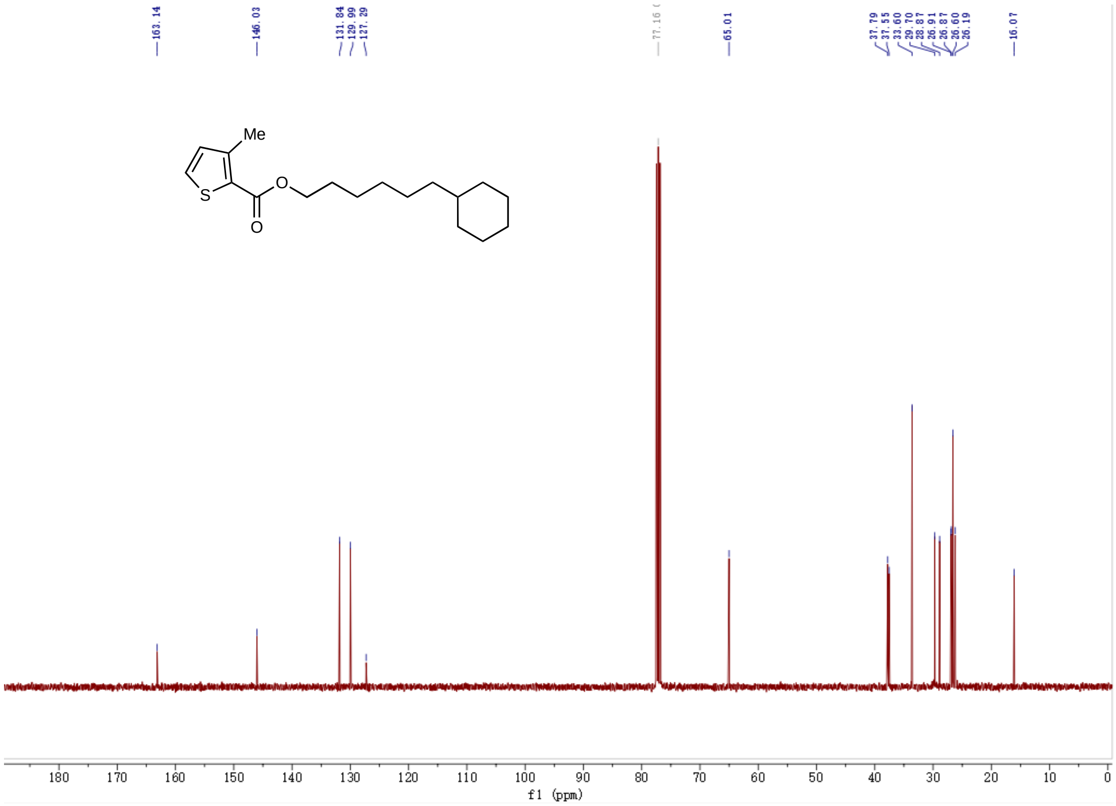

Supplementary Figure 192. ^{13}C NMR spectra of compound **8i**

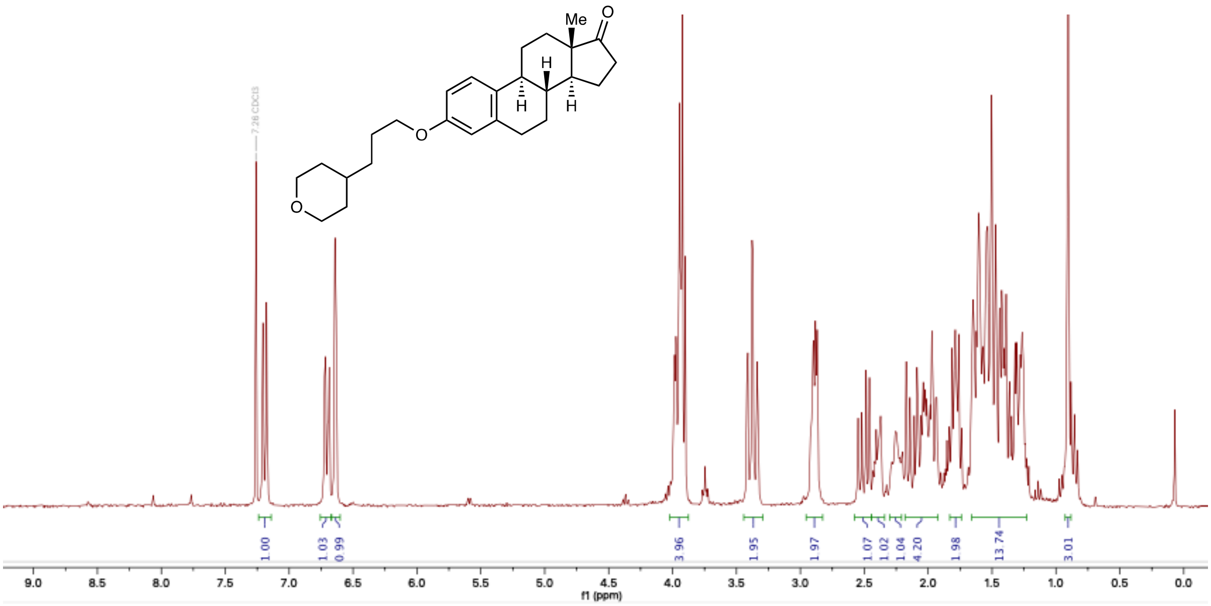

Supplementary Figure 193. ^{1}H NMR spectra of compound **8j**

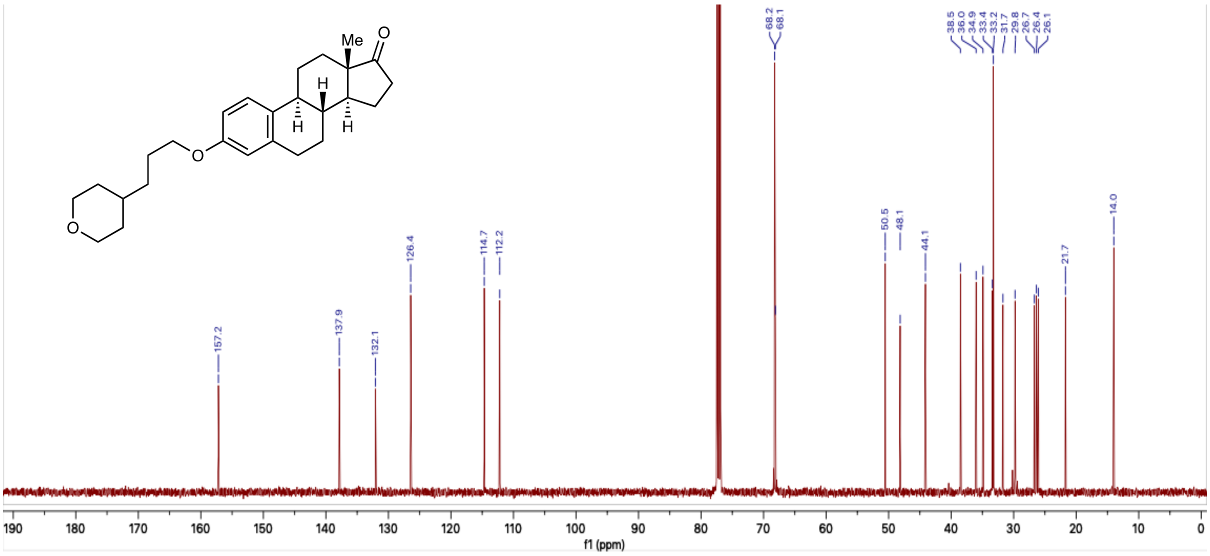

Supplementary Figure 194. ^{13}C NMR spectra of compound **8j**

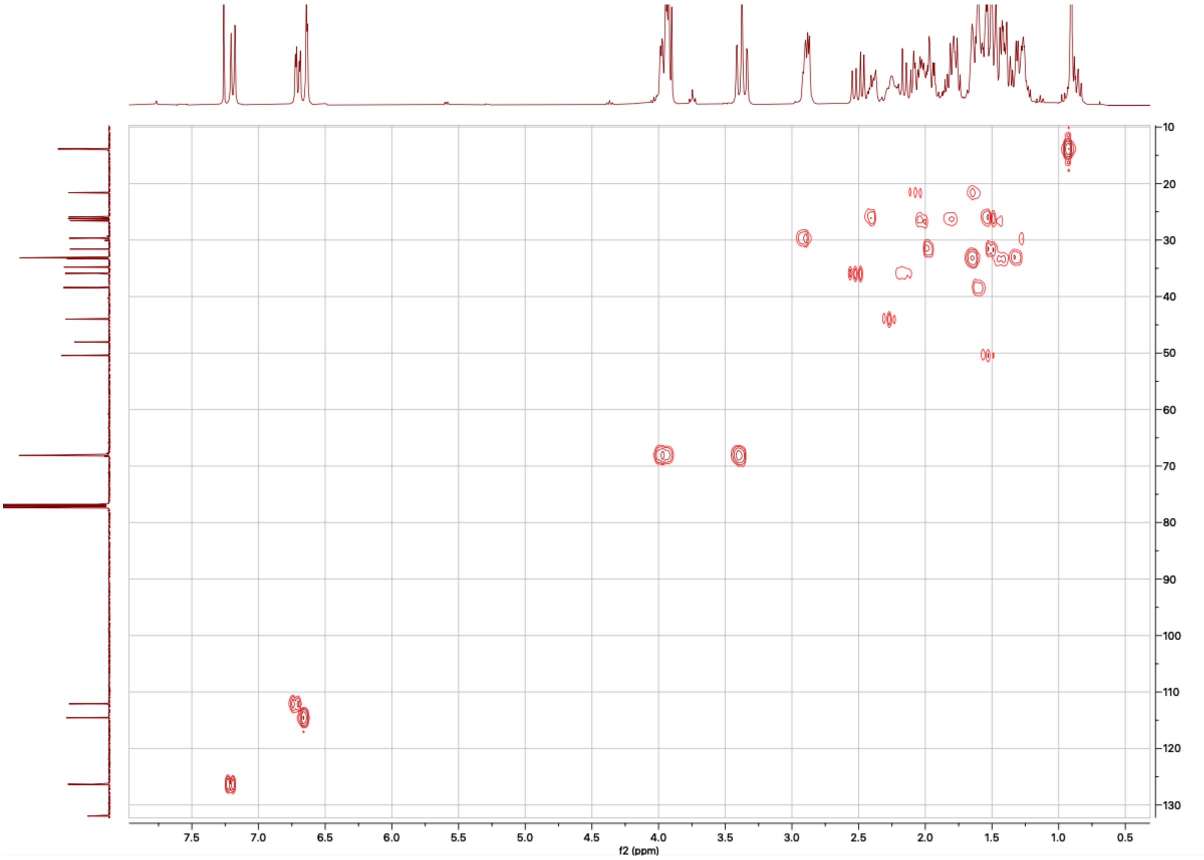

Supplementary Figure 195. HMQC NMR spectra of compound **8j**

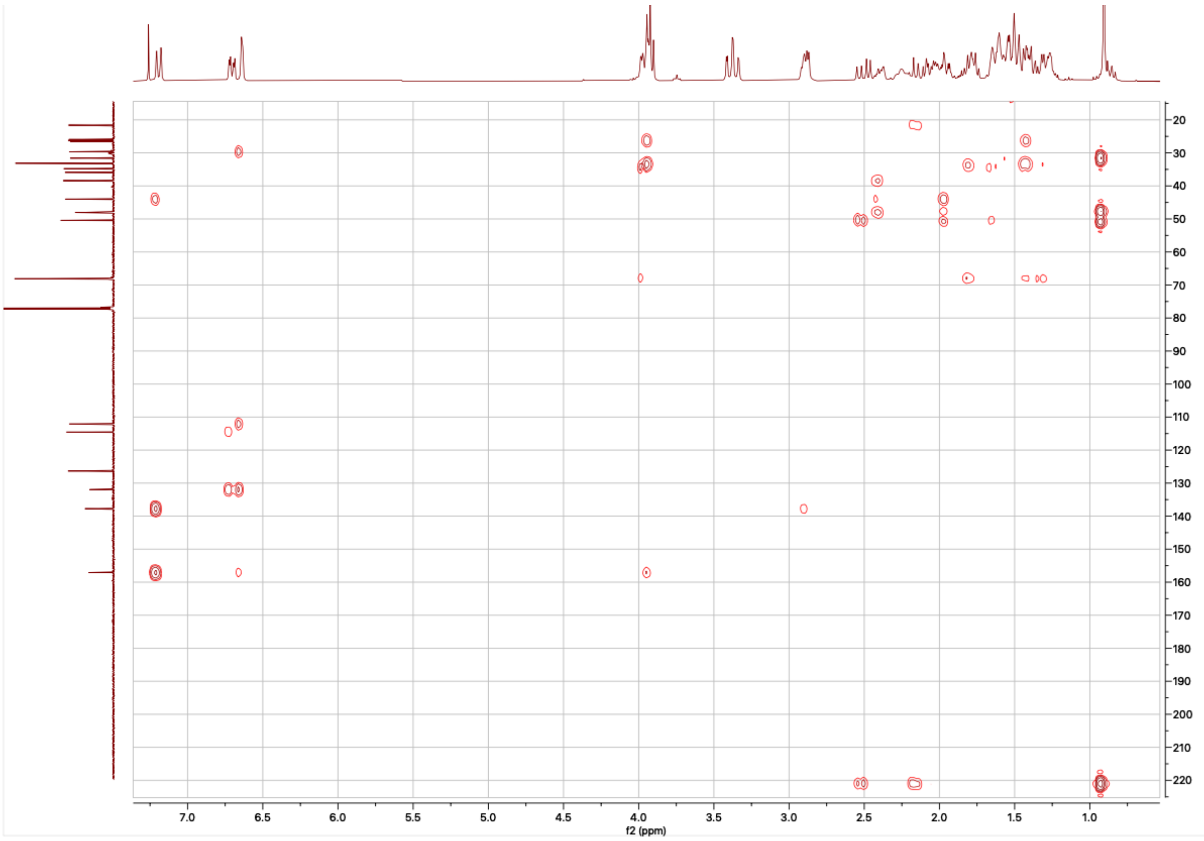

Supplementary Figure 196. HMBC NMR spectra of compound **8j**

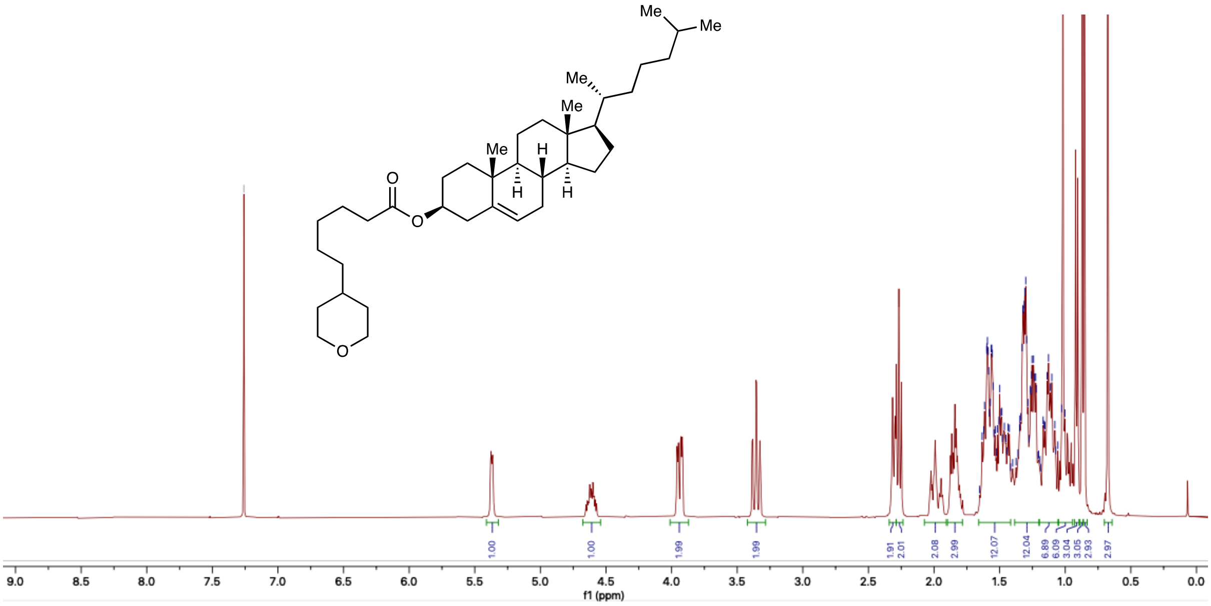

Supplementary Figure 197. ^{1}H NMR spectra of compound **8k**

^{13}C NMR of **8k**

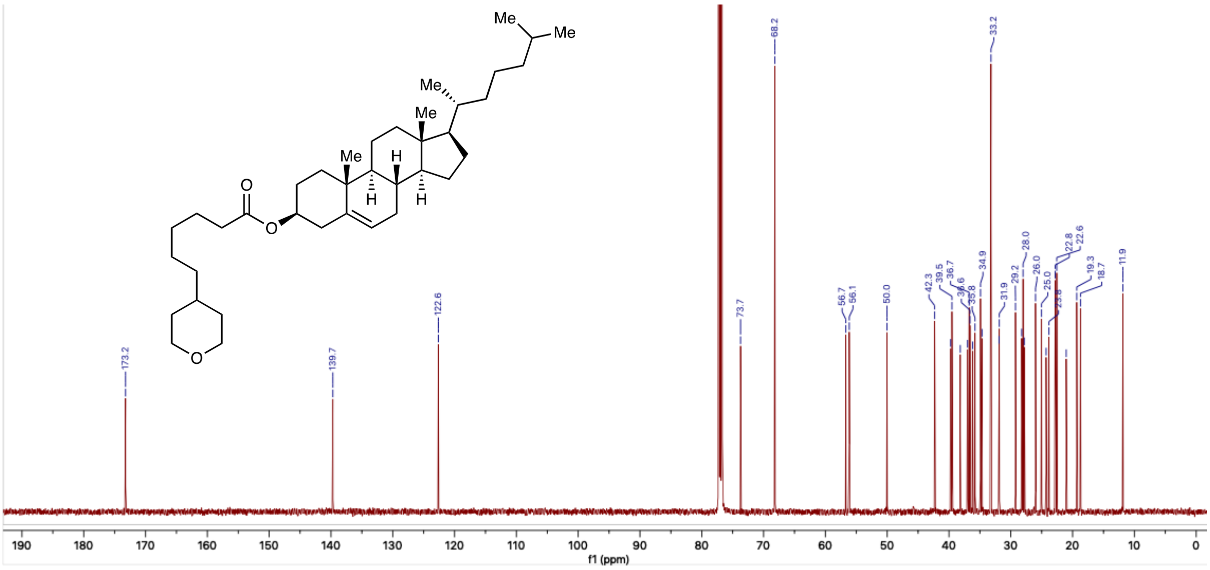

Supplementary Figure 198. ^{13}C NMR spectra of compound **8k**

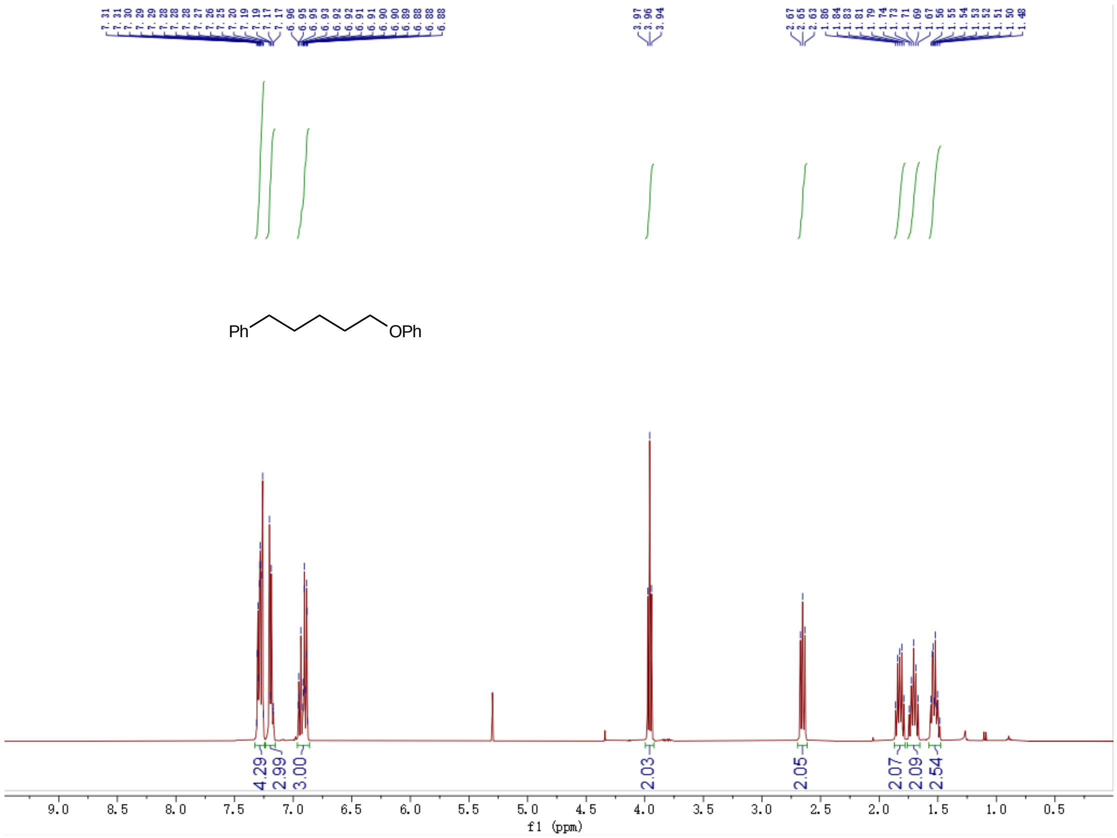

Supplementary Figure 199. ^{1}H NMR spectra of compound **8l**

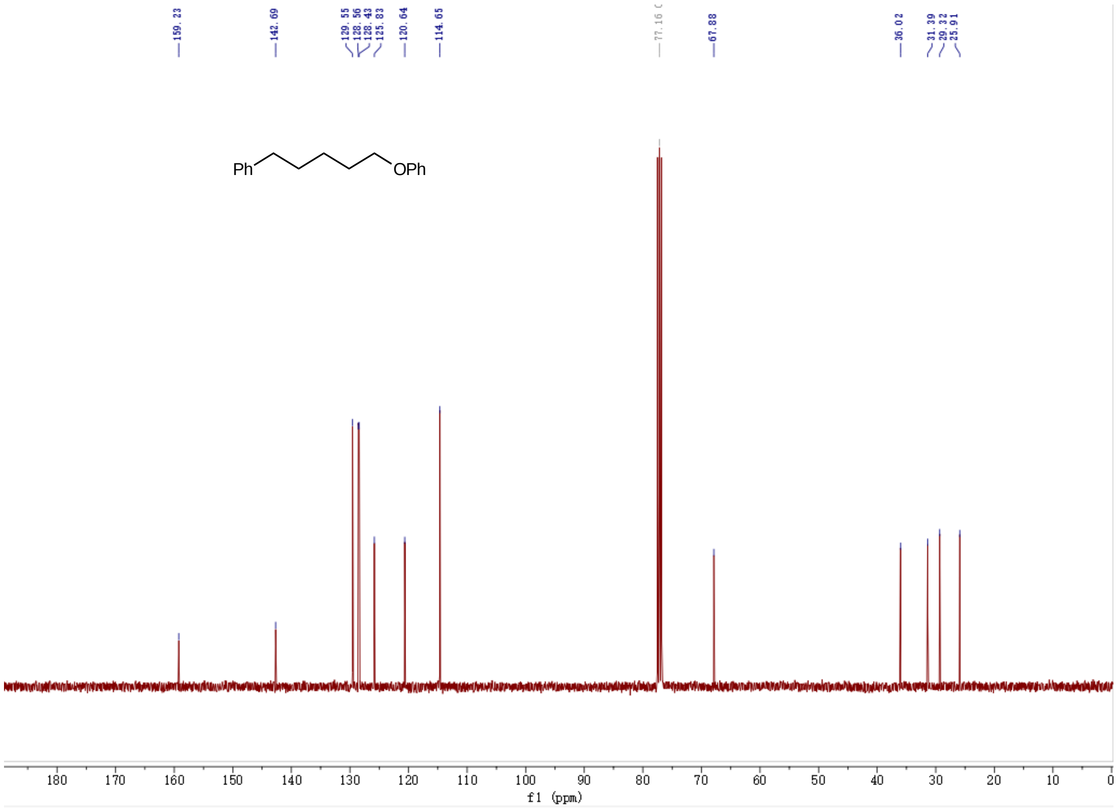

Supplementary Figure 200. ^{13}C NMR spectra of compound **8l**

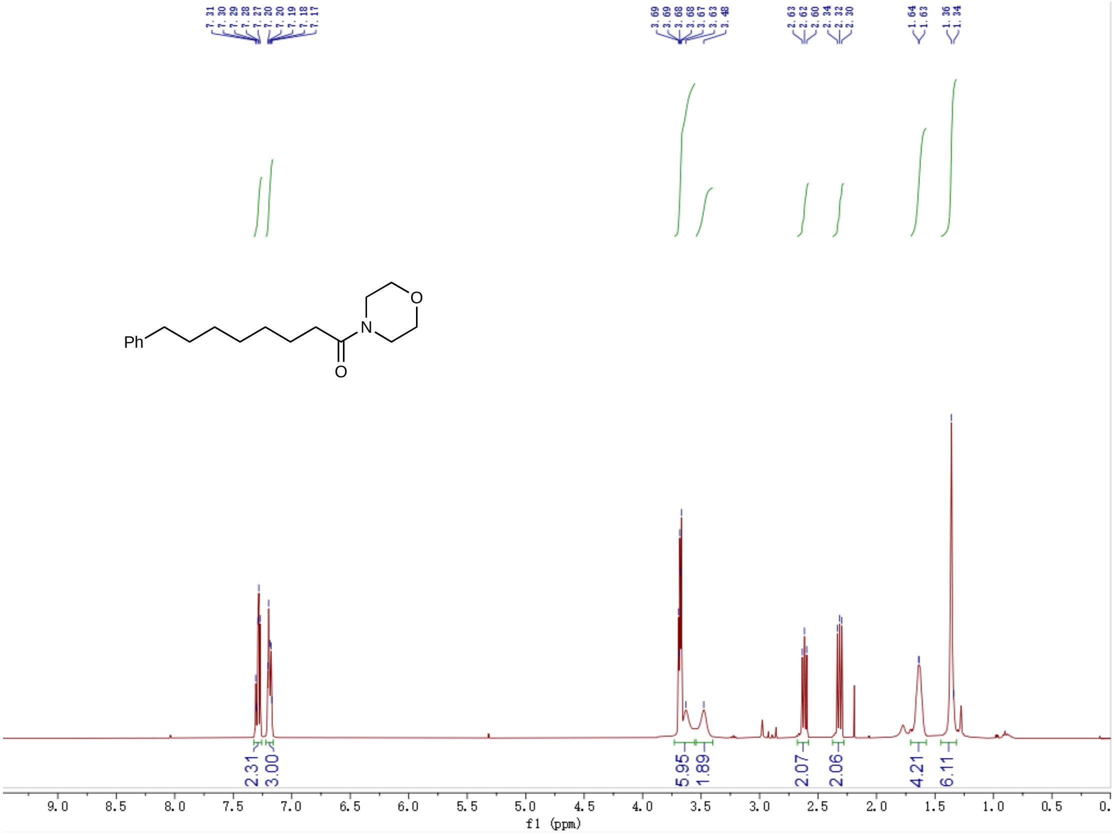

Supplementary Figure 201. ^{1}H NMR spectra of compound **8m**

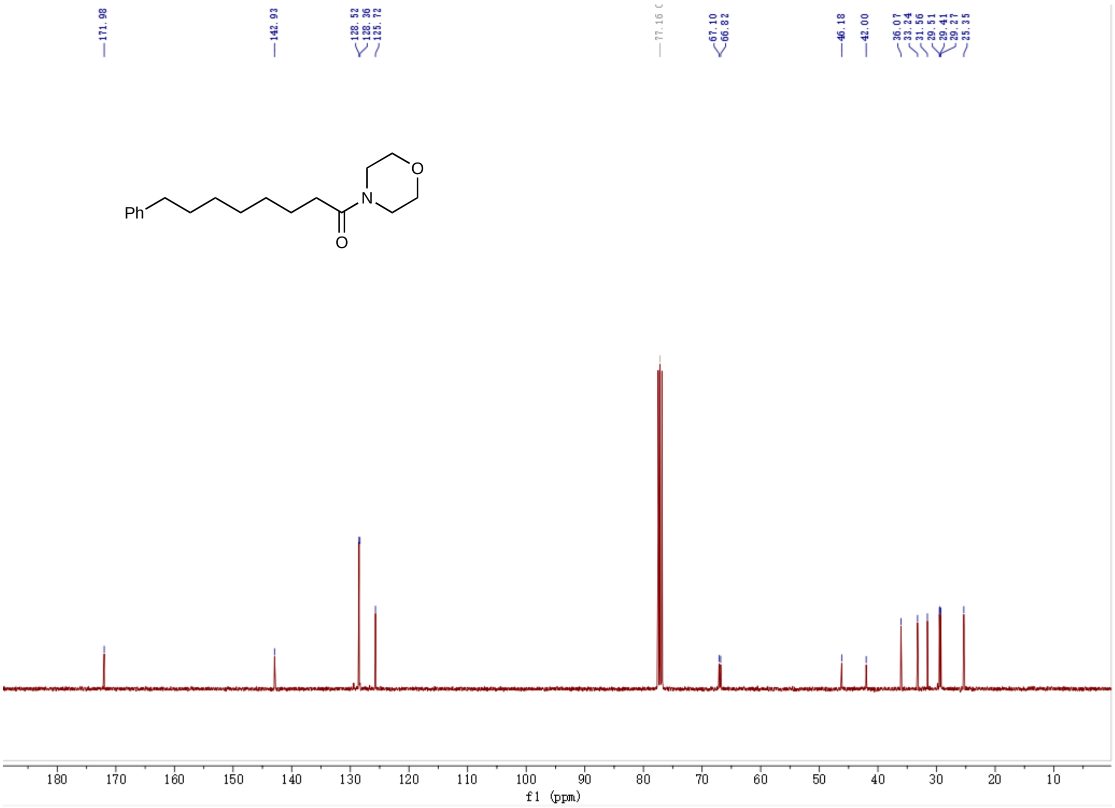

Supplementary Figure 202. ^{13}C NMR spectra of compound **8m**

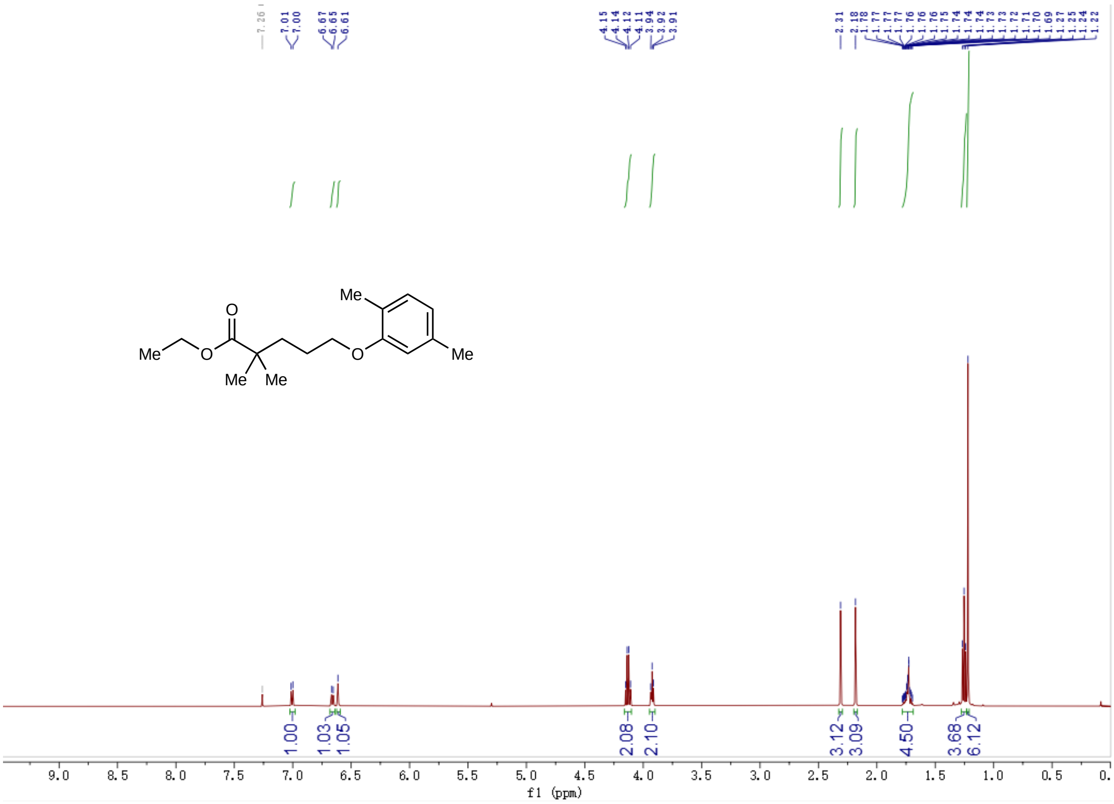

Supplementary Figure 203. ^{1}H NMR spectra of compound **8n**

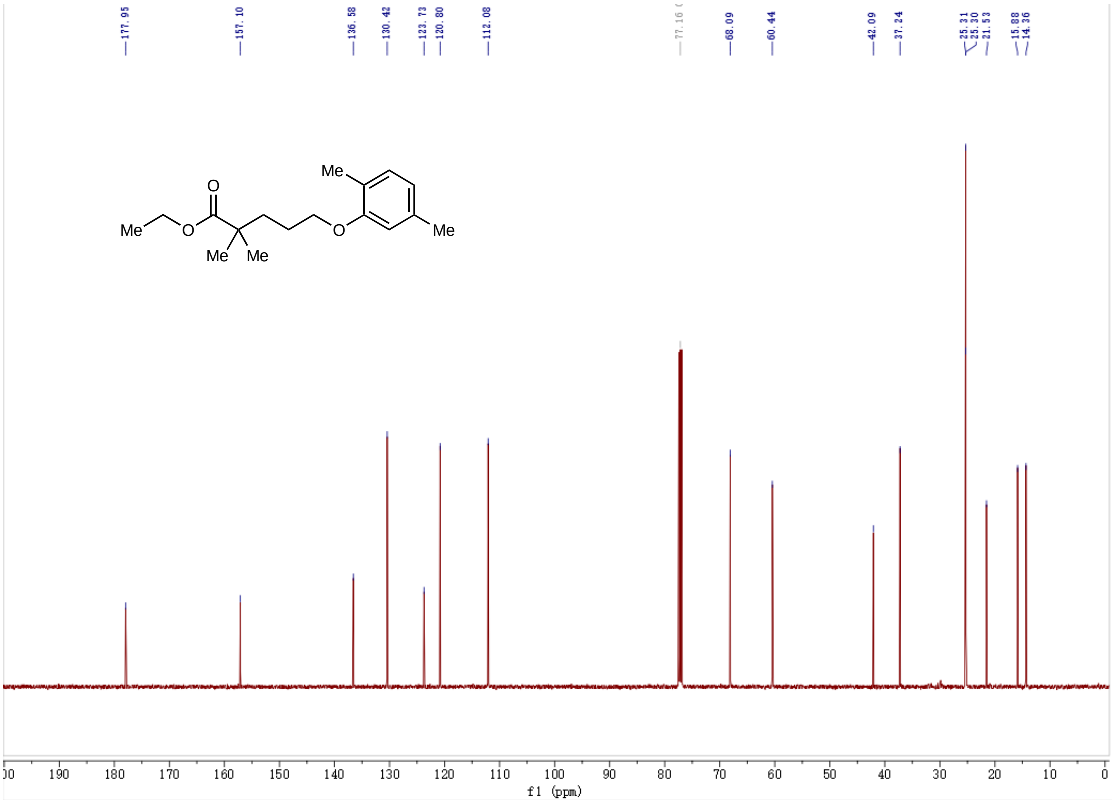

Supplementary Figure 204. ^{13}C NMR spectra of compound **8n**

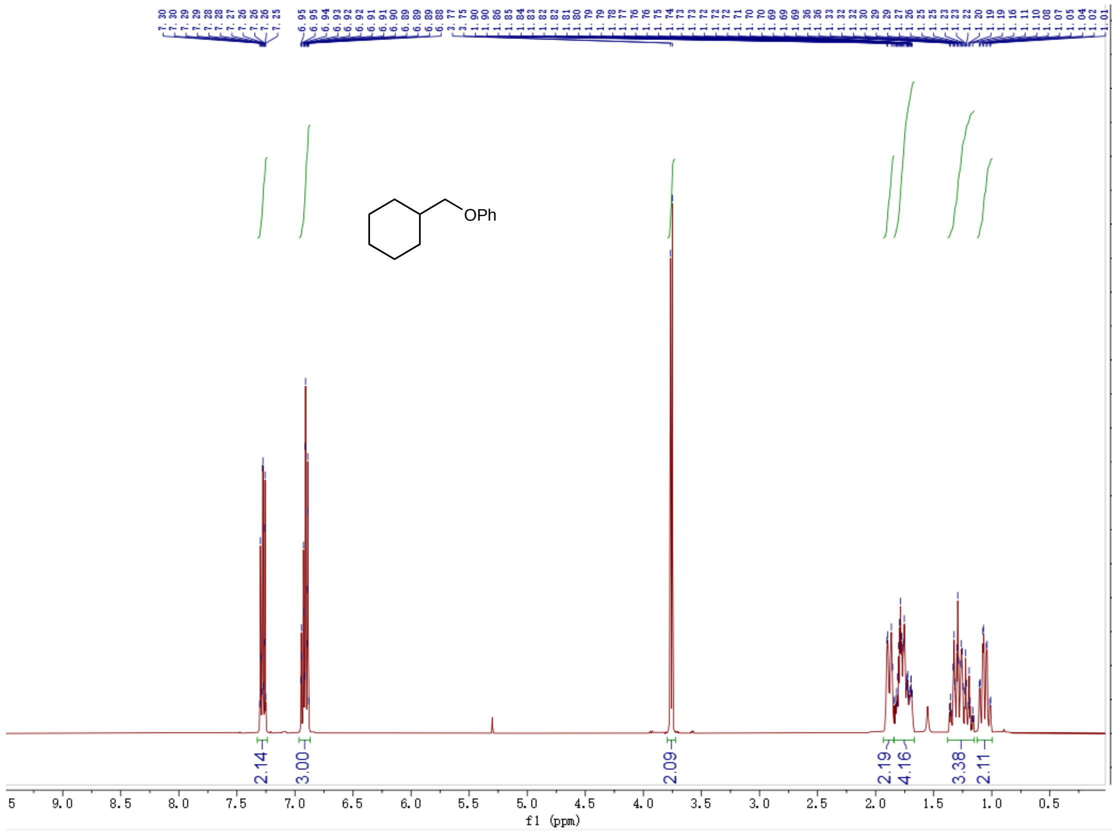

Supplementary Figure 205. ^{1}H NMR spectra of compound **8o**

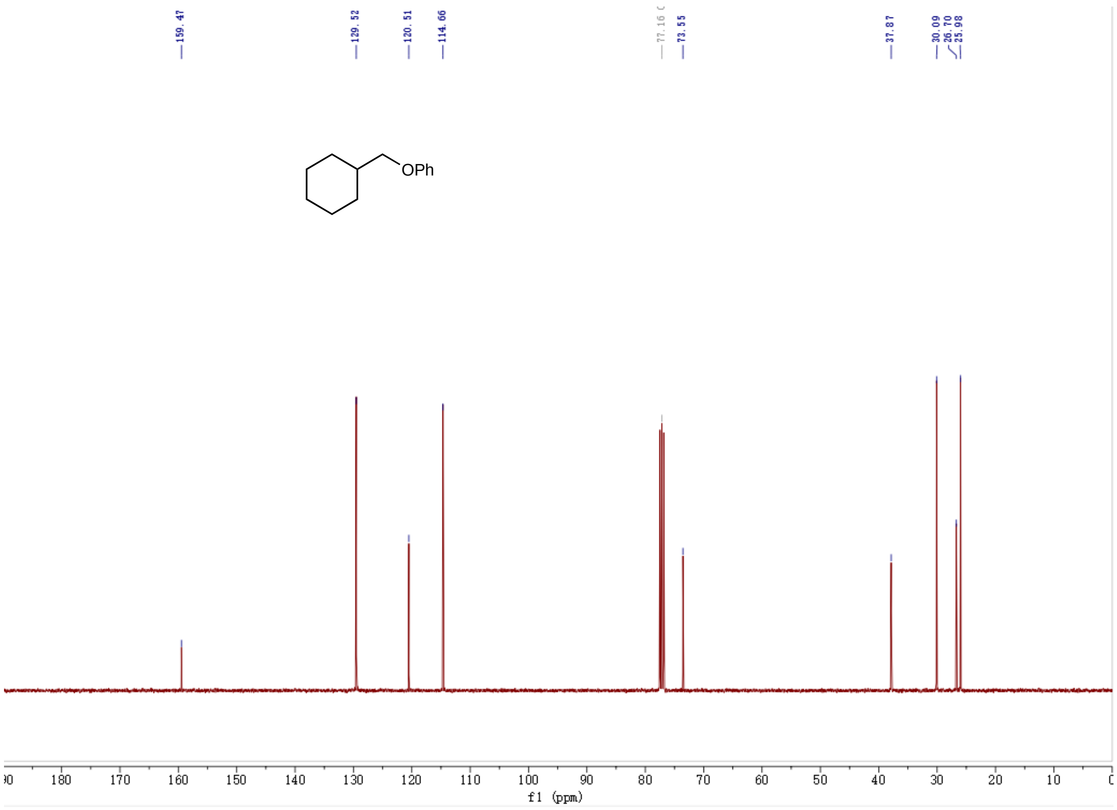

Supplementary Figure 206. ^{13}C NMR spectra of compound **8o**

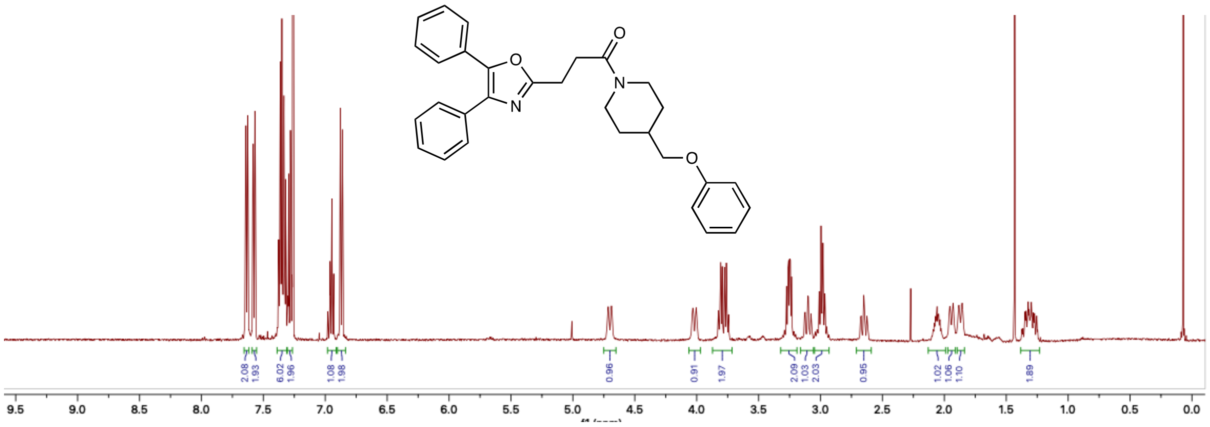

Supplementary Figure 207. ^{1}H NMR spectra of compound **8p**

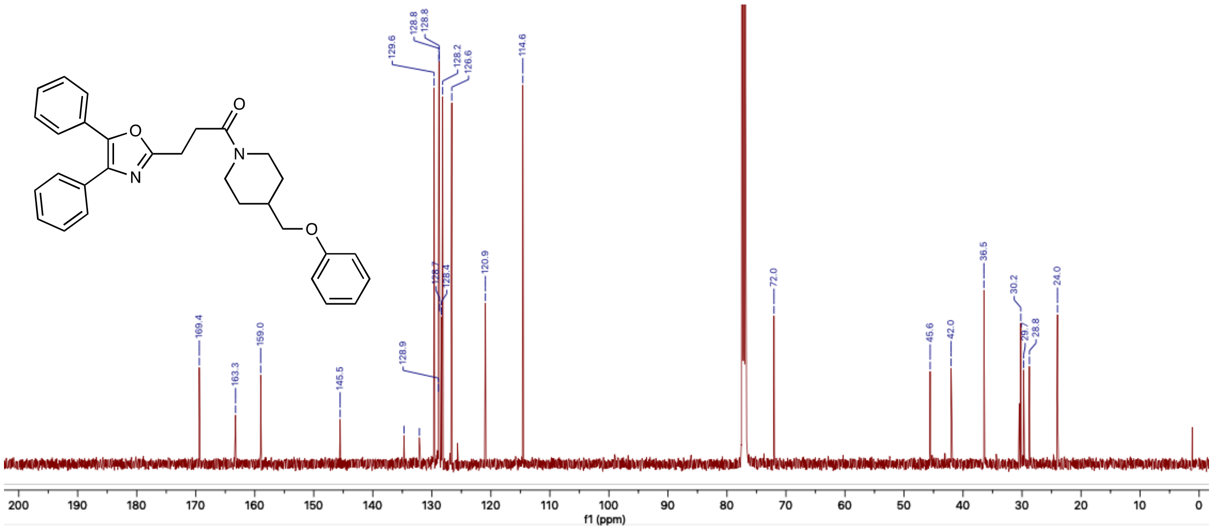

Supplementary Figure 208. ^{13}C NMR spectra of compound **8p**

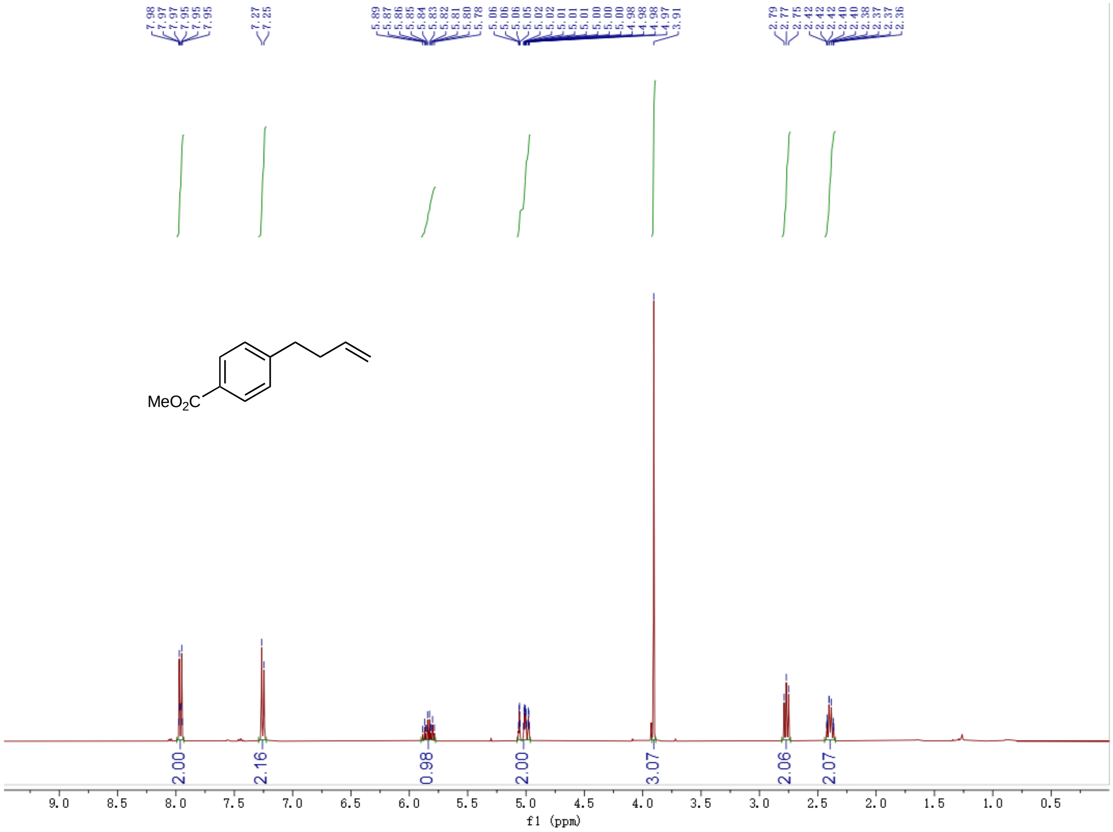

Supplementary Figure 209. ^{1}H NMR spectra of compound **10**

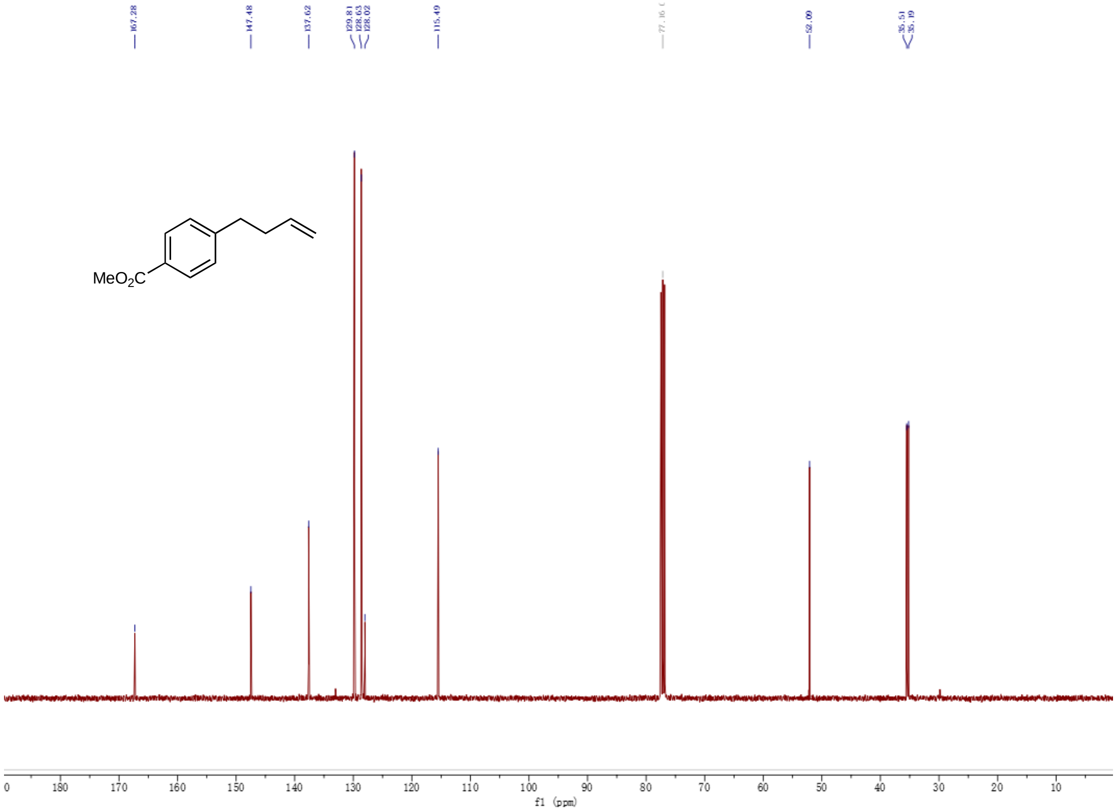

Supplementary Figure 210. ^{13}C NMR spectra of compound **10**

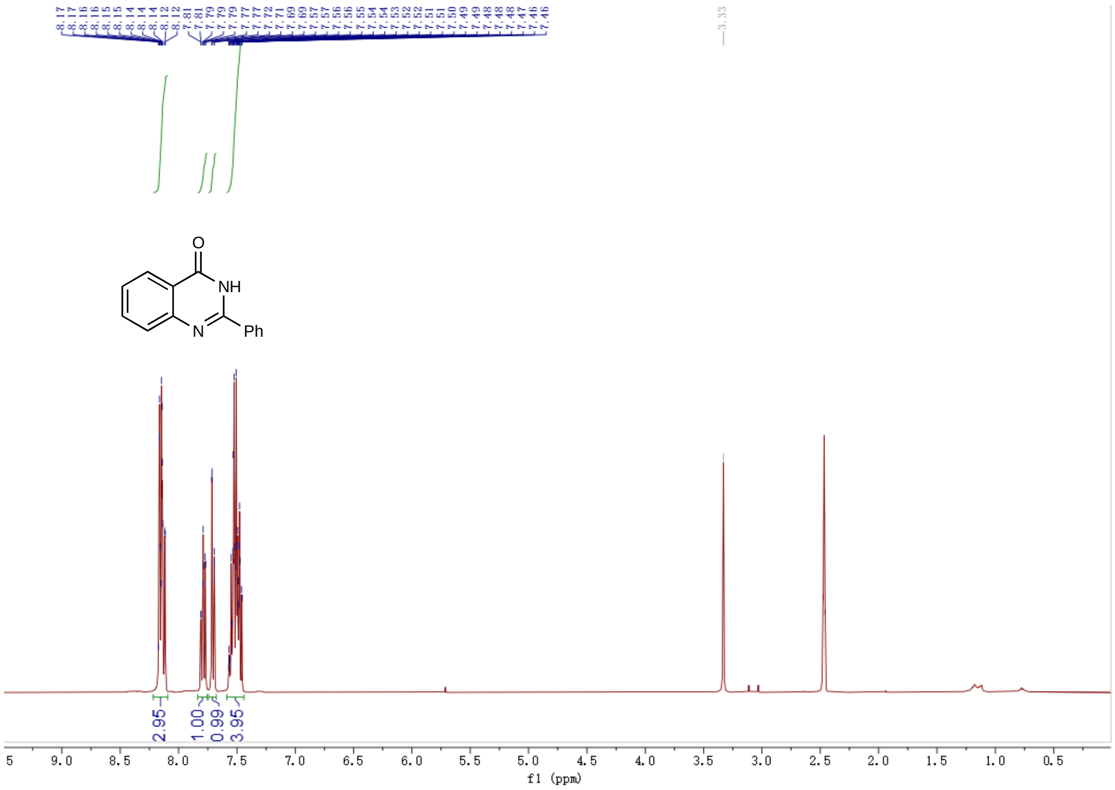

Supplementary Figure 211. ^{1}H NMR spectra of compound **11**

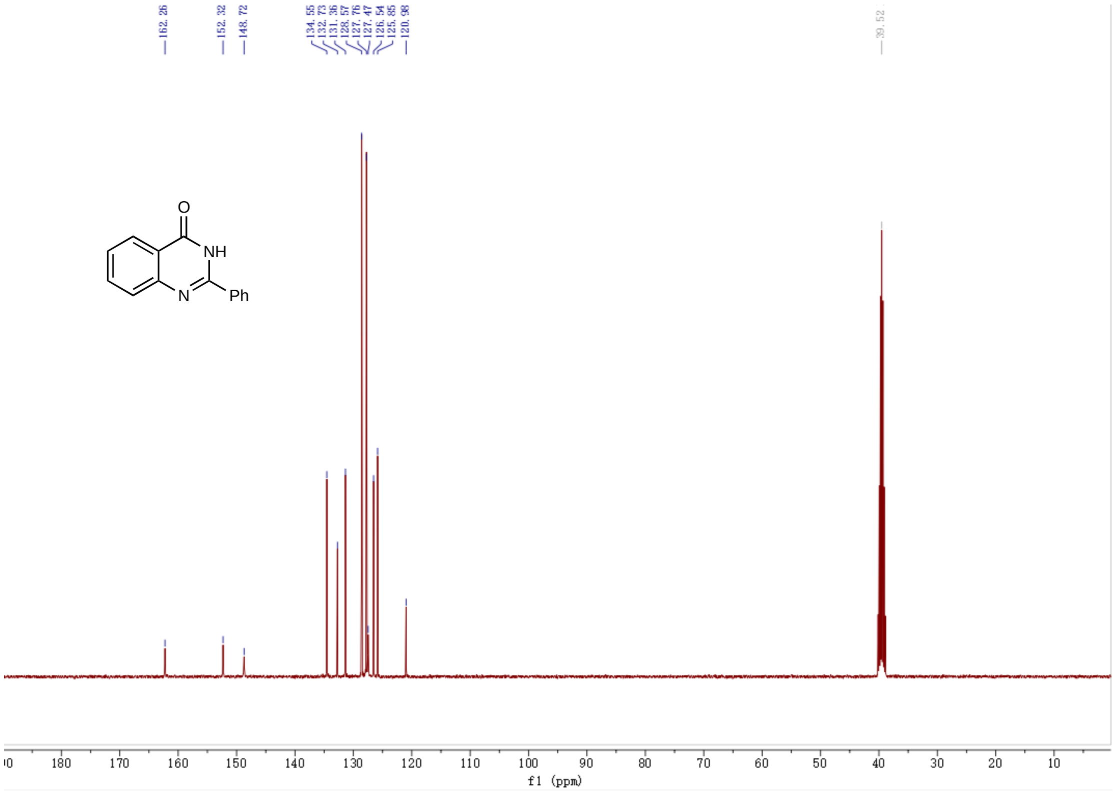

Supplementary Figure 212. ^{13}C NMR spectra of compound **11**

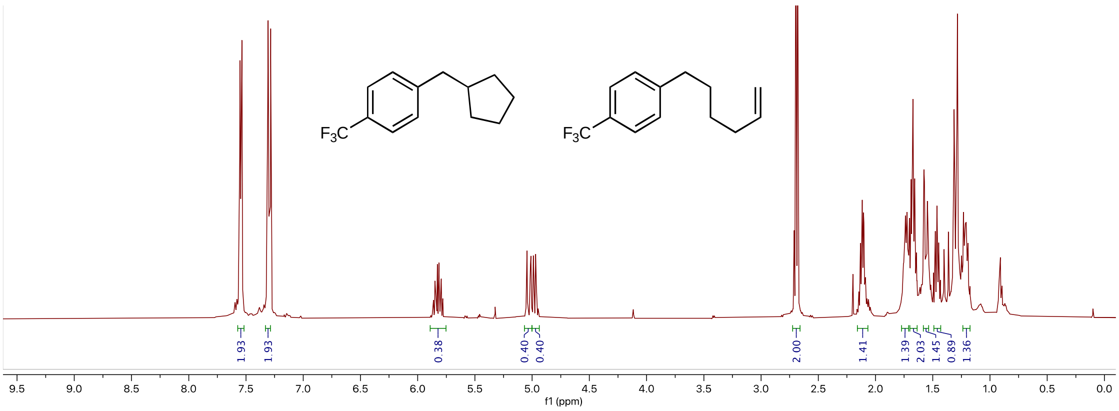

Supplementary Figure 213. ^{1}H NMR spectra of compound **12** and **13**

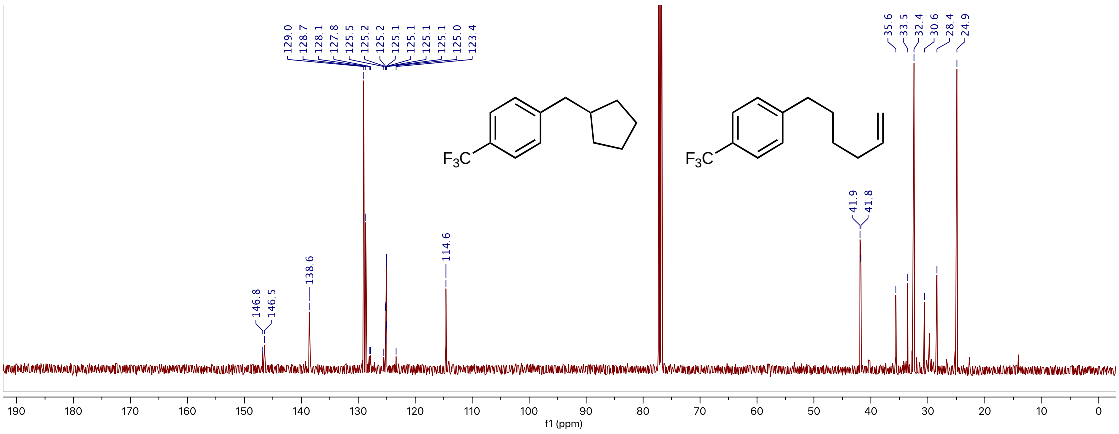

Supplementary Figure 214. ^{13}C NMR spectra of compound **12** and **13**

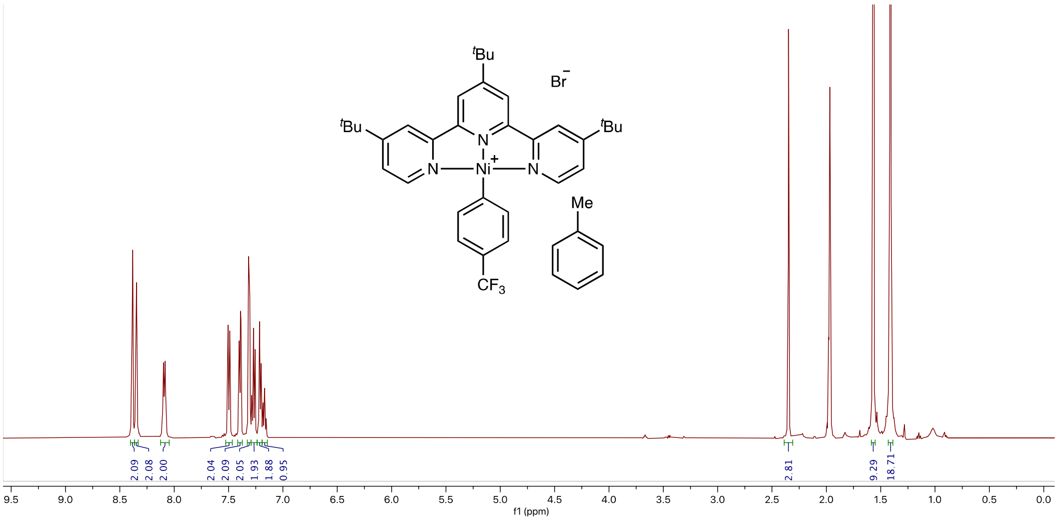

Supplementary Figure 215. ^{1}H NMR spectra of compound **Ni-I** in MeCN-d_3

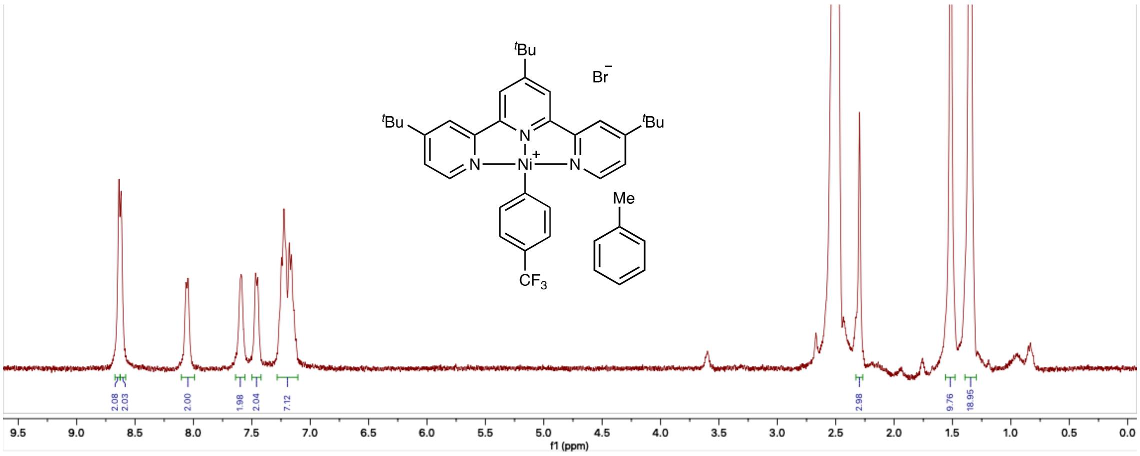

Supplementary Figure 216. ^{1}H NMR spectra of compound **Ni-I** in DMSO-d_6

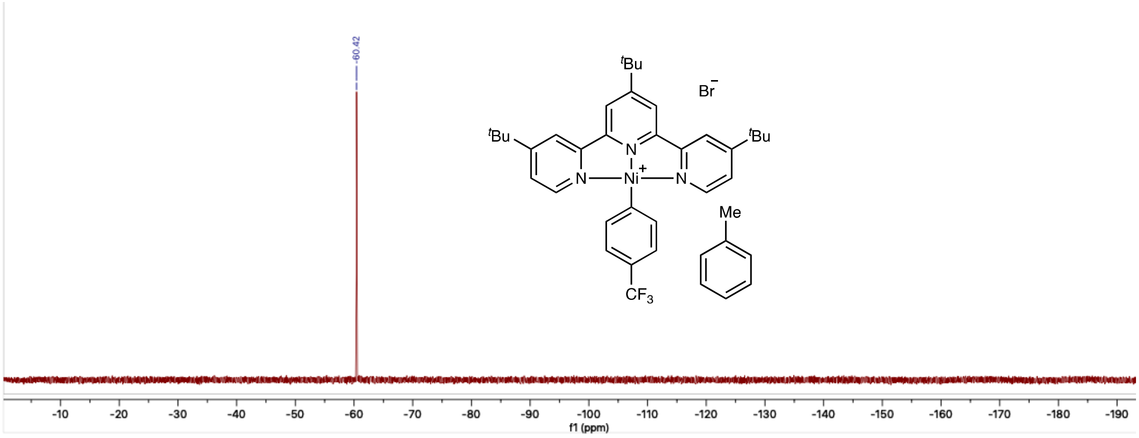

Supplementary Figure 217. ^{19}F NMR spectra of compound **Ni-I**

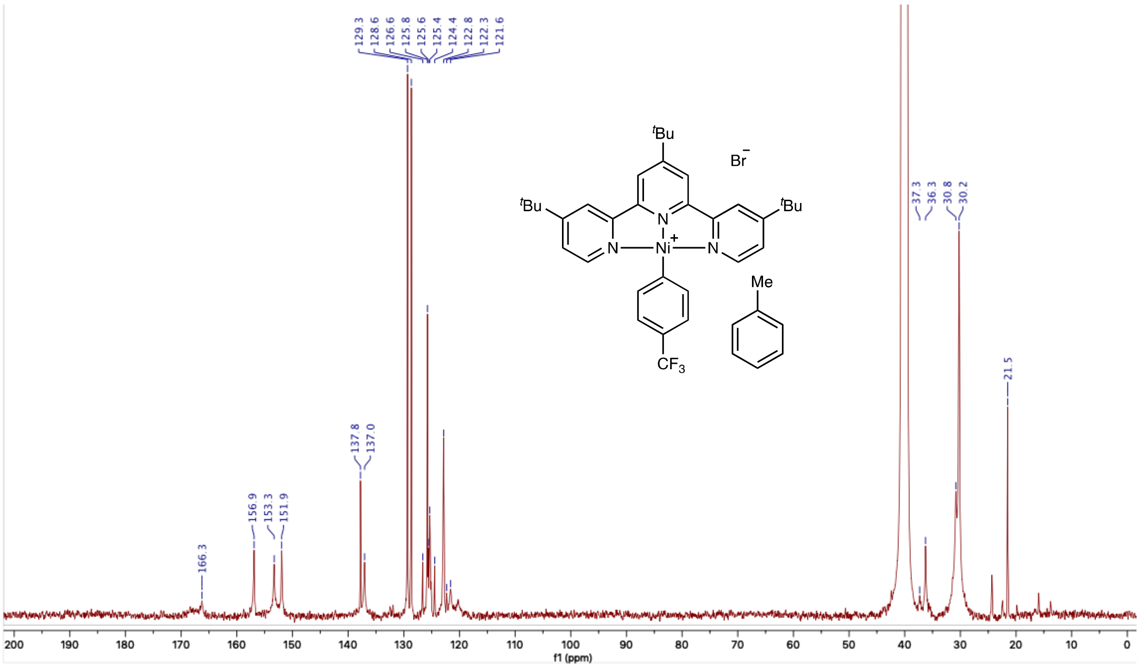

Supplementary Figure 218. ^{13}C NMR spectra of compound **Ni-I**

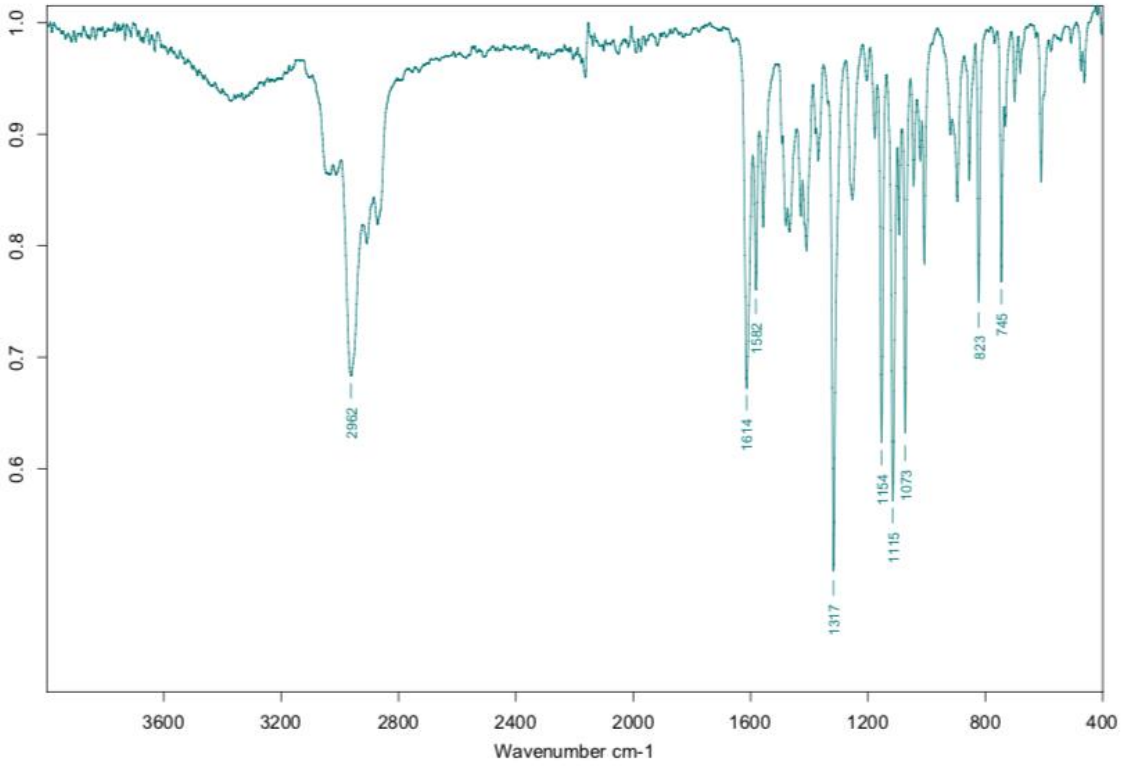

Supplementary Figure 219. IR spectra of compound **Ni-I**

Supplementary References

[1] Li, L.; Fang, L.; Wu, W.; Zhu, J. *Org. Lett*. **2020**, *22*, 5401.
[2] Wang, J.; Zhang, M,-M.; Wang, X.-S. *Res Chem Intermed*. **2017**, *43*, 2985.
[3] Soural, M.; Funk, P.; Kvapil, L.; Hradil, P.; Hlaváč, J.; Bertolasi, V. *ARKIVOC*, **2010**, *10*, 255.
[4] Ding, D.; Dong, H.; Wang, C. *Iscience*. **2000**, *23*, 101017.
[5] Bam, R.; Alexandros S. Pollatos, A. S.; Moser, A. J. West. J. G. *Chem. Sci*. **2021**, *12*, 1736.
[6] Jadhav, V. H.; Kim, J. G.; Jeong, H. J.; Kim, D. W. *J. Org. Chem*. **2015**, *80*, 7275.
[7] Theis, A.; Ritter, H.; *Macromolecules* **2003**, *36*, 7552.
[8] Wang, G.-W.; Wheatley, M.; Simonetti, M.; Cannas, D. M.; Larrosa, I., *Chem*, **2020**, *6*, 1459.
[9] Zhang, X.; MacMillan. D. W. C. *J. Am. Chem. Soc*. **2016**, *138*, 13862.
[10] Boudjelel, M.; Sadek, O.; Mallet-Ladeira, S.; García-Rodeja, Y.; Carrizo, E. D. S.; Miqueu, K.; Bouhadir, G.; Bourissou, D. *ACS Catal*. **2021**, *11*, 3822.
[11] Kawajiri,T.; Kato, M.; Nakata, H.; Goto, R.; Aibara,, S.; Ohta,, R.; Fujioka, H.; Sajiki, H.; Sawama, Y. *J. Org. Chem*. **2019**, *84*, 3853.
[12] Lee, Y. H.; Morandi, B. *Nature Chem*. **2018**, *10*, 1016.
[13] Chen, J.; Lin, J.-H.; Xiao, J.-C. *Chem. Commun*. **2018**, *54*, 7034.
[14] Zhang, P.; Le, C.; MacMillan, D. W. C. *J. Am. Chem. Soc*. **2016**, *138*, 8084.
[15] Ming, X.-X.; Tian, Z.-Y.; Zhang, C.-P. *Chem. Asian J*. **2019**, *14*, 3370.
[16] Eckert, P.; Organ, M. G. *Chem. Eur. J*. **2020**, *26*, 4861.
[17] Yue, H.; Zhu, C.; Shen, L.; Geng, Q.; Hock, K. J.; Yuan, T.; Cavallo, L.; Rueping, M. *Chem. Sci*. **2019**, *10*, 4430.
[18] Molander, G. A.; Canturk. B.; *Org. Lett*. **2008**, *10*, 2135.
[19] Qin, X.; Ding, G.; Gong, Y.; Jing, C.; Peng, G.; Liu, S.; Niu, L.; Zhang, S.; Luo, Z.; Li, H.; Gao, F.; *Dyes and Pigments*. **2016**, *132*, 27.
[20] Cong, F.; Lv. X.-Y.; Martin, R. *J. Am. Chem. Soc*. **2020**, *142*, 20594.
[21] Bering, L.; Jeyakumar, K.; Antonchick, A. P. *Org. Lett*. **2018**, *20*, 3911.
[22] Zhang, Y.-L.; Yang. L.; Wu, J.; Zhu, C.; Wang, P. *Org. Lett*. **2020**, *22*, 7768.
[23] Sun, S.-Z.; Romano, C.; Martin, R. *J. Am. Chem. Soc*. **2019**, *141*, 16197.
[24] Bhunia, A.; Bergander, K.; Studer, A. *J. Am. Chem. Soc*. **2018**, *140*, 16353.
[25] Gao, Y.; Yang, C.; Bai, S.; Liu, X.; Wu, Q.; Wang, J.; Jiang, C.; Qi, X. *Chem*. **2020**, *6*, 675.
[26] Sugataa, H.; Tsubogoa, T.; Kinoa, Y.; Uchiro, H. *Tetrahedron Lett*. **2017**, *58*, 1015.
[27] Primer, D. N.; Molander, G. A. *J. Am. Chem. Soc*. **2017**, *139*, 9847−9850
[28] Takeda, H.; Takeda, M.; Yoshioka, H.; Minamide, H.; Oki, Y.; Adachi, C.; *Opt. Mater. Express* **2019**, *9*, 1150.
[29] Das, S.; Mondal, R.; Chakraborty, G.; Guin, A. K.; Das, A.; Paul, N. D. *ACS Catal*. **2021**, *11*, 7498.
